# DNA damage response profile distinguishes poor-acting gliomas with shared methylome signatures

**DOI:** 10.1093/neuonc/noaf199

**Published:** 2025-08-27

**Authors:** Nalin Leelatian, Charu Singh, Richard Bouffard, Ranjini K Sundaram, Kirsten E Diggins, William Sullivan, Sateja Paradkar, Zeynep Erson Omay, Bret C Mobley, Susan E Gueble, Juan C Vasquez, Ranjit S Bindra

**Affiliations:** Department of Pathology, Microbiology, and Immunology, Vanderbilt University School of Medicine, Nashville, TN, USA; Department of Pathology, Yale School of Medicine, New Haven, CT, USA; Department of Therapeutic Radiology, Yale School of Medicine, New Haven, CT, USA; Department of Pathology, Yale School of Medicine, New Haven, CT, USA; Department of Therapeutic Radiology, Yale School of Medicine, New Haven, CT, USA; Cyclo Technologies, Roswell, GA, USA; Department of Therapeutic Radiology, Yale School of Medicine, New Haven, CT, USA; Department of Therapeutic Radiology, Yale School of Medicine, New Haven, CT, USA; Department of Neurosurgery, Yale School of Medicine, New Haven, CT, USA; Department of Pathology, Microbiology, and Immunology, Vanderbilt University School of Medicine, Nashville, TN, USA; Department of Therapeutic Radiology, Yale School of Medicine, New Haven, CT, USA; Department of Pediatric Hematology and Oncology, Yale School of Medicine, New Haven, CT, USA; Department of Therapeutic Radiology, Yale School of Medicine, New Haven, CT, USA; Department of Pathology, Yale School of Medicine, New Haven, CT, USA

**Keywords:** DNA damage response, glioma, methylation, single cell, transcriptomics

## Abstract

**Background:**

Therapies for diffuse glioma induce DNA damage response (DDR), and strategies to exploit DDR defects are active areas of investigation. While global DNA methylation profiling effectively classifies gliomas into subtypes, the epigenetic and gene expression patterns of DDR genes, and their contribution to tumor classification and outcomes, have yet to be fully elucidated. Thus, dissecting the DDR epigenetics, gene expression, and single-cell heterogeneity may reveal key molecular characteristics, refine prognosis, and identify novel treatment strategies and resistance mechanisms.

**Methods:**

We characterized DDR epigenetics and gene expression of TCGA glioblastomas (GBM) and low-grade gliomas (LGG). Single-cell protein analysis by imaging mass cytometry (IMC) was performed on a separate cohort of 118 diffuse gliomas.

**Results:**

Analysis of TCGA cohorts revealed two DDR methylation groups that correlated with *IDH* mutation status and previously reported molecular groups. DDR transcription profiling further classified tumors into four groups. Those with high DDR transcription across pathways were linked to poor survival independent of *IDH* or *MGMT* status, and potentially improved prognostication beyond established biomarkers. Single-cell characterization of a separate cohort revealed intratumoral DDR diversity and identified proliferative tumor cells with high DDR protein expression across pathways that are associated with unfavorable grade and survival.

**Conclusions:**

Tumor-level epigenetic and transcriptional DDR signatures alone can distinguish molecular-defined diagnosis and outcomes of gliomas beyond established biomarkers. A higher abundance of glioma cells with high DDR effector expression across pathways is associated with poor survival. Thus, clinical assessment of pan-DDR expression may inform prognosis and identify potential therapeutic targets.

**Key Points**

- DDR methylomics and transcriptomics are linked to glioma molecular diagnoses and outcomes.
- The presence of glioma cell subsets with high DDR gene expression predicts unfavorable survival.

**Importance of the Study**

This study showed that the molecular grades and clinical outcome of diffuse gliomas are intricately linked to the DNA methylation and transcriptional profiles of DDR genes. We also demonstrated the impact of DDR profiles toward clinical stratification beyond clinically established biomarkers. We illustrated the diversity of DDR effector protein expressions within individual gliomas, and the presence of cell subsets with high DDR expression that further refine clinical outcomes.

DNA damage response (DDR) pathways maintain genomic stability by sensing and repairing lesions that occur during replication or from extrinsic insults, including chemoradiation. While distinct pathways respond to specific types of DNA lesions, many DDR proteins participate in overlapping pathways to maintain genome integrity. Excessive DNA damage can induce cell cycle arrest and death, a mechanism exploited by cancer therapies that disrupt DNA replication or synthesis. Tumors with defects in specific DDR pathways may exhibit differential sensitivity to DNA damaging agents, highlighting the importance of understanding DDR landscapes to predict resistance and guide treatment decisions.

Diffuse glioma is the most common primary malignant brain tumor in adults.^1^ Standard care includes maximal surgical resection followed by radiation and alkylating chemotherapy, both of which depend on DDR function.^2^ Notably, O^*6*^-methylguanine-DNA methyltransferase (MGMT) acts as a “suicide” repair enzyme that removes alkyl groups deposited at O^*6*^-guanine by temozolomide.^3^ Therefore, epigenetic silencing of *MGMT* expression, common in *IDH*-mutant gliomas and occurring in nearly half of *IDH*-wildtype glioblastoma (GBM), serves as a predictive biomarker for temozolomide.^2,4–7^ However, temozolomide resistance can often arise from the development of mismatch repair (MMR) deficiencies, with MMR protein expression showing intratumoral diversity within gliomas at the single-cell level.^8,9^ Despite the complexity and importance of DDR, assessments beyond *MGMT* methylation are not routinely performed.^3,10,11^

DNA methylation profiling is used diagnostically to classify gliomas into prognostic categories.^5,12–15^ However, the influence of DDR gene signatures toward these classifications is unclear, despite their critical associations with glioma therapies.^2,16,17^ While studies have shown the value of bulk-tumor molecular profile,^5,18^ single-cell studies have demonstrated intratumoral heterogeneity, dynamic clonal evolution, and the presence of prognostically significant cell subsets.^19–24^ Single-cell diversity of DDR expression is present across different tumor types, including gliomas.^9,25–28^ Therefore, we leveraged bulk-tumor epigenetic and transcriptomic profiling of DDR genes and single-cell protein quantitation of DDR effectors to dissect the contribution of DDR pathways toward the classification and clinical stratification of diffuse glioma.

## Material and Methods

### TCGA Data Access

The Cancer Genome Atlas (TCGA) Genomic Data Commons’ glioblastoma (TCGA-GBM) and low-grade glioma (TCGA-LGG) datasets were downloaded using R package TCGAbiolinks on July 31^st^, 2022.^29^ Cases with either DNA methylation or gene expression data (792 samples) from the Harmonized database (reference genome hg38) were retrieved (689 samples with methylation data, 706 samples with gene expression data). Of these, 593 cases had both methylation and gene expression data. Clinical outcome data were obtained from cBioPortal on November 20, 2022.^30^

### TCGA Diagnosis Revision

Two neuropathologists reviewed lineage-defining alterations (*IDH* mutation and 1p/19q codeletion status) and grade-defining molecular changes of the TCGA gliomas included in this study (combined gain of chromosome 7 and loss of chromosome 10 (+ 7/-10), *TERT* promoter mutation, *EGFR* amplification, and *CDKN2A/B* deletion) (Supplementary Table S1). Previously reported molecular classes, including *MGMT* promoter methylation status, were obtained from an annotated dataset.^5^ Patient demographics were obtained from cBioPortal.^30,31^ The original pathologist-reported histologic descriptions were reviewed. The algorithm for diagnosis revision followed the fifth edition of the World Health Organization Classification of Central Nervous System Tumors (Supplementary Figure S1a).^32^ Three *IDH*-wildtype cases that were previously classified as G34 (2 cases) and K27 (1 case) were excluded.^5^ Diagnosis of “GBM, WHO grade 4” was assigned to cases that were previously classified as GBM and were *IDH*-wildtype (*n* = 233, 32.4%). For *IDH*-wildtype cases that were previously assigned either grade 2 or 3 designations, the presence of GBM-defining molecular alterations (+ 7/-10, *TERT* promoter mutation, and/or *EGFR* amplification) or unequivocal documentation of vascular proliferation/hyperplasia and/or necrosis was considered sufficient for the diagnosis of GBM (*n* = 72, 9.1%). Equivocal microscopic description terminologies (eg, subtle, early) were considered not definitive and insufficient for a grade 4 designation. *IDH*-wildtype cases not meeting histologic criteria for GBM were reviewed for GBM-defining molecular alterations, and, if negative, were assigned to “glioma, *IDH*-wildtype, not elsewhere classified (NEC)” (*n* = 13, 1.6%).^33^  *IDH*-wildtype cases not meeting the histologic criteria for GBM and lacking full assessment of GBM-defining molecular alterations were assigned to “glioma, *IDH*-wildtype, not otherwise specified (NOS)” (*n* = 10, 1.3%). For subsequent analyses, NEC and NOS cases were combined.

*IDH*-mutant cases with 1p/19q codeletion were classified as “oligodendroglioma, *IDH-*mutant” (*n* = 172, 21.7%), whereas those without 1p/19q codeletion were classified as “astrocytoma, *IDH-*mutant” (*n* = 275, 34.7%). Astrocytoma, *IDH-*mutant cases with either *CDKN2A/B* deletion or unequivocal vascular proliferation/hyperplasia and/or necrosis were classified as “astrocytoma, *IDH-*mutant, WHO grade 4” (*n* = 54, 6.8%), and the remainder were classified as “astrocytoma, *IDH-*mutant, lower grade” (*n* = 221, 27.9%). Because the criteria for the distinction between grades 2 and 3 *IDH*-mutant tumors are not well-defined, we did not assign specific grades for oligodendroglioma or lower-grade astrocytoma. Moreover, the pathology reporting formats varied greatly between cases and were not always quantitative, prohibiting a standardized comparison between cases. *IDH*-mutant cases with unknown 1p/19q status were classified as “glioma, *IDH*-mutant, NOS” (*n* = 2, 0.3%). For recurrent tumors without available annotated molecular information, *IDH* status was inferred from their primary counterparts when available. Lastly, cases with unknown *IDH* status were classified as “glioma, NOS” (*n* = 15, 1.6%).

### DNA Damage Repair Genes

DDR genes were selected based on a previously curated database,^34^ encompassing effectors of the major pathways (base excision repair, BER; nucleotide excision repair, NER; direct damage repair, DR; mismatch repair, MMR; homology-dependent recombination, HDR; non-homologous end joining, NHEJ; Fanconi anemia pathway, FA; and translesion DNA synthesis, TLS) and those that regulate nucleotide pool (NP).

### DNA Methylation

A total of 593 TCGA gliomas had available gene expression and DNA methylation data and were included for analyses. We assessed DNA methylation data in the Harmonized database profiled on the HumanMethylation450 platform (485,577 probes). After retrieving beta-values using TCGAbiolinks, we excluded 189,089 probes with no values and 6,079 probes mapping to sex chromosomes. For the remaining 290,409 probes, we used R package ELMER to identify probes that were associated with the transcription start sites (TSS), in accordance with the UCSC annotation^35^

We derived a list of the nearest 20 genes flanking these probes. Probes within 1,500 base pairs from the TSS of DDR genes listed in Supplementary Table S2 were retrieved for analysis (2,799 probes). We excluded 178,118 probes associated with non-DDR genes and 109,482 probes associated with non-TSS sites of DDR genes (Supplementary Figure S2).

### Gene Expression

We downloaded the gene expression data of the 593 gliomas. An array-array intensity correction and transcript normalization were performed using TCGAbiolinks.^29^ We retrieved gene expression data of 274 DDR genes, after excluding those lacking data (*TREX1* and *SLX1A*) (Supplementary Table S2). A per-gene z-score normalization was performed across samples.

### Tumor Classification by DDR Gene Methylation and Expression

Unsupervised clustering of 593 TCGA gliomas was performed using eigengap heuristic spectral clustering (Spectrum R package). We determined the optimal number of tumor clusters by DDR methylation (DDRm) or gene expression profile (DDRr), revealing two methylation clusters (DDRm1-DDRm2) and four gene expression clusters (DDRr1-DDRr4).

### Mutation Analysis and Corrected Mutation Count

Mutational profiles of the 593 TCGA gliomas were retrieved using TCGAbiolinks^29^ (available for 579 cases). Single-nucleotide variants (SNVs), deletions, and insertions were evaluated. The most commonly mutated genes detected in at least 10 samples were included for analysis. Reported tumor mutation burden was obtained from previously published data.^5^ Corrected mutation burden was calculated using the correction factors based on the consensus purity estimate of TCGA samples using a previously reported formula.^36,37^

### High-dimensional Methylation and Gene Expression Data Analysis

ComplexHeatmap R library was used to visualize gene expression results.^38^ UMAP analysis was performed in R using the Uniform Manifold Approximation and Projection (umap) library.^39^ UMAP was used to qualitatively compare the efficacy between DDR and non-DDR genes for tumor classification. Specifically, equal numbers of genes were selected either from the DDR or the non-DDR gene lists for side-by-side UMAP comparisons. Differentially expressed genes (DEGs) were compared between each DDRr cluster and other DDRr clusters (eg, DDRr4 vs DDRr1/2/3) using the TCGAanalyze_DEA function from the TCGAbiolinks R package, using functions from edgeR.^29^  *P*-values were adjusted using a false discovery rate of 0.01 to return DEGs.

### Antibody Validation and Conjugation

An imaging mass cytometry (IMC) antibody panel containing key DDR effectors and lineage markers was designed (Supplementary Table S3). Antibodies were obtained pre-conjugated or custom conjugated using the MaxPar X8 labeling kit (Standard Biotools, CA, USA). Antibodies were validated using immunofluorescence prior to isotope conjugation using a tissue microarray (TMA) containing 1-mm cores of formalin-fixed paraffin-embedded (FFPE) control tissue/cell lines. Antibody clones that demonstrated the expected expression pattern and cellular localization were selected. The specificity of the isotope-conjugated antibodies was verified on a 5-µm section of the same TMA using the Hyperion IMC System (Standard Biotools, CA, USA).

### Patient Sample Tissue Microarray and Imaging Mass Cytometry

Archived FFPE glioma specimens were obtained under the approval of the Yale Institutional Review Board (protocol 1301011360) in accordance with the Declaration of Helsinki. A TMA containing 1-mm cores of 118 gliomas was constructed, each representing a viable tumor region. A 5-µm section of the TMA was incubated for 2 h at 60 °C, followed by dewaxing in xylene and rehydrated in ethanol gradient. Heat-induced epitope retrieval was performed for 30 min in 1 mM EDTA (pH 8.0) at sub-boiling temperature in a steamer. The tissue was blocked with 3% bovine serum albumin (BSA) in phosphate-buffered saline (PBS) for 1 hour at room temperature, followed by an overnight staining at 4 °C with an antibody cocktail diluted in 1% BSA in PBS (Supplementary Table S3), which included a cocktail for pan-membrane labeling (ICSK1, ICSK2, ICSK3; Standard Biotools, CA, USA). Cell intercalator iridium (Ir) (Standard Biotools, CA, USA) was used for nuclear labeling. Images were acquired by Hyperion Mass Cytometry System. *IDH* mutation and *MGMT* promoter methylation status were obtained from the neuropathology reports. The time to progression or death was followed until July 2021 (Supplementary Table S4).

### Imaging Mass Cytometry Acquisition and Single-cell Analysis

Images were generated and exported using MCD Viewer software (Standard Biotools, CA, USA), and reviewed in ImageJ.^40^ CellProfiler was used to prepare images for segmentation using the nuclear (histone H3, Ir-191, and Ir-193) and pan-membrane signals.^41^ The output data from CellProfiler were uploaded into HistoCAT for single-cell signal quantification,^42^ exported as .csv files, and uploaded to OMIQ for analysis (omiq.ai, accessed March 2023). Signal normalization using the fdaNorm pipeline^43^ was performed within OMIQ. A total of 232,957 glioma cells were computationally isolated by manual gating (Supplementary Figure S11b), defined as histone H3-expressing events that lacked CD45 (leukocyte marker), CD31 (endothelial cell marker), and NeuN (neuron marker) expression. A t-distributed stochastic neighbor embedding (t-SNE) analysis was performed using DDR markers. Phenograph was performed within OMIQ to reveal glioma cell subsets based on their DDR signatures.^44^ Markers used for tSNE and Phenograph analyses are listed in Supplementary Table S3.

### Software and Data Analysis Resources

TCGA data analysis was performed in R (version 4.2.2) and R Studio (version 2022.07.2 + 576). Figures were generated in R Studio and Illustrator (version 27.0.1). R packages and software are listed in Supplementary Table S5.

## Results

### Re-classification of TCGA Gliomas in Accordance with the WHO Guidelines

We accessed 792 cases from the TCGA-GBM and TCGA-LGG datasets with either methylation or gene expression data. Two neuropathologists reassigned the diagnoses following the histomorphology and molecular criteria per the updated WHO classification (WHO2021; Supplementary Figure S1a-b and Supplementary Table S1).^32^ Overall, 53.3% (282 cases) of TCGA-LGG and 11.4% (30 cases) of TCGA-GBM were reclassified (Supplementary Figure S1c).

Among TCGA-LGG, 7.0% (37 cases) were reassigned as astrocytoma, *IDH*-mutant grade 4, and 13.6% (72 cases) as GBM (Supplementary Figure S1d). Of the previously assigned astrocytoma, 2.5% (5 cases) demonstrated 1p/19q codeletion and were reclassified as oligodendroglioma. Many previously assigned oligodendroglioma lacked 1p/19q codeletion and were reclassified as astrocytoma (41 cases, 21.1%). Oligoastrocytoma is no longer a recognized entity in WHO2021^32^; those with *IDH* mutation and known 1p/19q codeletion status were reclassified as either astrocytoma (lacking 1p/19q codeletion; 60%, 81 cases) or oligodendroglioma (1p/19q codeletion; 37 cases, 27.4%).

Most TCGA-GBM (233 cases, 88.6%) were given the same classification as GBM. Nineteen previously assigned GBM demonstrated *IDH* mutation, and 17 of these met the morphologic/molecular criteria for astrocytoma, *IDH*-mutant grade 4. The *IDH* status of 11 previously defined GBM cases was unknown, and these were classified as glioma, NOS.^33^

### Correlation Between DDR Gene Methylation and Molecular Diagnosis

**Figure 1.**
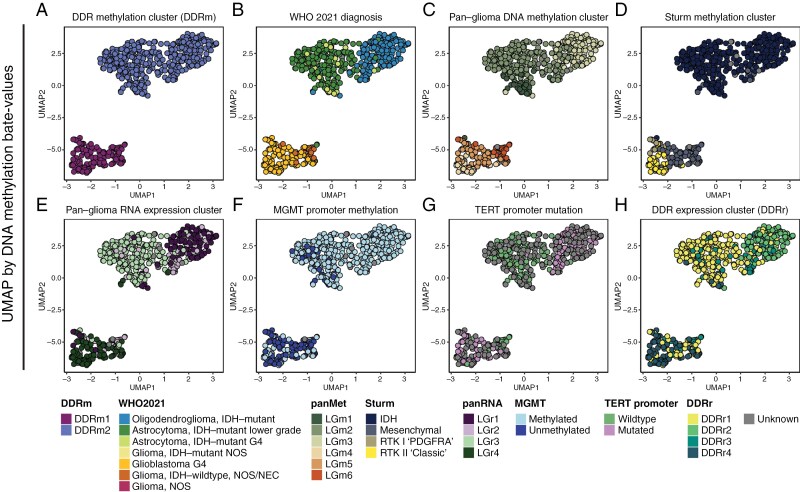
**DDR gene promoter methylation status correlates with molecular-defined diagnosis and previously reported molecular classes.** (a) UMAP analysis of TCGA glioma cases by the methylation beta-values at the DDR gene transcription start sites shows two methylation clusters (DDRm) identified by unsupervised spectral clustering, and (b) the corresponding reclassified molecular-defined tumor diagnoses in accordance with the WHO classification. Previously reported molecular classes of the TCGA gliomas included in this study are depicted in (c), (d), and (e). (f) *MGMT* promoter methylation and (g) *TERT* promoter mutation status are shown. (h) The corresponding gene expression clusters (DDRr) clusters identified by a separate unsupervised spectral clustering are depicted (see also Figures 2 and S3).

We subsequently characterized 593 TCGA cases with both DNA methylation and gene expression data (Figure 1 and Supplementary Table S2). We focused on methylation signatures at the promoter regions associated with DDR genes (2,799 probes, Supplementary Figure S2; see Materials and methods). *SMC6* and *FANCD2* were associated with a single probe each, while other DDR genes were associated with multiple probes (2-43 probes per gene). Unsupervised spectral clustering revealed two methylation groups, DDRm1 and DDRm2 (Figure 1a and Supplementary Figure S3a). DDRm clusters correlated with molecular-defined diagnoses (Figure 1b). DDRm1 showed a higher tumor mutation count (Supplementary Figure S3b-c) and a significantly lower DDR methylation beta-values (Supplementary Figure S3d). DDRm1 was predominantly GBM (127/157 cases, 80.9%). Most *IDH*-mutant tumors were classified as DDRm2 (oligodendroglioma 172/172 cases, 100%; astrocytoma 260/263 cases, 98.9%). DDRm1 and DDRm2 correlated with previous epigenetic-based (panMet LGm1-6 and Sturm; Figure 1c-d) and RNA-based (panRNA LGr1-4; Figure 1e) classifications.^5^ DDRm1 corresponded to hypomethylated LGm4/5/6 and non-*IDH* mutant epigenetic classes (Mesenchymal, RTK1 “PDGFRA,” and RTKII “Classic”) (136/157 cases, 86.6%),^5,45^ whereas DDRm2 was enriched for hypermethylated LGm1-3 (421/436 cases, 96.6%) and epigenetic “IDH” group (404/436 cases, 92.6%). Tumors with unmethylated *MGMT* promoter were classified more frequently as DDRm1 (87/116 cases, 75.0%) compared to DDRr2 (29/116 cases, 25.0%) (Figure 1f). Tumors with *TERT* promoter mutation were predominantly DDRm1 GBM (46/139 cases, 33.1%) or DDRm2 oligodendrogliomas (86/139 cases, 61.9%) (Figure 1g), as expected for these tumor types. Overall, DDRm classes were reflective of the global methylation phenotype and *IDH* status, indicating that DDR methylation profiles are sufficient determinants for molecular classification.

### Transcriptional Profiling of DDR Genes Correlated with Molecular and Methylation Subtypes

**Figure 2.**
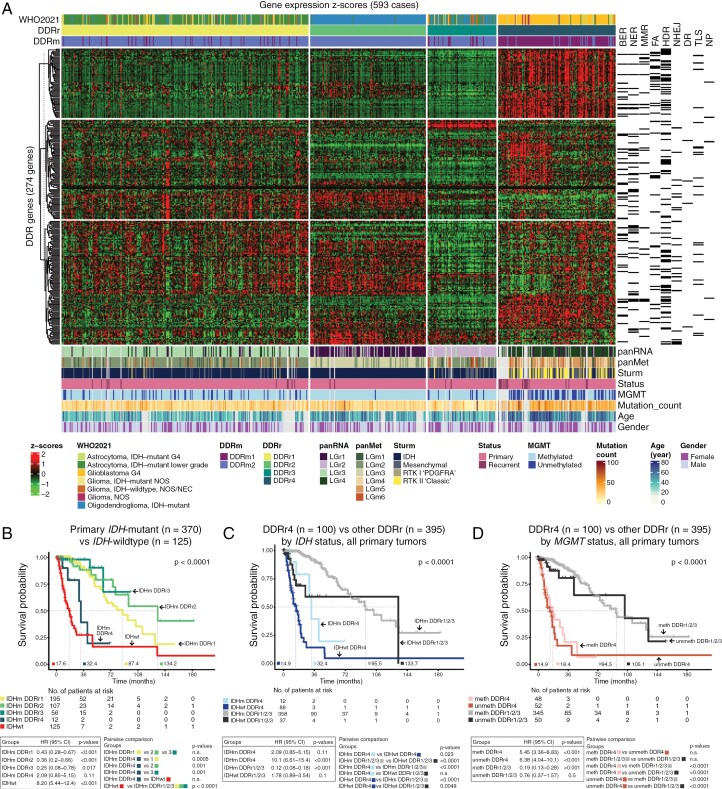
**Transcriptional profiling of DDR genes reveals tumor groups that are predictive of clinical outcomes.** (a) Spectral clustering by DDR gene expression z-scores reveals 4 tumor clusters (DDRr) and their correlation with the revised WHO 2021 diagnoses are shown (top). The corresponding DDR pathways of individual DDR genes are depicted (right). The correlation between DDRr clusters, DDRm, previously reported molecular classes, *MGMT* promoter methylation, *TERT* promoter mutation, tumor mutation burden, and patient demographics are shown (bottom). (b) Kaplan-Meier (KM) survival analysis compares the overall survival probability between primary *IDH*-mutant tumors in different DDRr clusters and *IDH*-wildtype tumors. (c) Survival analysis based on DDRr (DDRr4 vs others) and *IDH* mutation status is depicted. (d) Survival comparison of tumors from different DDRr clusters based on their *MGMT* promoter methylation status is also shown. P-values of the KM plots were derived from log-ranked tests. Hazard ratios (HR) with 95% confidence intervals (CI) and p-values from different tumor groups are summarized below the KM plots. BER = base excision repair, NER = nucleotide excision repair, MMR = mismatch repair, FA = Fanconi anemia, HDR = homology-directed repair, NHEJ = non-homologous end joining, DR = direct repair, TLS = trans-lesion synthesis, NP = nucleotide pool.

Spectral clustering of z-score-transformed data of 274 DDR genes demonstrated intertumoral diversity, and revealed four tumor clusters (DDRr1-4) with distinct DDR expression patterns that correlated with WHO2021 diagnoses (Figure 2a, Supplementary Figure S3e). DDRr4 was largely GBM (102/127, 80.3%). All 125 DDRr2 tumors were oligodendrogliomas. DDRr1 was enriched for lower-grade *IDH*-mutant astrocytoma (186/267, 69.7%), while DDRr3 contained both astrocytoma (30/74, 40.5%) and oligodendroglioma (32/74, 43.2%). The top mutated genes were reflective of the common types of gliomas within each DDRr cluster (Supplementary Figure S5). Besides *IDH1*, *TP53*, and *ATRX*, which are defining and/or characteristic mutations of glioma types, DDR gene mutations were rare. *CIC* alterations were seen in 72% (*n* = 77) of DDRr2 tumors, a common co-occurring mutation in oligodendrogliomas (Supplementary Figure S5b).^46^ Notably, only 12.6% of DDRr4 cases had *IDH1* mutation (Figure S5d). While DDR gene expression in DDRr4 was largely elevated (Figure 2a), the mutational burden in this group was significantly higher than others (*P* < .0001, Supplementary Figure S3f-g).

To determine whether the DDR genes were sufficient and critical for the classification of gliomas, UMAP analyses using either all or DDR-only gene expressions were compared (Supplementary Figure S6a). Both analyses demonstrated similar segregations of tumors based on their WHO2021 diagnoses, indicating that DDR gene expression profiles were sufficient for glioma classification. When either DDR (Supplementary Figure S6b) or non-DDR genes (Supplementary Figure S6c) were randomly selected, UMAP qualitatively demonstrated that DDR gene expression profiles were superior in separating gliomas by molecular diagnoses, supporting the critical roles of DDR genes in molecular-based tumor classification.

Glioma classification by DDR expression aligned with molecular and methylation subtypes (Supplementary Figure S4). Collective analysis of DDR methylation and gene expression demonstrated a correlation between DDRm and DDRr clusters (Figures 1h and 2a, Supplementary Figure S4c). DDRr4 had an overall DDR hypomethylation signature (Supplementary Figure S3h). DDRm1 showed an enrichment for DDRr4 tumors, which were predominantly GBM (Figure 1a, 1b, 1h). DDRr4 demonstrated high DDR gene expression across multiple pathways, a key feature that distinguished DDRr4 from other clusters (Supplementary Table S3 and Figure 2a). In contrast, DDRm2 composed predominantly of DDRr1-3 with lower DDR expression (Figures 1a, 1h, 2a). Specifically, DDRr3 demonstrated markedly reduced DDR gene expression across pathways, except for a few DDR genes, including *PPP4R4* and *YWHAG* (see below). DDRr clusters correlated with previously reported RNA-sequencing pan-glioma expression subtypes (panRNA, LGr1-4; Figure 2a and Supplementary Figure S4d).^5^  *IDH-*wildtype rich LGr4 were predominantly classified as DDRr4, most being GBM with high DDR expression. In contrast, LGr1/LGr2/LGr3 groups, enriched for *IDH*-mutant tumors, were largely classified as DDRr2, DDRr3, and DDRr1, respectively. These findings indicate that the DDR landscape is a critical driver of glioma classification by transcriptional profiling and corresponds to molecular-defined diagnoses.

### DDRr4 Carried Poor Outcome Independent of IDH Mutation or MGMT Promoter Methylation Status

We hypothesized that DDR profiles predict therapy response and survival. DDRr4 showed a significantly worse overall survival (OS) compared to other groups (Supplementary Figure S7, log-rank *P* < .0001, HR = 9.19, 95% CI 6.14-13.8). Since most DDRr4 tumors were GBM that inherently carry worse outcomes, we further evaluated the OS among *IDH*-mutant tumors from different DDRr clusters compared to *IDH*-wildtype cases (Figure 2b). DDRr4 *IDH*-mutant gliomas had a significantly worse OS compared to DDRr1/2/3 *IDH*-mutant gliomas (IDHm DDRr4 vs IDHm DDRr1 *P* = .0005, IDHm DDRr4 vs IDHm DDRr2/3 *P* = .001). The OS of DDRr4 *IDH*-mutant and *IDH*-wildtype gliomas were not significantly different (DDRr4 IDHm vs IDHwt *P* = .18), indicating that *IDH*-mutant gliomas with a DDRr4 profile were as clinically aggressive as their *IDH*-wildtype counterparts.

While DDRr and DDRm largely correlated, with *IDH*-mutant-rich DDRr1-3 predominantly clustering with the hypermethylated DDRm2, many DDRr1 and DDRr3 tumors showed relative DDR hypomethylation and were classified as DDRm1 (Figure 1h, 2a, Supplementary Figure S4c). The proportion of tumors in each DDRr cluster classified as DDRm1 was as follows: DDRr1 12.4% (33/267), DDRr2 0% (0/125), DDRr3 18.9% (14/74), and DDRr4 48.5% (110/227). Survival analysis based on the combined DDRr/DDRm profiles revealed that within the same DDRr, those with a hypomethylated phenotype (DDRm1) portended an even worse OS (Log-rank *P* < .0001; Supplementary Figure S8). For example, the median OS of DDRr4/DDRm1 was 14.9 months compared to 32.4 months of DDRr4/DDRm2 (*P* = .028).

The OS advantage of *IDH* mutation and *MGMT* promoter methylation in the TCGA, prior to additional DDR classification, was similar to previously reported data (Supplementary Figure S9). Even among *IDH*-wildtype gliomas that inherently carry poor survival (Supplementary Figure S9a), DDR gene expression profiles further revealed that DDRr4 *IDH*-wildtype carried a significantly worse OS than other *IDH*-wildtype tumors (Figure 2C, *P* = .0049). The adverse outcome of DDRr4 was also apparent among *IDH*-mutant gliomas compared to *IDH*-mutant tumors that were classified as DDRr1/2/3 (*P* < .0001). *IDH* mutation still portrayed some survival advantages within the DDRr4 group (*P* = .023). When tumors were further grouped based on their WHO2021 diagnoses, DDRr4 showed worse OS in both astrocytoma (Supplementary Figure S10a, *P* = .013, HR 4.71, 95% CI 1.62-13.8) and oligodendroglioma (Supplementary Figure S10b, *P* = .029, HR 11.9, 95% CI 1.4-102). Trends for worse OS of DDRr4 were also seen in lower grade astrocytoma, grade 4 astrocytoma, and GBM (Supplementary Figure S10c-e).

We next evaluated whether DDR gene expression profiling, specifically DDRr4, adds prognostic value beyond *MGMT* promoter methylation status (Figure 2d and Supplementary Figure S9b). Tumors were divided into four groups: (1) DDRr4 *MGMT* promoter methylated, (2) DDRr4 *MGMT* promoter unmethylated, (3) DDRr1/2/3 *MGMT* promoter methylated, and (4) DDRr1/2/3 *MGMT* promoter unmethylated. Critically, DDRr classification was the main predictor of survival, with DDRr4 and DDRr1/2/3 tumors having worse and favorable OS, respectively, regardless of *MGMT* promoter methylation status (*P* < .0001).

### DDR Transcription Across Pathways Is Enriched in High-Grade Tumors

**Figure 3.**
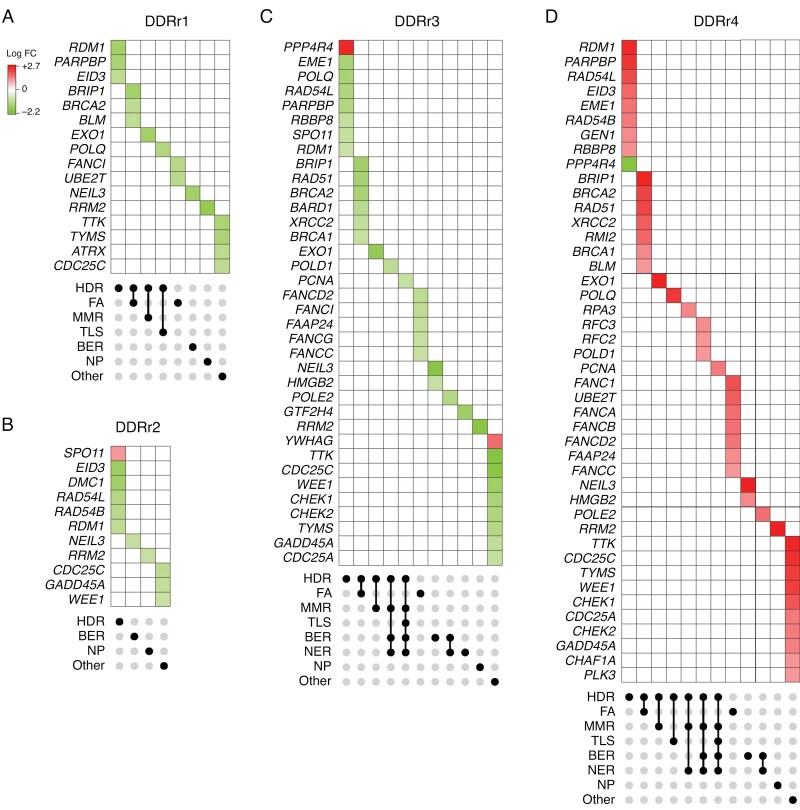
**Differential enrichment of DDR transcription gene expression across pathways in DDRr clusters.** Upset plots demonstrating differentially expressed DDR genes that were significantly enriched in (a) DDRr1, (b) DDRr2, (c) DDRr3, or (d) DDRr4 tumors. The heat represents the log fold change of the gene expression of a given gene within that DDRr cluster compared to that gene in other DDRr clusters. Gene-associated DDR pathways are shown as black circles at the bottom of each plot. BER = base excision repair, NER = nucleotide excision repair, MMR = mismatch repair, FA = Fanconi anemia, HDR = homology-directed repair, TLS = trans-lesion synthesis, NP = nucleotide pool.

Transcriptional profiling demonstrated a relatively increased DDR gene expression in DDRr4 (Figure 2a). We investigated whether the enrichment was pathway-specific, as this may suggest glioma type-specific oncogenesis and uncover novel biomarkers. Analysis of differentially expressed genes was performed to identify DDR genes that drove the distinction between DDRr clusters (Figure 3). Upregulation of DDR gene expression across several DDR pathways was observed in DDRr4, including homology-dependent recombination (HDR), Fanconi anemia pathway (FA), mismatch repair (MMR), translesion DNA synthesis (TLS), base excision repair (BER), and nucleotide excision repair (NER) (Figure 3d). HDR was a core pathway with differential regulation across DDRr clusters. Except *PPP4R4* (further discussed below), HDR-related genes were upregulated in DDRr4 but were downregulated in other DDRr clusters. Many upregulated HDR genes were also critical players in other overlapping pathways, including FA (eg, *BRIP1*, *BRCA2*), MMR (eg, *EXO1*), and BER (eg, *POLD1*, *PCNA*). Notably, upregulation of several FA-specific genes, including *FANCI*, *UBE2T*, and *FANCC*, was a unique feature of DDRr4, and many of these genes were downregulated in other DDRr clusters (particularly DDRr1 and DDRr3) that were associated with better outcomes (Supplementary Figure S7).

RNA expressions of *YWHAG* and *PPP4R4* were increased in DDRr3 compared to other groups (Figure 3c). *YWHAG* encodes 14-3-3γ protein, which showed increased expression in various cancers and likely mediates glioma progression.^47^  *PPP4R4* expression was upregulated in DDRr3 and downregulated in DDRr4 (Figure 3c-d). *PPP4R4* encodes protein phosphatase 4 regulatory subunit 4, a subunit of serine/threonine-protein phosphatase 4 (PP4), which aids the repair of double-strand breaks by HDR.^48,49^ Thus, downregulation of *PPP4R4* in DDRr4 is in keeping with the overall DDR upregulation in this group. High-level PP4 expression promotes glioma proliferation and is prognostically adverse.^50^

Upregulation of *SPO11* was observed in DDRr2 but showed reduced expression in DDRr3 (Figure 3b-c). SPO11 induces double-strand breaks in meiosis and shows aberrant expression in many cancer types, including melanoma, lung cancer,^51^ and colorectal cancer.^52^ Since DDRr2 comprised solely of oligodendrogliomas (Figure 2a), this finding may indicate lineage-specific DDR regulatory mechanisms. Beyond these genes, DDRr1, DDRr2, and DDRr3 tumors showed down regulation of transcriptional expression of DDR genes across several pathways.

Notably, genes related to non-homologous end-joining (NHEJ) and direct repair pathways, such as DNA-PK and 53BP1, were not differentially expressed across clusters, indicating limited utility as glioma biomarkers.^53–56^ Moreover, while effectors in the DNA reversal repair pathway have been implicated in the prediction of glioma responsiveness to standard treatment with alkylating agents,^6,57^ genes encoding MGMT and alkylated DNA repair protein B (AlkB) homologs did not show differential expression or methylation signatures that aid the distinction between glioma types.

### Single-cell Heterogeneity of DDR Protein Expression

**Figure 4.**
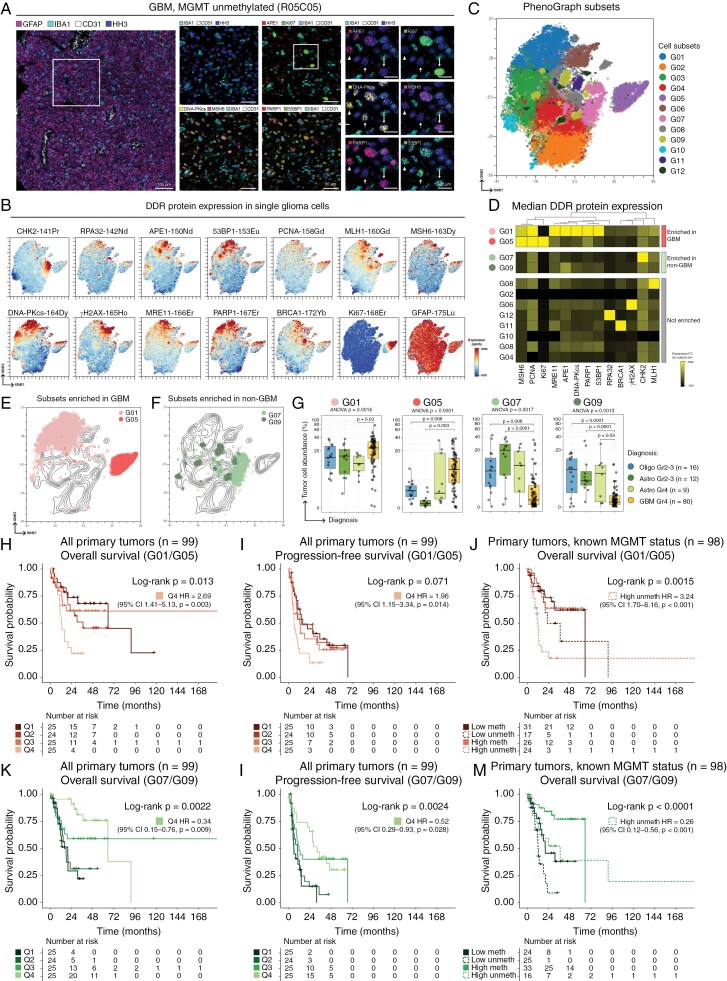
**The abundance of DDR-proficient tumor cells differs across tumors and correlates with clinical outcomes.** (a) Imaging mass cytometry images from a representative glioblastoma, *MGMT* promoter unmethylated, depicting diversity of DDR in single cells. (Left image) A low-magnification view showing glial/tumor cells (GFAP+, magenta), microglia (IBA1+, cyan), and endothelial cells (CD31+, white). Nuclear labeling with total histone H3 (HH3) is shown in blue. (Middle panels) Higher magnification images illustrate diversity of APE1, DNA-PKcs, MSH6, PARP1, and 53BP1 protein expression in single cells. (Right panels) Long arrows highlight a proliferative (Ki67+) glioma cell lacking APE1 with expression of other depicted DDR effectors. The middle arrowheads highlight a non-proliferative (Ki67-) glioma cell expressing only DNA-PKcs and 53BP1. The left arrowheads show a non-proliferative glioma cell expressing all DDR effectors shown here. (b) t-SNE analysis of glioma cells (see Supplementary Figure S11b for gating strategy), generated using only DDR markers (see Supplementary Table S3). Each map shows the expression of the designated DDR proteins, Ki67, or GFAP (scale = asinh transformation). (c) Identification of 12 DDR-defined glioma cell subsets using Phenograph. (d) Heatmap showing median expression of DDR protein effectors (columns, fold change by column’s minimum) in 12 DDR-defined glioma cell subsets (rows). Cell subsets enriched in glioblastoma (GBM; G01 and G05, red) and in non-GBM (G07 and G09, green) are shown. Cell density contour t-SNE maps of all glioma cells in the cohort, overlaid by (e) cell subsets enriched in GBM (G01 and G05) or (f) cell subsets enriched in non-GBM (G07 and G09). (g) Box and whisker plots comparing the abundance of G01, G05, G07, and G09 cell subsets in oligodendroglioma (blue), lower grade astrocytoma (dark green) astrocytoma WHO grade 4 (light green), or GBM (yellow). Y-axes represent tumor cell subset abundance (% per total tumor cells, scale = log_10_). The lower and upper hinges correspond to the first and third quartiles (the 25th and 75th percentiles), and the horizontal lines represent median values. Whiskers depict the 1.5x first and third quartiles. Gray circles represent individual tumors. Statistical analysis was performed using one-way ANOVA followed by pairwise t-tests. Kaplan-Meier plots illustrate (h and k) the overall survival and (i and l) the progression-free survival probabilities of cases based on the abundance of G01/G05 or G07/G09 cell subsets. Primary tumors (n = 99) were divided into quartiles based on cell subset abundance (Q1 = lowest, Q4 = highest). Analysis of the overall survival probabilities of primary tumors based on their reported *MGMT* promoter methylation status (n = 98) was performed. The cases were divided into two groups (high, top 50%; low, bottom 50%) based on the abundance of (j) G01/G05 or (m) G07/G09 cells). CI = confidence interval. P-values of the KM plots were derived from log-ranked tests.

We used imaging mass cytometry (IMC) on a tissue microarray (TMA) of 118 diffuse gliomas, applying a 26-marker panel containing DDR, lineage, and cellular compartment markers (Supplementary Table S3; Supplementary Figure S11a). Intratumoral heterogeneity of DDR protein expression was observed between single glioma tumor cells (Figure 4 and Supplementary Figure S11b, glioma tumor cells = CD45^neg^ CD31^neg^ NeuN^neg^). Similar to the DDRr signatures identified by RNA expression of the TCGA dataset (Figure 2a), tumor cells with expression of multiple DDR effector proteins were predominantly seen in GBM (Figure 4a). Qualitatively, tumor cells with different proliferative states in individual tumors showed diverse expressions of DDR effectors. For example, in GBM R05C05, Ki67+ proliferative tumor cells expressed effectors across DDR pathways, such as APE1, MSH6, PARP1, and 53BP1. In addition, several DDR effectors were also expressed in Ki67- non-proliferative glioma cells, albeit at a lower level. In contrast, cells lacking DDR protein expression were seen more frequently in *IDH*-mutant tumors, which, as expected, contained fewer Ki67+ proliferative cells (representative astrocytoma and oligodendroglioma in Supplementary Figure S12a and S12c, respectively).

We explored various parameters, including the abundance of glioma tumor cells that were defined by distinct DDR characteristics. A t-distributed stochastic neighbor embedding (t-SNE) analysis was performed on 232,957 single glioma cells across 118 tumors (ranging between 395 to 3,994 cells per tumor). This illustrated the diverse single-cell DDR phenotypes (Figure 4b and Supplementary Figure S12e).^58^ Overall, single-cell DDR landscape showed marked differences between *IDH*-wildtype and *IDH*-mutant gliomas, while only slight differences between *IDH*-mutant astrocytomas and oligodendrogliomas were observed (Supplementary Figure S12e-f). In addition, tumors of the same molecular-defined types also demonstrated intertumoral DDR heterogeneity (Supplementary Figure S12g). Glioma cells with high DDR protein expression frequently expressed DDR proteins across several pathways (Figure 4b, top of map, and Supplementary Figure S12f, short arrow). A distinct population of Ki67+ cells was identified and showed high expression of several DDR effectors (Figure 4b, right of map, and Supplementary Figure S12f, long arrow). Overall, the findings illustrated DDR protein expression heterogeneity at the single-cell level, and presence of tumor cells that harbor distinct DDR signatures.

PhenoGraph clustering identified 12 glioma subsets based on DDR protein expression (Figure 4c-d).^44^ Several subsets were enriched for tumor cells expressing DDR effectors across pathways, notably subsets G01 and G05, both of which showed high-level MSH6 and PCNA expression (Figure 4d). Notably, *PCNA* transcript level was also upregulated in TCGA glioma DDRr4 cluster (Figures 2a and 3d). In contrast, several glioma cell subsets show an overall low-level DDR expression (G02, G03, G04, G09, and G10) while others showed DDR effector-specific enrichment (G06 γH2AX+, G07 CHK2+, G08 MLH1+, G11 BRCA1+, G12 RPA32+). The abundance of cell subsets with distinct DDR phenotypes differed across types of diffuse gliomas (Supplementary Figure S12f-g). Tumor cells with high-level DDR expression were significantly enriched in the highly aggressive gliomas (Figure 4e and 4g). Specifically, G01 cells with high-level expression of DDR effectors across pathways were enriched in GBM, and showed lower abundance in *IDH*-mutant tumors, including grade 4 *IDH*-mutant astrocytomas. In contrast, the G05 subset, which is composed of Ki67+ tumor cells with proliferative activity, was reduced in lower-grade (grade 2-3) *IDH*-mutant gliomas. G05 cells showed slightly higher abundance in GBM compared to grade 4 *IDH*-mutant astrocytomas, although statistical significance was not reached.

Glioma cells with high CHK2 expression (G07) and those with low DDR protein expression (G09) were significantly enriched in *IDH*-mutant tumors compared to GBM (Figure 4f-g). Identification of enriched CHK2 protein expression in *IDH*-mutant gliomas by IMC, particularly in astrocytomas, contrasted with the upregulation of *CHEK2* transcript expression in DDRr4 gliomas, which were enriched for *IDH*-wildtype tumors (Figure 3d). Interestingly, cytoplasmic localization of CHK2 was observed in several *IDH*-mutant gliomas, which predominantly lacked phosphorylation at Threonine residue 68 (representative astrocytoma and oligodendroglioma shown in Supplementary Figures S12b and S12d, respectively). The mechanism underlying this observation remains to be elucidated but may indicate an inactive state of CHK2 given the lack of activating phosphorylation.

### Enrichment of Cells with DDR Expression Across Multiple Pathways Linked to Poor Outcome

We explored the effect of DDR-defined cell subset abundance toward clinical outcomes. We analyzed pre-therapy gliomas (*n* = 99), which were grouped based on the combined abundance of G01/G05 cells (subsets enriched in GBM, Figure 4h-j) or G07/G09 cells (subsets enriched in non-GBM, Figure 4k-m). Cell abundance was divided into quartiles (Q1 and Q4 being the bottom and the top quartiles based on the abundance of cells of interest, respectively). Tumors with the highest number of G01/G05 cells were associated with the worst OS (Figure 4h; Q4 *P* = .003, HR 2.69, 95% CI 1.41-5.13) and showed a trend for unfavorable PFS with those harboring the highest number of G01/G05 cells (Q4) being associated with significantly shorter time to progression (Figure 4i; Q4 *P* = .014, HR 1.96, 95% CI 1.15-3.34). In contrast, tumors with the highest abundance of G07/G09 cells were associated with significantly improved overall and progression-free survivals (Figure 4k-l).

To assess if the DDR-defined cell subsets provided additional prognostic implication beyond *MGMT* promoter methylation, we performed additional survival analyses on primary glioma with known *MGMT* status in the TMA cohort (*n* = 98). Tumors with low (Q1 + Q2) and high (Q3 + Q4) combined abundance of G01/G05 cells or G07/G09 cells were grouped based on their *MGMT* status. Our results showed that an increased abundance of G01/G05 cells (Figure 4j) and decreased abundance of G07/G09 cells (Figure 4m) are associated with worse overall survival even among *MGMT* unmethylated primary diffuse gliomas.

## Discussion

TCGA datasets were interrogated to examine how DDR gene methylation and expression states influence the classification and prognosis of adult-type diffuse gliomas. While global DNA methylation effectively classifies CNS tumors,^13,14^ we found that DDR gene epigenetic states alone could distinguish molecularly and prognostically diverse gliomas in line with WHO classification (Figure 1).^13^ Promoter methylation of DDR genes effectively separated gliomas into groups corresponding to known epigenetic and transcriptional subclasses.^5^

DDR gene expression stratified gliomas into prognostically distinct categories (Figure 2), with enrichment of DDR gene expression across multiple signaling pathways (Figure 3, DDRr4) associated with aggressive behavior independent of known biomarkers. Notably, *IDH*-mutant DDRr4 tumors showed a significantly worse OS compared to non-DDRr4 *IDH*-mutant tumors, independent of the tumors’ lineage-defined molecular diagnoses (Supplementary Figure S7). Importantly, *IDH*-mutant DDRr4 tumors behaved as poorly as their DDRr4 *IDH*-wildtype counterparts, with DDRr4 representing the most aggressive group of tumors. High DDR transcript expression at diagnosis further predicted adverse outcomes independent of *MGMT* status.

While bulk-tumor DDR profiles effectively categorized gliomas into prognostically distinct groups, intratumoral single-cell diversity is well known and has potential implications for treatment responsiveness.^9,19,20,22,24,52^ We showed that single-cell quantitation of DDR proteins revealed cell subsets with distinct signatures (Figure 4). The spectrum of DDR expression in individual tumor cells recapitulated the bulk-tumor level DDR profiles. Specifically, the abundance of cell subsets with distinct DDR characteristics was associated with outcomes, suggesting that the number of tumor cells expressing DDR effectors at initial diagnosis likely predicts chemosensitivity.

Overall, bulk-tumor and single-cell data suggest treatment resistance mechanisms may not depend on a specific pathway, but rather an interplay between multiple interconnected and cooperative DDR machinery to ensure efficient repair of genomic lesions induced by such treatment. A critical discovery is that pan-DDR profiling at baseline provides prognostic value beyond standard biomarkers, *MGMT* and *IDH* status. Functional investigation of the link between DDR upregulation and survival could yield important biological insights. One possibility lies in the recognized connection between stem-like state and upregulated DDR, potentially due to replication stress, which may induce therapy resistance.^59,60^ DDR upregulation could thus be a mechanistic response to the increased genomic stress. Alternatively, global hypomethylation may drive DDR upregulation (Figure 1a, 1h, Supplementary Figure S3d; see DDRr4 and DDRm1).

Studies to characterize responses to various treatments or combinations after DDR classification may inform personalized therapeutic strategies. Incorporating pan-DDR stratification in clinical trials to interrogate responses to various therapies could provide further insight into their prognostic and predictive values. This stratification may uncover novel insights into how pan-DDR characteristics in gliomas predict response to distinct therapeutic classes, such as alkylating agents, DNA cross-linkers, and DDR-targeted therapies.

The correlation between the abundance of high DDR expressing cells and poor outcomes suggests that these cells are inherently more chemoresistant, emphasizing the need to explore alternative interventions to overcome resistant subclones early in treatment. Future studies leveraging patient-derived and engineered glioma models, stratified by pan-DDR subtypes, could reveal treatment sensitivity, preferential responses to DDR-targeted therapies, and novel biomarkers of response.

## Supplementary Material

noaf199_Supplementary_Data

## Data Availability

TCGA data were obtained from the original published source, using the R library TCGAbiolinks. Glioma TMA data will be made available upon reasonable request.

## References

[1] Ostrom QT, Cioffi G, Waite K, Kruchko C, Barnholtz-Sloan JS. CBTRUS Statistical Report: Primary Brain and Other Central Nervous System Tumors Diagnosed in the United States in 2014-2018. Neuro Oncol. 2021;23(12 Suppl 2 12 Suppl 2):iii1–iii105. 34608945 10.1093/neuonc/noab200 PMC8491279

[2] Stupp R, Mason WP, van den Bent MJ, et al; European Organisation for Research and Treatment of Cancer Brain Tumor and Radiotherapy Groups. Radiotherapy plus concomitant and adjuvant temozolomide for glioblastoma. N Engl J Med. 2005;352(10):987–996. 15758009 10.1056/NEJMoa043330

[3] Singh N, Miner A, Hennis L, Mittal S. Mechanisms of temozolomide resistance in glioblastoma - a comprehensive review. Cancer Drug Resist. 2021;4(1):17–43. 34337348 10.20517/cdr.2020.79 PMC8319838

[4] Kessler T, Sahm F, Sadik A, et al Molecular differences in IDH wildtype glioblastoma according to MGMT promoter methylation. Neuro Oncol. 2018;20(3):367–379. 29016808 10.1093/neuonc/nox160 PMC5817966

[5] Ceccarelli M, Barthel FP, Malta TM, et al; TCGA Research Network. Molecular Profiling Reveals Biologically Discrete Subsets and Pathways of Progression in Diffuse Glioma. Cell. 2016;164(3):550–563. 26824661 10.1016/j.cell.2015.12.028 PMC4754110

[6] Hegi ME, Diserens AC, Gorlia T, et al MGMT gene silencing and benefit from temozolomide in glioblastoma. N Engl J Med. 2005;352(10):997–1003. 15758010 10.1056/NEJMoa043331

[7] Ganesa S, Sule A, Sundaram RK, Bindra RS. Mismatch repair proteins play a role in ATR activation upon temozolomide treatment in MGMT-methylated glioblastoma. Sci Rep. 2022;12(1):5827. 35388070 10.1038/s41598-022-09614-x PMC8987098

[8] Touat M, Li YY, Boynton AN, et al Mechanisms and therapeutic implications of hypermutation in gliomas. Nature. 2020;580(7804):517–523. 32322066 10.1038/s41586-020-2209-9 PMC8235024

[9] McCord M, Steffens A, Javier R, et al The efficacy of DNA mismatch repair enzyme immunohistochemistry as a screening test for hypermutated gliomas. Acta Neuropathol Commun. 2020;8(1):15. 32051040 10.1186/s40478-020-0892-2 PMC7017562

[10] Leelatian N, Hong CS, Bindra RS. The Role of Mismatch Repair in Glioblastoma Multiforme Treatment Response and Resistance. Neurosurg Clin N Am. 2021;32(2):171–180. 33781500 10.1016/j.nec.2020.12.009

[11] Yip S, Miao J, Cahill DP, et al MSH6 mutations arise in glioblastomas during temozolomide therapy and mediate temozolomide resistance. Clin Cancer Res. 2009;15(14):4622–4629. 19584161 10.1158/1078-0432.CCR-08-3012 PMC2737355

[12] Fernandez AF, Assenov Y, Martin-Subero JI, et al A DNA methylation fingerprint of 1628 human samples. Genome Res. 2012;22(2):407–419. 21613409 10.1101/gr.119867.110 PMC3266047

[13] Louis DN, Perry A, Wesseling P, et al The 2021 WHO Classification of Tumors of the Central Nervous System: a summary. Neuro Oncol. 2021;23(8):1231–1251. 34185076 10.1093/neuonc/noab106 PMC8328013

[14] Capper D, Jones DTW, Sill M, et al DNA methylation-based classification of central nervous system tumours. Nature. 2018;555(7697):469–474. 29539639 10.1038/nature26000 PMC6093218

[15] Koelsche C, Schrimpf D, Stichel D, et al Sarcoma classification by DNA methylation profiling. Nat Commun. 2021;12(1):498. 33479225 10.1038/s41467-020-20603-4 PMC7819999

[16] Helleday T, Petermann E, Lundin C, Hodgson B, Sharma RA. DNA repair pathways as targets for cancer therapy. Nat Rev Cancer. 2008;8(3):193–204. 18256616 10.1038/nrc2342

[17] Watanabe R, Nakasu Y, Tashiro H, et al O6-methylguanine DNA methyltransferase expression in tumor cells predicts outcome of radiotherapy plus concomitant and adjuvant temozolomide therapy in patients with primary glioblastoma. Brain Tumor Pathol. 2011;28(2):127–135. 21331613 10.1007/s10014-011-0022-8

[18] Klughammer J, Kiesel B, Roetzer T, et al The DNA methylation landscape of glioblastoma disease progression shows extensive heterogeneity in time and space. Nat Med. 2018;24(10):1611–1624. 30150718 10.1038/s41591-018-0156-x PMC6181207

[19] Snuderl M, Fazlollahi L, Le LP, et al Mosaic amplification of multiple receptor tyrosine kinase genes in glioblastoma. Cancer Cell. 2011;20(6):810–817. 22137795 10.1016/j.ccr.2011.11.005

[20] Patel AP, Tirosh I, Trombetta JJ, et al Single-cell RNA-seq highlights intratumoral heterogeneity in primary glioblastoma. Science. 2014;344(6190):1396–1401. 24925914 10.1126/science.1254257 PMC4123637

[21] Wang L, Jung J, Babikir H, et al A single-cell atlas of glioblastoma evolution under therapy reveals cell-intrinsic and cell-extrinsic therapeutic targets. Nat Cancer. 2022;3(12):1534–1552. 36539501 10.1038/s43018-022-00475-x PMC9767870

[22] Couturier CP, Ayyadhury S, Le PU, et al Single-cell RNA-seq reveals that glioblastoma recapitulates a normal neurodevelopmental hierarchy. Nat Commun. 2020;11(1):3406. 32641768 10.1038/s41467-020-17186-5 PMC7343844

[23] Johnson KC, Anderson KJ, Courtois ET, et al Single-cell multimodal glioma analyses identify epigenetic regulators of cellular plasticity and environmental stress response. Nat Genet. 2021;53(10):1456–1468. 34594038 10.1038/s41588-021-00926-8 PMC8570135

[24] Leelatian N, Sinnaeve J, Mistry AM, et al Unsupervised machine learning reveals risk stratifying glioblastoma tumor cells. Elife. 2020;9:e56879. 32573435 10.7554/eLife.56879 PMC7340505

[25] Park SR, Namkoong S, Friesen L, et al Single-Cell Transcriptome Analysis of Colon Cancer Cell Response to 5-Fluorouracil-Induced DNA Damage. Cell Rep. 2020;32(8):108077. 32846134 10.1016/j.celrep.2020.108077 PMC7486130

[26] McCarthy AJ, Capo-Chichi JM, Spence T, et al Heterogenous loss of mismatch repair (MMR) protein expression: a challenge for immunohistochemical interpretation and microsatellite instability (MSI) evaluation. J Pathol Clin Res. 2019;5(2):115–129. 30387329 10.1002/cjp2.120 PMC6463865

[27] Tachon G, Frouin E, Karayan-Tapon L, et al Heterogeneity of mismatch repair defect in colorectal cancer and its implications in clinical practice. Eur J Cancer. 2018;95:112–116. 29519639 10.1016/j.ejca.2018.01.087

[28] von Loga K, Woolston A, Punta M, et al Extreme intratumour heterogeneity and driver evolution in mismatch repair deficient gastro-oesophageal cancer. Nat Commun. 2020;11(1):139. 31949146 10.1038/s41467-019-13915-7 PMC6965135

[29] Colaprico A, Silva TC, Olsen C, et al TCGAbiolinks: an R/Bioconductor package for integrative analysis of TCGA data. Nucleic Acids Res. 2016;44(8):e71. 26704973 10.1093/nar/gkv1507 PMC4856967

[30] Gao J, Aksoy BA, Dogrusoz U, et al Integrative analysis of complex cancer genomics and clinical profiles using the cBioPortal. Sci Signal. 2013;6(269):l1. 10.1126/scisignal.2004088 PMC4160307 23550210

[31] Cerami E, Gao J, Dogrusoz U, et al The cBio cancer genomics portal: an open platform for exploring multidimensional cancer genomics data. Cancer Discov. 2012;2(5):401–404. 22588877 10.1158/2159-8290.CD-12-0095 PMC3956037

[32] WHO Classification of Tumours Editorial Board. Central nervous system tumours. Vol 6. 5th ed. Lyon (France): International Agency for Research on Cancer; 2021.

[33] Louis DN, Wesseling P, Paulus W, et al cIMPACT-NOW update 1: Not Otherwise Specified (NOS) and Not Elsewhere Classified (NEC). Acta Neuropathol. 2018;135(3):481–484. 29372318 10.1007/s00401-018-1808-0

[34] Knijnenburg TA, Wang L, Zimmermann MT, et al; Cancer Genome Atlas Research Network. Genomic and Molecular Landscape of DNA Damage Repair Deficiency across The Cancer Genome Atlas. Cell Rep. 2018;23(1):239–254.e6. 29617664 10.1016/j.celrep.2018.03.076 PMC5961503

[35] Abugessaisa I, Noguchi S, Hasegawa A, et al refTSS: A Reference Data Set for Human and Mouse Transcription Start Sites. J Mol Biol. 2019;431(13):2407–2422. 31075273 10.1016/j.jmb.2019.04.045

[36] Anagnostou V, Niknafs N, Marrone K, et al Multimodal genomic features predict outcome of immune checkpoint blockade in non-small-cell lung cancer. Nat Cancer. 2020;1(1):99–111. 32984843 10.1038/s43018-019-0008-8 PMC7514475

[37] Aran D, Sirota M, Butte AJ. Systematic pan-cancer analysis of tumour purity. Nat Commun. 2015;6:8971. 26634437 10.1038/ncomms9971 PMC4671203

[38] Gu Z, Eils R, Schlesner M. Complex heatmaps reveal patterns and correlations in multidimensional genomic data. Bioinformatics. 2016;32(18):2847–2849. 27207943 10.1093/bioinformatics/btw313

[39] McInnes L, Healy J, Melville J. UMAP: Uniform Manifold Approximation and Projection for Dimension Reduction. 2018:arXiv:1802.03426. https://ui.adsabs.harvard.edu/abs/2018arXiv180203426M. Accessed February 01, 2018.

[40] Schneider CA, Rasband WS, Eliceiri KW. NIH Image to ImageJ: 25 years of image analysis. Nat Methods. 2012;9(7):671–675. 22930834 10.1038/nmeth.2089 PMC5554542

[41] Stirling DR, Swain-Bowden MJ, Lucas AM, et al CellProfiler 4: improvements in speed, utility and usability. BMC Bioinf. 2021;22(1):433. 10.1186/s12859-021-04344-9 PMC8431850 34507520

[42] Schapiro D, Jackson HW, Raghuraman S, et al histoCAT: analysis of cell phenotypes and interactions in multiplex image cytometry data. Nat Methods. 2017;14(9):873–876. 28783155 10.1038/nmeth.4391 PMC5617107

[43] Hahne F, Khodabakhshi AH, Bashashati A, et al Per-channel basis normalization methods for flow cytometry data. Cytometry A. 2010;77(2):121–131. 19899135 10.1002/cyto.a.20823 PMC3648208

[44] Levine JH, Simonds EF, Bendall SC, et al Data-Driven Phenotypic Dissection of AML Reveals Progenitor-like Cells that Correlate with Prognosis. Cell. 2015;162(1):184–197. 26095251 10.1016/j.cell.2015.05.047 PMC4508757

[45] Sturm D, Witt H, Hovestadt V, et al Hotspot mutations in H3F3A and IDH1 define distinct epigenetic and biological subgroups of glioblastoma. Cancer Cell. 2012;22(4):425–437. 23079654 10.1016/j.ccr.2012.08.024

[46] Yip S, Butterfield YS, Morozova O, et al Concurrent CIC mutations, IDH mutations, and 1p/19q loss distinguish oligodendrogliomas from other cancers. J Pathol. 2012;226(1):7–16. 22072542 10.1002/path.2995 PMC3246739

[47] Lee YS, Lee JK, Bae Y, et al Suppression of 14-3-3gamma-mediated surface expression of ANO1 inhibits cancer progression of glioblastoma cells. Sci Rep. 2016;6:26413. 27212225 10.1038/srep26413 PMC4876403

[48] Kim JA, Hicks WM, Li J, Tay SY, Haber JE. Protein phosphatases pph3, ptc2, and ptc3 play redundant roles in DNA double-strand break repair by homologous recombination. Mol Cell Biol. 2011;31(3):507–516. 21135129 10.1128/MCB.01168-10 PMC3028631

[49] Villoria MT, Gutierrez-Escribano P, Alonso-Rodriguez E, et al PP4 phosphatase cooperates in recombinational DNA repair by enhancing double-strand break end resection. Nucleic Acids Res. 2019;47(20):10706–10727. 31544936 10.1093/nar/gkz794 PMC6846210

[50] Li M, Li X, Xu S, et al Protein phosphatase 4 catalytic subunit is overexpressed in glioma and promotes glioma cell proliferation and invasion. Tumour Biol. 2016;37(9):11893–11901. 27059736 10.1007/s13277-016-5054-6

[51] Koslowski M, Tureci O, Bell C, et al Multiple splice variants of lactate dehydrogenase C selectively expressed in human cancer. Cancer Res. 2002;62(22):6750–6755. 12438276

[52] Eldai H, Periyasamy S, Al Qarni S, et al Novel genes associated with colorectal cancer are revealed by high resolution cytogenetic analysis in a patient specific manner. PLoS One. 2013;8(10):e76251. 24204606 10.1371/journal.pone.0076251 PMC3813709

[53] Wang Y, Xu H, Liu T, et al Temporal DNA-PK activation drives genomic instability and therapy resistance in glioma stem cells. JCI Insight. 2018;3(3):e98096. 29415883 10.1172/jci.insight.98096 PMC5821187

[54] Xi G, Hayes E, Lewis R, et al CD133 and DNA-PK regulate MDR1 via the PI3K- or Akt-NF-kappaB pathway in multidrug-resistant glioblastoma cells in vitro. Oncogene. 2016;35(42):5576. 10.1038/onc.2016.64 27593924

[55] Zhuang W, Li B, Long L, et al Knockdown of the DNA-dependent protein kinase catalytic subunit radiosensitizes glioma-initiating cells by inducing autophagy. Brain Res. 2011;1371:7–15. 21108935 10.1016/j.brainres.2010.11.044

[56] Squatrito M, Vanoli F, Schultz N, Jasin M, Holland EC. 53BP1 is a haploinsufficient tumor suppressor and protects cells from radiation response in glioma. Cancer Res. 2012;72(20):5250–5260. 22915756 10.1158/0008-5472.CAN-12-0045 PMC3771704

[57] Johannessen TC, Prestegarden L, Grudic A, et al The DNA repair protein ALKBH2 mediates temozolomide resistance in human glioblastoma cells. Neuro Oncol. 2013;15(3):269–278. 23258843 10.1093/neuonc/nos301 PMC3578482

[58] Amir el AD, Davis KL, Tadmor MD, et al viSNE enables visualization of high dimensional single-cell data and reveals phenotypic heterogeneity of leukemia. Nat Biotechnol. 2013;31(6):545–552. 23685480 10.1038/nbt.2594 PMC4076922

[59] Bao S, Wu Q, McLendon RE, et al Glioma stem cells promote radioresistance by preferential activation of the DNA damage response. Nature. 2006;444(7120):756–760. 17051156 10.1038/nature05236

[60] Carruthers RD, Ahmed SU, Ramachandran S, et al Replication Stress Drives Constitutive Activation of the DNA Damage Response and Radioresistance in Glioblastoma Stem-like Cells. Cancer Res. 2018;78(17):5060–5071. 29976574 10.1158/0008-5472.CAN-18-0569 PMC6128404

