# Supplementary material for: DNA damage response profile distinguishes poor-acting gliomas with shared methylome signatures
Source: Neuro Oncol. 2025 Aug 27;28(1):117–29. doi: 10.1093/neuonc/noaf199 (PMC12962623; doi:10.1093/neuonc/noaf199)
Supplement: noaf199_Supplementary_Data [file noaf199_supplementary_data.zip › noaf199_suppl_Supplementary_Tables_S3.docx]

| **Table S3 Imaging mass cytometry antibodies** | | | | | |  |  |  |
| --- | --- | --- | --- | --- | --- | --- | --- | --- |
|  |  |  |  |  |  |  |  |  |
| **Targets** | **Channels** | **Markers** | | | **tSNE and Phenograph analyses (Fig 4b-f)** | **Dilutions** | **Clones** | **Sources** |
|  |  | **DDR** | **Identity** | **Cell compartment** |  |  |  |  |
| CHK2 | 141Pr |  |  |  | **x** | 0.25 ug/mL | 1C12 | CST |
| RPA32 | 142Nd |  |  |  | **x** | 0.02 ug/mL | E8X5P | CST |
| p-CHK2 | 146Nd |  |  |  |  | 9.4 ug/mL | E8Q1A | CST |
| APE1 | 150Nd |  |  |  | **x** | 0.025 ug/mL | E5Y2C | CST |
| CD31 | 151Eu |  |  |  |  | 1:200 | EPR3094 | Standard Biotools |
| CD45 | 152Sm |  |  |  |  | 1:200 | D9C6 | Standard Biotools |
| 53BP1 | 153Eu |  |  |  | **x** | 0.25 ug/mL | E7N5D | CST |
| IBA1 | 155Gd |  |  |  |  | 0.02 ug/mL | E4O4W | CST |
| PCNA | 158Gd |  |  |  | **x** | 0.015 ug/mL | D3H8P | CST |
| MLH1 | 160Gd |  |  |  | **x** | 1.5 ug/mL | EPR3894 | Abcam |
| NeuN | 161Dy |  |  |  |  | 0.09 ug/mL | D4G4O | CST |
| MSH6 | 163Dy |  |  |  | **x** | 1.75 ug/mL | 3E1 | CST |
| DNA-PKcs | 164Dy |  |  |  | **x** | 0.07 ug/mL | E6U3A | CST |
| γH2AX | 165Ho |  |  |  | **x** | 1:100 | N1-431 | Standard Biotools |
| MRE11 | 166Er |  |  |  | **x** | 2.6 ug/mL | 31H4 | CST |
| PARP1 | 167Er |  |  |  | **x** | 0.7 ug/mL | EPR18461 | Abcam |
| Ki67 | 168Er |  |  |  | **x** | 1:100 | B56 | Standard Biotools |
| mIDH1 | 169Tm |  |  |  | **x** | 1.0 ug/mL | DIA-H09 | Dianova |
| BRCA1 | 172Yb |  |  |  | **x** | 1:50 | MS110 | Standard Biotools |
| GFAP | 175Lu |  |  |  |  | 0.5 ug/mL | GA5 | CST |
| Histone H3 | 176Yb |  |  |  |  | 1:600 | D1H2 | Standard Biotools |
| Ir | 191Ir |  |  |  |  |  |  | Standard Biotools |
| Ir | 193Ir |  |  |  |  |  |  | Standard Biotools |
| ICSK1 | 195Pt |  |  |  |  |  |  | Standard Biotools |
| ICSK2 | 196Pt |  |  |  |  |  |  | Standard Biotools |
| ICSK3 | 198Pt |  |  |  |  |  |  | Standard Biotools |
