# Supplementary material for: DNA damage response profile distinguishes poor-acting gliomas with shared methylome signatures
Source: Neuro Oncol. 2025 Aug 27;28(1):117–29. doi: 10.1093/neuonc/noaf199 (PMC12962623; doi:10.1093/neuonc/noaf199)
Supplement: noaf199_Supplementary_Data [file noaf199_supplementary_data.zip › noaf199_suppl_Supplementary_Tables_S4.docx]

| **Table S4: Tissue microarray case information** | | | |  |  |  |  |  |  |  |  |  |  |  |  |  |  |  |  |  |
| --- | --- | --- | --- | --- | --- | --- | --- | --- | --- | --- | --- | --- | --- | --- | --- | --- | --- | --- | --- | --- |
|  |  |  |  |  |  |  |  |  |  |  |  |  |  |  |  |  |  |  |  |  |
| **Case** | **Progression (1=yes)** | **PFS (days)** | **Death (1=yes)** | **OS (days)** | **Diagnosis** | **Grade** | **Methylation** | **Status** | **Cell subset abundance (% of glioma cells)** | | | | | | | | | | | |
|  |  |  |  |  |  |  |  |  | **G02** | **G03** | **G04** | **G01** | **G08** | **G06** | **G12** | **G11** | **G09** | **G10** | **G07** | **G05** |
| R10C09 | 1 | 1541 | 0 | 4159 | Astrocytoma | Low | Unmethylated | Recurrent | 28.2 | 4.73 | 14.2 | 1.51 | 0.38 | 14 | 3.78 | 0.38 | 9.45 | 0 | 22.5 | 0.95 |
| R12C07 | 1 | 2314 | 0 | 4886 | Glioma IDHm | Low | NA | Recurrent | 24.2 | 31.8 | 12.9 | 3.78 | 0 | 0.95 | 2.84 | 0 | 12.7 | 0 | 10 | 0.95 |
| R07C04 | 1 | 438 | 0 | 494 | Glioblastoma | High | Methylated or partial | Primary | 0.76 | 3.8 | 82.5 | 0.25 | 0 | 6.58 | 1.01 | 0 | 0 | 0.25 | 0.25 | 4.56 |
| R12C06 | 1 | 1076 | 1 | 2784 | Astrocytoma | Low | Unmethylated | Primary | 5.48 | 4.54 | 3.78 | 4.73 | 0 | 0.38 | 0.19 | 0 | 3.21 | 37.2 | 40.3 | 0.19 |
| R14C01 | 0 | 1980 | 0 | 1980 | Oligodendroglioma | Low | Methylated or partial | Recurrent | 3.21 | 25 | 60.3 | 4.73 | 0.19 | 5.67 | 0 | 0 | 0.19 | 0.19 | 0.19 | 0.38 |
| R06C06 | 1 | 649 | 0 | 946 | Astrocytoma Gr4 | High | Unmethylated | Primary | 17 | 17.4 | 28.2 | 3.4 | 0.76 | 1.89 | 1.32 | 0.57 | 0.57 | 4.16 | 23.1 | 1.7 |
| R13C01 | 1 | 2040 | 0 | 3570 | Astrocytoma Gr4 | High | NA | Primary | 0.76 | 16.4 | 62 | 5.1 | 0.57 | 4.91 | 0.19 | 0.19 | 0 | 0.38 | 8.88 | 0.57 |
| R08C09 | 0 | 1521 | 0 | 1521 | Oligodendroglioma | Low | Methylated or partial | Primary | 29.3 | 21.4 | 22.3 | 5.29 | 0.19 | 14.2 | 0.57 | 0.57 | 1.89 | 0 | 2.65 | 1.7 |
| R14C08 | 0 | 1222 | 0 | 1222 | Oligodendroglioma | Low | Methylated or partial | Primary | 18.1 | 9.64 | 3.97 | 4.16 | 0.76 | 0.95 | 0 | 0 | 15.1 | 9.64 | 34.8 | 2.84 |
| R07C08 | 1 | 399 | 1 | 446 | Glioblastoma | High | Methylated or partial | Primary | 17.7 | 25.3 | 37.5 | 2.03 | 3.54 | 7.85 | 0 | 1.52 | 0.51 | 0.25 | 0.76 | 3.04 |
| R09C02 | 0 | 1381 | 0 | 1381 | Astrocytoma | Low | Methylated or partial | Primary | 33.1 | 11.3 | 30.6 | 4.35 | 4.91 | 6.05 | 4.35 | 2.08 | 1.89 | 0.19 | 0.95 | 0.19 |
| R13C06 | 1 | 271 | 1 | 1989 | Oligodendroglioma | Low | Methylated or partial | Primary | 20 | 10.2 | 3.4 | 5.86 | 0.57 | 2.46 | 0.76 | 0.19 | 10.8 | 0.19 | 42.5 | 3.02 |
| R10C01 | 1 | 3860 | 1 | 4433 | Astrocytoma | Low | Methylated or partial | Recurrent | 11.3 | 5.67 | 13 | 6.05 | 3.02 | 1.32 | 0.19 | 0.38 | 32.7 | 8.51 | 17.2 | 0.57 |
| R13C07 | 0 | 1919 | 0 | 1919 | Oligodendroglioma | Low | Methylated or partial | Primary | 22.9 | 25.1 | 14.7 | 9.83 | 0.19 | 1.89 | 1.51 | 1.7 | 12.3 | 0.38 | 8.51 | 0.95 |
| R11C08 | 1 | 487 | 0 | 971 | Glioblastoma | High | Unmethylated | Primary | 29.4 | 31.1 | 17.5 | 4.3 | 0.51 | 1.27 | 1.52 | 1.52 | 1.01 | 0 | 5.57 | 6.33 |
| R07C05 | 0 | 947 | 1 | 947 | Glioblastoma | High | Methylated or partial | Primary | 32.9 | 38 | 10.6 | 6.58 | 0.76 | 2.28 | 2.53 | 1.01 | 0.25 | 0.25 | 0 | 4.81 |
| R06C08 | 1 | 112 | 0 | 800 | Oligodendroglioma | Low | Methylated or partial | Primary | 21.7 | 21 | 5.48 | 8.51 | 0 | 16.1 | 2.08 | 2.46 | 17.8 | 0 | 0.57 | 4.35 |
| R04C04 | 1 | 148 | 0 | 380 | Glioblastoma | High | Unmethylated | Primary | 16.2 | 14.7 | 6.08 | 10.1 | 2.78 | 43.8 | 0.76 | 3.29 | 1.27 | 0.25 | 0.51 | 0.25 |
| R13C08 | 1 | 200 | 0 | 1484 | Astrocytoma | Low | Methylated or partial | Primary | 10.2 | 12.5 | 6.43 | 12.7 | 0.57 | 2.65 | 1.7 | 0.38 | 2.65 | 25 | 25.3 | 0 |
| R03C07 | 0 | 102 | 0 | 102 | Glioblastoma | High | Unmethylated | Primary | 14.4 | 14.4 | 14.2 | 3.8 | 0.25 | 41.3 | 0.76 | 0.25 | 0.76 | 0 | 0.25 | 9.62 |
| R06C03 | 1 | 324 | 1 | 613 | Glioblastoma | High | Unmethylated | Primary | 24.6 | 26.3 | 24.8 | 5.06 | 3.54 | 7.59 | 0.25 | 0.76 | 0.25 | 0 | 0.76 | 6.08 |
| R14C07 | 1 | 1319 | 0 | 1723 | Oligodendroglioma | Low | Methylated or partial | Primary | 29.7 | 21.7 | 11.2 | 12.5 | 0.19 | 5.29 | 0.95 | 1.32 | 12.5 | 0.19 | 2.46 | 2.08 |
| R10C04 | 1 | 94 | 1 | 149 | Glioblastoma | High | Methylated or partial | Primary | 47.8 | 23.3 | 5.82 | 10.6 | 0 | 1.52 | 1.52 | 0.25 | 0.76 | 0.25 | 3.8 | 4.3 |
| R02C04 | 1 | 104 | 1 | 120 | Glioblastoma | High | Methylated or partial | Primary | 31.1 | 24.6 | 12.2 | 4.56 | 0.51 | 4.56 | 2.03 | 0.51 | 0.76 | 0.25 | 8.61 | 10.4 |
| R03C09 | 1 | 320 | 1 | 360 | Glioblastoma | High | Unmethylated | Primary | 33.7 | 6.58 | 18.2 | 2.03 | 13.4 | 21.5 | 1.77 | 0.25 | 0.51 | 0 | 1.77 | 0.25 |
| R09C05 | 1 | 862 | 1 | 923 | Astrocytoma | Low | Methylated or partial | Primary | 9.64 | 4.16 | 1.51 | 15.1 | 1.13 | 4.35 | 4.35 | 0.76 | 23.6 | 3.02 | 32.1 | 0.19 |
| R09C03 | 0 | 825 | 1 | 825 | Oligodendroglioma | Low | Methylated or partial | Primary | 16.6 | 14.2 | 9.26 | 14.6 | 0.19 | 16.8 | 4.35 | 1.7 | 7.56 | 4.73 | 8.32 | 1.7 |
| R08C01 | 1 | 156 | 0 | 1141 | Glioblastoma | High | Methylated or partial | Primary | 29.1 | 26.6 | 17.5 | 11.1 | 0.51 | 8.61 | 0.25 | 0.51 | 0.25 | 0 | 0.25 | 5.32 |
| R08C08 | 0 | 1562 | 0 | 1562 | Astrocytoma | Low | Methylated or partial | Primary | 17 | 16.1 | 4.91 | 14.9 | 0.95 | 2.08 | 0.19 | 0.19 | 0.38 | 7.56 | 33.6 | 2.08 |
| R09C07 | 1 | 240 | 1 | 414 | Glioblastoma | High | Unmethylated | Recurrent | 3.04 | 31.6 | 41 | 11.9 | 2.53 | 3.8 | 0.76 | 0.25 | 0 | 0 | 1.52 | 3.54 |
| R08C07 | 1 | 4507 | 0 | 6335 | Astrocytoma Gr4 | High | Methylated or partial | Recurrent | 32.1 | 21.6 | 9.64 | 16.8 | 0.76 | 3.21 | 0.57 | 0 | 5.67 | 0 | 8.7 | 0.95 |
| R04C09 | 1 | 241 | 1 | 551 | Glioblastoma | High | Unmethylated | Primary | 28.6 | 19.2 | 18.5 | 14.4 | 1.01 | 9.37 | 2.03 | 1.01 | 0 | 0 | 2.28 | 3.54 |
| R08C06 | 0 | 1653 | 0 | 1653 | Astrocytoma Gr4 | High | Methylated or partial | Primary | 25.3 | 21.2 | 18.3 | 15.1 | 3.4 | 1.32 | 2.46 | 0.57 | 9.26 | 0.19 | 1.7 | 1.13 |
| R07C01 | 1 | 281 | 0 | 880 | Astrocytoma | Low | Methylated or partial | Primary | 12.1 | 15.9 | 9.26 | 17 | 0.38 | 15.1 | 1.51 | 0.38 | 2.84 | 1.51 | 21.7 | 2.27 |
| R05C02 | 1 | 829 | 0 | 1188 | Glioblastoma | High | Unmethylated | Primary | 6.08 | 15.2 | 4.05 | 11.1 | 9.37 | 2.78 | 1.27 | 0.76 | 0 | 15.9 | 33.4 | 0 |
| R05C01 | 1 | 35 | 1 | 35 | Glioblastoma | High | Unmethylated | Primary | 29.6 | 20.3 | 14.9 | 10.6 | 2.78 | 7.34 | 0 | 0.76 | 0.51 | 0.51 | 5.06 | 7.59 |
| R13C05 | 1 | 1541 | 0 | 4159 | Astrocytoma | Low | Unmethylated | Recurrent | 16.6 | 19.5 | 14.4 | 19.5 | 0.95 | 6.43 | 0.95 | 0.38 | 4.35 | 1.89 | 14.4 | 0.76 |
| R01C08 | 1 | 438 | 1 | 607 | Glioblastoma | High | Methylated or partial | Primary | 22.3 | 16.5 | 18.5 | 7.85 | 0 | 21 | 0.25 | 0.25 | 0 | 0 | 0 | 13.4 |
| R13C04 | 1 | 301 | 1 | 688 | Glioblastoma | High | Unmethylated | Primary | 20.3 | 25.3 | 13.7 | 20 | 0.25 | 13.2 | 1.01 | 2.28 | 1.27 | 1.01 | 0.76 | 1.01 |
| R02C09 | 0 | 130 | 0 | 130 | Glioblastoma | High | Unmethylated | Primary | 25.3 | 23.5 | 19 | 11.9 | 1.27 | 7.85 | 0.76 | 0.51 | 0.76 | 0.25 | 0.25 | 8.61 |
| R12C09 | 0 | 1980 | 0 | 1980 | Oligodendroglioma | Low | Methylated or partial | Primary | 20 | 28 | 23.4 | 18 | 2.65 | 1.32 | 0.19 | 0.19 | 0.38 | 0.19 | 4.35 | 1.32 |
| R06C09 | 0 | 1394 | 0 | 1394 | Oligodendroglioma | Low | Methylated or partial | Primary | 26.7 | 28.7 | 10.8 | 13.2 | 7.75 | 0.57 | 0.57 | 0.19 | 6.43 | 0.19 | 2.84 | 2.08 |
| R07C06 | 1 | 87 | 1 | 200 | Glioblastoma | High | Methylated or partial | Primary | 23.8 | 34.9 | 11.4 | 20.3 | 0 | 2.78 | 0.25 | 3.04 | 0 | 0 | 0 | 3.54 |
| R11C04 | 1 | 829 | 0 | 1597 | Oligodendroglioma | Low | Methylated or partial | Primary | 13.8 | 14 | 3.78 | 20 | 1.32 | 7.18 | 2.27 | 1.7 | 16.4 | 0.95 | 15.9 | 2.65 |
| R10C03 | 1 | 266 | 1 | 585 | Astrocytoma Gr4 | High | Methylated or partial | Primary | 27.1 | 22 | 13.7 | 6.84 | 0.25 | 5.82 | 0.25 | 0.51 | 1.27 | 0 | 4.56 | 17.7 |
| R08C03 | 0 | 1486 | 0 | 1486 | Astrocytoma Gr4 | High | Methylated or partial | Primary | 7.09 | 5.06 | 1.52 | 17 | 6.33 | 5.82 | 2.03 | 1.27 | 28.4 | 3.8 | 18.2 | 3.54 |
| R09C04 | 0 | 1208 | 0 | 1208 | Oligodendroglioma | Low | Methylated or partial | Primary | 18.5 | 15.7 | 3.97 | 26.3 | 0.95 | 10.2 | 4.16 | 0.57 | 0.95 | 2.08 | 15.7 | 0.95 |
| R03C06 | 1 | 369 | 1 | 752 | Glioblastoma | High | Unmethylated | Recurrent | 20.5 | 34.2 | 15.2 | 22.5 | 4.56 | 0.76 | 0 | 0.51 | 0.25 | 0 | 0.25 | 1.27 |
| R02C07 | 0 | 420 | 0 | 420 | Glioblastoma | High | Methylated or partial | Primary | 21.3 | 26.3 | 22.8 | 20.8 | 1.01 | 0.25 | 0.51 | 0.25 | 0 | 0 | 0 | 6.84 |
| R04C07 | 0 | 102 | 1 | 102 | Glioblastoma | High | Methylated or partial | Primary | 29.4 | 24.3 | 9.62 | 24.6 | 2.78 | 4.3 | 0.25 | 0.25 | 0.51 | 0 | 2.28 | 1.77 |
| R01C09 | 1 | 295 | 0 | 408 | Glioblastoma | High | Unmethylated | Primary | 18.5 | 24.1 | 20.3 | 20 | 3.29 | 1.77 | 0 | 1.27 | 0.51 | 0 | 4.3 | 6.08 |
| R09C01 | 1 | 1419 | 0 | 1500 | Oligodendroglioma | Low | Methylated or partial | Primary | 14.4 | 4.73 | 0.76 | 25.1 | 4.35 | 4.54 | 0.19 | 0.19 | 7.75 | 25.5 | 12.1 | 0.38 |
| R01C07 | 1 | 420 | 1 | 420 | Glioblastoma | High | Methylated or partial | Primary | 13.7 | 2.53 | 14.7 | 0 | 0.25 | 0.76 | 0.76 | 34.4 | 2.28 | 0 | 0.51 | 30.1 |
| R04C10 | 0 | 60 | 0 | 60 | Glioblastoma | High | Unmethylated | Primary | 12.2 | 12.4 | 4.05 | 22 | 5.57 | 6.58 | 0.25 | 0.51 | 1.52 | 3.04 | 28.6 | 3.29 |
| R01C10 | 1 | 968 | 0 | 997 | Glioblastoma | High | Methylated or partial | Primary | 28.9 | 21.8 | 3.8 | 28.4 | 0.51 | 4.3 | 9.11 | 0.76 | 0.51 | 0 | 0 | 2.03 |
| R09C08 | 1 | 872 | 1 | 1367 | Glioblastoma | High | Methylated or partial | Recurrent | 20.3 | 15.4 | 8.86 | 16.5 | 9.37 | 15.2 | 3.54 | 2.03 | 0 | 0 | 1.77 | 7.09 |
| R14C05 | 0 | 1873 | 0 | 1873 | Oligodendroglioma | Low | Methylated or partial | Primary | 22.5 | 24.6 | 4.73 | 29.3 | 0.95 | 3.21 | 0.57 | 2.08 | 3.59 | 0 | 5.67 | 2.84 |
| R10C06 | 1 | 96 | 1 | 403 | Glioblastoma | High | Unmethylated | Primary | 17.7 | 26.6 | 15.4 | 24.3 | 1.01 | 4.81 | 1.27 | 0.25 | 0.25 | 0 | 0.51 | 7.85 |
| R05C07 | 1 | 399 | 0 | 809 | Glioblastoma | High | Unmethylated | Primary | 23.5 | 24.1 | 10.1 | 21.5 | 0.51 | 1.77 | 1.01 | 0.25 | 1.77 | 0.25 | 3.54 | 11.6 |
| R01C01 | 1 | 192 | 0 | 452 | Glioblastoma | High | Unmethylated | Primary | 10.4 | 9.87 | 2.53 | 24.6 | 8.86 | 35.9 | 2.28 | 1.77 | 2.03 | 0.25 | 0.25 | 1.27 |
| R13C02 | 1 | 990 | 1 | 1131 | Glioblastoma | High | Unmethylated | Primary | 12.2 | 5.82 | 1.01 | 23.8 | 8.35 | 8.61 | 0 | 0.51 | 0.76 | 9.87 | 26.1 | 3.04 |
| R01C05 | 0 | 1301 | 0 | 1301 | Glioblastoma | High | Methylated or partial | Primary | 20.5 | 21 | 12.2 | 23.5 | 1.77 | 4.56 | 2.28 | 0.51 | 3.54 | 0 | 0 | 10.1 |
| R07C09 | 1 | 124 | 1 | 33164 | Glioblastoma | High | Unmethylated | Primary | 15.9 | 20 | 10.6 | 21.5 | 2.28 | 9.62 | 0 | 1.52 | 6.08 | 0 | 0.51 | 11.9 |
| R08C05 | 0 | 1667 | 0 | 1667 | Astrocytoma | Low | Methylated or partial | Recurrent | 25.9 | 14 | 2.84 | 28.7 | 7.75 | 11 | 3.21 | 0.57 | 5.48 | 0.19 | 0.19 | 0.19 |
| R08C10 | 0 | 1558 | 0 | 1558 | Oligodendroglioma | Low | Methylated or partial | Primary | 13.6 | 22.3 | 6.62 | 31.8 | 1.51 | 10.4 | 0.95 | 1.32 | 0.19 | 0.57 | 7.37 | 3.4 |
| R01C06 | 1 | 383 | 0 | 897 | Glioblastoma | High | Methylated or partial | Primary | 14.9 | 18 | 3.8 | 33.4 | 1.52 | 22 | 1.01 | 0.76 | 2.28 | 0 | 0 | 2.28 |
| R14C06 | 1 | 217 | 1 | 1463 | Astrocytoma Gr4 | High | Unmethylated | Recurrent | 26.3 | 13.9 | 11.4 | 9.87 | 26.8 | 9.87 | 0 | 0.25 | 0.51 | 0.25 | 0 | 0.76 |
| R05C06 | 1 | 48 | 0 | 190 | Glioblastoma | High | Unmethylated | Primary | 29.1 | 21.3 | 10.4 | 16.2 | 6.58 | 1.27 | 0 | 0 | 0.51 | 0 | 0 | 14.7 |
| R10C08 | 1 | 251 | 1 | 463 | Glioblastoma | High | Methylated or partial | Recurrent | 6.84 | 20.5 | 8.61 | 13.4 | 13.7 | 20 | 0.51 | 4.05 | 1.01 | 0 | 1.01 | 10.4 |
| R12C10 | 1 | 217 | 1 | 1463 | Astrocytoma Gr4 | High | Methylated or partial | Recurrent | 5.82 | 1.77 | 0.51 | 14.4 | 3.29 | 13.4 | 0.25 | 1.01 | 8.86 | 3.29 | 26.8 | 20.5 |
| R02C01 | 1 | 554 | 1 | 554 | Glioblastoma | High | Methylated or partial | Primary | 31.1 | 15.7 | 4.56 | 18.2 | 7.34 | 4.05 | 0.25 | 2.28 | 0 | 0 | 3.04 | 13.4 |
| R05C08 | 1 | 163 | 1 | 242 | Glioblastoma | High | Unmethylated | Primary | 17.2 | 14.4 | 10.1 | 12.7 | 26.1 | 12.7 | 0.51 | 3.8 | 0.51 | 0 | 1.27 | 0.76 |
| R01C04 | 1 | 19 | 0 | 674 | Glioblastoma | High | Methylated or partial | Primary | 16.2 | 12.9 | 6.58 | 29.9 | 5.06 | 10.9 | 2.03 | 1.01 | 9.87 | 0.51 | 0 | 5.06 |
| R12C01 | 1 | 361 | 0 | 482 | Glioblastoma | High | Methylated or partial | Primary | 21 | 20.8 | 7.34 | 29.4 | 1.77 | 3.04 | 0.76 | 1.52 | 0.51 | 0 | 5.06 | 8.86 |
| R09C09 | 1 | 167 | 1 | 319 | Glioblastoma | High | Unmethylated | Recurrent | 18.7 | 23 | 11.9 | 22 | 9.11 | 3.29 | 0 | 0.25 | 1.77 | 0.25 | 0.25 | 9.37 |
| R06C05 | 1 | 168 | 0 | 300 | Glioblastoma | High | Unmethylated | Primary | 25.1 | 21.5 | 7.09 | 35.2 | 2.03 | 0.76 | 2.03 | 0 | 1.52 | 0 | 1.52 | 3.29 |
| R02C08 | 1 | 123 | 1 | 123 | Glioblastoma | High | Unmethylated | Primary | 20.8 | 23.3 | 9.62 | 23.5 | 1.01 | 4.81 | 0.25 | 0 | 0.25 | 0 | 0.25 | 16.2 |
| R06C04 | 1 | 91 | 1 | 174 | Glioblastoma | High | Unmethylated | Primary | 13.4 | 10.9 | 5.57 | 29.4 | 9.87 | 22 | 0.51 | 3.29 | 3.29 | 0.25 | 0 | 1.52 |
| R05C04 | 0 | 263 | 1 | 263 | Glioblastoma | High | Unmethylated | Primary | 16.5 | 8.61 | 2.03 | 32.2 | 4.81 | 18 | 0.25 | 5.57 | 0.51 | 7.34 | 0.25 | 4.05 |
| R14C03 | 1 | 708 | 0 | 974 | Astrocytoma | Low | Methylated or partial | Recurrent | 28.2 | 11.9 | 1.51 | 36.3 | 5.1 | 6.43 | 0.57 | 0.38 | 3.78 | 0 | 5.48 | 0.38 |
| R07C03 | 0 | 1061 | 0 | 1061 | Oligodendroglioma | Low | Methylated or partial | Primary | 28.4 | 13.2 | 2.84 | 34.4 | 0.38 | 2.84 | 4.73 | 0 | 4.35 | 0 | 1.51 | 7.37 |
| R04C06 | 1 | 123 | 1 | 379 | Glioblastoma | High | Unmethylated | Primary | 25.8 | 18.5 | 5.32 | 32.9 | 1.77 | 6.08 | 0.25 | 0.51 | 0.76 | 0.25 | 0 | 7.85 |
| R12C02 | 1 | 1052 | 0 | 1638 | Glioblastoma | High | Methylated or partial | Primary | 22.8 | 19.5 | 6.58 | 31.1 | 2.03 | 4.56 | 0.51 | 0.51 | 1.27 | 0.25 | 1.01 | 9.87 |
| R11C03 | 0 | 1443 | 0 | 1443 | Astrocytoma | Low | Methylated or partial | Primary | 25.1 | 15.3 | 3.21 | 31.4 | 11.2 | 3.59 | 0.95 | 0.76 | 3.59 | 0.19 | 4.16 | 0.57 |
| R10C02 | 1 | 301 | 1 | 688 | Glioblastoma | High | Unmethylated | Recurrent | 18 | 9.87 | 4.05 | 33.7 | 2.78 | 7.85 | 1.52 | 5.06 | 9.11 | 0 | 0.51 | 7.59 |
| R08C04 | 1 | 118 | 0 | 196 | Glioblastoma | High | Methylated or partial | Primary | 22 | 19.5 | 3.29 | 30.6 | 1.01 | 4.05 | 3.8 | 1.27 | 0.51 | 0 | 1.27 | 12.7 |
| R10C07 | 1 | 2042 | 0 | 3570 | Astrocytoma Gr4 | High | Methylated or partial | Recurrent | 17.5 | 11.6 | 6.33 | 10.1 | 0 | 11.6 | 0.25 | 0.51 | 7.59 | 0 | 0 | 34.4 |
| R04C08 | 1 | 190 | 1 | 364 | Glioblastoma | High | Unmethylated | Primary | 18.2 | 20.5 | 13.9 | 25.1 | 0 | 0.76 | 0.25 | 0.76 | 0.51 | 0 | 0 | 20 |
| R05C05 | 1 | 167 | 1 | 384 | Glioblastoma | High | Unmethylated | Primary | 22.5 | 20.5 | 3.04 | 39.5 | 1.52 | 1.77 | 0.25 | 1.77 | 0.76 | 0 | 3.54 | 4.81 |
| R11C06 | 1 | 751 | 0 | 901 | Glioblastoma | High | Methylated or partial | Recurrent | 20 | 21.5 | 7.34 | 34.4 | 0.76 | 2.28 | 1.52 | 0 | 0.25 | 0 | 1.01 | 10.9 |
| R05C03 | 1 | 88 | 1 | 88 | Glioblastoma | High | Unmethylated | Primary | 25.8 | 14.2 | 2.78 | 34.9 | 3.04 | 4.3 | 1.27 | 0.76 | 0.76 | 0.51 | 2.78 | 8.86 |
| R12C03 | 1 | 154 | 0 | 1028 | Glioblastoma | High | Unmethylated | Primary | 17.7 | 20.5 | 8.86 | 33.2 | 8.1 | 3.29 | 1.27 | 1.52 | 0 | 0 | 0 | 5.57 |
| R07C07 | 0 | 1945 | 0 | 1945 | Glioblastoma | High | Methylated or partial | Primary | 19.7 | 11.9 | 4.56 | 16.2 | 26.6 | 0.25 | 0 | 0 | 0 | 5.57 | 10.1 | 5.06 |
| R11C05 | 1 | 99 | 0 | 201 | Glioblastoma | High | Unmethylated | Recurrent | 3.29 | 0.76 | 0.25 | 6.84 | 41.8 | 11.1 | 0.25 | 0 | 1.27 | 34.2 | 0.25 | 0 |
| R06C02 | 1 | 726 | 1 | 726 | Glioblastoma | High | Unmethylated | Primary | 12.7 | 9.87 | 3.04 | 30.6 | 1.01 | 3.8 | 1.01 | 0.51 | 1.27 | 1.01 | 17.7 | 17.5 |
| R02C03 | 1 | 311 | 1 | 311 | Glioblastoma | High | Methylated or partial | Primary | 13.7 | 17 | 7.34 | 27.8 | 7.34 | 6.84 | 3.04 | 1.01 | 0.51 | 0 | 1.01 | 14.4 |
| R06C01 | 1 | 284 | 1 | 329 | Glioblastoma | High | Unmethylated | Primary | 17.5 | 12.4 | 5.06 | 34.4 | 1.52 | 5.57 | 2.03 | 2.78 | 0.76 | 0.25 | 3.29 | 14.4 |
| R02C05 | 1 | 246 | 1 | 246 | Glioblastoma | High | Methylated or partial | Primary | 25.6 | 12.9 | 3.29 | 33.4 | 0 | 6.33 | 1.01 | 0.25 | 0 | 0 | 0.25 | 17 |
| R02C02 | 1 | 89 | 1 | 338 | Glioblastoma | High | Unmethylated | Primary | 17.2 | 14.7 | 2.28 | 40 | 1.27 | 2.78 | 1.01 | 9.87 | 1.01 | 0 | 0.25 | 9.62 |
| R14C09 | 1 | 377 | 1 | 614 | Glioblastoma | High | Methylated or partial | Primary | 23.8 | 15.4 | 7.34 | 17 | 1.52 | 1.01 | 0.76 | 0 | 0 | 0 | 0 | 33.2 |
| R01C03 | 0 | 21 | 0 | 21 | Glioblastoma | High | Methylated or partial | Primary | 18.2 | 9.11 | 2.03 | 25.6 | 9.37 | 2.78 | 1.01 | 1.01 | 1.01 | 1.01 | 11.9 | 17 |
| R04C02 | 1 | 117 | 1 | 117 | Glioblastoma | High | Unmethylated | Primary | 13.4 | 14.2 | 4.05 | 31.1 | 1.52 | 1.01 | 1.27 | 11.6 | 0.51 | 0 | 0.76 | 20.5 |
| R03C05 | 1 | 202 | 1 | 400 | Glioblastoma | High | Methylated or partial | Primary | 16.5 | 9.87 | 0.25 | 50.4 | 0.25 | 14.4 | 0.25 | 0.25 | 0.76 | 0 | 2.78 | 4.3 |
| R04C01 | 1 | 329 | 1 | 488 | Glioblastoma | High | Unmethylated | Primary | 20.8 | 11.1 | 1.77 | 40.5 | 14.2 | 2.03 | 1.27 | 0.76 | 3.29 | 0.25 | 3.54 | 0.51 |
| R01C02 | 0 | 163 | 0 | 163 | Glioblastoma | High | Methylated or partial | Primary | 16.2 | 19.5 | 1.01 | 48.9 | 0.76 | 2.53 | 1.01 | 1.01 | 1.27 | 0 | 1.77 | 6.08 |
| R04C05 | 0 | 70 | 1 | 70 | Glioblastoma | High | Unmethylated | Primary | 13.4 | 4.81 | 0.25 | 31.6 | 23.5 | 19.2 | 0.76 | 1.77 | 2.28 | 1.27 | 0 | 1.01 |
| R05C09 | 1 | 95 | 0 | 164 | Glioblastoma | High | Unmethylated | Primary | 15.7 | 11.9 | 10.6 | 15.2 | 8.1 | 2.28 | 1.01 | 0.76 | 0 | 0 | 1.52 | 32.9 |
| R03C03 | 1 | 137 | 1 | 137 | Glioblastoma | High | Methylated or partial | Primary | 16.2 | 12.4 | 4.3 | 15.9 | 21.8 | 2.53 | 1.77 | 1.27 | 0.25 | 0.25 | 4.05 | 19.2 |
| R04C03 | 1 | 116 | 0 | 164 | Glioblastoma | High | Unmethylated | Primary | 17 | 16.5 | 4.3 | 42.8 | 8.61 | 2.53 | 2.28 | 0.25 | 0 | 0 | 0.25 | 5.57 |
| R03C02 | 1 | 712 | 0 | 966 | Glioblastoma | High | Methylated or partial | Primary | 20.8 | 12.2 | 3.8 | 22 | 2.28 | 2.03 | 1.52 | 0 | 1.27 | 0 | 1.52 | 32.7 |
| R03C04 | 0 | 382 | 0 | 382 | Glioblastoma | High | Methylated or partial | Primary | 4.3 | 3.04 | 1.01 | 9.11 | 46.3 | 2.03 | 0.25 | 0.25 | 0.25 | 31.6 | 0 | 1.77 |
| R12C05 | 1 | 101 | 1 | 197 | Glioblastoma | High | Unmethylated | Primary | 12.9 | 14.2 | 2.28 | 31.6 | 1.52 | 5.06 | 0.76 | 2.28 | 1.01 | 0 | 0.76 | 27.6 |
| R08C02 | 1 | 151 | 1 | 493 | Glioblastoma | High | Methylated or partial | Primary | 9.62 | 7.34 | 1.77 | 24.1 | 0.76 | 14.9 | 1.27 | 0.25 | 0.76 | 0 | 1.01 | 38.2 |
| R05C10 | 1 | 168 | 0 | 333 | Glioblastoma | High | Unmethylated | Primary | 18 | 7.85 | 2.53 | 39.7 | 4.3 | 3.8 | 0.76 | 0.76 | 0.25 | 0 | 0 | 22 |
| R10C05 | 0 | 825 | 0 | 825 | Glioblastoma | High | Methylated or partial | Primary | 12.9 | 10.9 | 2.78 | 46.3 | 5.06 | 1.27 | 1.52 | 0.76 | 1.27 | 0.25 | 0.25 | 16.7 |
| R02C06 | 0 | 1138 | 0 | 1138 | Glioblastoma | High | Methylated or partial | Primary | 6.84 | 14.2 | 0.25 | 36.5 | 0.51 | 0.76 | 0.51 | 0 | 1.01 | 0 | 3.8 | 35.7 |
| R03C01 | 1 | 14 | 1 | 14 | Glioblastoma | High | Methylated or partial | Primary | 13.7 | 6.08 | 1.52 | 22 | 51.6 | 0.51 | 0.76 | 0.76 | 0 | 0 | 0.51 | 2.53 |
| R03C10 | 1 | 184 | 1 | 258 | Glioblastoma | High | Unmethylated | Primary | 4.81 | 5.32 | 0.76 | 75.2 | 0.76 | 1.77 | 0 | 0.76 | 0.51 | 0 | 0 | 10.1 |
