# Supplementary material for: DNA damage response profile distinguishes poor-acting gliomas with shared methylome signatures
Source: Neuro Oncol. 2025 Aug 27;28(1):117–29. doi: 10.1093/neuonc/noaf199 (PMC12962623; doi:10.1093/neuonc/noaf199)
Supplement: noaf199_Supplementary_Data [file noaf199_supplementary_data.zip › noaf199_suppl_Supplementary_Tables_S5.docx]

| **Table S5: Software and data resource** | |
| --- | --- |
|  |  |
| **Resource** | **Source** |
| **Software and R libraries** |  |
| R | https://cran.r-project.org/bin/windows/ |
| R Studio | https://posit.co/download/rstudio-desktop/ |
| TCGAbiolinks R library | <https://bioconductor.org/packages/release/bioc/html/TCGAbiolinks.html> |
| ELMER R library | <https://rdrr.io/bioc/ELMER/> |
| Spectrum R library | <https://github.com/crj32/Spectrum/blob/master/R/spectrum.R> |
| ComplexHeatmap R library | <https://bioconductor.org/packages/release/bioc/html/ComplexHeatmap.html> |
| UMAP R library | https://github.com/lmcinnes/umap |
| MCD viewer | <https://www.standardbio.com/products/software> |
| ImageJ | <https://imagej.net/ij/> |
| CellProfiler | <https://cellprofiler.org/> |
| HistoCAT | <https://github.com/BodenmillerGroup/histocat-web> |
| OMIQ | <https://www.omiq.ai/> |
| **Data** |  |
| Annotated DDR gene list | Knijnenburg, et al. 2018 |
| TCGA clinical and molecular data | [https://www.cbioportal.org/ and Ceccarelli M, et al. 2016](https://www.cbioportal.org/%20and%20Ceccarelli%20M,%20et%20al.%202016) |
