# Supplementary material for: DNA damage response profile distinguishes poor-acting gliomas with shared methylome signatures
Source: Neuro Oncol. 2025 Aug 27;28(1):117–29. doi: 10.1093/neuonc/noaf199 (PMC12962623; doi:10.1093/neuonc/noaf199)
Supplement: noaf199_Supplementary_Data [file noaf199_supplementary_data.zip › noaf199_suppl_Supplementary_Figures_S1-S12.docx]

**Supplementary Figures**


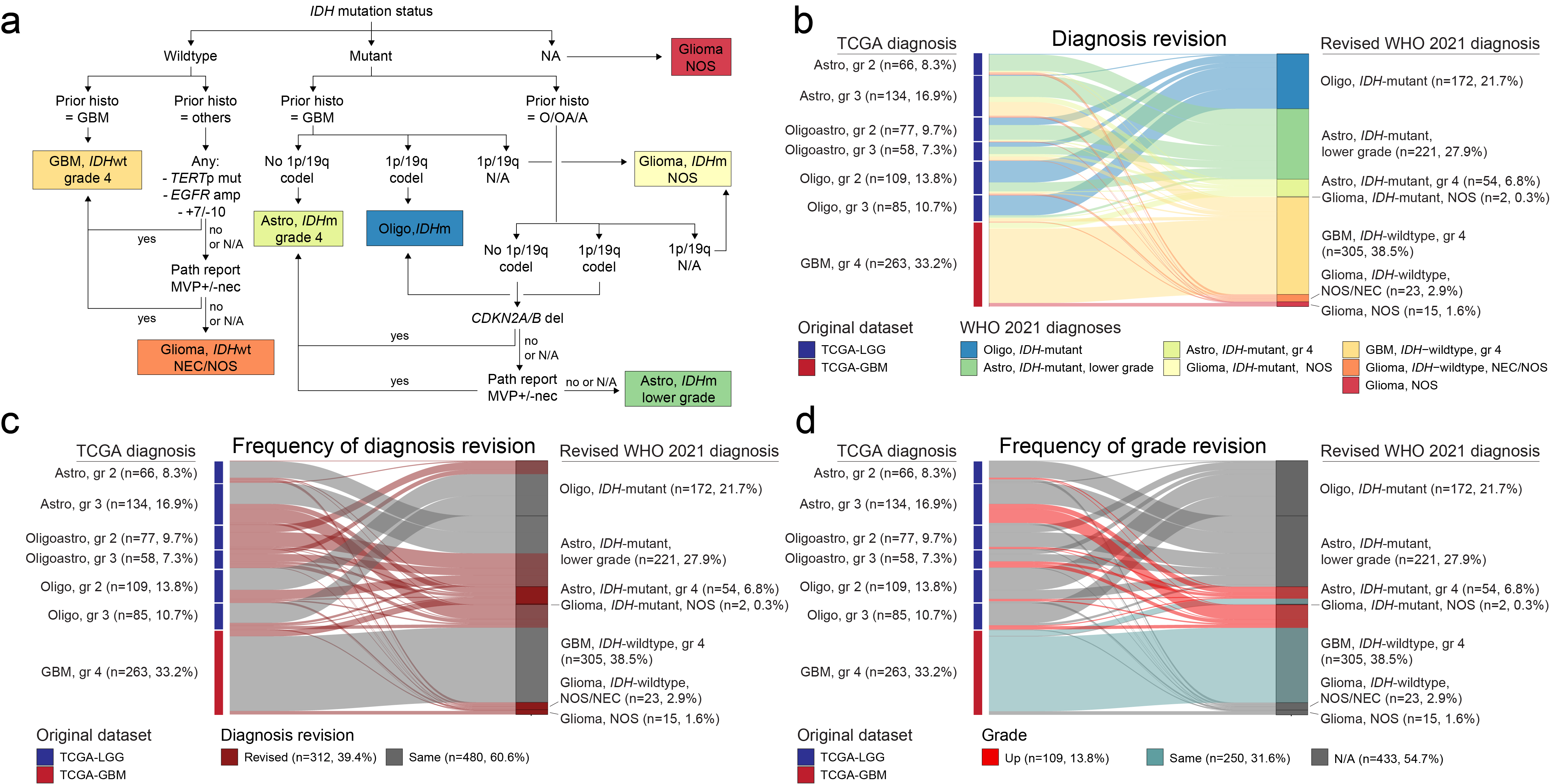


**Figure S1: Reclassification schematic for diffuse gliomas from TCGA-GBM and TCGA-LGG datasets.** (a) Algorithm for revising the diagnosis of TCGA gliomas with integration of molecular and histologic criteria according to WHO 2021 guidelines. Riverplots demonstrate (b) the original TCGA diagnoses (left; TCGA-GBM red, TCGA-LGG blue) and the revised WHO 2021 diagnoses (right) of 792 TCGA gliomas. (c) The frequency of diagnosis revision and (d) change in diagnosis grades are shown.


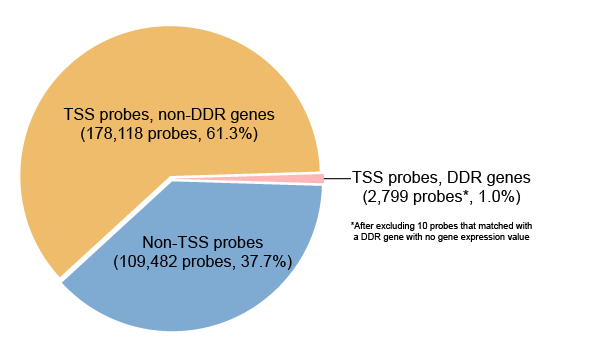


**Figure S2: Selection of probes at the DDR gene transcription start sties.** A total of 2,799 DDR TSS probes were identified from the initial 287,600 methylation probes.


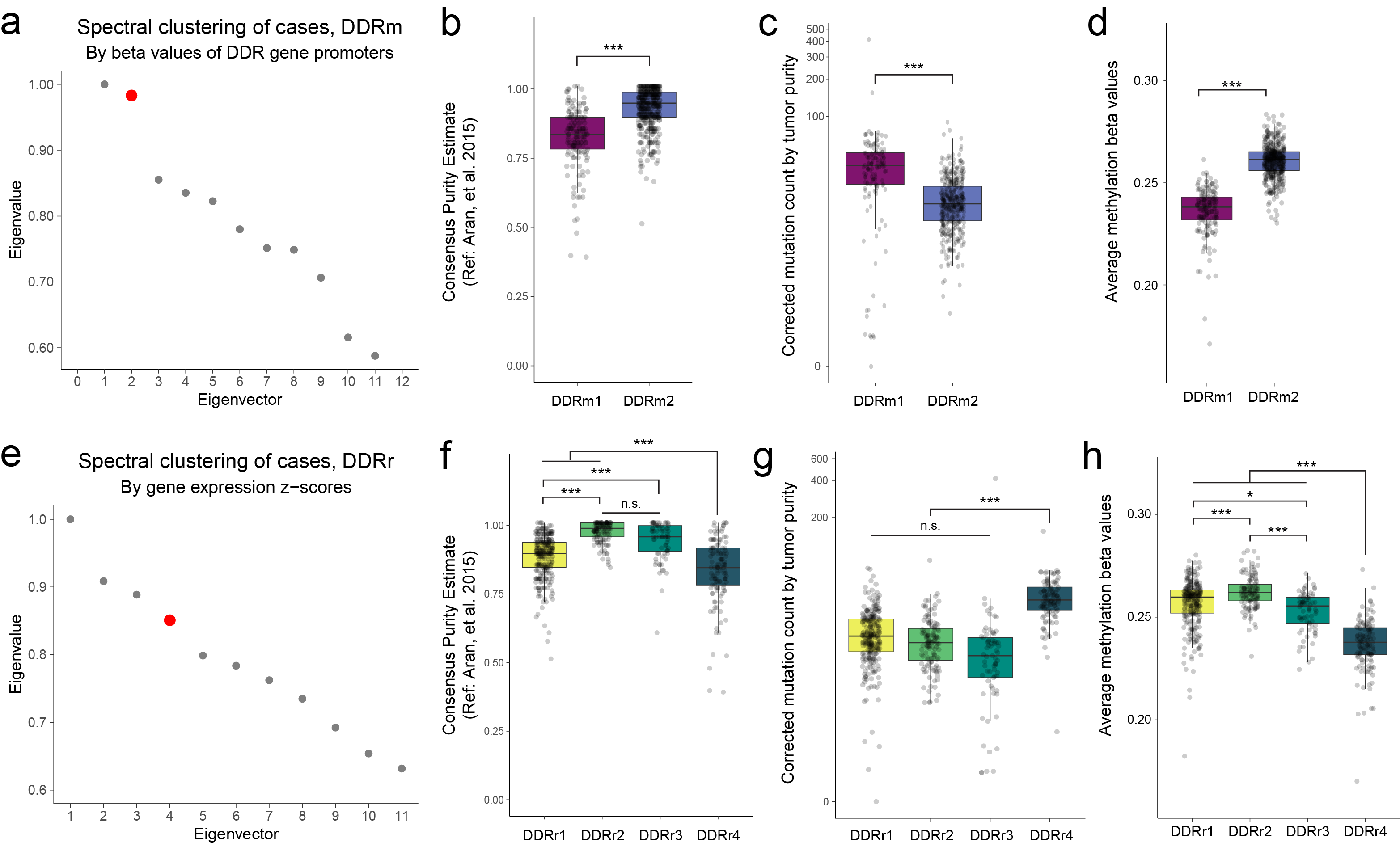


**Figure S3: Methylation and gene expression clusters by DDR genes demonstrate distinct methylation characteristics and tumor mutation burden.** Spectral clustering determined the optimal number of tumor clusters defined by either (a) the methylation beta values of probes at DDR gene promoters (DDRm) or (e) gene expression z-scores (DDRr). The consensus tumor purity estimate (previously reported) for each DDRm or DDRr cluster is depicted in (b) and (f), respectively. The corrected mutation burden based on the consensus tumor purity estimate, by DDRm (c) and DDRr (g), are shown. The average methylation beta-values at DDR promoters are compared between the different (d) DDRm clusters and (h) DDRr clusters. A two-tailed t-test was used in panels (b), (c), and (d). For (f), (g), and (h), one-way ANOVA was performed, followed by a pairwise comparison. * p-value < 0.01, *** p-value < 0.001, and n.s. = not significant.


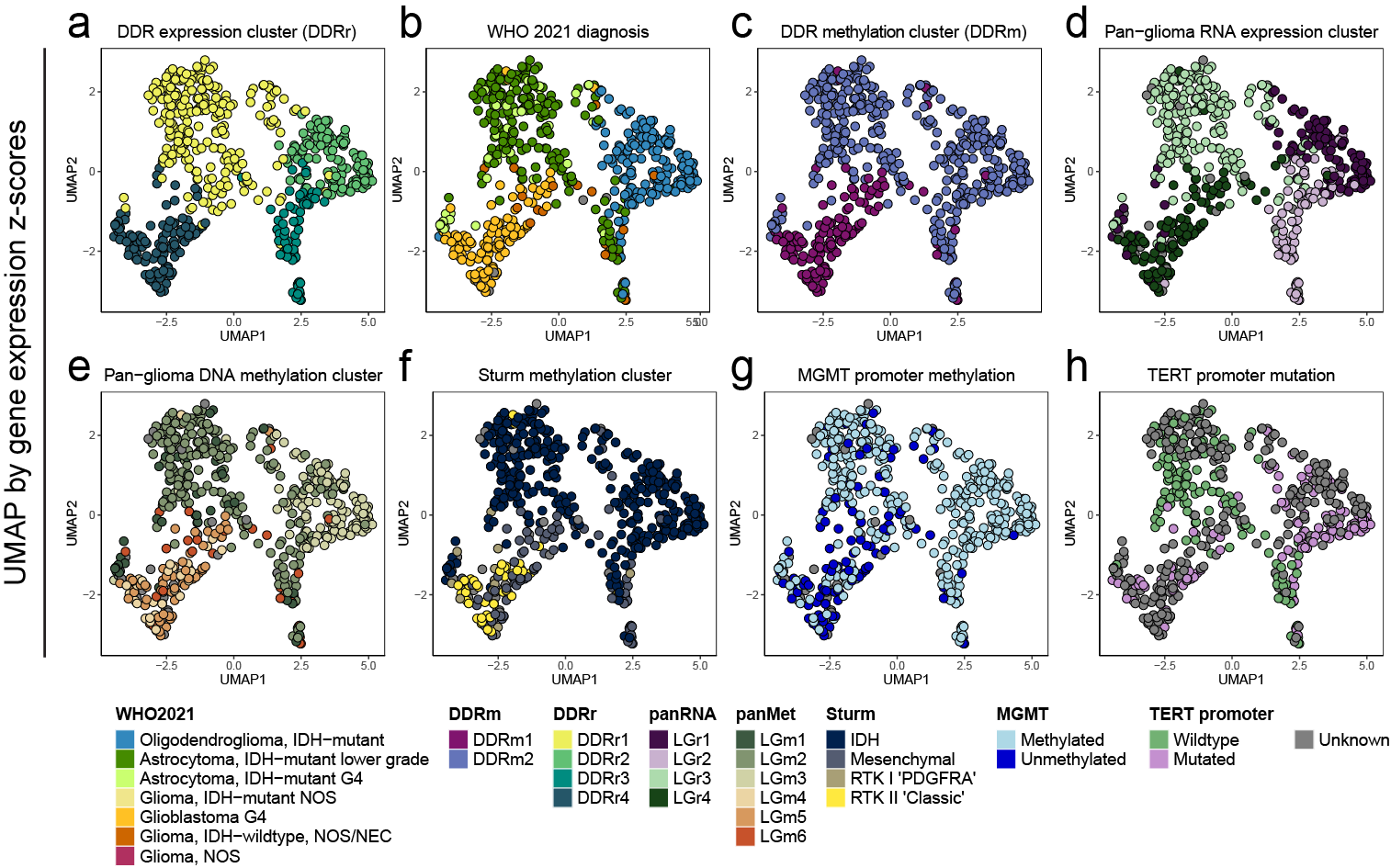


**Figure S4: DDR gene RNA expression status correlates with WHO diagnosis classification, and molecular signatures including previously reported molecular classes.** (a) UMAP analysis by normalized transcriptional expression levels of DDR gene shows the gene expression clusters (DDRr) clusters identified by unsupervised spectral clustering, and (b) the corresponding updated tumor classification in accordance with the WHO criteria and (c) the DDR methylation clusters (DDRm). UMAP is overlaid by (d-f) the previously reports molecular classes, (g) *MGMT* promoter methylation status, and (h)*TERT* promoter mutation status.


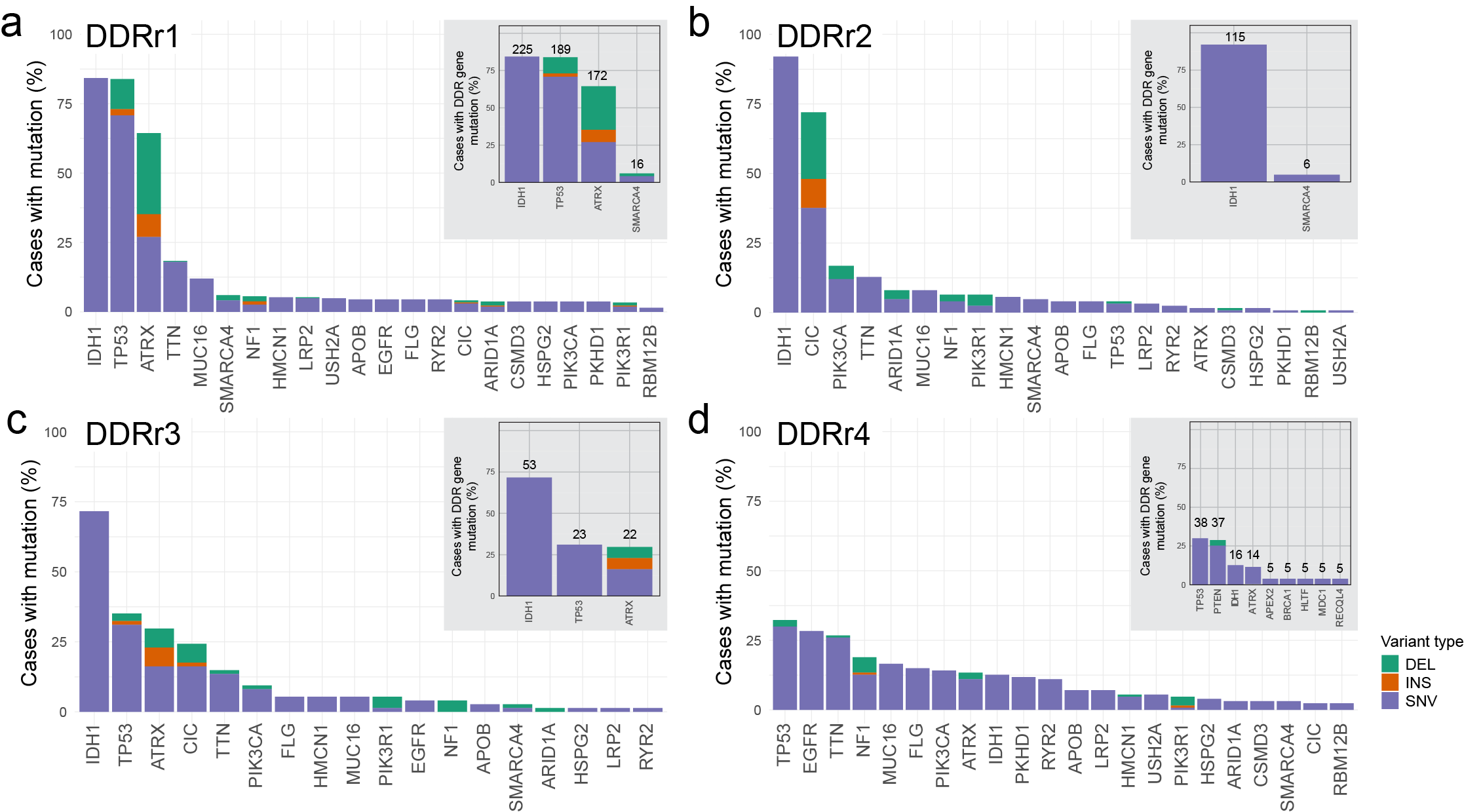


**Figure S5: Commonly mutated genes in each DDRr cluster reflect the frequency of molecular defined tumor types.** The most commonly mutated genes (x-axes) in (a) DDRr1, (b) DDRr2, (c) DDRr3, and (d) DDRr4 are shown. Y-axes represent frequencies of cases with a certain mutated gene in each DDRr shown as percentages. The gray insets illustrate the frequencies of DDR gene mutations in each DDRr cluster. DEL = deletions (green), INS = insertions (orange), SNV = single nucleotide variants (blue)


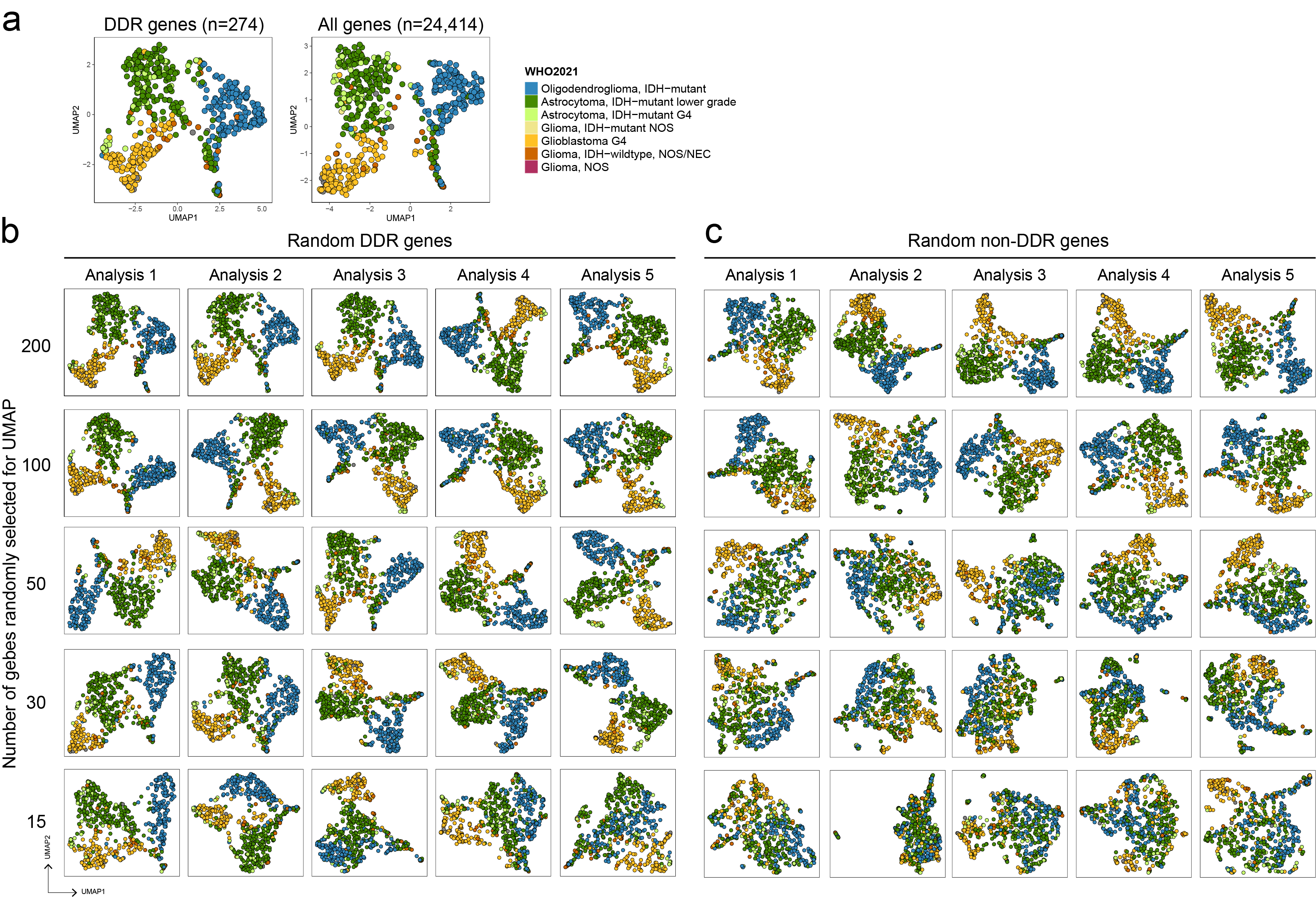


**Figure S6: DDR genes are sufficient and crucial for glioma classification.** (a) UMAP analyses of gene expression z-scores of 274 DDR genes (left) vs all 24,414 genes (right) are depicted. Random numbers (200, 100, 50, 30, and 15) of either (b) DDR genes or (c) non-DDR genes were selected for repeated UMAP analyses (5 analyses each).


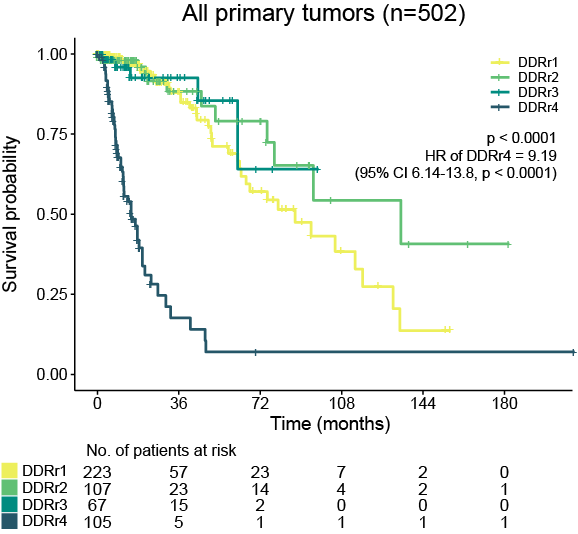


**Figure S7: Survival probability by DDRr clusters.** Kaplan-Meier plot demonstrates the survival probability of all primary TCGA tumors compares the overall survival probability between different DDRr groups. P-value was derived from log-ranked tests. Hazard ratios (HR) with 95% confidence intervals (CI) are shown.


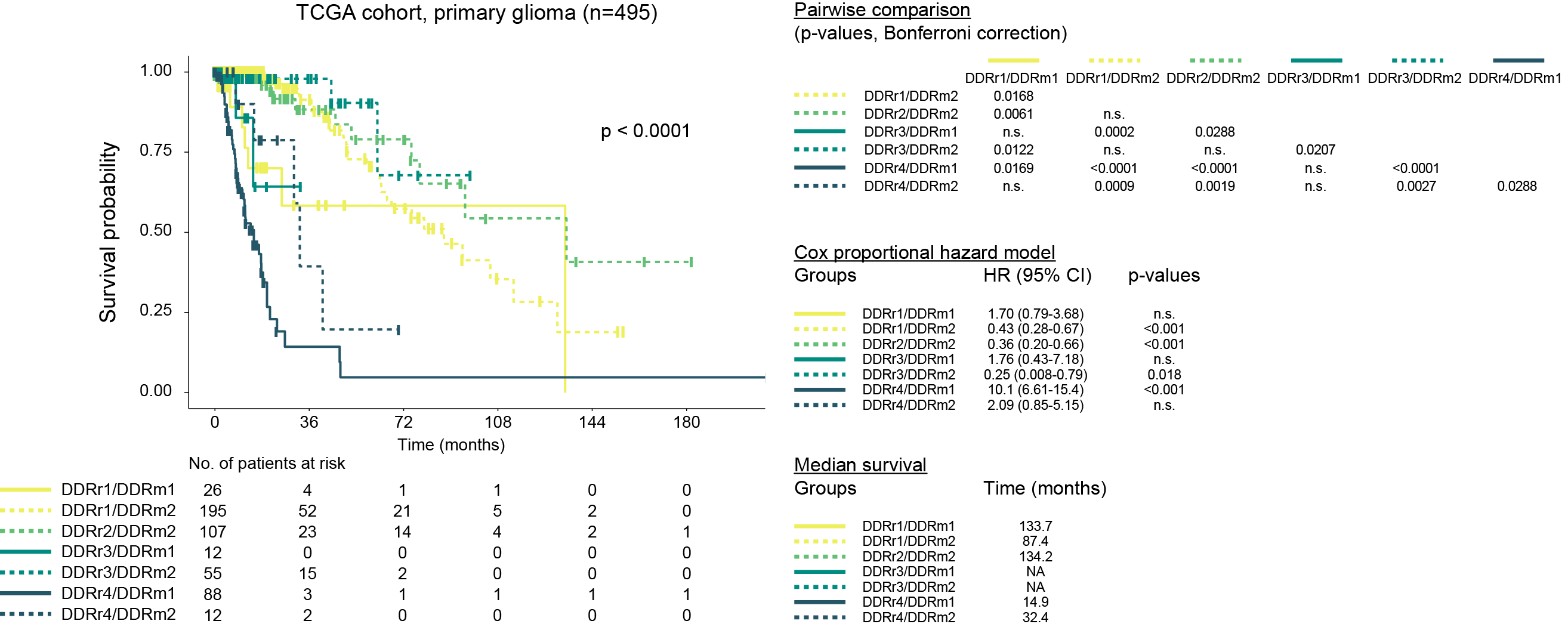


**Figure S8: Hypomethylated DDR gene promoters portend a worse survival probability beyond DDR gene expression.** Kaplan-Meier plot of the survival probability of TCGA gliomas grouped by their combined DDRr and DDRm clusters. P-values of the KM plot were derived from a log-ranked test. Hazard ratios (HR) with 95% confidence intervals (CI), and the median survival (months) for each subgroup are shown. Of note, all DDRr2 gliomas were classified as DDRm2.


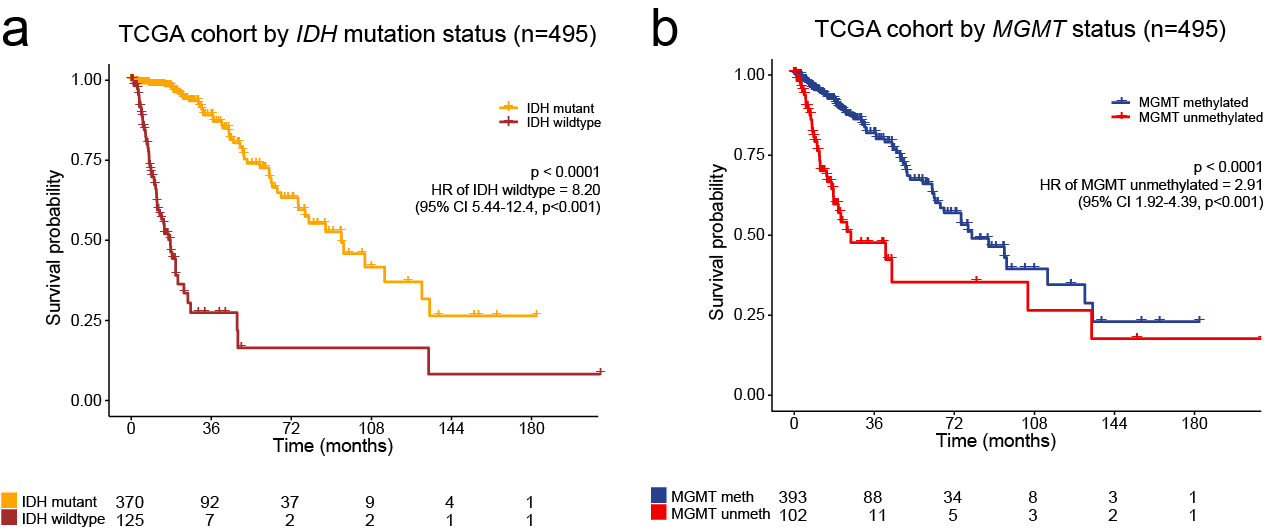


**Figure S9: Overall survival of the TCGA cohort.** Kaplan-Meier plots illustrate the overall survival of the TCGA cases used in this study, stratified either by (a) *IDH* mutation status, or (b) *MGMT* promoter methylation status. P-values were derived from log-ranked tests. Hazard ratios (HR) with 95% confidence intervals (CI) are shown.


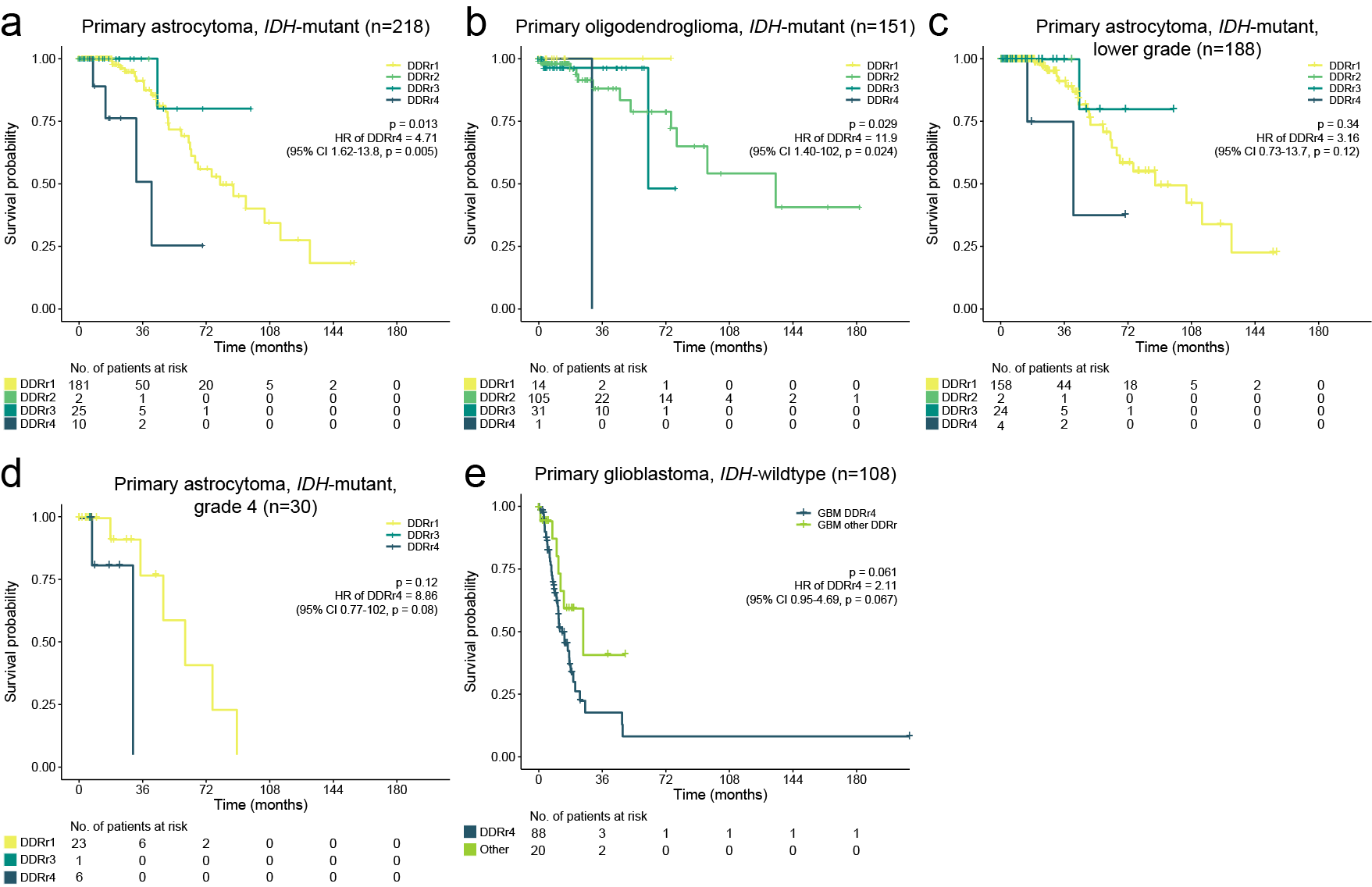


**Figure S10: DDRr4 cluster is associated with poor survival independent of the molecular-defined diagnosis.** Primary diffuse gliomas were separated by their molecular-defined diagnoses and grades, and separate survival analyses were performed. Kaplan-Meier (KM) survival analysis of (a) all astrocytoma, (b) oligodendrogliomas, (c) lower grade astrocytoma, (d) grade 4 astrocytoma, and (e) glioblastoma are shown. P-values plots were derived from log-ranked tests. Hazard ratios (HR) with 95% confidence intervals (CI) are shown. Of note, none of the grade 4 astrocytoma were classified as DDRr2.


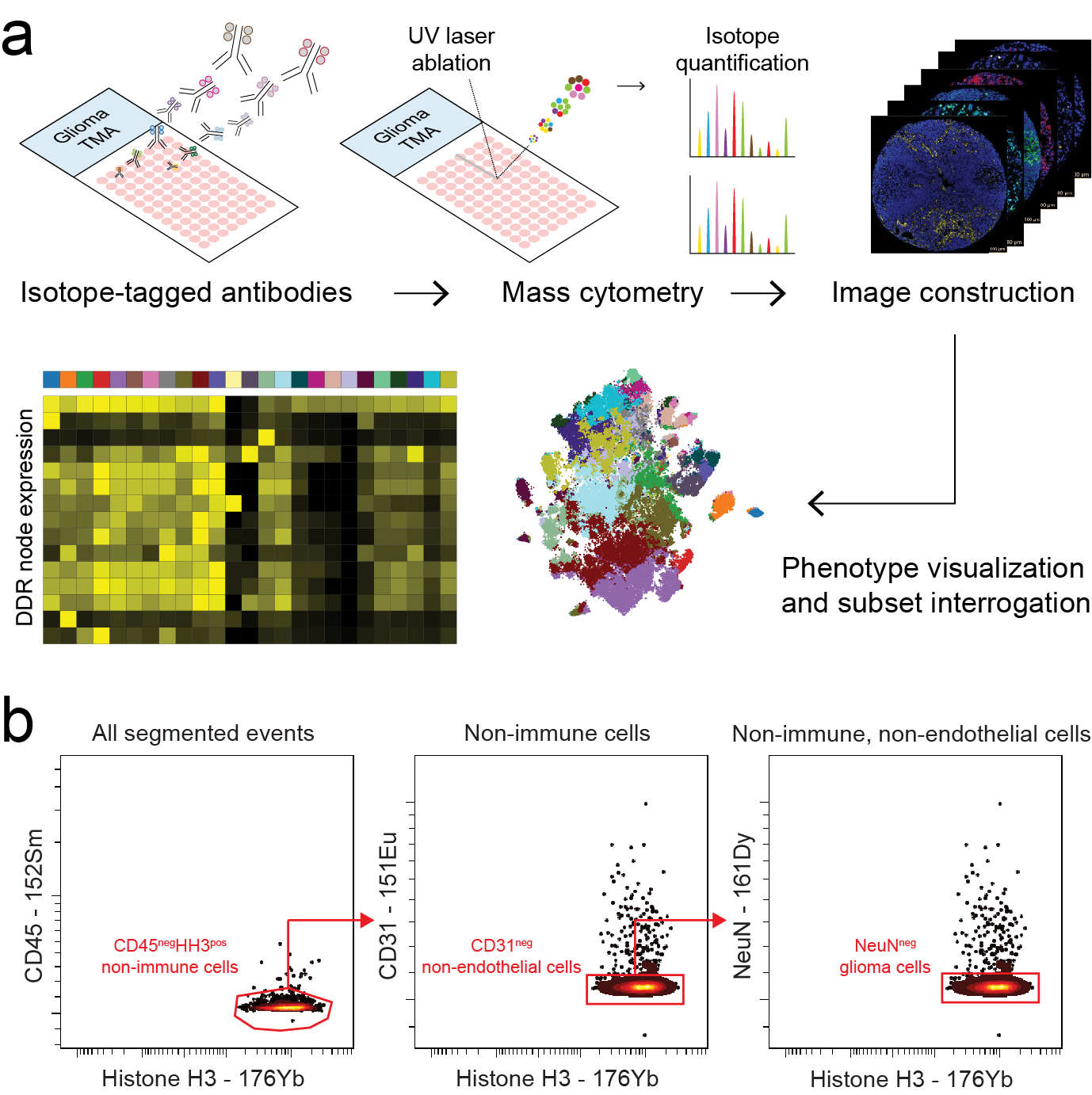


**Figure S11: Analysis workflow by IMC and identification of glioma cells.** (a) Experimental and analysis workflow for protein-level quantitation of DDR effector expression using tissue microarray and imaging mass cytometry. (b) Single cell gating strategy to obtain CD45^neg^ CD31^neg^ NeuN^neg^ glioma tumor cells for subsequent analyses.


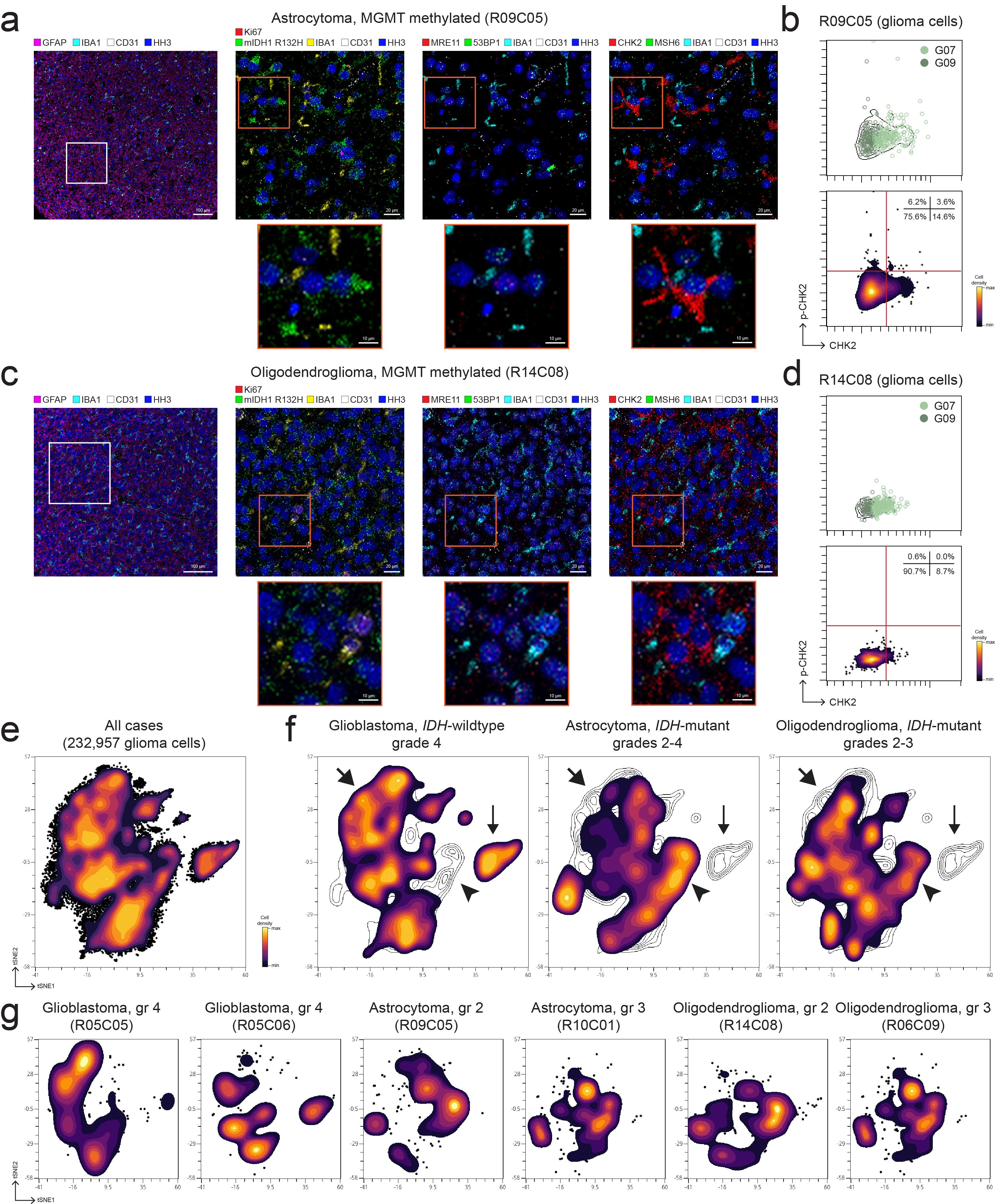


**Figure S12:** **Intertumoral and intratumoral diversity of DDR effector protein expression in single tumor cells.** Representative images of (a) an astrocytoma (R09C05) and (c) an oligodendroglioma (R14C08), obtained by imaging mass cytometry, illustrating the expression of DDR and lineage-defined markers. Low (left) and high (right panels) magnification images are shown. Two-dimensional plots comparing total CHK2 (x-axes) and phosphorylated CHK2 (p-CHK2, Thr68 residue) in (b) astrocytoma R09C05 and (d) oligodendroglioma R14C08 are shown. (e) Cell density t-SNE plot illustrates the distribution of all glioma tumor cells (232,957 cells). (f) The distributions of tumor cells based on tumor diagnoses are also shown (glioblastoma, left; astrocytoma, middle; oligodendroglioma, right). The underlying contour plots represent the distribution of all glioma cells in the analysis, same as panel (e). Small and large arrows highlight proliferative (Ki67+) and non-proliferative (Ki67-) cells that were enriched in GBM, respectively (see Fig 4b). Arrowheads indicate cells that were enriched in astrocytoma and oligodendroglioma. (g) Cell density plots of representative individual tumors are shown (two of each: glioblastoma, left; astrocytoma, middle; oligodendroglioma, right).
