# Supplementary material for: DNA damage response profile distinguishes poor-acting gliomas with shared methylome signatures
Source: Neuro Oncol. 2025 Aug 27;28(1):117–29. doi: 10.1093/neuonc/noaf199 (PMC12962623; doi:10.1093/neuonc/noaf199)
Supplement: noaf199_Supplementary_Data [file noaf199_supplementary_data.zip › noaf199_suppl_Supplementary_Tables_S1.docx]

| **Table S1: Demographics, molecular characteristics, and diagnosis of TCGA cases** | | | | | |  |  |  |  |  |  |  |  |  |  |  |  |  |  |  |  |
| --- | --- | --- | --- | --- | --- | --- | --- | --- | --- | --- | --- | --- | --- | --- | --- | --- | --- | --- | --- | --- | --- |
|  |  |  |  |  |  |  |  |  |  |  |  |  |  |  |  |  |  |  |  |  |  |
| **Sample ID** | **Patient ID** | **Diagnosis age (years)** | **Overall Survival Status** | **Sex** | **Status** | **Oroginal TCGA Diagnosis** | **Revised WHO 2021 Diagnosis** | **Criteria used for upgrade** | **Notes** | **DDRm** | **DDRr** | **Chr.7 gain/Chr.10 loss** | **IDH-1P19Q Subtype** | **IDH status** | **MGMT Promoter Ptatus** | **TERT Promoter Status** | **Mutation Count** | **Original Subtype** | **Pan Glioma DNA Methylation Cluster** | **Pan Glioma RNA Expression Cluster** | **Supervised DNA Methylation Cluster** |
| TCGA-02-0047-01 | TCGA-02-0047 | 78 | 1:DECEASED | Male | Primary | Glioblastoma G4 | Glioblastoma G4 |  |  | NA | NA | No combined CNA | Non-codel | WT | Unmethylated | NA | 46 | Proneural | LGm6 | LGr3 | LGm6-GBM |
| TCGA-02-0055-01 | TCGA-02-0055 | 62 | 1:DECEASED | Female | Primary | Glioblastoma G4 | Glioblastoma G4 |  |  | NA | NA | No combined CNA | Non-codel | WT | Unmethylated | NA | 42 | Mesenchymal | LGm5 | LGr4 | Mesenchymal-like |
| TCGA-02-2483-01 | TCGA-02-2483 | 43 | 0:LIVING | Male | Primary | Glioblastoma G4 | Astrocytoma, IDH-mutant G4 |  |  | NA | NA | No combined CNA | Non-codel | Mutant | Methylated | WT | 24 | G-CIMP | LGm1 | LGr3 | G-CIMP-low |
| TCGA-02-2485-01 | TCGA-02-2485 | 53 | 0:LIVING | Male | Primary | Glioblastoma G4 | Glioblastoma G4 |  |  | NA | NA | Gain chr 7 & loss chr 10 | Non-codel | WT | Unmethylated | Mutant | 44 | Classical | LGm4 | LGr4 | Classic-like |
| TCGA-02-2486-01 | TCGA-02-2486 | 64 | 0:LIVING | Male | Primary | Glioblastoma G4 | Glioblastoma G4 |  |  | NA | NA | Gain chr 7 & loss chr 10 | Non-codel | WT | Unmethylated | NA | 47 | Mesenchymal | LGm4 | LGr4 | Classic-like |
| TCGA-06-0125-01A | TCGA-06-0125 | 63 | 1:DECEASED | Female | Primary | Glioblastoma G4 | Glioblastoma G4 |  |  | DDRm1 | DDRr4 | No combined CNA | Non-codel | WT | Methylated | Mutant | 55 | Classical | LGm4 | LGr4 | Classic-like |
| TCGA-06-0125-02A | TCGA-06-0125 | NA | NA | NA | Recurrent | Glioblastoma G4 | Glioblastoma G4 |  |  | DDRm1 | DDRr4 | NA | NA | NA | NA | NA | NA | NA | NA | NA | NA |
| TCGA-06-0129-01 | TCGA-06-0129 | 30 | 1:DECEASED | Male | Primary | Glioblastoma G4 | Astrocytoma, IDH-mutant G4 |  |  | NA | NA | No combined CNA | Non-codel | Mutant | Methylated | NA | 33 | G-CIMP | LGm1 | LGr3 | G-CIMP-low |
| TCGA-06-0130-01 | TCGA-06-0130 | 54 | 1:DECEASED | Male | Primary | Glioblastoma G4 | Glioblastoma G4 |  |  | NA | NA | No combined CNA | Non-codel | WT | Unmethylated | NA | 21 | Mesenchymal | LGm5 | LGr4 | Mesenchymal-like |
| TCGA-06-0132-01 | TCGA-06-0132 | 49 | 1:DECEASED | Male | Primary | Glioblastoma G4 | Glioblastoma G4 |  |  | NA | NA | No combined CNA | Non-codel | WT | NA | NA | 15 | Neural | NA | LGr2 | NA |
| TCGA-06-0138-01 | TCGA-06-0138 | 43 | 1:DECEASED | Male | Primary | Glioblastoma G4 | Glioblastoma G4 |  |  | NA | NA | Gain chr 7 & loss chr 10 | Non-codel | WT | NA | NA | NA | Neural | NA | LGr4 | NA |
| TCGA-06-0139-01 | TCGA-06-0139 | 40 | 1:DECEASED | Male | Primary | Glioblastoma G4 | Glioblastoma G4 |  |  | NA | NA | No combined CNA | Non-codel | WT | Unmethylated | NA | 1 | Mesenchymal | LGm6 | LGr4 | LGm6-GBM |
| TCGA-06-0141-01 | TCGA-06-0141 | 62 | 1:DECEASED | Male | Primary | Glioblastoma G4 | Glioblastoma G4 |  |  | NA | NA | No combined CNA | Non-codel | WT | Unmethylated | NA | 5 | Mesenchymal | LGm6 | LGr4 | LGm6-GBM |
| TCGA-06-0152-01A | TCGA-06-0152 | 68 | 1:DECEASED | Male | Primary | Glioblastoma G4 | Glioblastoma G4 |  |  | NA | NA | Gain chr 7 & loss chr 10 | Non-codel | WT | NA | NA | 47 | Mesenchymal | NA | LGr4 | NA |
| TCGA-06-0152-02A | TCGA-06-0152 | NA | NA | NA | Recurrent | Glioblastoma G4 | Glioblastoma G4 |  |  | DDRm1 | DDRr4 | NA | NA | NA | NA | NA | NA | NA | NA | NA | NA |
| TCGA-06-0156-01 | TCGA-06-0156 | 57 | 1:DECEASED | Male | Primary | Glioblastoma G4 | Glioma, IDH-mutant NOS |  |  | NA | NA | NA | NA | Mutant | NA | NA | NA | Proneural | NA | LGr4 | NA |
| TCGA-06-0157-01 | TCGA-06-0157 | 63 | 1:DECEASED | Female | Primary | Glioblastoma G4 | Glioblastoma G4 |  |  | NA | NA | Gain chr 7 & loss chr 10 | Non-codel | WT | NA | Mutant | 41 | Classical | NA | LGr4 | NA |
| TCGA-06-0158-01 | TCGA-06-0158 | 73 | 1:DECEASED | Male | Primary | Glioblastoma G4 | Glioblastoma G4 |  |  | NA | NA | No combined CNA | Non-codel | WT | NA | NA | 38 | Classical | NA | LGr4 | NA |
| TCGA-06-0168-01 | TCGA-06-0168 | 59 | 1:DECEASED | Female | Primary | Glioblastoma G4 | Glioblastoma G4 |  |  | NA | NA | Gain chr 7 & loss chr 10 | Non-codel | WT | NA | NA | 34 | Mesenchymal | NA | LGr4 | NA |
| TCGA-06-0171-01A | TCGA-06-0171 | 65 | 1:DECEASED | Male | Primary | Glioblastoma G4 | Glioblastoma G4 |  |  | NA | NA | Gain chr 7 & loss chr 10 | Non-codel | WT | NA | Mutant | 37 | Neural | NA | LGr4 | NA |
| TCGA-06-0171-02A | TCGA-06-0171 | NA | NA | NA | Recurrent | Glioblastoma G4 | Glioblastoma G4 |  |  | DDRm1 | DDRr4 | NA | NA | NA | NA | NA | NA | NA | NA | NA | NA |
| TCGA-06-0174-01 | TCGA-06-0174 | 54 | 1:DECEASED | Male | Primary | Glioblastoma G4 | Glioblastoma G4 |  |  | NA | NA | Gain chr 7 & loss chr 10 | Non-codel | WT | NA | NA | 53 | Proneural | NA | LGr1 | NA |
| TCGA-06-0178-01 | TCGA-06-0178 | 38 | 0:LIVING | Male | Primary | Glioblastoma G4 | Astrocytoma, IDH-mutant G4 |  |  | NA | NA | No combined CNA | Non-codel | Mutant | NA | NA | NA | Neural | NA | LGr4 | NA |
| TCGA-06-0184-01 | TCGA-06-0184 | 63 | 0:LIVING | Male | Primary | Glioblastoma G4 | Glioblastoma G4 |  |  | NA | NA | Gain chr 7 & loss chr 10 | Non-codel | WT | NA | NA | 39 | Mesenchymal | NA | LGr4 | NA |
| TCGA-06-0187-01 | TCGA-06-0187 | 69 | 1:DECEASED | Male | Primary | Glioblastoma G4 | Glioblastoma G4 |  |  | NA | NA | No combined CNA | Non-codel | WT | NA | NA | NA | Classical | NA | LGr4 | NA |
| TCGA-06-0190-01A | TCGA-06-0190 | 62 | 1:DECEASED | Male | Primary | Glioblastoma G4 | Glioblastoma G4 |  |  | DDRm1 | DDRr4 | Gain chr 7 & loss chr 10 | Non-codel | WT | NA | Mutant | 44 | Mesenchymal | NA | LGr4 | NA |
| TCGA-06-0190-02A | TCGA-06-0190 | NA | NA | NA | Recurrent | Glioblastoma G4 | Glioblastoma G4 |  |  | DDRm1 | DDRr4 | NA | NA | NA | NA | NA | NA | NA | NA | NA | NA |
| TCGA-06-0210-01A | TCGA-06-0210 | 72 | 1:DECEASED | Female | Primary | Glioblastoma G4 | Glioblastoma G4 |  |  | DDRm1 | DDRr4 | Gain chr 7 & loss chr 10 | Non-codel | WT | NA | Mutant | 41 | Mesenchymal | NA | LGr4 | NA |
| TCGA-06-0210-02A | TCGA-06-0210 | NA | NA | NA | Recurrent | Glioblastoma G4 | Glioblastoma G4 |  |  | DDRm1 | DDRr4 | NA | NA | NA | NA | NA | NA | NA | NA | NA | NA |
| TCGA-06-0211-01A | TCGA-06-0211 | 47 | 1:DECEASED | Male | Primary | Glioblastoma G4 | Glioblastoma G4 |  |  | DDRm1 | DDRr4 | Gain chr 7 & loss chr 10 | Non-codel | WT | NA | Mutant | 46 | Classical | NA | LGr4 | NA |
| TCGA-06-0211-02A | TCGA-06-0211 | NA | NA | NA | Recurrent | Glioblastoma G4 | Glioblastoma G4 |  |  | DDRm1 | DDRr4 | NA | NA | NA | NA | NA | NA | NA | NA | NA | NA |
| TCGA-06-0219-01 | TCGA-06-0219 | 67 | 1:DECEASED | Male | Primary | Glioblastoma G4 | Glioblastoma G4 |  |  | NA | NA | No combined CNA | Non-codel | WT | NA | NA | 35 | Neural | NA | LGr4 | NA |
| TCGA-06-0221-01A | TCGA-06-0221 | 31 | 1:DECEASED | Male | Primary | Glioblastoma G4 | Astrocytoma, IDH-mutant G4 |  |  | NA | NA | No combined CNA | Non-codel | Mutant | NA | WT | 16 | Neural | NA | unclassified | NA |
| TCGA-06-0221-02A | TCGA-06-0221 | NA | NA | NA | Recurrent | Glioblastoma G4 | Astrocytoma, IDH-mutant G4 |  |  | DDRm2 | DDRr4 | NA | NA | NA | NA | NA | NA | NA | NA | NA | NA |
| TCGA-06-0238-01 | TCGA-06-0238 | 46 | 1:DECEASED | Male | Primary | Glioblastoma G4 | Glioblastoma G4 |  |  | NA | NA | Gain chr 7 & loss chr 10 | Non-codel | WT | NA | NA | 31 | Proneural | NA | LGr1 | NA |
| TCGA-06-0644-01 | TCGA-06-0644 | 71 | 0:LIVING | Male | Primary | Glioblastoma G4 | Glioblastoma G4 |  |  | NA | NA | Gain chr 7 & loss chr 10 | Non-codel | WT | NA | NA | 43 | Mesenchymal | NA | LGr4 | NA |
| TCGA-06-0645-01 | TCGA-06-0645 | 55 | 1:DECEASED | Female | Primary | Glioblastoma G4 | Glioblastoma G4 |  |  | NA | NA | Gain chr 7 & loss chr 10 | Non-codel | WT | NA | NA | 41 | Mesenchymal | NA | LGr4 | NA |
| TCGA-06-0646-01 | TCGA-06-0646 | 60 | 1:DECEASED | Male | Primary | Glioblastoma G4 | Glioblastoma G4 |  |  | NA | NA | Gain chr 7 & loss chr 10 | Non-codel | WT | NA | NA | 30 | Proneural | NA | LGr4 | NA |
| TCGA-06-0649-01 | TCGA-06-0649 | 73 | 1:DECEASED | Female | Primary | Glioblastoma G4 | Glioblastoma G4 |  |  | NA | NA | Gain chr 7 & loss chr 10 | Non-codel | WT | NA | NA | 65 | Neural | NA | LGr4 | NA |
| TCGA-06-0650-01A | TCGA-06-0650 | 39 | 1:DECEASED | Female | Primary | Glioblastoma G4 | Glioblastoma G4 |  |  | NA | NA | No combined CNA | Non-codel | WT | Unmethylated | NA | 14 | Mesenchymal | LGm6 | LGr4 | LGm6-GBM |
| TCGA-06-0686-01 | TCGA-06-0686 | 53 | 0:LIVING | Male | Primary | Glioblastoma G4 | Glioblastoma G4 |  |  | NA | NA | Gain chr 7 & loss chr 10 | Non-codel | WT | NA | Mutant | 41 | Proneural | NA | LGr1 | NA |
| TCGA-06-0743-01 | TCGA-06-0743 | 69 | 0:LIVING | Male | Primary | Glioblastoma G4 | Glioblastoma G4 |  |  | NA | NA | Gain chr 7 & loss chr 10 | Non-codel | WT | NA | NA | 71 | Classical | NA | LGr4 | NA |
| TCGA-06-0744-01 | TCGA-06-0744 | 66 | 0:LIVING | Male | Primary | Glioblastoma G4 | Glioblastoma G4 |  |  | NA | NA | Gain chr 7 & loss chr 10 | Non-codel | WT | NA | Mutant | 53 | Classical | NA | LGr4 | NA |
| TCGA-06-0745-01 | TCGA-06-0745 | 59 | 1:DECEASED | Male | Primary | Glioblastoma G4 | Glioblastoma G4 |  |  | NA | NA | Gain chr 7 & loss chr 10 | Non-codel | WT | NA | Mutant | 40 | Proneural | NA | LGr4 | NA |
| TCGA-06-0747-01 | TCGA-06-0747 | 53 | 1:DECEASED | Male | Primary | Glioblastoma G4 | Glioblastoma G4 |  |  | NA | NA | No combined CNA | Non-codel | WT | NA | NA | 62 | Classical | NA | LGr4 | NA |
| TCGA-06-0749-01 | TCGA-06-0749 | 50 | 1:DECEASED | Male | Primary | Glioblastoma G4 | Glioblastoma G4 |  |  | NA | NA | Gain chr 7 & loss chr 10 | Non-codel | WT | NA | NA | 36 | Neural | NA | LGr4 | NA |
| TCGA-06-0750-01 | TCGA-06-0750 | 43 | 1:DECEASED | Male | Primary | Glioblastoma G4 | Glioblastoma G4 |  |  | NA | NA | Gain chr 7 & loss chr 10 | Non-codel | WT | NA | NA | 28 | Mesenchymal | NA | LGr4 | NA |
| TCGA-06-0878-01 | TCGA-06-0878 | 74 | 0:LIVING | Male | Primary | Glioblastoma G4 | Glioblastoma G4 |  |  | NA | NA | Gain chr 7 & loss chr 10 | Non-codel | WT | Unmethylated | NA | 26 | Mesenchymal | LGm5 | LGr4 | Mesenchymal-like |
| TCGA-06-0882-01 | TCGA-06-0882 | 30 | 0:LIVING | Male | Primary | Glioblastoma G4 | Glioblastoma G4 |  |  | NA | NA | Gain chr 7 & loss chr 10 | Non-codel | WT | Unmethylated | NA | 24 | Neural | LGm6 | LGr4 | LGm6-GBM |
| TCGA-06-1804-01A | TCGA-06-1804 | 81 | 1:DECEASED | Female | Primary | Glioblastoma G4 | Glioblastoma G4 |  |  | DDRm1 | DDRr4 | Gain chr 7 & loss chr 10 | Non-codel | WT | Methylated | NA | 60 | Classical | LGm4 | LGr4 | Classic-like |
| TCGA-06-1806-01A | TCGA-06-1806 | 47 | 1:DECEASED | Male | Primary | Glioblastoma G4 | Glioblastoma G4 |  |  | NA | NA | No combined CNA | Non-codel | WT | Unmethylated | NA | 16 | NA | LGm6 | NA | LGm6-GBM |
| TCGA-06-2557-01 | TCGA-06-2557 | 76 | 1:DECEASED | Male | Primary | Glioblastoma G4 | Glioblastoma G4 |  |  | NA | NA | No combined CNA | Non-codel | WT | Unmethylated | Mutant | 33 | Mesenchymal | LGm4 | LGr4 | Classic-like |
| TCGA-06-2558-01 | TCGA-06-2558 | 75 | 1:DECEASED | Female | Primary | Glioblastoma G4 | Glioblastoma G4 |  |  | NA | NA | Gain chr 7 & loss chr 10 | Non-codel | WT | Unmethylated | NA | 54 | Proneural | LGm5 | LGr1 | Mesenchymal-like |
| TCGA-06-2559-01 | TCGA-06-2559 | 83 | 1:DECEASED | Male | Primary | Glioblastoma G4 | Glioblastoma G4 |  |  | NA | NA | No combined CNA | Non-codel | WT | Methylated | NA | 40 | Proneural | LGm5 | LGr1 | Mesenchymal-like |
| TCGA-06-2561-01 | TCGA-06-2561 | 53 | 0:LIVING | Female | Primary | Glioblastoma G4 | Glioblastoma G4 |  |  | NA | NA | Gain chr 7 & loss chr 10 | Non-codel | WT | Unmethylated | NA | 34 | Mesenchymal | LGm5 | LGr4 | Mesenchymal-like |
| TCGA-06-2562-01 | TCGA-06-2562 | 81 | 0:LIVING | Male | Primary | Glioblastoma G4 | Glioblastoma G4 |  |  | NA | NA | Gain chr 7 & loss chr 10 | Non-codel | WT | Unmethylated | NA | 47 | Mesenchymal | LGm4 | LGr4 | Classic-like |
| TCGA-06-2563-01 | TCGA-06-2563 | 72 | 0:LIVING | Female | Primary | Glioblastoma G4 | Glioblastoma G4 |  |  | NA | NA | Gain chr 7 & loss chr 10 | Non-codel | WT | Methylated | NA | 59 | Classical | LGm4 | LGr4 | Classic-like |
| TCGA-06-2564-01 | TCGA-06-2564 | 50 | 0:LIVING | Male | Primary | Glioblastoma G4 | Glioblastoma G4 |  |  | NA | NA | Gain chr 7 & loss chr 10 | Non-codel | WT | Unmethylated | NA | 51 | Classical | LGm5 | LGr4 | Mesenchymal-like |
| TCGA-06-2565-01 | TCGA-06-2565 | 59 | 0:LIVING | Male | Primary | Glioblastoma G4 | Glioblastoma G4 |  |  | NA | NA | No combined CNA | Non-codel | WT | Methylated | NA | 34 | Classical | LGm4 | LGr4 | Classic-like |
| TCGA-06-2567-01 | TCGA-06-2567 | 65 | 1:DECEASED | Male | Primary | Glioblastoma G4 | Glioblastoma G4 |  |  | NA | NA | Gain chr 7 & loss chr 10 | Non-codel | WT | Methylated | NA | 43 | Neural | LGm5 | LGr4 | Mesenchymal-like |
| TCGA-06-2569-01 | TCGA-06-2569 | 24 | 0:LIVING | Female | Primary | Glioblastoma G4 | Glioblastoma G4 |  |  | NA | NA | No combined CNA | Non-codel | WT | Unmethylated | NA | 25 | Mesenchymal | LGm6 | LGr4 | LGm6-GBM |
| TCGA-06-2570-01 | TCGA-06-2570 | 21 | 0:LIVING | Female | Primary | Glioblastoma G4 | Astrocytoma, IDH-mutant G4 |  |  | NA | NA | No combined CNA | Non-codel | Mutant | Methylated | WT | 23 | G-CIMP | LGm1 | LGr1 | G-CIMP-low |
| TCGA-06-5408-01A | TCGA-06-5408 | 54 | 1:DECEASED | Female | Primary | Glioblastoma G4 | Glioblastoma G4 |  |  | DDRm1 | DDRr4 | Gain chr 7 & loss chr 10 | Non-codel | WT | Unmethylated | NA | 34 | Classical | LGm5 | LGr4 | Mesenchymal-like |
| TCGA-06-5410-01A | TCGA-06-5410 | 72 | 1:DECEASED | Female | Primary | Glioblastoma G4 | Glioblastoma G4 |  |  | DDRm1 | DDRr4 | No combined CNA | Non-codel | WT | Methylated | NA | 5 | Mesenchymal | LGm6 | LGr4 | LGm6-GBM |
| TCGA-06-5411-01A | TCGA-06-5411 | 51 | 1:DECEASED | Male | Primary | Glioblastoma G4 | Glioblastoma G4 |  |  | DDRm1 | DDRr4 | Gain chr 7 & loss chr 10 | Non-codel | WT | Unmethylated | Mutant | 21 | Neural | LGm5 | LGr2 | Mesenchymal-like |
| TCGA-06-5412-01A | TCGA-06-5412 | 78 | 1:DECEASED | Female | Primary | Glioblastoma G4 | Glioblastoma G4 |  |  | DDRm1 | DDRr4 | No combined CNA | Non-codel | WT | Methylated | NA | 32 | Mesenchymal | LGm5 | LGr4 | Mesenchymal-like |
| TCGA-06-5413-01A | TCGA-06-5413 | 67 | 0:LIVING | Male | Primary | Glioblastoma G4 | Glioblastoma G4 |  |  | DDRm1 | DDRr4 | Gain chr 7 & loss chr 10 | Non-codel | WT | Unmethylated | NA | 37 | Neural | LGm5 | LGr4 | Classic-like |
| TCGA-06-5414-01A | TCGA-06-5414 | 61 | 0:LIVING | Male | Primary | Glioblastoma G4 | Glioblastoma G4 |  |  | DDRm1 | DDRr4 | Gain chr 7 & loss chr 10 | Non-codel | WT | Unmethylated | NA | 27 | Classical | LGm4 | LGr4 | Classic-like |
| TCGA-06-5415-01A | TCGA-06-5415 | 60 | 0:LIVING | Male | Primary | Glioblastoma G4 | Glioblastoma G4 |  |  | NA | NA | Gain chr 7 & loss chr 10 | Non-codel | WT | Unmethylated | Mutant | 38 | Classical | LGm4 | LGr4 | Classic-like |
| TCGA-06-5416-01A | TCGA-06-5416 | 23 | 0:LIVING | Female | Primary | Glioblastoma G4 | Glioma NOS |  |  | DDRm1 | DDRr4 | No combined CNA | Non-codel | NA | Unmethylated | NA | NA | Proneural | LGm6 | LGr1 | NA |
| TCGA-06-5417-01A | TCGA-06-5417 | 45 | 0:LIVING | Female | Primary | Glioblastoma G4 | Glioma, IDH-mutant NOS |  |  | DDRm2 | DDRr4 | NA | NA | Mutant | Methylated | NA | 42 | G-CIMP | LGm1 | LGr3 | NA |
| TCGA-06-5418-01A | TCGA-06-5418 | 75 | 1:DECEASED | Female | Primary | Glioblastoma G4 | Glioblastoma G4 |  |  | DDRm1 | DDRr4 | Gain chr 7 & loss chr 10 | Non-codel | WT | Unmethylated | NA | 33 | Mesenchymal | LGm5 | LGr4 | Mesenchymal-like |
| TCGA-06-5856-01A | TCGA-06-5856 | 58 | 1:DECEASED | Male | Primary | Glioblastoma G4 | Glioblastoma G4 |  |  | DDRm1 | DDRr4 | Gain chr 7 & loss chr 10 | Non-codel | WT | Unmethylated | NA | 36 | Classical | LGm4 | LGr4 | Classic-like |
| TCGA-06-5858-01A | TCGA-06-5858 | 45 | 0:LIVING | Female | Primary | Glioblastoma G4 | Glioblastoma G4 |  |  | DDRm1 | DDRr4 | No combined CNA | Non-codel | WT | Unmethylated | NA | 124 | Mesenchymal | LGm6 | LGr4 | LGm6-GBM |
| TCGA-06-5859-01A | TCGA-06-5859 | 63 | 0:LIVING | Male | Primary | Glioblastoma G4 | Glioblastoma G4 |  |  | DDRm1 | DDRr4 | Gain chr 7 & loss chr 10 | Non-codel | WT | Unmethylated | NA | 37 | Neural | LGm4 | LGr4 | Classic-like |
| TCGA-06-6388-01A | TCGA-06-6388 | 64 | 1:DECEASED | Female | Primary | Glioblastoma G4 | Glioblastoma G4 |  |  | NA | NA | Gain chr 7 & loss chr 10 | Non-codel | WT | Unmethylated | NA | 34 | NA | LGm5 | NA | Mesenchymal-like |
| TCGA-06-6389-01A | TCGA-06-6389 | 49 | 0:LIVING | Female | Primary | Glioblastoma G4 | Astrocytoma, IDH-mutant G4 |  |  | NA | NA | No combined CNA | Non-codel | Mutant | Methylated | NA | 20 | G-CIMP | LGm1 | LGr3 | G-CIMP-high |
| TCGA-06-6390-01A | TCGA-06-6390 | 58 | 1:DECEASED | Male | Primary | Glioblastoma G4 | Glioblastoma G4 |  |  | NA | NA | Gain chr 7 & loss chr 10 | Non-codel | WT | Unmethylated | NA | 26 | Classical | LGm5 | LGr4 | Mesenchymal-like |
| TCGA-06-6391-01A | TCGA-06-6391 | 44 | 1:DECEASED | Female | Primary | Glioblastoma G4 | Glioblastoma G4 |  |  | NA | NA | No combined CNA | Non-codel | WT | Unmethylated | NA | 44 | Proneural | LGm6 | LGr2 | LGm6-GBM |
| TCGA-06-6693-01A | TCGA-06-6693 | 64 | 1:DECEASED | Female | Primary | Glioblastoma G4 | Glioblastoma G4 |  |  | NA | NA | No combined CNA | Non-codel | WT | Unmethylated | NA | 37 | NA | LGm4 | NA | Classic-like |
| TCGA-06-6694-01A | TCGA-06-6694 | 76 | 1:DECEASED | Female | Primary | Glioblastoma G4 | Glioblastoma G4 |  |  | NA | NA | No combined CNA | Non-codel | WT | Methylated | NA | 46 | NA | LGm5 | NA | Mesenchymal-like |
| TCGA-06-6695-01A | TCGA-06-6695 | 64 | 0:LIVING | Male | Primary | Glioblastoma G4 | Glioblastoma G4 |  |  | NA | NA | Gain chr 7 & loss chr 10 | Non-codel | WT | Methylated | NA | 47 | NA | LGm4 | NA | Classic-like |
| TCGA-06-6697-01A | TCGA-06-6697 | 65 | 0:LIVING | Male | Primary | Glioblastoma G4 | Glioblastoma G4 |  |  | NA | NA | Gain chr 7 & loss chr 10 | Non-codel | WT | Unmethylated | NA | 46 | NA | LGm5 | NA | Mesenchymal-like |
| TCGA-06-6698-01A | TCGA-06-6698 | 53 | 0:LIVING | Female | Primary | Glioblastoma G4 | Glioblastoma G4 |  |  | NA | NA | No combined CNA | Non-codel | WT | Unmethylated | NA | 28 | NA | LGm6 | NA | LGm6-GBM |
| TCGA-06-6699-01A | TCGA-06-6699 | 58 | 0:LIVING | Female | Primary | Glioblastoma G4 | Glioblastoma G4 |  |  | NA | NA | Gain chr 7 & loss chr 10 | Non-codel | WT | Unmethylated | NA | 45 | NA | LGm4 | NA | Classic-like |
| TCGA-06-6700-01A | TCGA-06-6700 | 76 | 0:LIVING | Male | Primary | Glioblastoma G4 | Glioblastoma G4 |  |  | NA | NA | No combined CNA | Non-codel | WT | Methylated | NA | 40 | NA | LGm5 | NA | Mesenchymal-like |
| TCGA-06-6701-01A | TCGA-06-6701 | 60 | 0:LIVING | Male | Primary | Glioblastoma G4 | Astrocytoma, IDH-mutant G4 |  |  | NA | NA | No combined CNA | Non-codel | Mutant | Methylated | NA | 26 | NA | LGm2 | NA | G-CIMP-high |
| TCGA-06-A5U0-01A | TCGA-06-A5U0 | 21 | 0:LIVING | Female | Primary | Glioblastoma G4 | Glioblastoma G4 |  |  | NA | NA | No combined CNA | Non-codel | WT | Methylated | NA | 26 | NA | LGm6 | NA | LGm6-GBM |
| TCGA-06-A5U1-01A | TCGA-06-A5U1 | 78 | 1:DECEASED | Female | Primary | Glioblastoma G4 | Glioblastoma G4 |  |  | NA | NA | Gain chr 7 & loss chr 10 | Non-codel | WT | Methylated | NA | 44 | NA | LGm5 | NA | Mesenchymal-like |
| TCGA-06-A6S0-01A | TCGA-06-A6S0 | 79 | 1:DECEASED | Male | Primary | Glioblastoma G4 | Glioblastoma G4 |  |  | NA | NA | No combined CNA | Non-codel | WT | Unmethylated | NA | 39 | NA | LGm4 | NA | Classic-like |
| TCGA-06-A6S1-01A | TCGA-06-A6S1 | 53 | 1:DECEASED | Female | Primary | Glioblastoma G4 | Glioblastoma G4 |  |  | NA | NA | Gain chr 7 & loss chr 10 | Non-codel | WT | Unmethylated | NA | 30 | NA | LGm5 | NA | Mesenchymal-like |
| TCGA-06-A7TK-01A | TCGA-06-A7TK | 64 | 0:LIVING | Male | Primary | Glioblastoma G4 | Glioblastoma G4 |  |  | NA | NA | NA | Non-codel | WT | Methylated | NA | 43 | NA | LGm4 | NA | Classic-like |
| TCGA-06-A7TL-01A | TCGA-06-A7TL | NA | NA | NA | Primary | Glioblastoma G4 | Astrocytoma, IDH-mutant G4 |  |  | NA | NA | NA | Non-codel | Mutant | Methylated | NA | 22 | NA | LGm1 | NA | G-CIMP-high |
| TCGA-08-0386-01 | TCGA-08-0386 | 74 | 1:DECEASED | Male | Primary | Glioblastoma G4 | Glioblastoma G4 |  |  | NA | NA | Gain chr 7 & loss chr 10 | Non-codel | WT | NA | NA | 5 | Neural | NA | LGr4 | NA |
| TCGA-12-0616-01 | TCGA-12-0616 | 36 | 1:DECEASED | Female | Primary | Glioblastoma G4 | Glioblastoma G4 |  |  | NA | NA | No combined CNA | Non-codel | WT | NA | NA | 25 | Proneural | NA | LGr1 | NA |
| TCGA-12-0618-01 | TCGA-12-0618 | 49 | 1:DECEASED | Male | Primary | Glioblastoma G4 | Glioblastoma G4 |  |  | NA | NA | Gain chr 7 & loss chr 10 | Non-codel | WT | NA | NA | 31 | Proneural | NA | LGr1 | NA |
| TCGA-12-0619-01 | TCGA-12-0619 | 60 | 1:DECEASED | Male | Primary | Glioblastoma G4 | Glioblastoma G4 |  |  | NA | NA | Gain chr 7 & loss chr 10 | Non-codel | WT | NA | NA | 47 | Mesenchymal | NA | LGr4 | NA |
| TCGA-12-0821-01 | TCGA-12-0821 | 62 | 1:DECEASED | Male | Primary | Glioblastoma G4 | Glioblastoma G4 |  |  | NA | NA | Gain chr 7 & loss chr 10 | Non-codel | WT | Unmethylated | NA | 45 | Neural | LGm6 | LGr4 | LGm6-GBM |
| TCGA-12-1597-01 | TCGA-12-1597 | 62 | 1:DECEASED | Female | Primary | Glioblastoma G4 | Glioblastoma G4 |  |  | NA | NA | NA | NA | WT | Unmethylated | NA | 40 | Proneural | LGm4 | LGr1 | Classic-like |
| TCGA-12-3650-01 | TCGA-12-3650 | 46 | 1:DECEASED | Male | Primary | Glioblastoma G4 | Glioblastoma G4 |  |  | NA | NA | Gain chr 7 & loss chr 10 | Non-codel | WT | Unmethylated | NA | 41 | Proneural | LGm4 | LGr1 | Classic-like |
| TCGA-12-3652-01 | TCGA-12-3652 | 60 | 1:DECEASED | Male | Primary | Glioblastoma G4 | Glioblastoma G4 |  |  | NA | NA | No combined CNA | Non-codel | WT | Unmethylated | NA | 47 | Classical | LGm5 | LGr4 | Classic-like |
| TCGA-12-3653-01 | TCGA-12-3653 | 34 | 1:DECEASED | Female | Primary | Glioblastoma G4 | Glioblastoma G4 |  |  | NA | NA | Gain chr 7 & loss chr 10 | Non-codel | WT | Unmethylated | NA | 23 | Classical | LGm5 | LGr4 | Mesenchymal-like |
| TCGA-12-5295-01A | TCGA-12-5295 | 60 | 1:DECEASED | Female | Primary | Glioblastoma G4 | Glioblastoma G4 |  |  | DDRm1 | DDRr4 | Gain chr 7 & loss chr 10 | Non-codel | WT | Methylated | NA | 58 | Neural | LGm4 | LGr4 | Classic-like |
| TCGA-12-5299-01A | TCGA-12-5299 | 56 | 1:DECEASED | Female | Primary | Glioblastoma G4 | Glioblastoma G4 |  |  | DDRm1 | DDRr4 | Gain chr 7 & loss chr 10 | Non-codel | WT | Unmethylated | NA | 28 | Classical | LGm5 | LGr4 | Mesenchymal-like |
| TCGA-12-5301-01A | TCGA-12-5301 | 59 | 1:DECEASED | Male | Primary | Glioblastoma G4 | Glioblastoma G4 |  |  | NA | NA | Gain chr 7 & loss chr 10 | Non-codel | WT | Methylated | NA | 51 | Neural | LGm5 | LGr4 | Mesenchymal-like |
| TCGA-14-0736-01A | TCGA-14-0736 | 49 | 1:DECEASED | Male | Primary | Glioblastoma G4 | Glioma NOS |  |  | NA | NA | Gain chr 7 & loss chr 10 | Non-codel | NA | Unmethylated | NA | NA | Mesenchymal | LGm6 | LGr4 | NA |
| TCGA-14-0736-02A | TCGA-14-0736 | NA | NA | NA | Recurrent | Glioblastoma G4 | Glioma NOS |  |  | DDRm1 | DDRr4 | NA | NA | NA | NA | NA | NA | NA | NA | NA | NA |
| TCGA-14-0740-01B | TCGA-14-0740 | 70 | 1:DECEASED | Male | Primary | Glioblastoma G4 | Glioblastoma G4 |  |  | NA | NA | No combined CNA | Non-codel | WT | Methylated | NA | 34 | NA | LGm6 | NA | LGm6-GBM |
| TCGA-14-0781-01B | TCGA-14-0781 | 49 | 1:DECEASED | Male | Primary | Glioblastoma G4 | Glioblastoma G4 |  |  | DDRm1 | DDRr4 | No combined CNA | Non-codel | WT | Unmethylated | NA | 19 | Mesenchymal | LGm5 | LGr4 | Mesenchymal-like |
| TCGA-14-0787-01 | TCGA-14-0787 | 69 | 1:DECEASED | Male | Primary | Glioblastoma G4 | Glioblastoma G4 |  |  | NA | NA | Gain chr 7 & loss chr 10 | Non-codel | WT | Methylated | NA | 25 | Classical | LGm4 | LGr4 | Classic-like |
| TCGA-14-0789-01 | TCGA-14-0789 | 54 | 1:DECEASED | Male | Primary | Glioblastoma G4 | Glioblastoma G4 |  |  | NA | NA | Gain chr 7 & loss chr 10 | Non-codel | WT | Methylated | NA | 54 | Mesenchymal | LGm5 | LGr4 | Classic-like |
| TCGA-14-0790-01 | TCGA-14-0790 | 64 | 1:DECEASED | Female | Primary | Glioblastoma G4 | Glioblastoma G4 |  |  | NA | NA | Gain chr 7 & loss chr 10 | Non-codel | WT | Methylated | NA | 49 | Classical | LGm4 | LGr4 | Classic-like |
| TCGA-14-0817-01 | TCGA-14-0817 | 69 | 1:DECEASED | Female | Primary | Glioblastoma G4 | Glioblastoma G4 |  |  | NA | NA | Gain chr 7 & loss chr 10 | Non-codel | WT | Unmethylated | NA | 54 | Neural | LGm4 | LGr4 | Classic-like |
| TCGA-14-0862-01B | TCGA-14-0862 | 60 | 1:DECEASED | Male | Primary | Glioblastoma G4 | Glioblastoma G4 |  |  | NA | NA | No combined CNA | Non-codel | WT | Methylated | NA | 31 | NA | LGm4 | NA | Classic-like |
| TCGA-14-0871-01 | TCGA-14-0871 | 74 | 1:DECEASED | Female | Primary | Glioblastoma G4 | Glioblastoma G4 |  |  | NA | NA | Gain chr 7 & loss chr 10 | Non-codel | WT | Unmethylated | NA | 39 | Mesenchymal | LGm6 | LGr4 | LGm6-GBM |
| TCGA-14-1034-01 | TCGA-14-1034 | 60 | 1:DECEASED | Female | Primary | Glioblastoma G4 | Glioblastoma G4 |  |  | NA | NA | Gain chr 7 & loss chr 10 | Non-codel | WT | Methylated | Mutant | 60 | Mesenchymal | LGm4 | LGr4 | Classic-like |
| TCGA-14-1034-02B | TCGA-14-1034 | NA | NA | NA | Recurrent | Glioblastoma G4 | Glioblastoma G4 |  |  | DDRm1 | DDRr4 | NA | NA | NA | NA | NA | NA | NA | NA | NA | NA |
| TCGA-14-1043-01B | TCGA-14-1043 | 61 | 1:DECEASED | Male | Primary | Glioblastoma G4 | Glioblastoma G4 |  |  | NA | NA | Gain chr 7 & loss chr 10 | Non-codel | WT | Unmethylated | NA | 16 | NA | LGm5 | NA | Mesenchymal-like |
| TCGA-14-1395-01B | TCGA-14-1395 | 52 | 1:DECEASED | Male | Primary | Glioblastoma G4 | Glioblastoma G4 |  |  | NA | NA | Gain chr 7 & loss chr 10 | Non-codel | WT | Unmethylated | NA | 44 | NA | LGm5 | NA | Mesenchymal-like |
| TCGA-14-1402-01A | TCGA-14-1402 | 58 | 1:DECEASED | Female | Primary | Glioblastoma G4 | Glioma NOS |  |  | NA | NA | No combined CNA | Non-codel | NA | Methylated | Mutant | NA | Classical | LGm4 | LGr4 | NA |
| TCGA-14-1402-02A | TCGA-14-1402 | NA | NA | NA | Recurrent | Glioblastoma G4 | Glioma NOS |  |  | DDRm1 | DDRr4 | NA | NA | NA | NA | NA | NA | NA | NA | NA | NA |
| TCGA-14-1450-01B | TCGA-14-1450 | 57 | 0:LIVING | Female | Primary | Glioblastoma G4 | Glioblastoma G4 |  |  | NA | NA | Gain chr 7 & loss chr 10 | Non-codel | WT | Methylated | NA | 43 | NA | LGm4 | NA | Classic-like |
| TCGA-14-1823-01 | TCGA-14-1823 | 58 | 1:DECEASED | Female | Primary | Glioblastoma G4 | Glioblastoma G4 |  |  | NA | NA | Gain chr 7 & loss chr 10 | Non-codel | WT | Methylated | Mutant | 38 | Mesenchymal | LGm4 | LGr4 | Classic-like |
| TCGA-14-1825-01 | TCGA-14-1825 | 70 | 1:DECEASED | Male | Primary | Glioblastoma G4 | Glioblastoma G4 |  |  | NA | NA | Gain chr 7 & loss chr 10 | Non-codel | WT | Unmethylated | NA | 40 | Proneural | LGm5 | LGr1 | Mesenchymal-like |
| TCGA-14-1829-01 | TCGA-14-1829 | 57 | 0:LIVING | Male | Primary | Glioblastoma G4 | Glioblastoma G4 |  |  | NA | NA | Gain chr 7 & loss chr 10 | Non-codel | WT | Unmethylated | NA | 37 | Neural | LGm5 | LGr4 | Mesenchymal-like |
| TCGA-14-2554-01 | TCGA-14-2554 | 52 | 1:DECEASED | Female | Primary | Glioblastoma G4 | Glioblastoma G4 |  |  | NA | NA | Gain chr 7 & loss chr 10 | Non-codel | WT | Unmethylated | Mutant | 54 | Neural | LGm5 | LGr4 | Mesenchymal-like |
| TCGA-15-0742-01 | TCGA-15-0742 | 65 | 1:DECEASED | Male | Primary | Glioblastoma G4 | Glioblastoma G4 |  |  | NA | NA | Gain chr 7 & loss chr 10 | Non-codel | WT | NA | NA | 60 | Classical | NA | LGr4 | NA |
| TCGA-15-1444-01A | TCGA-15-1444 | 21 | 1:DECEASED | Male | Primary | Glioblastoma G4 | Astrocytoma, IDH-mutant G4 |  |  | DDRm2 | DDRr1 | No combined CNA | Non-codel | Mutant | Methylated | WT | 16 | Proneural | LGm2 | LGr3 | G-CIMP-high |
| TCGA-16-0846-01 | TCGA-16-0846 | 85 | 1:DECEASED | Male | Primary | Glioblastoma G4 | Glioblastoma G4 |  |  | NA | NA | Gain chr 7 & loss chr 10 | Non-codel | WT | Methylated | NA | 44 | Proneural | LGm5 | LGr4 | Classic-like |
| TCGA-16-1045-01 | TCGA-16-1045 | 49 | 1:DECEASED | Female | Primary | Glioblastoma G4 | Glioblastoma G4 |  |  | NA | NA | Gain chr 7 & loss chr 10 | Non-codel | WT | Methylated | NA | 55 | Mesenchymal | LGm5 | LGr4 | Mesenchymal-like |
| TCGA-19-0957-01C | TCGA-19-0957 | 48 | 1:DECEASED | Female | Primary | Glioblastoma G4 | Glioblastoma G4 |  |  | NA | NA | Gain chr 7 & loss chr 10 | Non-codel | WT | Unmethylated | NA | NA | Proneural | LGm4 | LGr1 | Classic-like |
| TCGA-19-0957-02A | TCGA-19-0957 | NA | NA | NA | Recurrent | Glioblastoma G4 | Glioblastoma G4 |  |  | DDRm1 | DDRr4 | NA | NA | NA | NA | NA | NA | NA | NA | NA | NA |
| TCGA-19-1389-01A | TCGA-19-1389 | 51 | 1:DECEASED | Male | Primary | Glioblastoma G4 | Glioblastoma G4 |  |  | NA | NA | Gain chr 7 & loss chr 10 | Non-codel | WT | Unmethylated | Mutant | NA | Mesenchymal | LGm6 | LGr4 | LGm6-GBM |
| TCGA-19-1389-02A | TCGA-19-1389 | NA | NA | NA | Recurrent | Glioblastoma G4 | Glioblastoma G4 |  |  | DDRm1 | DDRr4 | NA | NA | NA | NA | NA | NA | NA | NA | NA | NA |
| TCGA-19-1390-01 | TCGA-19-1390 | 63 | 1:DECEASED | Female | Primary | Glioblastoma G4 | Glioblastoma G4 |  |  | NA | NA | No combined CNA | Non-codel | WT | Methylated | NA | 81 | Proneural | LGm4 | LGr1 | Classic-like |
| TCGA-19-1787-01 | TCGA-19-1787 | 48 | 1:DECEASED | Male | Primary | Glioblastoma G4 | Glioma NOS |  |  | NA | NA | Gain chr 7 & loss chr 10 | Non-codel | NA | Methylated | NA | NA | Mesenchymal | LGm4 | LGr4 | NA |
| TCGA-19-2619-01 | TCGA-19-2619 | 55 | 0:LIVING | Female | Primary | Glioblastoma G4 | Glioblastoma G4 |  |  | NA | NA | Gain chr 7 & loss chr 10 | Non-codel | WT | Methylated | NA | 35 | Classical | LGm5 | LGr4 | Mesenchymal-like |
| TCGA-19-2620-01 | TCGA-19-2620 | 70 | 1:DECEASED | Male | Primary | Glioblastoma G4 | Glioblastoma G4 |  |  | NA | NA | No combined CNA | Non-codel | WT | Methylated | Mutant | 54 | Neural | LGm4 | LGr4 | Classic-like |
| TCGA-19-2624-01 | TCGA-19-2624 | 51 | 1:DECEASED | Male | Primary | Glioblastoma G4 | Glioblastoma G4 |  |  | NA | NA | Gain chr 7 & loss chr 10 | Non-codel | WT | Unmethylated | Mutant | 25 | Proneural | LGm5 | LGr1 | Mesenchymal-like |
| TCGA-19-2625-01 | TCGA-19-2625 | 76 | 1:DECEASED | Female | Primary | Glioblastoma G4 | Glioblastoma G4 |  |  | NA | NA | No combined CNA | Non-codel | WT | Unmethylated | NA | 42 | Classical | LGm5 | LGr4 | Mesenchymal-like |
| TCGA-19-2629-01 | TCGA-19-2629 | 60 | 1:DECEASED | Male | Primary | Glioblastoma G4 | Astrocytoma, IDH-mutant G4 |  |  | NA | NA | No combined CNA | Non-codel | Mutant | Unmethylated | WT | 59 | G-CIMP | LGm1 | LGr3 | G-CIMP-low |
| TCGA-19-4065-01A | TCGA-19-4065 | 36 | 0:LIVING | Male | Primary | Glioblastoma G4 | Glioma NOS |  |  | DDRm1 | DDRr4 | Gain chr 7 & loss chr 10 | Non-codel | NA | Unmethylated | NA | NA | NA | LGm5 | LGr4 | NA |
| TCGA-19-4065-02A | TCGA-19-4065 | NA | NA | NA | Recurrent | Glioblastoma G4 | Glioma NOS |  |  | DDRm1 | DDRr4 | NA | NA | NA | NA | NA | NA | NA | NA | NA | NA |
| TCGA-19-5947-01A | TCGA-19-5947 | 47 | 1:DECEASED | Female | Primary | Glioblastoma G4 | Glioblastoma G4 |  |  | NA | NA | Gain chr 7 & loss chr 10 | Non-codel | WT | Unmethylated | NA | 20 | Mesenchymal | LGm5 | LGr4 | Mesenchymal-like |
| TCGA-19-5950-01A | TCGA-19-5950 | 52 | 0:LIVING | Female | Primary | Glioblastoma G4 | Glioblastoma G4 |  |  | NA | NA | Gain chr 7 & loss chr 10 | Non-codel | WT | Methylated | NA | 48 | Classical | LGm4 | LGr4 | Classic-like |
| TCGA-19-5951-01A | TCGA-19-5951 | 76 | 1:DECEASED | Female | Primary | Glioblastoma G4 | Glioblastoma G4 |  |  | NA | NA | No combined CNA | Non-codel | WT | Unmethylated | NA | 58 | Classical | LGm4 | LGr4 | Classic-like |
| TCGA-19-5952-01A | TCGA-19-5952 | 62 | 1:DECEASED | Male | Primary | Glioblastoma G4 | Glioblastoma G4 |  |  | NA | NA | Gain chr 7 & loss chr 10 | Non-codel | WT | Unmethylated | NA | 33 | Classical | LGm5 | LGr4 | Mesenchymal-like |
| TCGA-19-5953-01B | TCGA-19-5953 | 58 | 1:DECEASED | Male | Primary | Glioblastoma G4 | Glioblastoma G4 |  |  | NA | NA | Gain chr 7 & loss chr 10 | Non-codel | WT | Methylated | NA | 34 | NA | LGm4 | NA | Classic-like |
| TCGA-19-5954-01A | TCGA-19-5954 | 72 | 0:LIVING | Female | Primary | Glioblastoma G4 | Glioblastoma G4 |  |  | NA | NA | Gain chr 7 & loss chr 10 | Non-codel | WT | Methylated | NA | 64 | Classical | LGm5 | LGr4 | Mesenchymal-like |
| TCGA-19-5955-01A | TCGA-19-5955 | 83 | 1:DECEASED | Male | Primary | Glioblastoma G4 | Glioblastoma G4 |  |  | NA | NA | No combined CNA | Non-codel | WT | Methylated | NA | 39 | Mesenchymal | LGm5 | LGr4 | Mesenchymal-like |
| TCGA-19-5956-01A | TCGA-19-5956 | 53 | 0:LIVING | Female | Primary | Glioblastoma G4 | Glioma NOS |  |  | NA | NA | No combined CNA | Non-codel | NA | Unmethylated | NA | NA | Proneural | LGm6 | LGr1 | NA |
| TCGA-19-5958-01A | TCGA-19-5958 | 56 | 0:LIVING | Male | Primary | Glioblastoma G4 | Glioblastoma G4 |  |  | NA | NA | No combined CNA | Non-codel | WT | Unmethylated | NA | 31 | Classical | LGm5 | LGr4 | Mesenchymal-like |
| TCGA-19-5959-01A | TCGA-19-5959 | 77 | 0:LIVING | Female | Primary | Glioblastoma G4 | Glioblastoma G4 |  |  | NA | NA | Gain chr 7 & loss chr 10 | Non-codel | WT | Methylated | NA | 67 | Classical | LGm4 | LGr4 | Classic-like |
| TCGA-19-5960-01A | TCGA-19-5960 | 56 | 0:LIVING | Male | Primary | Glioblastoma G4 | Glioblastoma G4 |  |  | DDRm1 | DDRr4 | Gain chr 7 & loss chr 10 | Non-codel | WT | Unmethylated | Mutant | 34 | Proneural | LGm4 | LGr4 | Classic-like |
| TCGA-19-A60I-01A | TCGA-19-A60I | 39 | 0:LIVING | Male | Primary | Glioblastoma G4 | Glioblastoma G4 |  |  | NA | NA | Gain chr 7 & loss chr 10 | Non-codel | WT | Methylated | NA | 22 | NA | LGm4 | NA | Classic-like |
| TCGA-19-A6J4-01A | TCGA-19-A6J4 | 68 | 1:DECEASED | Male | Primary | Glioblastoma G4 | Glioblastoma G4 |  |  | NA | NA | Gain chr 7 & loss chr 10 | Non-codel | WT | Unmethylated | NA | 20 | NA | LGm5 | NA | Mesenchymal-like |
| TCGA-19-A6J5-01A | TCGA-19-A6J5 | 40 | 0:LIVING | Male | Primary | Glioblastoma G4 | Astrocytoma, IDH-mutant G4 |  |  | NA | NA | Gain chr 7 & loss chr 10 | Non-codel | Mutant | Methylated | NA | 34 | NA | LGm3 | NA | G-CIMP-high |
| TCGA-26-1442-01A | TCGA-26-1442 | 43 | 0:LIVING | Male | Primary | Glioblastoma G4 | Astrocytoma, IDH-mutant G4 |  |  | DDRm2 | DDRr1 | No combined CNA | Non-codel | Mutant | Methylated | NA | 29 | G-CIMP | LGm2 | LGr3 | G-CIMP-high |
| TCGA-26-5132-01A | TCGA-26-5132 | 74 | 0:LIVING | Male | Primary | Glioblastoma G4 | Glioblastoma G4 |  |  | DDRm1 | DDRr4 | Gain chr 7 & loss chr 10 | Non-codel | WT | Methylated | Mutant | 36 | Classical | LGm4 | LGr4 | Classic-like |
| TCGA-26-5133-01A | TCGA-26-5133 | 59 | 0:LIVING | Male | Primary | Glioblastoma G4 | Glioblastoma G4 |  |  | DDRm1 | DDRr4 | No combined CNA | Non-codel | WT | Unmethylated | NA | 21 | G-CIMP | LGm1 | LGr3 | LGm6-GBM |
| TCGA-26-5134-01A | TCGA-26-5134 | 74 | 0:LIVING | Male | Primary | Glioblastoma G4 | Glioblastoma G4 |  |  | DDRm1 | DDRr4 | No combined CNA | Non-codel | WT | Unmethylated | NA | 45 | Proneural | LGm5 | LGr1 | Mesenchymal-like |
| TCGA-26-5135-01A | TCGA-26-5135 | 72 | 1:DECEASED | Female | Primary | Glioblastoma G4 | Glioblastoma G4 |  |  | DDRm1 | DDRr4 | Gain chr 7 & loss chr 10 | Non-codel | WT | Methylated | Mutant | 44 | Proneural | LGm5 | LGr1 | Mesenchymal-like |
| TCGA-26-5136-01B | TCGA-26-5136 | 78 | 0:LIVING | Female | Primary | Glioblastoma G4 | Glioblastoma G4 |  |  | DDRm1 | DDRr4 | Gain chr 7 & loss chr 10 | Non-codel | WT | Methylated | NA | 34 | Mesenchymal | LGm5 | LGr4 | Mesenchymal-like |
| TCGA-26-5139-01A | TCGA-26-5139 | 65 | 0:LIVING | Female | Primary | Glioblastoma G4 | Glioblastoma G4 |  |  | DDRm1 | DDRr4 | Gain chr 7 & loss chr 10 | Non-codel | WT | Unmethylated | NA | 33 | Mesenchymal | LGm5 | LGr4 | Mesenchymal-like |
| TCGA-26-6173-01A | TCGA-26-6173 | 57 | 0:LIVING | Male | Primary | Glioblastoma G4 | Glioblastoma G4 |  |  | NA | NA | No combined CNA | Non-codel | WT | Unmethylated | NA | 26 | NA | LGm5 | NA | Mesenchymal-like |
| TCGA-26-6174-01A | TCGA-26-6174 | 65 | 0:LIVING | Female | Primary | Glioblastoma G4 | Glioblastoma G4 |  |  | NA | NA | No combined CNA | Non-codel | WT | Methylated | NA | 71 | NA | LGm5 | NA | Mesenchymal-like |
| TCGA-26-A7UX-01B | TCGA-26-A7UX | 47 | 0:LIVING | Male | Primary | Glioblastoma G4 | Glioblastoma G4 |  |  | NA | NA | NA | Non-codel | WT | Methylated | NA | 30 | NA | LGm5 | NA | Mesenchymal-like |
| TCGA-27-1830-01 | TCGA-27-1830 | 57 | 1:DECEASED | Male | Primary | Glioblastoma G4 | Glioblastoma G4 |  |  | NA | NA | Gain chr 7 & loss chr 10 | Non-codel | WT | Unmethylated | NA | 31 | Proneural | LGm5 | LGr4 | Mesenchymal-like |
| TCGA-27-1831-01 | TCGA-27-1831 | 66 | 1:DECEASED | Male | Primary | Glioblastoma G4 | Glioblastoma G4 |  |  | NA | NA | Gain chr 7 & loss chr 10 | Non-codel | WT | Unmethylated | Mutant | 44 | Neural | LGm5 | LGr4 | Mesenchymal-like |
| TCGA-27-1832-01 | TCGA-27-1832 | 59 | 1:DECEASED | Female | Primary | Glioblastoma G4 | Glioblastoma G4 |  |  | NA | NA | Gain chr 7 & loss chr 10 | Non-codel | WT | Unmethylated | NA | 24 | Mesenchymal | LGm5 | LGr4 | Mesenchymal-like |
| TCGA-27-1834-01 | TCGA-27-1834 | 56 | 1:DECEASED | Male | Primary | Glioblastoma G4 | Glioblastoma G4 |  |  | NA | NA | Gain chr 7 & loss chr 10 | Non-codel | WT | Methylated | NA | 29 | Neural | LGm5 | LGr4 | Mesenchymal-like |
| TCGA-27-1835-01 | TCGA-27-1835 | 53 | 1:DECEASED | Female | Primary | Glioblastoma G4 | Glioblastoma G4 |  |  | NA | NA | Gain chr 7 & loss chr 10 | Non-codel | WT | Methylated | NA | 54 | Classical | LGm5 | LGr4 | Mesenchymal-like |
| TCGA-27-1837-01 | TCGA-27-1837 | 36 | 1:DECEASED | Male | Primary | Glioblastoma G4 | Glioblastoma G4 |  |  | NA | NA | No combined CNA | Non-codel | WT | Methylated | NA | 31 | Classical | LGm5 | LGr4 | Mesenchymal-like |
| TCGA-27-2519-01 | TCGA-27-2519 | 48 | 0:LIVING | Male | Primary | Glioblastoma G4 | Glioblastoma G4 |  |  | NA | NA | Gain chr 7 & loss chr 10 | Non-codel | WT | Unmethylated | NA | 33 | Mesenchymal | LGm5 | LGr4 | Mesenchymal-like |
| TCGA-27-2521-01 | TCGA-27-2521 | 34 | 0:LIVING | Male | Primary | Glioblastoma G4 | Astrocytoma, IDH-mutant G4 |  |  | NA | NA | No combined CNA | Non-codel | Mutant | Methylated | NA | 32 | G-CIMP | LGm1 | LGr3 | G-CIMP-low |
| TCGA-27-2523-01 | TCGA-27-2523 | 63 | 1:DECEASED | Male | Primary | Glioblastoma G4 | Glioblastoma G4 |  |  | NA | NA | Gain chr 7 & loss chr 10 | Non-codel | WT | Methylated | Mutant | 37 | Classical | LGm4 | LGr4 | Classic-like |
| TCGA-27-2524-01 | TCGA-27-2524 | 56 | 1:DECEASED | Male | Primary | Glioblastoma G4 | Glioblastoma G4 |  |  | NA | NA | Gain chr 7 & loss chr 10 | Non-codel | WT | Unmethylated | NA | 36 | Mesenchymal | LGm5 | LGr4 | Mesenchymal-like |
| TCGA-27-2526-01 | TCGA-27-2526 | 79 | 1:DECEASED | Female | Primary | Glioblastoma G4 | Glioblastoma G4 |  |  | NA | NA | No combined CNA | Non-codel | WT | Unmethylated | NA | 38 | Neural | LGm5 | LGr4 | Mesenchymal-like |
| TCGA-27-2528-01 | TCGA-27-2528 | 62 | 1:DECEASED | Male | Primary | Glioblastoma G4 | Glioblastoma G4 |  |  | NA | NA | No combined CNA | Non-codel | WT | Methylated | Mutant | 42 | Classical | LGm4 | LGr4 | Classic-like |
| TCGA-28-1747-01 | TCGA-28-1747 | 44 | 1:DECEASED | Male | Primary | Glioblastoma G4 | Glioblastoma G4 |  |  | NA | NA | No combined CNA | Non-codel | WT | Methylated | NA | 34 | Classical | LGm5 | LGr4 | Mesenchymal-like |
| TCGA-28-1753-01 | TCGA-28-1753 | 53 | 0:LIVING | Male | Primary | Glioblastoma G4 | Glioblastoma G4 |  |  | NA | NA | Gain chr 7 & loss chr 10 | Non-codel | WT | Unmethylated | NA | 35 | Mesenchymal | LGm5 | LGr4 | Mesenchymal-like |
| TCGA-28-2499-01 | TCGA-28-2499 | 59 | 0:LIVING | Male | Primary | Glioblastoma G4 | Glioblastoma G4 |  |  | NA | NA | NA | NA | WT | Unmethylated | NA | 24 | NA | LGm4 | LGr4 | Classic-like |
| TCGA-28-2501-01A | TCGA-28-2501 | NA | NA | NA | Primary | Glioblastoma G4 | Glioblastoma G4 |  |  | NA | NA | Gain chr 7 & loss chr 10 | Non-codel | WT | Methylated | NA | 29 | NA | LGm5 | LGr4 | Mesenchymal-like |
| TCGA-28-2509-01 | TCGA-28-2509 | 77 | 0:LIVING | Female | Primary | Glioblastoma G4 | Glioblastoma G4 |  |  | NA | NA | Gain chr 7 & loss chr 10 | Non-codel | WT | Methylated | NA | 41 | Mesenchymal | LGm4 | LGr4 | Classic-like |
| TCGA-28-2510-01A | TCGA-28-2510 | NA | NA | NA | Primary | Glioblastoma G4 | Glioblastoma G4 |  |  | DDRm1 | DDRr3 | No combined CNA | Non-codel | WT | Unmethylated | NA | 14 | NA | LGm6 | LGr2 | LGm6-GBM |
| TCGA-28-2513-01 | TCGA-28-2513 | 69 | 0:LIVING | Female | Primary | Glioblastoma G4 | Glioblastoma G4 |  |  | NA | NA | Gain chr 7 & loss chr 10 | Non-codel | WT | Unmethylated | NA | 53 | Mesenchymal | LGm5 | LGr4 | Mesenchymal-like |
| TCGA-28-2514-01 | TCGA-28-2514 | 45 | 0:LIVING | Male | Primary | Glioblastoma G4 | Glioblastoma G4 |  |  | NA | NA | No combined CNA | Non-codel | WT | Unmethylated | NA | 36 | Classical | LGm5 | LGr4 | Mesenchymal-like |
| TCGA-28-5204-01A | TCGA-28-5204 | 72 | 1:DECEASED | Male | Primary | Glioblastoma G4 | Glioblastoma G4 |  |  | DDRm1 | DDRr4 | Gain chr 7 & loss chr 10 | Non-codel | WT | Unmethylated | NA | 36 | Neural | LGm4 | LGr4 | Classic-like |
| TCGA-28-5207-01A | TCGA-28-5207 | 71 | 1:DECEASED | Male | Primary | Glioblastoma G4 | Glioblastoma G4 |  |  | DDRm1 | DDRr4 | Gain chr 7 & loss chr 10 | Non-codel | WT | Unmethylated | NA | 33 | Mesenchymal | LGm5 | LGr4 | Mesenchymal-like |
| TCGA-28-5208-01A | TCGA-28-5208 | 52 | 0:LIVING | Male | Primary | Glioblastoma G4 | Glioblastoma G4 |  |  | DDRm1 | DDRr4 | No combined CNA | Non-codel | WT | Methylated | NA | 48 | Mesenchymal | LGm4 | LGr4 | Classic-like |
| TCGA-28-5209-01A | TCGA-28-5209 | 66 | 0:LIVING | Female | Primary | Glioblastoma G4 | Glioblastoma G4 |  |  | DDRm1 | DDRr4 | Gain chr 7 & loss chr 10 | Non-codel | WT | Methylated | NA | 72 | Mesenchymal | LGm4 | LGr4 | Classic-like |
| TCGA-28-5211-01C | TCGA-28-5211 | 42 | 0:LIVING | Male | Primary | Glioblastoma G4 | Glioblastoma G4 |  |  | NA | NA | Gain chr 7 & loss chr 10 | Non-codel | WT | Unmethylated | NA | 35 | NA | LGm5 | NA | Mesenchymal-like |
| TCGA-28-5213-01A | TCGA-28-5213 | 72 | 0:LIVING | Male | Primary | Glioblastoma G4 | Glioblastoma G4 |  |  | DDRm1 | DDRr4 | Gain chr 7 & loss chr 10 | Non-codel | WT | Unmethylated | NA | 38 | Mesenchymal | LGm5 | LGr4 | Mesenchymal-like |
| TCGA-28-5214-01A | TCGA-28-5214 | 53 | 0:LIVING | Male | Primary | Glioblastoma G4 | Glioblastoma G4 |  |  | NA | NA | Gain chr 7 & loss chr 10 | Non-codel | WT | Unmethylated | NA | 24 | Mesenchymal | LGm4 | LGr4 | Classic-like |
| TCGA-28-5215-01A | TCGA-28-5215 | 62 | 1:DECEASED | Female | Primary | Glioblastoma G4 | Glioblastoma G4 |  |  | DDRm1 | DDRr4 | No combined CNA | Non-codel | WT | Methylated | NA | 22 | Mesenchymal | LGm5 | LGr3 | Mesenchymal-like |
| TCGA-28-5216-01A | TCGA-28-5216 | 52 | 0:LIVING | Male | Primary | Glioblastoma G4 | Glioblastoma G4 |  |  | DDRm1 | DDRr4 | No combined CNA | Non-codel | WT | Unmethylated | NA | 34 | Mesenchymal | LGm5 | LGr1 | Mesenchymal-like |
| TCGA-28-5218-01A | TCGA-28-5218 | 63 | 1:DECEASED | Male | Primary | Glioblastoma G4 | Glioblastoma G4 |  |  | DDRm1 | DDRr4 | No combined CNA | Non-codel | WT | Unmethylated | NA | 19 | Mesenchymal | LGm6 | LGr4 | LGm6-GBM |
| TCGA-28-5219-01A | TCGA-28-5219 | 47 | 0:LIVING | Female | Primary | Glioblastoma G4 | Glioblastoma G4 |  |  | NA | NA | Gain chr 7 & loss chr 10 | Non-codel | WT | Methylated | NA | 34 | Classical | LGm5 | LGr4 | Mesenchymal-like |
| TCGA-28-5220-01A | TCGA-28-5220 | 67 | 0:LIVING | Male | Primary | Glioblastoma G4 | Glioblastoma G4 |  |  | DDRm1 | DDRr4 | Gain chr 7 & loss chr 10 | Non-codel | WT | Unmethylated | NA | 28 | Classical | LGm5 | LGr4 | Mesenchymal-like |
| TCGA-28-6450-01A | TCGA-28-6450 | 60 | 1:DECEASED | Male | Primary | Glioblastoma G4 | Glioblastoma G4 |  |  | NA | NA | Gain chr 7 & loss chr 10 | Non-codel | WT | Unmethylated | NA | 49 | Classical | LGm4 | LGr4 | Classic-like |
| TCGA-32-1970-01 | TCGA-32-1970 | 59 | 1:DECEASED | Male | Primary | Glioblastoma G4 | Glioblastoma G4 |  |  | NA | NA | Gain chr 7 & loss chr 10 | Non-codel | WT | Unmethylated | Mutant | 57 | Classical | LGm5 | LGr4 | Mesenchymal-like |
| TCGA-32-1979-01A | TCGA-32-1979 | 69 | 1:DECEASED | Female | Primary | Glioblastoma G4 | Glioblastoma G4 |  |  | NA | NA | Gain chr 7 & loss chr 10 | Non-codel | WT | Unmethylated | NA | 56 | Classical | LGm4 | LGr4 | Classic-like |
| TCGA-32-1980-01A | TCGA-32-1980 | 72 | 1:DECEASED | Male | Primary | Glioblastoma G4 | Glioblastoma G4 |  |  | DDRm1 | DDRr1 | No combined CNA | Non-codel | WT | Unmethylated | NA | 5 | Neural | LGm6 | LGr2 | LGm6-GBM |
| TCGA-32-1982-01 | TCGA-32-1982 | 76 | 1:DECEASED | Female | Primary | Glioblastoma G4 | Glioblastoma G4 |  |  | NA | NA | Gain chr 7 & loss chr 10 | Non-codel | WT | Methylated | NA | 56 | Neural | LGm4 | LGr4 | Classic-like |
| TCGA-32-2615-01 | TCGA-32-2615 | 62 | 1:DECEASED | Male | Primary | Glioblastoma G4 | Glioblastoma G4 |  |  | NA | NA | No combined CNA | Non-codel | WT | Unmethylated | NA | 25 | Mesenchymal | LGm5 | LGr4 | Mesenchymal-like |
| TCGA-32-2616-01 | TCGA-32-2616 | 48 | 1:DECEASED | Female | Primary | Glioblastoma G4 | Glioma NOS |  |  | NA | NA | No combined CNA | Non-codel | NA | Methylated | NA | NA | Mesenchymal | LGm5 | LGr4 | NA |
| TCGA-32-2632-01 | TCGA-32-2632 | 80 | 1:DECEASED | Male | Primary | Glioblastoma G4 | Glioblastoma G4 |  |  | NA | NA | No combined CNA | Non-codel | WT | Unmethylated | NA | 69 | Mesenchymal | LGm5 | LGr4 | Mesenchymal-like |
| TCGA-32-2634-01 | TCGA-32-2634 | 82 | 0:LIVING | Male | Primary | Glioblastoma G4 | Glioblastoma G4 |  |  | NA | NA | No combined CNA | Non-codel | WT | Methylated | NA | 40 | Proneural | LGm4 | LGr1 | Classic-like |
| TCGA-32-2638-01 | TCGA-32-2638 | 67 | 0:LIVING | Male | Primary | Glioblastoma G4 | Glioblastoma G4 |  |  | NA | NA | Gain chr 7 & loss chr 10 | Non-codel | WT | Methylated | NA | 34 | Classical | LGm4 | LGr4 | Classic-like |
| TCGA-32-4213-01 | TCGA-32-4213 | 47 | 0:LIVING | Female | Primary | Glioblastoma G4 | Glioblastoma G4 |  |  | NA | NA | No combined CNA | Non-codel | WT | Methylated | NA | 39 | Mesenchymal | LGm5 | LGr4 | Mesenchymal-like |
| TCGA-32-5222-01A | TCGA-32-5222 | 66 | 0:LIVING | Male | Primary | Glioblastoma G4 | Glioblastoma G4 |  |  | DDRm1 | DDRr4 | Gain chr 7 & loss chr 10 | Non-codel | WT | Methylated | NA | 49 | Proneural | LGm4 | LGr4 | Classic-like |
| TCGA-41-2571-01 | TCGA-41-2571 | 89 | 1:DECEASED | Male | Primary | Glioblastoma G4 | Glioblastoma G4 |  |  | NA | NA | Gain chr 7 & loss chr 10 | Non-codel | WT | Unmethylated | NA | 38 | Proneural | LGm5 | LGr1 | Mesenchymal-like |
| TCGA-41-2572-01 | TCGA-41-2572 | 67 | 1:DECEASED | Male | Primary | Glioblastoma G4 | Glioblastoma G4 |  |  | NA | NA | Gain chr 7 & loss chr 10 | Non-codel | WT | Unmethylated | NA | 33 | Classical | LGm4 | LGr4 | Classic-like |
| TCGA-41-3915-01 | TCGA-41-3915 | 48 | 0:LIVING | Male | Primary | Glioblastoma G4 | Glioblastoma G4 |  |  | NA | NA | Gain chr 7 & loss chr 10 | Non-codel | WT | Methylated | NA | 22 | Mesenchymal | LGm5 | LGr4 | Mesenchymal-like |
| TCGA-41-4097-01 | TCGA-41-4097 | 63 | 1:DECEASED | Female | Primary | Glioblastoma G4 | Glioblastoma G4 |  |  | NA | NA | No combined CNA | Non-codel | WT | Unmethylated | NA | 23 | Mesenchymal | LGm4 | LGr4 | Classic-like |
| TCGA-41-5651-01A | TCGA-41-5651 | 59 | 0:LIVING | Female | Primary | Glioblastoma G4 | Glioblastoma G4 |  |  | DDRm1 | DDRr4 | Gain chr 7 & loss chr 10 | Non-codel | WT | Methylated | Mutant | 63 | Proneural | LGm4 | LGr1 | Classic-like |
| TCGA-41-6646-01A | TCGA-41-6646 | 73 | 0:LIVING | Female | Primary | Glioblastoma G4 | Glioblastoma G4 |  |  | NA | NA | Gain chr 7 & loss chr 10 | Non-codel | WT | Methylated | NA | 44 | NA | LGm4 | NA | Classic-like |
| TCGA-4W-AA9R-01A | TCGA-4W-AA9R | NA | NA | NA | Primary | Glioblastoma G4 | Glioblastoma G4 |  |  | NA | NA | NA | Non-codel | WT | Methylated | NA | 52 | NA | LGm4 | NA | Classic-like |
| TCGA-4W-AA9S-01A | TCGA-4W-AA9S | 69 | 0:LIVING | Male | Primary | Glioblastoma G4 | Glioblastoma G4 |  |  | NA | NA | NA | Non-codel | WT | Methylated | NA | 29 | NA | LGm4 | NA | Classic-like |
| TCGA-4W-AA9T-01A | TCGA-4W-AA9T | 54 | 0:LIVING | Female | Primary | Glioblastoma G4 | Glioblastoma G4 |  |  | NA | NA | NA | Non-codel | WT | Methylated | NA | 40 | NA | LGm4 | NA | Classic-like |
| TCGA-74-6573-01A | TCGA-74-6573 | 67 | 1:DECEASED | Male | Primary | Glioblastoma G4 | Glioblastoma G4 |  |  | NA | NA | Gain chr 7 & loss chr 10 | Non-codel | WT | Unmethylated | NA | 33 | NA | LGm5 | NA | Mesenchymal-like |
| TCGA-74-6575-01A | TCGA-74-6575 | 73 | 0:LIVING | Female | Primary | Glioblastoma G4 | Glioblastoma G4 |  |  | NA | NA | Gain chr 7 & loss chr 10 | Non-codel | WT | Unmethylated | NA | 57 | NA | LGm5 | NA | Mesenchymal-like |
| TCGA-74-6577-01A | TCGA-74-6577 | 51 | 0:LIVING | Male | Primary | Glioblastoma G4 | Glioblastoma G4 |  |  | NA | NA | Gain chr 7 & loss chr 10 | Non-codel | WT | Unmethylated | NA | 31 | NA | LGm5 | NA | Mesenchymal-like |
| TCGA-74-6578-01A | TCGA-74-6578 | 58 | 0:LIVING | Male | Primary | Glioblastoma G4 | Glioblastoma G4 |  |  | NA | NA | Gain chr 7 & loss chr 10 | Non-codel | WT | Methylated | NA | 50 | NA | LGm5 | NA | Classic-like |
| TCGA-74-6581-01A | TCGA-74-6581 | 77 | 0:LIVING | Male | Primary | Glioblastoma G4 | Glioma NOS |  |  | NA | NA | Gain chr 7 & loss chr 10 | Non-codel | NA | Unmethylated | NA | NA | NA | LGm4 | NA | NA |
| TCGA-74-6584-01A | TCGA-74-6584 | 55 | 0:LIVING | Female | Primary | Glioblastoma G4 | Glioblastoma G4 |  |  | NA | NA | Gain chr 7 & loss chr 10 | Non-codel | WT | Unmethylated | NA | 27 | NA | LGm5 | NA | Mesenchymal-like |
| TCGA-76-4925-01A | TCGA-76-4925 | 76 | 1:DECEASED | Male | Primary | Glioblastoma G4 | Glioblastoma G4 |  |  | DDRm1 | DDRr4 | Gain chr 7 & loss chr 10 | Non-codel | WT | Methylated | NA | 50 | Proneural | LGm4 | LGr4 | Classic-like |
| TCGA-76-4926-01B | TCGA-76-4926 | 68 | 1:DECEASED | Male | Primary | Glioblastoma G4 | Glioblastoma G4 |  |  | DDRm1 | DDRr4 | Gain chr 7 & loss chr 10 | Non-codel | WT | Unmethylated | NA | 45 | Classical | LGm5 | LGr4 | Mesenchymal-like |
| TCGA-76-4927-01A | TCGA-76-4927 | 58 | 1:DECEASED | Male | Primary | Glioblastoma G4 | Glioblastoma G4 |  |  | DDRm1 | DDRr4 | NA | NA | WT | Unmethylated | NA | 33 | Neural | LGm4 | LGr4 | Classic-like |
| TCGA-76-4928-01B | TCGA-76-4928 | 85 | 1:DECEASED | Female | Primary | Glioblastoma G4 | Glioblastoma G4 |  |  | DDRm1 | DDRr4 | Gain chr 7 & loss chr 10 | Non-codel | WT | Methylated | NA | 51 | Classical | LGm5 | LGr4 | Mesenchymal-like |
| TCGA-76-4929-01A | TCGA-76-4929 | 76 | 1:DECEASED | Female | Primary | Glioblastoma G4 | Glioblastoma G4 |  |  | DDRm1 | DDRr4 | Gain chr 7 & loss chr 10 | Non-codel | WT | Methylated | NA | 43 | Neural | LGm4 | LGr4 | Classic-like |
| TCGA-76-4931-01A | TCGA-76-4931 | 70 | 1:DECEASED | Female | Primary | Glioblastoma G4 | Glioblastoma G4 |  |  | DDRm1 | DDRr4 | Gain chr 7 & loss chr 10 | Non-codel | WT | Unmethylated | NA | 31 | Classical | LGm5 | LGr4 | Mesenchymal-like |
| TCGA-76-4932-01A | TCGA-76-4932 | 50 | 1:DECEASED | Female | Primary | Glioblastoma G4 | Glioblastoma G4 |  |  | DDRm1 | DDRr4 | NA | NA | WT | Methylated | NA | 44 | Proneural | LGm4 | LGr4 | Classic-like |
| TCGA-76-4934-01A | TCGA-76-4934 | 66 | 1:DECEASED | Female | Primary | Glioblastoma G4 | Glioblastoma G4 |  |  | NA | NA | No combined CNA | Non-codel | WT | Methylated | NA | 41 | Proneural | LGm6 | LGr1 | Mesenchymal-like |
| TCGA-76-4935-01A | TCGA-76-4935 | 52 | 0:LIVING | Female | Primary | Glioblastoma G4 | Glioblastoma G4 |  |  | NA | NA | Gain chr 7 & loss chr 10 | Non-codel | WT | Methylated | NA | 32 | Proneural | LGm5 | LGr1 | Mesenchymal-like |
| TCGA-76-6191-01A | TCGA-76-6191 | 57 | 1:DECEASED | Male | Primary | Glioblastoma G4 | Glioblastoma G4 |  |  | NA | NA | Gain chr 7 & loss chr 10 | Non-codel | WT | Unmethylated | NA | 45 | Proneural | LGm5 | LGr1 | Mesenchymal-like |
| TCGA-76-6192-01A | TCGA-76-6192 | 74 | 1:DECEASED | Male | Primary | Glioblastoma G4 | Glioblastoma G4 |  |  | NA | NA | Gain chr 7 & loss chr 10 | Non-codel | WT | Unmethylated | NA | 36 | Proneural | LGm5 | LGr4 | Mesenchymal-like |
| TCGA-76-6193-01A | TCGA-76-6193 | 78 | 1:DECEASED | Male | Primary | Glioblastoma G4 | Glioblastoma G4 |  |  | NA | NA | No combined CNA | Non-codel | WT | Unmethylated | NA | 32 | Mesenchymal | LGm5 | LGr4 | Mesenchymal-like |
| TCGA-76-6280-01A | TCGA-76-6280 | 57 | 1:DECEASED | Male | Primary | Glioblastoma G4 | Glioblastoma G4 |  |  | NA | NA | Gain chr 7 & loss chr 10 | Non-codel | WT | Methylated | NA | 41 | NA | LGm4 | NA | Classic-like |
| TCGA-76-6282-01A | TCGA-76-6282 | 63 | 1:DECEASED | Male | Primary | Glioblastoma G4 | Glioblastoma G4 |  |  | NA | NA | Gain chr 7 & loss chr 10 | Non-codel | WT | Unmethylated | NA | 35 | Mesenchymal | LGm5 | LGr4 | Mesenchymal-like |
| TCGA-76-6283-01A | TCGA-76-6283 | NA | NA | NA | Primary | Glioblastoma G4 | Glioblastoma G4 |  |  | NA | NA | Gain chr 7 & loss chr 10 | Non-codel | WT | Methylated | NA | 71 | NA | LGm4 | NA | Classic-like |
| TCGA-76-6285-01A | TCGA-76-6285 | 64 | 1:DECEASED | Female | Primary | Glioblastoma G4 | Glioblastoma G4 |  |  | NA | NA | Gain chr 7 & loss chr 10 | Non-codel | WT | Unmethylated | NA | 42 | Proneural | LGm5 | LGr1 | Mesenchymal-like |
| TCGA-76-6286-01A | TCGA-76-6286 | 60 | 1:DECEASED | Male | Primary | Glioblastoma G4 | Glioblastoma G4 |  |  | NA | NA | Gain chr 7 & loss chr 10 | Non-codel | WT | Unmethylated | NA | 47 | NA | LGm4 | NA | Classic-like |
| TCGA-76-6656-01A | TCGA-76-6656 | 66 | 1:DECEASED | Male | Primary | Glioblastoma G4 | Glioblastoma G4 |  |  | NA | NA | No combined CNA | Non-codel | WT | Methylated | NA | 73 | NA | LGm4 | NA | Classic-like |
| TCGA-76-6657-01A | TCGA-76-6657 | 74 | 1:DECEASED | Male | Primary | Glioblastoma G4 | Glioblastoma G4 |  |  | NA | NA | Gain chr 7 & loss chr 10 | Non-codel | WT | Methylated | NA | 45 | NA | LGm4 | NA | Classic-like |
| TCGA-76-6660-01A | TCGA-76-6660 | NA | NA | NA | Primary | Glioblastoma G4 | Glioblastoma G4 |  |  | NA | NA | Gain chr 7 & loss chr 10 | Non-codel | WT | Unmethylated | NA | 54 | NA | LGm4 | NA | Classic-like |
| TCGA-76-6661-01B | TCGA-76-6661 | 54 | 0:LIVING | Male | Primary | Glioblastoma G4 | Glioblastoma G4 |  |  | NA | NA | Gain chr 7 & loss chr 10 | Non-codel | WT | Unmethylated | NA | 40 | NA | LGm4 | NA | Classic-like |
| TCGA-76-6662-01A | TCGA-76-6662 | 58 | 0:LIVING | Male | Primary | Glioblastoma G4 | Glioblastoma G4 |  |  | NA | NA | Gain chr 7 & loss chr 10 | Non-codel | WT | Unmethylated | NA | 31 | NA | LGm4 | NA | Classic-like |
| TCGA-76-6663-01A | TCGA-76-6663 | 44 | 0:LIVING | Female | Primary | Glioblastoma G4 | Glioblastoma G4 |  |  | NA | NA | Gain chr 7 & loss chr 10 | Non-codel | WT | Unmethylated | NA | 35 | NA | LGm4 | NA | Classic-like |
| TCGA-76-6664-01A | TCGA-76-6664 | 49 | 0:LIVING | Female | Primary | Glioblastoma G4 | Glioblastoma G4 |  |  | NA | NA | Gain chr 7 & loss chr 10 | Non-codel | WT | Methylated | NA | 39 | NA | LGm5 | NA | Mesenchymal-like |
| TCGA-81-5910-01A | TCGA-81-5910 | 64 | 1:DECEASED | Male | Primary | Glioblastoma G4 | Glioblastoma G4 |  |  | NA | NA | Gain chr 7 & loss chr 10 | Non-codel | WT | Unmethylated | NA | 34 | Classical | LGm5 | LGr4 | Mesenchymal-like |
| TCGA-81-5911-01A | TCGA-81-5911 | 33 | 0:LIVING | Male | Primary | Glioblastoma G4 | Glioblastoma G4 |  |  | NA | NA | Gain chr 7 & loss chr 10 | Non-codel | WT | Unmethylated | NA | 24 | NA | LGm4 | NA | Classic-like |
| TCGA-87-5896-01A | TCGA-87-5896 | 50 | 0:LIVING | Female | Primary | Glioblastoma G4 | Glioblastoma G4 |  |  | NA | NA | Gain chr 7 & loss chr 10 | Non-codel | WT | Unmethylated | NA | 36 | Classical | LGm4 | LGr4 | Classic-like |
| TCGA-CS-4938-01B | TCGA-CS-4938 | 31 | 0:LIVING | Female | Primary | Astrocytoma G2 | Astrocytoma, IDH-mutant lower grade |  |  | DDRm2 | DDRr1 | No combined CNA | Non-codel | Mutant | Unmethylated | WT | 11 | IDHmut-non-codel | LGm2 | LGr3 | G-CIMP-high |
| TCGA-CS-4941-01A | TCGA-CS-4941 | 67 | 1:DECEASED | Male | Primary | Astrocytoma G3 | Glioblastoma G4 | EGFR amp, +7/-10, TERTp mut |  | DDRm1 | DDRr4 | Gain chr 7 & loss chr 10 | Non-codel | WT | Methylated | Mutant | 42 | IDHwt | LGm5 | LGr4 | Mesenchymal-like |
| TCGA-CS-4942-01A | TCGA-CS-4942 | 44 | 1:DECEASED | Female | Primary | Astrocytoma G3 | Astrocytoma, IDH-mutant lower grade |  |  | DDRm2 | DDRr1 | No combined CNA | Non-codel | Mutant | Unmethylated | WT | 18 | IDHmut-non-codel | LGm2 | LGr3 | G-CIMP-high |
| TCGA-CS-4943-01A | TCGA-CS-4943 | 37 | 0:LIVING | Male | Primary | Astrocytoma G3 | Astrocytoma, IDH-mutant G4 | CDKN2A/Bdel |  | DDRm2 | DDRr4 | No combined CNA | Non-codel | Mutant | Methylated | WT | 20 | IDHmut-non-codel | LGm2 | LGr3 | G-CIMP-high |
| TCGA-CS-4944-01A | TCGA-CS-4944 | 50 | 0:LIVING | Male | Primary | Astrocytoma G2 | Astrocytoma, IDH-mutant lower grade |  |  | DDRm2 | DDRr1 | No combined CNA | Non-codel | Mutant | Methylated | Mutant | 17 | IDHmut-non-codel | LGm2 | LGr3 | G-CIMP-high |
| TCGA-CS-5390-01A | TCGA-CS-5390 | 47 | 0:LIVING | Female | Primary | Oligodendroglioma G2 | Oligodendroglioma, IDH-mutant |  |  | DDRm2 | DDRr2 | No combined CNA | Codel | Mutant | Methylated | Mutant | 27 | IDHmut-codel | LGm3 | LGr1 | Codel |
| TCGA-CS-5393-01A | TCGA-CS-5393 | 39 | 0:LIVING | Male | Primary | Astrocytoma G3 | Astrocytoma, IDH-mutant lower grade |  |  | DDRm2 | DDRr1 | No combined CNA | Non-codel | Mutant | Methylated | WT | 20 | IDHmut-non-codel | LGm2 | LGr3 | G-CIMP-high |
| TCGA-CS-5394-01A | TCGA-CS-5394 | 40 | 0:LIVING | Male | Primary | Astrocytoma G3 | Astrocytoma, IDH-mutant lower grade |  |  | DDRm2 | DDRr2 | No combined CNA | Non-codel | Mutant | Methylated | WT | 19 | IDHmut-non-codel | LGm3 | LGr1 | Codel |
| TCGA-CS-5395-01A | TCGA-CS-5395 | 43 | 1:DECEASED | Male | Primary | Oligodendroglioma G2 | Glioblastoma G4 | +7/-10, TERTp mut |  | DDRm1 | DDRr4 | Gain chr 7 & loss chr 10 | Non-codel | WT | Unmethylated | Mutant | 34 | IDHwt | LGm5 | LGr4 | Mesenchymal-like |
| TCGA-CS-5396-01A | TCGA-CS-5396 | 53 | 0:LIVING | Female | Primary | Oligodendroglioma G3 | Oligodendroglioma, IDH-mutant |  |  | DDRm2 | DDRr2 | No combined CNA | Codel | Mutant | Methylated | Mutant | 21 | IDHmut-codel | LGm3 | LGr1 | Codel |
| TCGA-CS-5397-01A | TCGA-CS-5397 | 54 | 1:DECEASED | Female | Primary | Astrocytoma G3 | Glioblastoma G4 | +7/-10, TERTp mut |  | DDRm1 | DDRr4 | Gain chr 7 & loss chr 10 | Non-codel | WT | Unmethylated | Mutant | 27 | IDHwt | LGm5 | LGr4 | Mesenchymal-like |
| TCGA-CS-6186-01A | TCGA-CS-6186 | 58 | 1:DECEASED | Male | Primary | Oligoastrocytoma G3 | Glioblastoma G4 | +7/-10, TERTp mut |  | DDRm1 | DDRr4 | Gain chr 7 & loss chr 10 | Non-codel | WT | Unmethylated | Mutant | 43 | IDHwt | LGm4 | LGr4 | Classic-like |
| TCGA-CS-6188-01A | TCGA-CS-6188 | 48 | 0:LIVING | Male | Primary | Astrocytoma G3 | Glioblastoma G4 | EGFR amp, +7/-10, TERTp mut |  | DDRm1 | DDRr4 | Gain chr 7 & loss chr 10 | Non-codel | WT | Unmethylated | Mutant | 31 | IDHwt | LGm4 | LGr4 | Mesenchymal-like |
| TCGA-CS-6290-01A | TCGA-CS-6290 | 31 | 0:LIVING | Male | Primary | Astrocytoma G3 | Astrocytoma, IDH-mutant lower grade |  |  | DDRm2 | DDRr1 | No combined CNA | Non-codel | Mutant | Methylated | WT | 11 | IDHmut-non-codel | LGm2 | LGr3 | G-CIMP-high |
| TCGA-CS-6665-01A | TCGA-CS-6665 | 51 | 0:LIVING | Female | Primary | Astrocytoma G3 | Astrocytoma, IDH-mutant lower grade |  |  | NA | NA | No combined CNA | Non-codel | Mutant | Methylated | WT | 54 | IDHmut-non-codel | LGm1 | LGr3 | G-CIMP-low |
| TCGA-CS-6666-01A | TCGA-CS-6666 | 22 | 0:LIVING | Male | Primary | Astrocytoma G3 | Astrocytoma, IDH-mutant lower grade |  |  | DDRm2 | DDRr1 | No combined CNA | Non-codel | Mutant | Methylated | WT | 16 | IDHmut-non-codel | LGm2 | LGr3 | G-CIMP-high |
| TCGA-CS-6667-01A | TCGA-CS-6667 | 39 | 0:LIVING | Female | Primary | Astrocytoma G2 | Astrocytoma, IDH-mutant lower grade |  |  | DDRm2 | DDRr1 | No combined CNA | Non-codel | Mutant | Methylated | WT | 16 | IDHmut-non-codel | LGm2 | LGr3 | G-CIMP-high |
| TCGA-CS-6668-01A | TCGA-CS-6668 | 57 | 0:LIVING | Female | Primary | Oligodendroglioma G2 | Oligodendroglioma, IDH-mutant |  |  | DDRm2 | DDRr2 | No combined CNA | Codel | Mutant | Methylated | Mutant | 17 | IDHmut-codel | LGm3 | LGr1 | Codel |
| TCGA-CS-6669-01A | TCGA-CS-6669 | 26 | 0:LIVING | Female | Primary | Oligodendroglioma G2 | Glioma, IDH-wildtype NEC |  |  | DDRm1 | DDRr3 | No combined CNA | Non-codel | WT | Unmethylated | WT | NA | IDHwt | LGm6 | LGr2 | PA-like |
| TCGA-CS-6670-01A | TCGA-CS-6670 | 43 | 0:LIVING | Male | Primary | Oligodendroglioma G3 | Oligodendroglioma, IDH-mutant |  |  | DDRm2 | DDRr2 | No combined CNA | Codel | Mutant | Methylated | NA | 25 | IDHmut-codel | LGm3 | LGr2 | Codel |
| TCGA-DB-5270-01A | TCGA-DB-5270 | 38 | 0:LIVING | Female | Primary | Oligoastrocytoma G3 | Astrocytoma, IDH-mutant lower grade |  |  | DDRm2 | DDRr3 | No combined CNA | Non-codel | Mutant | Methylated | NA | 11 | IDHmut-non-codel | LGm2 | LGr2 | G-CIMP-high |
| TCGA-DB-5273-01A | TCGA-DB-5273 | 33 | 0:LIVING | Male | Primary | Astrocytoma G3 | Astrocytoma, IDH-mutant lower grade |  |  | DDRm2 | DDRr1 | No combined CNA | Non-codel | Mutant | Unmethylated | WT | 9 | IDHmut-non-codel | LGm2 | LGr3 | G-CIMP-high |
| TCGA-DB-5274-01A | TCGA-DB-5274 | 37 | 0:LIVING | Female | Primary | Oligoastrocytoma G3 | Oligodendroglioma, IDH-mutant |  |  | DDRm2 | DDRr1 | No combined CNA | Codel | Mutant | Methylated | Mutant | 25 | IDHmut-codel | LGm3 | LGr1 | Codel |
| TCGA-DB-5275-01A | TCGA-DB-5275 | 36 | 0:LIVING | Male | Primary | Oligoastrocytoma G3 | Astrocytoma, IDH-mutant lower grade |  |  | DDRm2 | DDRr1 | No combined CNA | Non-codel | Mutant | Methylated | WT | 27 | IDHmut-non-codel | LGm2 | LGr3 | G-CIMP-high |
| TCGA-DB-5276-01A | TCGA-DB-5276 | 32 | 0:LIVING | Male | Primary | Oligoastrocytoma G3 | Astrocytoma, IDH-mutant lower grade |  |  | DDRm2 | DDRr1 | No combined CNA | Non-codel | Mutant | Methylated | WT | 9 | IDHmut-non-codel | LGm2 | LGr3 | G-CIMP-high |
| TCGA-DB-5277-01A | TCGA-DB-5277 | 34 | 1:DECEASED | Male | Primary | Astrocytoma G3 | Astrocytoma, IDH-mutant lower grade |  |  | DDRm2 | DDRr1 | No combined CNA | Non-codel | Mutant | Methylated | WT | 29 | IDHmut-non-codel | LGm1 | LGr3 | G-CIMP-high |
| TCGA-DB-5278-01A | TCGA-DB-5278 | 17 | 0:LIVING | Male | Primary | Oligodendroglioma G2 | Oligodendroglioma, IDH-mutant |  |  | DDRm2 | DDRr3 | No combined CNA | Codel | Mutant | Methylated | WT | 7 | IDHmut-codel | LGm2 | LGr2 | Codel |
| TCGA-DB-5279-01A | TCGA-DB-5279 | 59 | 0:LIVING | Male | Primary | Oligodendroglioma G2 | Oligodendroglioma, IDH-mutant |  |  | DDRm2 | DDRr2 | No combined CNA | Codel | Mutant | Methylated | Mutant | 28 | IDHmut-codel | LGm3 | LGr3 | Codel |
| TCGA-DB-5280-01A | TCGA-DB-5280 | 43 | 0:LIVING | Male | Primary | Oligoastrocytoma G2 | Astrocytoma, IDH-mutant lower grade |  |  | DDRm2 | DDRr1 | No combined CNA | Non-codel | Mutant | Methylated | WT | 16 | IDHmut-non-codel | LGm2 | LGr3 | G-CIMP-high |
| TCGA-DB-5281-01A | TCGA-DB-5281 | 61 | 0:LIVING | Male | Primary | Oligoastrocytoma G3 | Astrocytoma, IDH-mutant lower grade |  |  | DDRm2 | DDRr1 | No combined CNA | Non-codel | Mutant | Methylated | WT | 41 | IDHmut-non-codel | LGm2 | LGr3 | G-CIMP-high |
| TCGA-DB-A4X9-01A | TCGA-DB-A4X9 | 33 | 0:LIVING | Female | Primary | Oligoastrocytoma G2 | Astrocytoma, IDH-mutant lower grade |  |  | DDRm2 | DDRr1 | No combined CNA | Non-codel | Mutant | Methylated | WT | 20 | IDHmut-non-codel | LGm2 | LGr3 | G-CIMP-high |
| TCGA-DB-A4XA-01A | TCGA-DB-A4XA | 30 | 0:LIVING | Male | Primary | Oligoastrocytoma G2 | Oligodendroglioma, IDH-mutant |  |  | DDRm2 | DDRr2 | No combined CNA | Codel | Mutant | Methylated | Mutant | 7 | IDHmut-codel | LGm2 | LGr1 | Codel |
| TCGA-DB-A4XB-01A | TCGA-DB-A4XB | 38 | 0:LIVING | Male | Primary | Astrocytoma G3 | Astrocytoma, IDH-mutant lower grade |  |  | DDRm2 | DDRr1 | No combined CNA | Non-codel | Mutant | Methylated | WT | 26 | IDHmut-non-codel | LGm1 | LGr3 | G-CIMP-high |
| TCGA-DB-A4XC-01A | TCGA-DB-A4XC | 26 | 0:LIVING | Male | Primary | Oligoastrocytoma G2 | Astrocytoma, IDH-mutant lower grade |  |  | DDRm2 | DDRr1 | No combined CNA | Non-codel | Mutant | Methylated | WT | 13 | IDHmut-non-codel | LGm2 | LGr3 | G-CIMP-high |
| TCGA-DB-A4XD-01A | TCGA-DB-A4XD | 32 | 0:LIVING | Male | Primary | Astrocytoma G3 | Astrocytoma, IDH-mutant lower grade |  |  | DDRm2 | DDRr1 | No combined CNA | Non-codel | Mutant | Methylated | WT | 18 | IDHmut-non-codel | LGm2 | LGr3 | G-CIMP-high |
| TCGA-DB-A4XE-01A | TCGA-DB-A4XE | 27 | 0:LIVING | Female | Primary | Oligoastrocytoma G3 | Astrocytoma, IDH-mutant lower grade |  |  | DDRm2 | DDRr1 | No combined CNA | Non-codel | Mutant | Methylated | WT | 17 | IDHmut-non-codel | LGm2 | LGr3 | G-CIMP-high |
| TCGA-DB-A4XF-01A | TCGA-DB-A4XF | 41 | 0:LIVING | Female | Primary | Astrocytoma G3 | Astrocytoma, IDH-mutant lower grade |  |  | DDRm2 | DDRr1 | No combined CNA | Non-codel | Mutant | Methylated | WT | 17 | IDHmut-non-codel | LGm2 | LGr3 | G-CIMP-high |
| TCGA-DB-A4XG-01A | TCGA-DB-A4XG | 34 | 0:LIVING | Male | Primary | Oligodendroglioma G3 | Oligodendroglioma, IDH-mutant |  |  | DDRm2 | DDRr2 | No combined CNA | Codel | Mutant | Methylated | Mutant | 15 | IDHmut-codel | LGm3 | LGr1 | Codel |
| TCGA-DB-A4XH-01A | TCGA-DB-A4XH | 53 | 0:LIVING | Female | Primary | Oligoastrocytoma G2 | Oligodendroglioma, IDH-mutant |  |  | DDRm2 | DDRr3 | No combined CNA | Codel | Mutant | Methylated | Mutant | 23 | IDHmut-codel | LGm3 | LGr2 | Codel |
| TCGA-DB-A64L-01A | TCGA-DB-A64L | 67 | 0:LIVING | Female | Primary | Oligodendroglioma G2 | Oligodendroglioma, IDH-mutant |  |  | DDRm2 | DDRr2 | No combined CNA | Codel | Mutant | Methylated | Mutant | 44 | IDHmut-codel | LGm3 | LGr2 | Codel |
| TCGA-DB-A64O-01A | TCGA-DB-A64O | 59 | 0:LIVING | Male | Primary | Oligoastrocytoma G2 | Glioblastoma G4 | +7/-10, TERTp mut |  | DDRm1 | DDRr1 | Gain chr 7 & loss chr 10 | Non-codel | WT | Unmethylated | Mutant | 16 | IDHwt | LGm5 | LGr4 | Mesenchymal-like |
| TCGA-DB-A64P-01A | TCGA-DB-A64P | 40 | 0:LIVING | Male | Primary | Oligodendroglioma G3 | Oligodendroglioma, IDH-mutant |  |  | DDRm2 | DDRr2 | No combined CNA | Codel | Mutant | Methylated | Mutant | 18 | IDHmut-codel | LGm3 | LGr1 | Codel |
| TCGA-DB-A64Q-01A | TCGA-DB-A64Q | 31 | 0:LIVING | Female | Primary | Oligoastrocytoma G2 | Oligodendroglioma, IDH-mutant |  |  | DDRm2 | DDRr2 | No combined CNA | Codel | Mutant | Methylated | Mutant | 14 | IDHmut-codel | LGm3 | LGr1 | Codel |
| TCGA-DB-A64R-01A | TCGA-DB-A64R | 24 | 0:LIVING | Female | Primary | Oligodendroglioma G2 | Oligodendroglioma, IDH-mutant |  |  | DDRm2 | DDRr2 | No combined CNA | Codel | Mutant | Methylated | Mutant | 11 | IDHmut-codel | LGm2 | LGr1 | Codel |
| TCGA-DB-A64S-01A | TCGA-DB-A64S | 20 | 0:LIVING | Male | Primary | Oligoastrocytoma G2 | Astrocytoma, IDH-mutant lower grade |  |  | DDRm2 | DDRr1 | No combined CNA | Non-codel | Mutant | Unmethylated | WT | 8 | IDHmut-non-codel | LGm2 | LGr3 | G-CIMP-high |
| TCGA-DB-A64U-01A | TCGA-DB-A64U | 38 | 0:LIVING | Female | Primary | Oligoastrocytoma G2 | Oligodendroglioma, IDH-mutant |  |  | DDRm2 | DDRr2 | No combined CNA | Codel | Mutant | Methylated | Mutant | 6 | IDHmut-codel | LGm2 | LGr1 | Codel |
| TCGA-DB-A64V-01A | TCGA-DB-A64V | 54 | 0:LIVING | Male | Primary | Oligodendroglioma G2 | Oligodendroglioma, IDH-mutant |  |  | DDRm2 | DDRr1 | No combined CNA | Codel | Mutant | Methylated | Mutant | 18 | IDHmut-codel | LGm3 | LGr3 | Codel |
| TCGA-DB-A64W-01A | TCGA-DB-A64W | 65 | 0:LIVING | Female | Primary | Oligoastrocytoma G3 | Oligodendroglioma, IDH-mutant |  |  | DDRm2 | DDRr2 | No combined CNA | Codel | Mutant | Methylated | Mutant | 36 | IDHmut-codel | LGm3 | LGr1 | Codel |
| TCGA-DB-A64X-01A | TCGA-DB-A64X | 56 | 0:LIVING | Female | Primary | Astrocytoma G3 | Astrocytoma, IDH-mutant lower grade |  |  | DDRm2 | DDRr1 | No combined CNA | Non-codel | Mutant | Methylated | WT | 45 | IDHmut-non-codel | LGm2 | LGr3 | G-CIMP-high |
| TCGA-DB-A75K-01A | TCGA-DB-A75K | 55 | 0:LIVING | Female | Primary | Oligoastrocytoma G3 | Oligodendroglioma, IDH-mutant |  |  | DDRm2 | DDRr2 | No combined CNA | Codel | Mutant | Methylated | NA | 29 | IDHmut-codel | LGm3 | LGr1 | Codel |
| TCGA-DB-A75L-01A | TCGA-DB-A75L | 36 | 0:LIVING | Female | Primary | Astrocytoma G3 | Astrocytoma, IDH-mutant G4 | CDKN2A/Bdel |  | DDRm2 | DDRr1 | No combined CNA | Non-codel | Mutant | Methylated | NA | 29 | IDHmut-non-codel | LGm2 | LGr3 | G-CIMP-high |
| TCGA-DB-A75M-01A | TCGA-DB-A75M | 47 | 0:LIVING | Male | Primary | Astrocytoma G2 | Astrocytoma, IDH-mutant lower grade |  |  | DDRm2 | DDRr1 | No combined CNA | Non-codel | Mutant | Methylated | NA | 20 | IDHmut-non-codel | LGm2 | LGr3 | G-CIMP-high |
| TCGA-DB-A75O-01A | TCGA-DB-A75O | 29 | 0:LIVING | Male | Primary | Astrocytoma G3 | Astrocytoma, IDH-mutant lower grade |  |  | DDRm2 | DDRr1 | No combined CNA | Non-codel | Mutant | Methylated | NA | 13 | IDHmut-non-codel | LGm2 | LGr3 | G-CIMP-high |
| TCGA-DB-A75P-01A | TCGA-DB-A75P | 25 | 0:LIVING | Female | Primary | Astrocytoma G2 | Glioma, IDH-wildtype NOS |  |  | DDRm1 | DDRr1 | No combined CNA | Non-codel | WT | Unmethylated | NA | 2 | IDHwt | LGm6 | LGr2 | PA-like |
| TCGA-DH-5140-01A | TCGA-DH-5140 | 38 | 1:DECEASED | Female | Primary | Oligoastrocytoma G3 | Glioma, IDH-wildtype NEC |  |  | DDRm1 | DDRr4 | No combined CNA | Non-codel | WT | Unmethylated | WT | 19 | IDHwt | LGm6 | LGr1 | PA-like |
| TCGA-DH-5141-01A | TCGA-DH-5141 | 32 | 0:LIVING | Male | Primary | Oligodendroglioma G3 | Oligodendroglioma, IDH-mutant |  |  | DDRm2 | DDRr2 | No combined CNA | Codel | Mutant | Methylated | Mutant | 16 | IDHmut-codel | LGm3 | LGr1 | Codel |
| TCGA-DH-5142-01A | TCGA-DH-5142 | 29 | 0:LIVING | Male | Primary | Astrocytoma G3 | Astrocytoma, IDH-mutant lower grade |  |  | DDRm2 | DDRr1 | No combined CNA | Non-codel | Mutant | Methylated | WT | 17 | IDHmut-non-codel | LGm1 | LGr3 | G-CIMP-high |
| TCGA-DH-5143-01A | TCGA-DH-5143 | 30 | 0:LIVING | Male | Primary | Oligoastrocytoma G3 | Astrocytoma, IDH-mutant lower grade |  |  | DDRm2 | DDRr1 | No combined CNA | Non-codel | Mutant | Methylated | WT | 21 | IDHmut-non-codel | LGm2 | LGr3 | G-CIMP-high |
| TCGA-DH-5144-01A | TCGA-DH-5144 | 56 | 0:LIVING | Female | Primary | Oligodendroglioma G3 | Oligodendroglioma, IDH-mutant |  |  | DDRm2 | DDRr2 | No combined CNA | Codel | Mutant | Methylated | Mutant | 21 | IDHmut-codel | LGm3 | LGr1 | Codel |
| TCGA-DH-A669-01A | TCGA-DH-A669 | 70 | 1:DECEASED | Male | Primary | Oligodendroglioma G3 | Oligodendroglioma, IDH-mutant |  |  | DDRm2 | DDRr4 | No combined CNA | Codel | Mutant | Methylated | Mutant | 43 | IDHmut-codel | LGm3 | LGr1 | Codel |
| TCGA-DH-A669-02A | TCGA-DH-A669 | NA | NA | NA | Recurrent | Oligodendroglioma G3 | Oligodendroglioma, IDH-mutant |  |  | DDRm2 | DDRr1 | NA | NA | NA | NA | NA | NA | NA | NA | NA | NA |
| TCGA-DH-A66B-01A | TCGA-DH-A66B | 52 | 0:LIVING | Male | Primary | Astrocytoma G3 | Astrocytoma, IDH-mutant lower grade |  |  | DDRm2 | DDRr1 | No combined CNA | Non-codel | Mutant | Methylated | WT | 32 | IDHmut-non-codel | LGm2 | LGr3 | G-CIMP-high |
| TCGA-DH-A66D-01A | TCGA-DH-A66D | 43 | 0:LIVING | Female | Primary | Astrocytoma G3 | Astrocytoma, IDH-mutant lower grade |  |  | DDRm2 | DDRr1 | No combined CNA | Non-codel | Mutant | Methylated | NA | 31 | IDHmut-non-codel | LGm2 | LGr3 | G-CIMP-high |
| TCGA-DH-A66F-01A | TCGA-DH-A66F | 49 | 0:LIVING | Male | Primary | Oligodendroglioma G2 | Oligodendroglioma, IDH-mutant |  |  | DDRm2 | DDRr2 | No combined CNA | Codel | Mutant | Methylated | Mutant | 13 | IDHmut-codel | LGm3 | LGr1 | Codel |
| TCGA-DH-A66G-01A | TCGA-DH-A66G | 49 | 0:LIVING | Female | Primary | Oligodendroglioma G3 | Astrocytoma, IDH-mutant lower grade |  |  | DDRm2 | DDRr3 | No combined CNA | Non-codel | Mutant | Methylated | NA | 17 | IDHmut-non-codel | LGm2 | NA | G-CIMP-high |
| TCGA-DH-A7UR-01A | TCGA-DH-A7UR | 59 | 0:LIVING | Female | Primary | Oligodendroglioma G3 | Oligodendroglioma, IDH-mutant |  |  | DDRm2 | DDRr2 | No combined CNA | Codel | Mutant | Methylated | NA | 37 | IDHmut-codel | LGm3 | LGr1 | Codel |
| TCGA-DH-A7US-01A | TCGA-DH-A7US | 50 | 0:LIVING | Male | Primary | Oligodendroglioma G2 | Oligodendroglioma, IDH-mutant |  |  | DDRm2 | DDRr2 | No combined CNA | Codel | Mutant | Methylated | NA | 18 | IDHmut-codel | LGm2 | LGr1 | Codel |
| TCGA-DH-A7UT-01A | TCGA-DH-A7UT | 30 | 0:LIVING | Male | Primary | Astrocytoma G3 | Astrocytoma, IDH-mutant lower grade |  |  | DDRm2 | DDRr1 | No combined CNA | Non-codel | Mutant | Methylated | NA | 13 | IDHmut-non-codel | LGm1 | LGr3 | G-CIMP-high |
| TCGA-DH-A7UU-01A | TCGA-DH-A7UU | 43 | 0:LIVING | Male | Primary | Astrocytoma G3 | Astrocytoma, IDH-mutant lower grade |  |  | DDRm2 | DDRr1 | No combined CNA | Non-codel | Mutant | Methylated | NA | 33 | IDHmut-non-codel | LGm2 | LGr3 | G-CIMP-high |
| TCGA-DH-A7UV-01A | TCGA-DH-A7UV | 49 | 0:LIVING | Male | Primary | Astrocytoma G3 | Astrocytoma, IDH-mutant lower grade |  |  | DDRm2 | DDRr1 | No combined CNA | Non-codel | Mutant | Methylated | NA | 27 | IDHmut-non-codel | LGm2 | LGr3 | G-CIMP-high |
| TCGA-DU-5847-01A | TCGA-DU-5847 | 34 | 0:LIVING | Female | Primary | Astrocytoma G3 | Glioblastoma G4 | TERTp mut |  | DDRm1 | DDRr4 | No combined CNA | Non-codel | WT | Methylated | Mutant | 32 | IDHwt | LGm5 | LGr4 | Mesenchymal-like |
| TCGA-DU-5849-01A | TCGA-DU-5849 | 48 | 0:LIVING | Male | Primary | Oligodendroglioma G2 | Oligodendroglioma, IDH-mutant |  |  | DDRm2 | DDRr3 | No combined CNA | Codel | Mutant | Methylated | Mutant | 19 | IDHmut-codel | LGm3 | LGr2 | Codel |
| TCGA-DU-5851-01A | TCGA-DU-5851 | 40 | 0:LIVING | Female | Primary | Oligoastrocytoma G3 | Astrocytoma, IDH-mutant lower grade |  |  | NA | NA | No combined CNA | Non-codel | Mutant | Unmethylated | WT | 18 | IDHmut-non-codel | LGm2 | LGr2 | G-CIMP-high |
| TCGA-DU-5852-01A | TCGA-DU-5852 | 61 | 1:DECEASED | Female | Primary | Oligoastrocytoma G3 | Glioblastoma G4 | EGFR amp, TERTp mut |  | DDRm1 | DDRr4 | No combined CNA | Non-codel | WT | Methylated | Mutant | 59 | IDHwt | LGm5 | LGr4 | Mesenchymal-like |
| TCGA-DU-5853-01A | TCGA-DU-5853 | 29 | 0:LIVING | Male | Primary | Oligoastrocytoma G2 | Astrocytoma, IDH-mutant lower grade |  |  | DDRm2 | DDRr1 | No combined CNA | Non-codel | Mutant | Methylated | WT | 12 | IDHmut-non-codel | LGm2 | LGr3 | G-CIMP-high |
| TCGA-DU-5854-01A | TCGA-DU-5854 | 57 | 0:LIVING | Female | Primary | Astrocytoma G3 | Glioblastoma G4 | EGFR amp, +7/-10, TERTp mut |  | DDRm1 | DDRr4 | Gain chr 7 & loss chr 10 | Non-codel | WT | Unmethylated | Mutant | 36 | IDHwt | LGm5 | LGr4 | Mesenchymal-like |
| TCGA-DU-5855-01A | TCGA-DU-5855 | 49 | 0:LIVING | Female | Primary | Oligoastrocytoma G3 | Astrocytoma, IDH-mutant lower grade |  |  | DDRm2 | DDRr1 | No combined CNA | Non-codel | Mutant | Methylated | WT | 31 | IDHmut-non-codel | LGm2 | LGr3 | G-CIMP-high |
| TCGA-DU-5870-01A | TCGA-DU-5870 | 34 | 0:LIVING | Female | Primary | Oligodendroglioma G2 | Oligodendroglioma, IDH-mutant |  |  | DDRm2 | DDRr2 | No combined CNA | Codel | Mutant | Methylated | Mutant | 11 | IDHmut-codel | LGm3 | LGr1 | Codel |
| TCGA-DU-5870-02A | TCGA-DU-5870 | NA | NA | NA | Recurrent | Oligodendroglioma G3 | Oligodendroglioma, IDH-mutant |  |  | DDRm2 | DDRr2 | NA | NA | NA | NA | NA | NA | NA | NA | NA | NA |
| TCGA-DU-5871-01A | TCGA-DU-5871 | 37 | 0:LIVING | Female | Primary | Oligoastrocytoma G2 | Astrocytoma, IDH-mutant lower grade |  |  | DDRm2 | DDRr1 | No combined CNA | Non-codel | Mutant | Methylated | WT | 20 | IDHmut-non-codel | LGm2 | LGr3 | G-CIMP-high |
| TCGA-DU-5872-01A | TCGA-DU-5872 | 43 | 0:LIVING | Female | Primary | Oligoastrocytoma G2 | Astrocytoma, IDH-mutant lower grade |  |  | DDRm2 | DDRr1 | No combined CNA | Non-codel | Mutant | Methylated | WT | 24 | IDHmut-non-codel | LGm2 | LGr3 | G-CIMP-high |
| TCGA-DU-5872-02A | TCGA-DU-5872 | NA | NA | NA | Recurrent | Oligoastrocytoma G2 | Astrocytoma, IDH-mutant lower grade |  |  | DDRm1 | DDRr1 | NA | NA | NA | NA | NA | NA | NA | NA | NA | NA |
| TCGA-DU-5874-01A | TCGA-DU-5874 | 62 | 0:LIVING | Female | Primary | Oligodendroglioma G2 | Oligodendroglioma, IDH-mutant |  |  | DDRm2 | DDRr2 | No combined CNA | Codel | Mutant | Methylated | Mutant | 32 | IDHmut-codel | LGm3 | LGr1 | Codel |
| TCGA-DU-6392-01A | TCGA-DU-6392 | 35 | 0:LIVING | Female | Primary | Astrocytoma G3 | Glioblastoma G4 | Vascular proliferation |  | DDRm1 | DDRr4 | No combined CNA | Non-codel | WT | Unmethylated | NA | NA | IDHwt | LGm6 | LGr1 | PA-like |
| TCGA-DU-6393-01A | TCGA-DU-6393 | 66 | 1:DECEASED | Male | Primary | Oligodendroglioma G3 | Oligodendroglioma, IDH-mutant |  |  | DDRm2 | DDRr2 | No combined CNA | Codel | Mutant | Methylated | Mutant | 22 | IDHmut-codel | LGm3 | LGr1 | Codel |
| TCGA-DU-6394-01A | TCGA-DU-6394 | 53 | 1:DECEASED | Male | Primary | Oligodendroglioma G3 | Oligodendroglioma, IDH-mutant |  |  | DDRm2 | DDRr2 | No combined CNA | Codel | Mutant | Methylated | Mutant | 25 | IDHmut-codel | LGm3 | LGr1 | Codel |
| TCGA-DU-6395-01A | TCGA-DU-6395 | 31 | 1:DECEASED | Male | Primary | Oligoastrocytoma G2 | Astrocytoma, IDH-mutant lower grade |  |  | DDRm2 | DDRr1 | No combined CNA | Non-codel | Mutant | Methylated | NA | 18 | IDHmut-non-codel | LGm2 | LGr3 | G-CIMP-high |
| TCGA-DU-6396-01A | TCGA-DU-6396 | 31 | 1:DECEASED | Female | Primary | Oligoastrocytoma G3 | Astrocytoma, IDH-mutant lower grade |  |  | DDRm2 | DDRr1 | No combined CNA | Non-codel | Mutant | Methylated | WT | 31 | IDHmut-non-codel | LGm1 | LGr3 | G-CIMP-high |
| TCGA-DU-6397-01A | TCGA-DU-6397 | 45 | 1:DECEASED | Male | Primary | Oligodendroglioma G3 | Oligodendroglioma, IDH-mutant |  |  | DDRm2 | DDRr2 | No combined CNA | Codel | Mutant | Methylated | Mutant | 17 | IDHmut-codel | LGm3 | LGr1 | Codel |
| TCGA-DU-6397-02A | TCGA-DU-6397 | NA | NA | NA | Recurrent | Oligodendroglioma G3 | Oligodendroglioma, IDH-mutant |  |  | DDRm2 | DDRr2 | NA | NA | NA | NA | NA | NA | NA | NA | NA | NA |
| TCGA-DU-6399-01A | TCGA-DU-6399 | 54 | 1:DECEASED | Male | Primary | Oligodendroglioma G2 | Astrocytoma, IDH-mutant lower grade |  |  | DDRm2 | DDRr1 | No combined CNA | Non-codel | Mutant | Methylated | WT | 32 | IDHwt | LGm2 | LGr3 | G-CIMP-high |
| TCGA-DU-6400-01A | TCGA-DU-6400 | 66 | 1:DECEASED | Female | Primary | Oligodendroglioma G2 | Oligodendroglioma, IDH-mutant |  |  | DDRm2 | DDRr2 | No combined CNA | Codel | Mutant | Methylated | Mutant | 37 | IDHmut-codel | LGm3 | LGr1 | Codel |
| TCGA-DU-6401-01A | TCGA-DU-6401 | 31 | 1:DECEASED | Female | Primary | Oligodendroglioma G2 | Astrocytoma, IDH-mutant lower grade |  |  | DDRm2 | DDRr1 | No combined CNA | Non-codel | Mutant | Methylated | WT | 16 | IDHmut-non-codel | LGm2 | LGr3 | G-CIMP-high |
| TCGA-DU-6402-01A | TCGA-DU-6402 | 52 | 1:DECEASED | Male | Primary | Astrocytoma G3 | Glioblastoma G4 | +7/-10, TERTp mut |  | DDRm1 | DDRr4 | Gain chr 7 & loss chr 10 | Non-codel | WT | Unmethylated | Mutant | 35 | IDHwt | LGm4 | LGr4 | Classic-like |
| TCGA-DU-6403-01A | TCGA-DU-6403 | 60 | 1:DECEASED | Female | Primary | Oligoastrocytoma G3 | Glioblastoma G4 | EGFR amp, +7/-10, TERTp mut |  | DDRm1 | DDRr4 | Gain chr 7 & loss chr 10 | Non-codel | WT | Unmethylated | Mutant | 50 | IDHwt | LGm5 | LGr1 | Classic-like |
| TCGA-DU-6404-01A | TCGA-DU-6404 | 24 | 1:DECEASED | Female | Primary | Oligodendroglioma G3 | Glioma, IDH-wildtype NEC |  |  | DDRm1 | DDRr1 | No combined CNA | Non-codel | WT | Unmethylated | WT | 9 | IDHwt | LGm6 | LGr4 | PA-like |
| TCGA-DU-6404-02A | TCGA-DU-6404 | NA | NA | NA | Recurrent | Oligodendroglioma G3 | Glioma, IDH-wildtype NEC |  |  | DDRm1 | DDRr1 | NA | NA | NA | NA | NA | NA | NA | NA | NA | NA |
| TCGA-DU-6404-02B | TCGA-DU-6404 | NA | NA | NA | Recurrent | Glioblastoma G4 | Glioblastoma G4 | Necrosis |  | DDRm1 | DDRr4 | NA | NA | NA | NA | NA | NA | NA | NA | NA | NA |
| TCGA-DU-6405-01A | TCGA-DU-6405 | 51 | 1:DECEASED | Female | Primary | Astrocytoma G3 | Glioblastoma G4 | EGFR amp, +7/-10, TERTp mut |  | DDRm1 | DDRr4 | Gain chr 7 & loss chr 10 | Non-codel | WT | Methylated | Mutant | 46 | IDHwt | LGm4 | LGr4 | Classic-like |
| TCGA-DU-6406-01A | TCGA-DU-6406 | 59 | 1:DECEASED | Female | Primary | Oligoastrocytoma G3 | Glioblastoma G4 | +7/-10, EGFR amp |  | DDRm1 | DDRr4 | Gain chr 7 & loss chr 10 | Non-codel | WT | Unmethylated | NA | 3 | IDHwt | LGm4 | LGr4 | Classic-like |
| TCGA-DU-6407-01A | TCGA-DU-6407 | 35 | 1:DECEASED | Female | Primary | Oligodendroglioma G2 | Astrocytoma, IDH-mutant G4 | CDKN2A/Bdel |  | DDRm2 | DDRr1 | No combined CNA | Non-codel | Mutant | Methylated | WT | 16 | IDHmut-non-codel | LGm2 | LGr3 | G-CIMP-high |
| TCGA-DU-6407-02A | TCGA-DU-6407 | NA | NA | NA | Recurrent | Oligoastrocytoma G2 | Astrocytoma, IDH-mutant G4 |  |  | DDRm2 | DDRr1 | NA | NA | NA | NA | NA | NA | NA | NA | NA | NA |
| TCGA-DU-6407-02B | TCGA-DU-6407 | NA | NA | NA | Recurrent | Glioblastoma G4 | Astrocytoma, IDH-mutant G4 |  |  | DDRm2 | DDRr4 | NA | NA | NA | NA | NA | NA | NA | NA | NA | NA |
| TCGA-DU-6408-01A | TCGA-DU-6408 | 23 | 1:DECEASED | Female | Primary | Oligodendroglioma G3 | Astrocytoma, IDH-mutant lower grade |  | Vascular hyperplasia mentioned in report but mitotic activity is low. No vascular proliferation is seen upon review of tissue. | DDRm2 | DDRr1 | No combined CNA | Non-codel | Mutant | Methylated | WT | 17 | IDHmut-non-codel | LGm1 | LGr3 | G-CIMP-high |
| TCGA-DU-6410-01A | TCGA-DU-6410 | 56 | 0:LIVING | Male | Primary | Oligodendroglioma G3 | Oligodendroglioma, IDH-mutant |  |  | DDRm2 | DDRr2 | No combined CNA | Codel | Mutant | Methylated | Mutant | 28 | IDHmut-codel | LGm3 | LGr1 | Codel |
| TCGA-DU-6542-01A | TCGA-DU-6542 | 25 | 0:LIVING | Male | Primary | Glioblastoma G4 | Astrocytoma, IDH-mutant G4 | Vascular proliferation, necrosis |  | DDRm2 | DDRr1 | No combined CNA | Non-codel | Mutant | Methylated | WT | 17 | IDHmut-non-codel | LGm2 | LGr3 | G-CIMP-high |
| TCGA-DU-7006-01A | TCGA-DU-7006 | 60 | 1:DECEASED | Female | Primary | Astrocytoma G3 | Glioblastoma G4 | +7/-10, TERTp mut |  | DDRm1 | DDRr4 | Gain chr 7 & loss chr 10 | Non-codel | WT | Methylated | Mutant | 44 | IDHwt | LGm5 | LGr4 | Mesenchymal-like |
| TCGA-DU-7007-01A | TCGA-DU-7007 | 33 | 1:DECEASED | Male | Primary | Astrocytoma G2 | Astrocytoma, IDH-mutant lower grade |  |  | DDRm2 | DDRr1 | No combined CNA | Non-codel | Mutant | Methylated | WT | 29 | IDHmut-non-codel | LGm2 | LGr3 | G-CIMP-high |
| TCGA-DU-7008-01A | TCGA-DU-7008 | 41 | 0:LIVING | Female | Primary | Oligodendroglioma G2 | Astrocytoma, IDH-mutant lower grade |  |  | DDRm2 | DDRr1 | No combined CNA | Non-codel | Mutant | Methylated | WT | 18 | IDHmut-non-codel | LGm2 | LGr3 | G-CIMP-high |
| TCGA-DU-7009-01A | TCGA-DU-7009 | 32 | 0:LIVING | Female | Primary | Oligodendroglioma G2 | Oligodendroglioma, IDH-mutant |  |  | DDRm2 | DDRr2 | No combined CNA | Codel | Mutant | Methylated | Mutant | 12 | IDHmut-codel | LGm3 | LGr2 | Codel |
| TCGA-DU-7010-01A | TCGA-DU-7010 | 58 | 1:DECEASED | Female | Primary | Astrocytoma G3 | Astrocytoma, IDH-mutant lower grade |  |  | DDRm2 | DDRr4 | No combined CNA | Non-codel | Mutant | Methylated | WT | 67 | IDHmut-non-codel | LGm1 | LGr3 | G-CIMP-low |
| TCGA-DU-7011-01A | TCGA-DU-7011 | 25 | 1:DECEASED | Male | Primary | Oligoastrocytoma G2 | Astrocytoma, IDH-mutant lower grade |  |  | DDRm2 | DDRr1 | No combined CNA | Non-codel | Mutant | Unmethylated | NA | 10 | IDHmut-non-codel | LGm2 | LGr3 | G-CIMP-high |
| TCGA-DU-7012-01A | TCGA-DU-7012 | 74 | 1:DECEASED | Female | Primary | Astrocytoma G3 | Glioblastoma G4 | +7/-10, TERTp mut |  | DDRm1 | DDRr4 | Gain chr 7 & loss chr 10 | Non-codel | WT | Methylated | Mutant | 38 | IDHwt | LGm5 | LGr4 | Mesenchymal-like |
| TCGA-DU-7013-01A | TCGA-DU-7013 | 59 | 1:DECEASED | Male | Primary | Astrocytoma G3 | Glioblastoma G4 | EGFR amp, +7/-10, TERTp mut |  | DDRm1 | DDRr4 | Gain chr 7 & loss chr 10 | Non-codel | WT | Unmethylated | Mutant | 29 | IDHwt | LGm4 | LGr4 | Classic-like |
| TCGA-DU-7014-01A | TCGA-DU-7014 | 59 | 1:DECEASED | Male | Primary | Oligodendroglioma G2 | Glioma NOS |  |  | DDRm2 | DDRr1 | No combined CNA | Non-codel | NA | Methylated | NA | NA | NA | LGm2 | LGr3 | NA |
| TCGA-DU-7015-01A | TCGA-DU-7015 | 41 | 0:LIVING | Female | Primary | Oligodendroglioma G2 | Astrocytoma, IDH-mutant lower grade |  |  | DDRm2 | DDRr1 | No combined CNA | Non-codel | Mutant | Methylated | WT | 17 | IDHmut-non-codel | LGm2 | LGr3 | G-CIMP-high |
| TCGA-DU-7018-01A | TCGA-DU-7018 | 57 | 1:DECEASED | Female | Primary | Oligodendroglioma G3 | Oligodendroglioma, IDH-mutant |  |  | DDRm2 | DDRr2 | No combined CNA | Codel | Mutant | Methylated | Mutant | 23 | IDHmut-codel | LGm3 | LGr1 | Codel |
| TCGA-DU-7019-01A | TCGA-DU-7019 | 39 | 0:LIVING | Male | Primary | Oligoastrocytoma G3 | Astrocytoma, IDH-mutant lower grade |  | Only subtle vascular hyperplasia described | DDRm2 | DDRr1 | No combined CNA | Non-codel | Mutant | Methylated | WT | 18 | IDHmut-non-codel | LGm2 | LGr3 | G-CIMP-high |
| TCGA-DU-7290-01A | TCGA-DU-7290 | 45 | 1:DECEASED | Female | Primary | Astrocytoma G3 | Glioblastoma G4 | TERTp mut |  | DDRm1 | DDRr4 | No combined CNA | Non-codel | WT | Unmethylated | Mutant | 28 | IDHwt | LGm5 | LGr4 | Mesenchymal-like |
| TCGA-DU-7292-01A | TCGA-DU-7292 | 69 | 1:DECEASED | Male | Primary | Astrocytoma G3 | Glioblastoma G4 | EGFR amp |  | DDRm1 | DDRr3 | No combined CNA | Non-codel | WT | Methylated | WT | 33 | IDHwt | LGm4 | LGr2 | Classic-like |
| TCGA-DU-7294-01A | TCGA-DU-7294 | 53 | 0:LIVING | Female | Primary | Oligodendroglioma G2 | Oligodendroglioma, IDH-mutant |  |  | DDRm2 | DDRr2 | No combined CNA | Codel | Mutant | Methylated | Mutant | 38 | IDHmut-codel | LGm3 | LGr1 | Codel |
| TCGA-DU-7298-01A | TCGA-DU-7298 | 38 | 1:DECEASED | Female | Primary | Astrocytoma G3 | Astrocytoma, IDH-mutant G4 | CDKN2A/Bdel |  | DDRm2 | DDRr1 | No combined CNA | Non-codel | Mutant | Methylated | WT | 33 | IDHmut-non-codel | LGm2 | LGr3 | G-CIMP-high |
| TCGA-DU-7299-01A | TCGA-DU-7299 | 33 | 1:DECEASED | Male | Primary | Astrocytoma G3 | Astrocytoma, IDH-mutant lower grade |  |  | DDRm2 | DDRr1 | No combined CNA | Non-codel | Mutant | Methylated | WT | 22 | IDHmut-non-codel | LGm2 | LGr3 | G-CIMP-high |
| TCGA-DU-7300-01A | TCGA-DU-7300 | 53 | 1:DECEASED | Female | Primary | Oligodendroglioma G3 | Oligodendroglioma, IDH-mutant |  |  | DDRm2 | DDRr3 | No combined CNA | Codel | Mutant | Methylated | Mutant | 41 | IDHmut-codel | LGm3 | LGr2 | Codel |
| TCGA-DU-7301-01A | TCGA-DU-7301 | 53 | 1:DECEASED | Male | Primary | Oligodendroglioma G2 | Astrocytoma, IDH-mutant lower grade |  |  | DDRm2 | DDRr1 | No combined CNA | Non-codel | Mutant | Methylated | WT | 18 | IDHmut-non-codel | LGm2 | LGr3 | G-CIMP-high |
| TCGA-DU-7302-01A | TCGA-DU-7302 | 48 | 0:LIVING | Female | Primary | Oligodendroglioma G3 | Oligodendroglioma, IDH-mutant |  |  | DDRm2 | DDRr3 | No combined CNA | Codel | Mutant | Methylated | Mutant | 20 | IDHmut-codel | LGm3 | LGr2 | Codel |
| TCGA-DU-7304-01A | TCGA-DU-7304 | 43 | 1:DECEASED | Male | Primary | Oligoastrocytoma G3 | Astrocytoma, IDH-mutant lower grade |  |  | DDRm2 | DDRr1 | No combined CNA | Non-codel | Mutant | Methylated | WT | 18 | IDHmut-non-codel | LGm2 | LGr3 | G-CIMP-high |
| TCGA-DU-7304-02A | TCGA-DU-7304 | NA | NA | NA | Recurrent | Oligoastrocytoma G3 | Astrocytoma, IDH-mutant lower grade |  |  | DDRm2 | DDRr1 | NA | NA | NA | NA | NA | NA | NA | NA | NA | NA |
| TCGA-DU-7306-01A | TCGA-DU-7306 | 67 | 0:LIVING | Male | Primary | Oligoastrocytoma G2 | Astrocytoma, IDH-mutant lower grade |  |  | DDRm2 | DDRr1 | No combined CNA | Non-codel | Mutant | Methylated | WT | 39 | IDHmut-non-codel | LGm2 | LGr3 | G-CIMP-high |
| TCGA-DU-7309-01A | TCGA-DU-7309 | 41 | 0:LIVING | Female | Primary | Oligodendroglioma G3 | Astrocytoma, IDH-mutant lower grade |  | Only early microvascular proliferation described | DDRm2 | DDRr3 | No combined CNA | Non-codel | Mutant | Methylated | WT | 23 | IDHmut-non-codel | LGm2 | LGr2 | G-CIMP-high |
| TCGA-DU-8158-01A | TCGA-DU-8158 | 57 | 1:DECEASED | Female | Primary | Astrocytoma G3 | Glioblastoma G4 | TERTp mut |  | DDRm1 | DDRr4 | No combined CNA | Non-codel | WT | Unmethylated | Mutant | 31 | IDHwt | LGm5 | LGr4 | Mesenchymal-like |
| TCGA-DU-8161-01A | TCGA-DU-8161 | 63 | 1:DECEASED | Female | Primary | Oligoastrocytoma G3 | Glioblastoma G4 | +7/-10, TERTp mut |  | DDRm1 | DDRr4 | Gain chr 7 & loss chr 10 | Non-codel | WT | Unmethylated | Mutant | 33 | IDHwt | LGm5 | LGr4 | Mesenchymal-like |
| TCGA-DU-8162-01A | TCGA-DU-8162 | 61 | 1:DECEASED | Female | Primary | Oligoastrocytoma G3 | Glioblastoma G4 | EGFR amp |  | DDRm1 | DDRr3 | No combined CNA | Non-codel | WT | Unmethylated | WT | 10 | IDHwt | LGm5 | LGr2 | Mesenchymal-like |
| TCGA-DU-8163-01A | TCGA-DU-8163 | 29 | 0:LIVING | Male | Primary | Oligoastrocytoma G3 | Astrocytoma, IDH-mutant G4 | Vascular proliferation |  | DDRm2 | DDRr1 | No combined CNA | Non-codel | Mutant | Unmethylated | WT | 9 | IDHmut-non-codel | LGm2 | LGr3 | G-CIMP-high |
| TCGA-DU-8164-01A | TCGA-DU-8164 | 51 | 0:LIVING | Male | Primary | Oligodendroglioma G2 | Oligodendroglioma, IDH-mutant |  |  | DDRm2 | DDRr2 | No combined CNA | Codel | Mutant | Methylated | Mutant | 22 | IDHmut-codel | LGm3 | LGr2 | Codel |
| TCGA-DU-8165-01A | TCGA-DU-8165 | 60 | 0:LIVING | Female | Primary | Oligodendroglioma G3 | Glioblastoma G4 | +7/-10, TERTp mut |  | DDRm1 | DDRr4 | Gain chr 7 & loss chr 10 | Non-codel | WT | Unmethylated | Mutant | 46 | IDHwt | LGm5 | LGr4 | Mesenchymal-like |
| TCGA-DU-8166-01A | TCGA-DU-8166 | 29 | 0:LIVING | Female | Primary | Oligoastrocytoma G2 | Astrocytoma, IDH-mutant lower grade |  |  | DDRm2 | DDRr1 | No combined CNA | Non-codel | Mutant | Methylated | WT | 13 | IDHmut-non-codel | LGm2 | LGr3 | G-CIMP-high |
| TCGA-DU-8167-01A | TCGA-DU-8167 | 69 | 0:LIVING | Female | Primary | Oligoastrocytoma G2 | Astrocytoma, IDH-mutant lower grade |  |  | DDRm2 | DDRr1 | No combined CNA | Non-codel | Mutant | Methylated | WT | 26 | IDHmut-non-codel | LGm2 | LGr3 | G-CIMP-high |
| TCGA-DU-8168-01A | TCGA-DU-8168 | 55 | 0:LIVING | Female | Primary | Oligodendroglioma G3 | Oligodendroglioma, IDH-mutant |  |  | DDRm2 | DDRr2 | No combined CNA | Codel | Mutant | Methylated | Mutant | 42 | IDHmut-codel | LGm3 | LGr1 | Codel |
| TCGA-DU-A5TP-01A | TCGA-DU-A5TP | 33 | 0:LIVING | Male | Primary | Astrocytoma G3 | Astrocytoma, IDH-mutant lower grade |  |  | DDRm2 | DDRr1 | No combined CNA | Non-codel | Mutant | Methylated | WT | 17 | IDHmut-non-codel | LGm1 | LGr3 | G-CIMP-high |
| TCGA-DU-A5TR-01A | TCGA-DU-A5TR | 51 | 0:LIVING | Male | Primary | Oligoastrocytoma G2 | Astrocytoma, IDH-mutant lower grade |  |  | DDRm2 | DDRr1 | No combined CNA | Non-codel | Mutant | Methylated | WT | 31 | IDHmut-non-codel | LGm2 | LGr3 | G-CIMP-high |
| TCGA-DU-A5TS-01A | TCGA-DU-A5TS | 42 | 0:LIVING | Male | Primary | Oligodendroglioma G2 | Astrocytoma, IDH-mutant lower grade |  |  | DDRm2 | DDRr1 | No combined CNA | Non-codel | Mutant | Methylated | WT | 31 | IDHmut-non-codel | LGm2 | LGr3 | G-CIMP-high |
| TCGA-DU-A5TT-01A | TCGA-DU-A5TT | 70 | 0:LIVING | Male | Primary | Oligodendroglioma G3 | Glioblastoma G4 | TERTp mut |  | DDRm1 | DDRr1 | No combined CNA | Non-codel | WT | Methylated | Mutant | 39 | IDHwt | LGm5 | LGr4 | Mesenchymal-like |
| TCGA-DU-A5TU-01A | TCGA-DU-A5TU | 62 | 0:LIVING | Female | Primary | Astrocytoma G2 | Astrocytoma, IDH-mutant lower grade |  |  | DDRm2 | DDRr1 | No combined CNA | Non-codel | Mutant | Methylated | WT | 25 | IDHmut-non-codel | LGm3 | LGr3 | G-CIMP-high |
| TCGA-DU-A5TW-01A | TCGA-DU-A5TW | 33 | 0:LIVING | Female | Primary | Astrocytoma G3 | Astrocytoma, IDH-mutant lower grade |  |  | DDRm2 | DDRr1 | No combined CNA | Non-codel | Mutant | Methylated | WT | 28 | IDHmut-non-codel | LGm2 | LGr3 | G-CIMP-high |
| TCGA-DU-A5TY-01A | TCGA-DU-A5TY | 46 | 0:LIVING | Female | Primary | Astrocytoma G3 | Glioblastoma G4 | +7/-10, TERTp mut |  | DDRm1 | DDRr4 | Gain chr 7 & loss chr 10 | Non-codel | WT | Methylated | Mutant | 34 | IDHwt | LGm5 | LGr4 | Mesenchymal-like |
| TCGA-DU-A6S2-01A | TCGA-DU-A6S2 | 37 | 0:LIVING | Female | Primary | Oligodendroglioma G2 | Oligodendroglioma, IDH-mutant |  |  | DDRm2 | DDRr3 | No combined CNA | Codel | Mutant | Methylated | NA | 12 | IDHmut-codel | LGm2 | LGr2 | Codel |
| TCGA-DU-A6S3-01A | TCGA-DU-A6S3 | 60 | 0:LIVING | Male | Primary | Oligodendroglioma G2 | Oligodendroglioma, IDH-mutant |  |  | DDRm2 | DDRr2 | No combined CNA | Codel | Mutant | Methylated | NA | 25 | IDHmut-codel | LGm3 | LGr2 | Codel |
| TCGA-DU-A6S6-01A | TCGA-DU-A6S6 | 35 | 0:LIVING | Female | Primary | Oligoastrocytoma G2 | Oligodendroglioma, IDH-mutant |  |  | DDRm2 | DDRr3 | No combined CNA | Codel | Mutant | Methylated | NA | 13 | IDHmut-codel | LGm2 | LGr2 | Codel |
| TCGA-DU-A6S7-01A | TCGA-DU-A6S7 | 27 | 0:LIVING | Female | Primary | Astrocytoma G3 | Astrocytoma, IDH-mutant lower grade |  |  | DDRm2 | DDRr1 | No combined CNA | Non-codel | Mutant | Methylated | NA | 17 | IDHmut-non-codel | LGm2 | LGr3 | G-CIMP-high |
| TCGA-DU-A6S8-01A | TCGA-DU-A6S8 | 74 | 0:LIVING | Female | Primary | Oligodendroglioma G3 | Oligodendroglioma, IDH-mutant |  |  | DDRm2 | DDRr2 | No combined CNA | Codel | Mutant | Methylated | NA | 45 | IDHmut-codel | LGm3 | LGr1 | Codel |
| TCGA-DU-A76K-01A | TCGA-DU-A76K | 87 | 1:DECEASED | Male | Primary | Oligodendroglioma G2 | Glioblastoma G4 | -0.7 |  | DDRm1 | DDRr1 | Gain chr 7 & loss chr 10 | Non-codel | WT | Unmethylated | NA | 38 | IDHwt | LGm4 | LGr4 | Classic-like |
| TCGA-DU-A76L-01A | TCGA-DU-A76L | 54 | 1:DECEASED | Male | Primary | Oligodendroglioma G3 | Glioblastoma G4 | +7/-10, EGFR amp |  | DDRm1 | DDRr4 | Gain chr 7 & loss chr 10 | Non-codel | WT | Methylated | NA | 29 | IDHwt | LGm5 | LGr1 | Mesenchymal-like |
| TCGA-DU-A76O-01A | TCGA-DU-A76O | 30 | 0:LIVING | Male | Primary | Astrocytoma G2 | Astrocytoma, IDH-mutant lower grade |  |  | DDRm2 | DDRr1 | No combined CNA | Non-codel | Mutant | Methylated | NA | 14 | IDHmut-non-codel | LGm2 | LGr3 | G-CIMP-high |
| TCGA-DU-A76R-01A | TCGA-DU-A76R | 51 | 1:DECEASED | Male | Primary | Oligodendroglioma G3 | Oligodendroglioma, IDH-mutant |  |  | DDRm2 | DDRr2 | No combined CNA | Codel | Mutant | Methylated | NA | 30 | IDHmut-codel | LGm3 | LGr3 | Codel |
| TCGA-DU-A7T6-01A | TCGA-DU-A7T6 | 73 | 1:DECEASED | Female | Primary | Oligodendroglioma G3 | Oligodendroglioma, IDH-mutant |  |  | DDRm2 | DDRr2 | No combined CNA | Codel | Mutant | Methylated | NA | 83 | IDHmut-codel | LGm3 | LGr1 | Codel |
| TCGA-DU-A7T8-01A | TCGA-DU-A7T8 | 35 | 0:LIVING | Male | Primary | Oligoastrocytoma G3 | Astrocytoma, IDH-mutant lower grade |  |  | DDRm2 | DDRr1 | No combined CNA | Non-codel | Mutant | Methylated | NA | 14 | IDHmut-non-codel | LGm2 | LGr3 | G-CIMP-high |
| TCGA-DU-A7TA-01A | TCGA-DU-A7TA | 32 | 0:LIVING | Male | Primary | Oligodendroglioma G2 | Astrocytoma, IDH-mutant lower grade |  |  | DDRm2 | DDRr1 | No combined CNA | Non-codel | Mutant | Methylated | NA | 33 | IDHmut-non-codel | LGm2 | LGr3 | G-CIMP-high |
| TCGA-DU-A7TB-01A | TCGA-DU-A7TB | 56 | 0:LIVING | Male | Primary | Oligodendroglioma G2 | Glioma, IDH-wildtype NOS |  |  | DDRm1 | DDRr1 | No combined CNA | Non-codel | WT | Unmethylated | NA | 26 | IDHwt | LGm6 | LGr1 | PA-like |
| TCGA-DU-A7TC-01A | TCGA-DU-A7TC | 32 | 0:LIVING | Male | Primary | Astrocytoma G2 | Astrocytoma, IDH-mutant lower grade |  |  | DDRm2 | DDRr3 | No combined CNA | Non-codel | Mutant | Methylated | NA | 18 | IDHmut-non-codel | LGm2 | LGr2 | G-CIMP-high |
| TCGA-DU-A7TD-01A | TCGA-DU-A7TD | 52 | 1:DECEASED | Male | Primary | Oligoastrocytoma G3 | Glioblastoma G4 | +7/-10, EGFR amp |  | DDRm1 | DDRr4 | Gain chr 7 & loss chr 10 | Non-codel | WT | Unmethylated | NA | 31 | IDHwt | LGm5 | LGr4 | Mesenchymal-like |
| TCGA-DU-A7TG-01A | TCGA-DU-A7TG | 40 | 1:DECEASED | Male | Primary | Oligodendroglioma G2 | Astrocytoma, IDH-mutant lower grade |  |  | DDRm2 | DDRr3 | No combined CNA | Non-codel | Mutant | Methylated | NA | 6 | IDHmut-non-codel | LGm1 | LGr2 | G-CIMP-high |
| TCGA-DU-A7TI-01A | TCGA-DU-A7TI | NA | NA | NA | Primary | Astrocytoma G3 | Glioma NOS |  |  | DDRm2 | DDRr1 | No combined CNA | Non-codel | NA | Methylated | NA | NA | NA | LGm2 | LGr3 | NA |
| TCGA-DU-A7TJ-01A | TCGA-DU-A7TJ | 55 | 0:LIVING | Male | Primary | Astrocytoma G3 | Glioblastoma G4 | +7/-10, EGFR amp |  | DDRm1 | DDRr4 | Gain chr 7 & loss chr 10 | Non-codel | WT | Methylated | NA | 30 | IDHwt | LGm5 | LGr4 | Mesenchymal-like |
| TCGA-E1-5302-01A | TCGA-E1-5302 | 41 | 1:DECEASED | Male | Primary | Astrocytoma G3 | Astrocytoma, IDH-mutant lower grade |  |  | DDRm2 | DDRr1 | No combined CNA | Non-codel | Mutant | Methylated | WT | 13 | IDHmut-non-codel | LGm2 | LGr3 | G-CIMP-high |
| TCGA-E1-5303-01A | TCGA-E1-5303 | 38 | 1:DECEASED | Male | Primary | Astrocytoma G3 | Astrocytoma, IDH-mutant lower grade |  |  | DDRm2 | DDRr1 | No combined CNA | Non-codel | Mutant | Methylated | WT | 23 | IDHmut-non-codel | LGm2 | LGr3 | G-CIMP-high |
| TCGA-E1-5304-01A | TCGA-E1-5304 | 42 | 1:DECEASED | Male | Primary | Astrocytoma G3 | Astrocytoma, IDH-mutant lower grade |  |  | DDRm2 | DDRr4 | No combined CNA | Non-codel | Mutant | Unmethylated | WT | 25 | IDHmut-non-codel | LGm1 | LGr3 | G-CIMP-low |
| TCGA-E1-5305-01A | TCGA-E1-5305 | 34 | 1:DECEASED | Male | Primary | Astrocytoma G3 | Astrocytoma, IDH-mutant G4 | Focal vascular proliferation |  | DDRm2 | DDRr1 | No combined CNA | Non-codel | Mutant | Methylated | WT | 22 | IDHmut-non-codel | LGm2 | LGr3 | G-CIMP-high |
| TCGA-E1-5307-01A | TCGA-E1-5307 | 62 | 1:DECEASED | Female | Primary | Astrocytoma G3 | Astrocytoma, IDH-mutant lower grade |  |  | DDRm2 | DDRr1 | No combined CNA | Non-codel | Mutant | Methylated | WT | 46 | IDHmut-non-codel | LGm3 | LGr3 | G-CIMP-high |
| TCGA-E1-5311-01A | TCGA-E1-5311 | 31 | 1:DECEASED | Male | Primary | Oligodendroglioma G3 | Oligodendroglioma, IDH-mutant |  |  | DDRm2 | DDRr2 | No combined CNA | Codel | Mutant | Methylated | Mutant | 12 | IDHmut-codel | LGm3 | LGr2 | Codel |
| TCGA-E1-5318-01A | TCGA-E1-5318 | 42 | 1:DECEASED | Female | Primary | Oligodendroglioma G2 | Oligodendroglioma, IDH-mutant |  |  | DDRm2 | DDRr2 | No combined CNA | Codel | Mutant | Methylated | Mutant | 23 | IDHmut-codel | LGm3 | LGr1 | Codel |
| TCGA-E1-5319-01A | TCGA-E1-5319 | 48 | 1:DECEASED | Female | Primary | Oligodendroglioma G2 | Oligodendroglioma, IDH-mutant |  |  | DDRm2 | DDRr2 | No combined CNA | Codel | Mutant | Methylated | Mutant | 26 | IDHmut-codel | LGm3 | LGr1 | Codel |
| TCGA-E1-5322-01A | TCGA-E1-5322 | 38 | 1:DECEASED | Female | Primary | Oligoastrocytoma G2 | Astrocytoma, IDH-mutant lower grade |  |  | DDRm2 | DDRr1 | No combined CNA | Non-codel | Mutant | Methylated | Mutant | 17 | IDHmut-non-codel | LGm2 | LGr3 | G-CIMP-high |
| TCGA-E1-A7YD-01A | TCGA-E1-A7YD | NA | NA | NA | Primary | Astrocytoma G3 | Glioblastoma G4 | +7/-10, EGFR amp |  | DDRm1 | DDRr4 | Gain chr 7 & loss chr 10 | Non-codel | WT | Unmethylated | NA | 43 | IDHwt | LGm5 | LGr4 | Mesenchymal-like |
| TCGA-E1-A7YE-01A | TCGA-E1-A7YE | NA | NA | NA | Primary | Astrocytoma G3 | Astrocytoma, IDH-mutant lower grade |  |  | DDRm2 | DDRr1 | No combined CNA | Non-codel | Mutant | Unmethylated | NA | 56 | IDHmut-non-codel | LGm1 | LGr3 | G-CIMP-low |
| TCGA-E1-A7YH-01A | TCGA-E1-A7YH | NA | NA | NA | Primary | Astrocytoma G3 | Astrocytoma, IDH-mutant lower grade |  |  | DDRm2 | DDRr1 | No combined CNA | Non-codel | Mutant | Methylated | NA | 17 | IDHmut-non-codel | LGm2 | LGr3 | G-CIMP-high |
| TCGA-E1-A7YI-01A | TCGA-E1-A7YI | NA | NA | NA | Primary | Astrocytoma G3 | Astrocytoma, IDH-mutant G4 | CDKN2A/Bdel |  | DDRm2 | DDRr4 | No combined CNA | Non-codel | Mutant | Methylated | NA | 33 | IDHmut-non-codel | LGm1 | LGr1 | G-CIMP-low |
| TCGA-E1-A7YJ-01A | TCGA-E1-A7YJ | NA | NA | NA | Primary | Astrocytoma G3 | Glioblastoma G4 | +7/-10, EGFR amp |  | DDRm1 | DDRr1 | Gain chr 7 & loss chr 10 | Non-codel | WT | Unmethylated | NA | 27 | IDHwt | LGm4 | LGr4 | Classic-like |
| TCGA-E1-A7YK-01A | TCGA-E1-A7YK | NA | NA | NA | Primary | Astrocytoma G3 | Astrocytoma, IDH-mutant lower grade |  |  | DDRm2 | DDRr1 | No combined CNA | Non-codel | Mutant | Methylated | NA | 30 | IDHmut-non-codel | LGm1 | LGr3 | G-CIMP-high |
| TCGA-E1-A7YL-01A | TCGA-E1-A7YL | NA | NA | NA | Primary | Astrocytoma G3 | Glioblastoma G4 | -0.7 |  | DDRm1 | DDRr4 | Gain chr 7 & loss chr 10 | Non-codel | WT | Unmethylated | NA | 46 | IDHwt | LGm5 | LGr4 | Mesenchymal-like |
| TCGA-E1-A7YM-01A | TCGA-E1-A7YM | NA | NA | NA | Primary | Astrocytoma G3 | Glioblastoma G4 | -0.7 |  | DDRm1 | DDRr1 | Gain chr 7 & loss chr 10 | Non-codel | WT | Unmethylated | NA | 34 | IDHwt | LGm5 | LGr4 | Mesenchymal-like |
| TCGA-E1-A7YN-01A | TCGA-E1-A7YN | NA | NA | NA | Primary | Astrocytoma G3 | Glioblastoma G4 | -0.7 |  | DDRm1 | DDRr1 | Gain chr 7 & loss chr 10 | Non-codel | WT | Methylated | NA | 42 | IDHwt | LGm5 | LGr4 | Mesenchymal-like |
| TCGA-E1-A7YO-01A | TCGA-E1-A7YO | 45 | 1:DECEASED | Male | Primary | Oligodendroglioma G3 | Oligodendroglioma, IDH-mutant |  |  | DDRm2 | DDRr2 | No combined CNA | Codel | Mutant | Methylated | NA | 23 | IDHmut-codel | LGm3 | LGr2 | Codel |
| TCGA-E1-A7YQ-01A | TCGA-E1-A7YQ | NA | NA | NA | Primary | Oligodendroglioma G3 | Glioblastoma G4 | +7/-10, EGFR amp |  | DDRm1 | DDRr4 | Gain chr 7 & loss chr 10 | Non-codel | WT | Unmethylated | NA | 25 | IDHwt | LGm4 | LGr4 | Classic-like |
| TCGA-E1-A7YS-01A | TCGA-E1-A7YS | NA | NA | NA | Primary | Oligodendroglioma G3 | Oligodendroglioma, IDH-mutant |  |  | DDRm2 | DDRr2 | No combined CNA | Codel | Mutant | Methylated | NA | 42 | IDHmut-codel | LGm3 | LGr1 | Codel |
| TCGA-E1-A7YU-01A | TCGA-E1-A7YU | NA | NA | NA | Primary | Oligoastrocytoma G3 | Astrocytoma, IDH-mutant G4 | Vascular proliferation |  | DDRm2 | DDRr1 | No combined CNA | Non-codel | Mutant | Methylated | NA | 18 | IDHmut-non-codel | LGm2 | LGr3 | G-CIMP-high |
| TCGA-E1-A7YV-01A | TCGA-E1-A7YV | 26 | 1:DECEASED | Female | Primary | Oligoastrocytoma G3 | Astrocytoma, IDH-mutant G4 | Microvascular changes |  | DDRm2 | DDRr4 | No combined CNA | Non-codel | Mutant | Methylated | NA | 28 | IDHmut-non-codel | LGm1 | LGr1 | G-CIMP-low |
| TCGA-E1-A7YW-01A | TCGA-E1-A7YW | 28 | 1:DECEASED | Male | Primary | Oligoastrocytoma G2 | Astrocytoma, IDH-mutant lower grade |  |  | DDRm2 | DDRr1 | No combined CNA | Non-codel | Mutant | Methylated | NA | 15 | IDHmut-non-codel | LGm2 | LGr3 | G-CIMP-high |
| TCGA-E1-A7YY-01A | TCGA-E1-A7YY | NA | NA | NA | Primary | Astrocytoma G2 | Astrocytoma, IDH-mutant lower grade |  |  | DDRm1 | DDRr3 | No combined CNA | Non-codel | Mutant | Methylated | NA | 1 | IDHmut-non-codel | LGm1 | LGr2 | G-CIMP-high |
| TCGA-E1-A7Z2-01A | TCGA-E1-A7Z2 | NA | NA | NA | Primary | Oligodendroglioma G2 | Glioblastoma G4 | -0.7 |  | DDRm1 | DDRr1 | Gain chr 7 & loss chr 10 | Non-codel | WT | Unmethylated | NA | 24 | IDHwt | LGm5 | LGr4 | Mesenchymal-like |
| TCGA-E1-A7Z3-01A | TCGA-E1-A7Z3 | NA | NA | NA | Primary | Astrocytoma G2 | Astrocytoma, IDH-mutant G4 | CDKN2A/Bdel |  | DDRm2 | DDRr3 | No combined CNA | Non-codel | Mutant | Methylated | NA | 16 | IDHmut-non-codel | LGm2 | LGr2 | G-CIMP-high |
| TCGA-E1-A7Z4-01A | TCGA-E1-A7Z4 | NA | NA | NA | Primary | Astrocytoma G2 | Astrocytoma, IDH-mutant lower grade |  |  | DDRm2 | DDRr1 | No combined CNA | Non-codel | Mutant | Methylated | NA | 9 | IDHmut-non-codel | LGm2 | LGr3 | G-CIMP-high |
| TCGA-E1-A7Z6-01A | TCGA-E1-A7Z6 | 41 | 1:DECEASED | Female | Primary | Astrocytoma G2 | Astrocytoma, IDH-mutant lower grade |  |  | DDRm2 | DDRr1 | No combined CNA | Non-codel | Mutant | Methylated | NA | 12 | IDHmut-non-codel | LGm2 | LGr3 | G-CIMP-high |
| TCGA-EZ-7264-01A | TCGA-EZ-7264 | 47 | 0:LIVING | Female | Primary | Oligodendroglioma G2 | Oligodendroglioma, IDH-mutant |  |  | DDRm2 | DDRr2 | No combined CNA | Codel | Mutant | Methylated | Mutant | 19 | IDHmut-codel | LGm3 | LGr1 | Codel |
| TCGA-F6-A8O3-01A | TCGA-F6-A8O3 | 34 | 0:LIVING | Male | Primary | Oligodendroglioma G2 | Oligodendroglioma, IDH-mutant |  |  | DDRm2 | DDRr2 | No combined CNA | Codel | Mutant | Methylated | NA | 20 | IDHmut-codel | LGm3 | LGr1 | Codel |
| TCGA-F6-A8O4-01A | TCGA-F6-A8O4 | 44 | 0:LIVING | Male | Primary | Astrocytoma G2 | Astrocytoma, IDH-mutant G4 | CDKN2A/Bdel |  | DDRm2 | DDRr1 | No combined CNA | Non-codel | Mutant | Methylated | NA | 23 | IDHmut-non-codel | LGm2 | LGr3 | G-CIMP-high |
| TCGA-FG-5962-01B | TCGA-FG-5962 | 54 | 0:LIVING | Male | Primary | Oligodendroglioma G3 | Oligodendroglioma, IDH-mutant |  |  | DDRm2 | DDRr3 | No combined CNA | Codel | Mutant | Methylated | Mutant | 21 | IDHmut-codel | LGm3 | LGr2 | Codel |
| TCGA-FG-5963-01A | TCGA-FG-5963 | 23 | 1:DECEASED | Male | Primary | Astrocytoma G3 | Glioblastoma G4 | EGFR amp |  | DDRm1 | DDRr1 | No combined CNA | Non-codel | WT | Unmethylated | WT | 19 | IDHwt | LGm6 | LGr3 | PA-like |
| TCGA-FG-5963-02A | TCGA-FG-5963 | NA | NA | NA | Recurrent | Glioblastoma G4 | Glioblastoma G4 |  |  | DDRm1 | DDRr4 | NA | NA | NA | NA | NA | NA | NA | NA | NA | NA |
| TCGA-FG-5964-01A | TCGA-FG-5964 | 62 | 0:LIVING | Male | Primary | Oligodendroglioma G2 | Oligodendroglioma, IDH-mutant |  |  | DDRm2 | DDRr2 | No combined CNA | Codel | Mutant | Methylated | Mutant | 26 | IDHmut-codel | LGm3 | LGr3 | Codel |
| TCGA-FG-5965-01B | TCGA-FG-5965 | 39 | 1:DECEASED | Female | Primary | Oligoastrocytoma G2 | Astrocytoma, IDH-mutant G4 | CDKN2A/Bdel |  | DDRm2 | DDRr1 | No combined CNA | Non-codel | Mutant | Methylated | WT | 31 | IDHmut-non-codel | LGm2 | LGr3 | G-CIMP-high |
| TCGA-FG-5965-02A | TCGA-FG-5965 | NA | NA | NA | Recurrent | Oligoastrocytoma G3 | Astrocytoma, IDH-mutant G4 |  |  | DDRm2 | DDRr4 | NA | NA | NA | NA | NA | NA | NA | NA | NA | NA |
| TCGA-FG-5965-02B | TCGA-FG-5965 | NA | NA | NA | Recurrent | Glioblastoma G4 | Astrocytoma, IDH-mutant G4 |  |  | DDRm2 | DDRr3 | NA | NA | NA | NA | NA | NA | NA | NA | NA | NA |
| TCGA-FG-6688-01A | TCGA-FG-6688 | 59 | 0:LIVING | Female | Primary | Astrocytoma G3 | Glioblastoma G4 | EGFR amp, +7/-10, TERTp mut |  | DDRm1 | DDRr4 | Gain chr 7 & loss chr 10 | Non-codel | WT | Methylated | Mutant | 45 | IDHwt | LGm5 | LGr4 | Mesenchymal-like |
| TCGA-FG-6689-01A | TCGA-FG-6689 | 30 | 0:LIVING | Male | Primary | Astrocytoma G2 | Astrocytoma, IDH-mutant lower grade |  |  | DDRm2 | DDRr3 | No combined CNA | Non-codel | Mutant | Methylated | NA | 12 | IDHmut-non-codel | LGm2 | LGr3 | G-CIMP-high |
| TCGA-FG-6690-01A | TCGA-FG-6690 | 70 | 0:LIVING | Male | Primary | Oligodendroglioma G2 | Astrocytoma, IDH-mutant lower grade |  |  | DDRm2 | DDRr1 | No combined CNA | Non-codel | Mutant | Methylated | WT | 15 | IDHmut-non-codel | LGm2 | LGr3 | G-CIMP-high |
| TCGA-FG-6691-01A | TCGA-FG-6691 | 23 | 0:LIVING | Female | Primary | Astrocytoma G2 | Astrocytoma, IDH-mutant lower grade |  |  | DDRm2 | DDRr1 | No combined CNA | Non-codel | Mutant | Unmethylated | WT | 8 | IDHmut-non-codel | LGm2 | LGr3 | G-CIMP-high |
| TCGA-FG-6692-01A | TCGA-FG-6692 | 63 | 1:DECEASED | Male | Primary | Oligodendroglioma G3 | Glioblastoma G4 | EGFR amp, +7/-10, TERTp mut |  | DDRm1 | DDRr4 | Gain chr 7 & loss chr 10 | Non-codel | WT | Methylated | Mutant | 58 | IDHwt | LGm4 | LGr4 | Classic-like |
| TCGA-FG-7634-01A | TCGA-FG-7634 | 28 | 0:LIVING | Male | Primary | Oligodendroglioma G2 | Oligodendroglioma, IDH-mutant |  |  | DDRm2 | DDRr1 | No combined CNA | Codel | Mutant | Methylated | Mutant | 11 | IDHmut-codel | LGm2 | LGr1 | Codel |
| TCGA-FG-7636-01A | TCGA-FG-7636 | 48 | 0:LIVING | Male | Primary | Astrocytoma G3 | Astrocytoma, IDH-mutant lower grade |  |  | DDRm2 | DDRr1 | No combined CNA | Non-codel | Mutant | Methylated | WT | 29 | IDHmut-non-codel | LGm2 | LGr3 | G-CIMP-high |
| TCGA-FG-7637-01A | TCGA-FG-7637 | 49 | 0:LIVING | Male | Primary | Oligoastrocytoma G2 | Astrocytoma, IDH-mutant lower grade |  | Necrosis is mentioned. However, other tumor features appear low grade (i.e., no mitotic activity) | DDRm2 | DDRr2 | NA | Non-codel | Mutant | Methylated | WT | 20 | IDHmut-non-codel | LGm3 | LGr1 | Codel |
| TCGA-FG-7638-01B | TCGA-FG-7638 | 31 | 0:LIVING | Female | Primary | Oligodendroglioma G3 | Oligodendroglioma, IDH-mutant |  |  | DDRm2 | DDRr2 | No combined CNA | Codel | Mutant | Methylated | Mutant | 11 | IDHmut-codel | LGm3 | LGr2 | Codel |
| TCGA-FG-7641-01B | TCGA-FG-7641 | 31 | 0:LIVING | Male | Primary | Oligodendroglioma G2 | Oligodendroglioma, IDH-mutant |  |  | DDRm2 | DDRr3 | No combined CNA | Codel | Mutant | Methylated | Mutant | 16 | IDHmut-codel | LGm3 | LGr2 | Codel |
| TCGA-FG-7643-01A | TCGA-FG-7643 | 49 | 0:LIVING | Female | Primary | Oligoastrocytoma G2 | Glioblastoma G4 | TERTp mut |  | DDRm1 | DDRr3 | No combined CNA | Non-codel | WT | Methylated | Mutant | 21 | IDHwt | LGm5 | LGr2 | Mesenchymal-like |
| TCGA-FG-8181-01A | TCGA-FG-8181 | 23 | 0:LIVING | Male | Primary | Oligoastrocytoma G3 | Glioma, IDH-wildtype NEC |  |  | DDRm1 | DDRr3 | No combined CNA | Non-codel | WT | Unmethylated | WT | NA | IDHwt | LGm6 | LGr2 | PA-like |
| TCGA-FG-8182-01A | TCGA-FG-8182 | 35 | 0:LIVING | Male | Primary | Oligodendroglioma G2 | Astrocytoma, IDH-mutant lower grade |  |  | DDRm2 | DDRr1 | No combined CNA | Non-codel | Mutant | Methylated | WT | 14 | IDHmut-non-codel | LGm2 | LGr3 | G-CIMP-high |
| TCGA-FG-8185-01A | TCGA-FG-8185 | 37 | 0:LIVING | Male | Primary | Astrocytoma G3 | Astrocytoma, IDH-mutant lower grade |  |  | DDRm2 | DDRr1 | No combined CNA | Non-codel | Mutant | Methylated | WT | 21 | IDHmut-non-codel | LGm2 | LGr3 | G-CIMP-high |
| TCGA-FG-8186-01A | TCGA-FG-8186 | 42 | 0:LIVING | Female | Primary | Oligoastrocytoma G3 | Oligodendroglioma, IDH-mutant |  |  | DDRm2 | DDRr2 | No combined CNA | Codel | Mutant | Methylated | Mutant | 17 | IDHmut-codel | LGm2 | LGr1 | Codel |
| TCGA-FG-8187-01A | TCGA-FG-8187 | 31 | 0:LIVING | Male | Primary | Oligoastrocytoma G2 | Oligodendroglioma, IDH-mutant |  |  | DDRm2 | DDRr2 | No combined CNA | Codel | Mutant | Methylated | Mutant | 6 | IDHmut-codel | LGm2 | LGr3 | Codel |
| TCGA-FG-8188-01A | TCGA-FG-8188 | 41 | 0:LIVING | Male | Primary | Oligoastrocytoma G2 | Astrocytoma, IDH-mutant lower grade |  |  | DDRm2 | DDRr1 | No combined CNA | Non-codel | Mutant | Methylated | WT | 20 | IDHmut-non-codel | LGm2 | LGr3 | G-CIMP-high |
| TCGA-FG-8189-01B | TCGA-FG-8189 | 33 | 0:LIVING | Female | Primary | Oligodendroglioma G2 | Astrocytoma, IDH-mutant lower grade |  |  | DDRm1 | DDRr3 | No combined CNA | Non-codel | Mutant | Methylated | WT | NA | IDHmut-non-codel | LGm6 | LGr2 | G-CIMP-high |
| TCGA-FG-8191-01A | TCGA-FG-8191 | 30 | 0:LIVING | Male | Primary | Oligodendroglioma G3 | Astrocytoma, IDH-mutant lower grade |  |  | DDRm2 | DDRr1 | No combined CNA | Non-codel | Mutant | Unmethylated | WT | 15 | IDHmut-non-codel | LGm2 | LGr3 | G-CIMP-high |
| TCGA-FG-A4MT-01A | TCGA-FG-A4MT | 27 | 0:LIVING | Female | Primary | Oligodendroglioma G2 | Astrocytoma, IDH-mutant lower grade |  |  | DDRm2 | DDRr1 | No combined CNA | Non-codel | Mutant | Methylated | WT | 17 | IDHmut-non-codel | LGm2 | LGr3 | G-CIMP-high |
| TCGA-FG-A4MT-02A | TCGA-FG-A4MT | NA | NA | NA | Recurrent | Astrocytoma G3 | Astrocytoma, IDH-mutant lower grade |  |  | DDRm2 | DDRr3 | NA | NA | NA | NA | NA | NA | NA | NA | NA | NA |
| TCGA-FG-A4MU-01B | TCGA-FG-A4MU | 58 | 0:LIVING | Male | Primary | Oligoastrocytoma G3 | Glioblastoma G4 | EGFR amp, +7/-10, TERTp mut |  | DDRm1 | DDRr4 | Gain chr 7 & loss chr 10 | Non-codel | WT | Methylated | Mutant | 52 | IDHwt | LGm5 | LGr4 | Mesenchymal-like |
| TCGA-FG-A4MW-01A | TCGA-FG-A4MW | 63 | 0:LIVING | Male | Primary | Oligoastrocytoma G3 | Glioblastoma G4 | EGFR amp, +7/-10, TERTp mut |  | DDRm1 | DDRr4 | Gain chr 7 & loss chr 10 | Non-codel | WT | Methylated | Mutant | 54 | IDHwt | LGm4 | LGr4 | Classic-like |
| TCGA-FG-A4MX-01A | TCGA-FG-A4MX | 47 | 0:LIVING | Male | Primary | Astrocytoma G2 | Astrocytoma, IDH-mutant lower grade |  |  | DDRm2 | DDRr1 | No combined CNA | Non-codel | Mutant | Methylated | WT | 15 | IDHmut-non-codel | LGm2 | LGr3 | G-CIMP-high |
| TCGA-FG-A4MY-01A | TCGA-FG-A4MY | 44 | 0:LIVING | Female | Primary | Oligoastrocytoma G2 | Astrocytoma, IDH-mutant lower grade |  |  | DDRm2 | DDRr1 | No combined CNA | Non-codel | Mutant | Methylated | WT | 21 | IDHmut-non-codel | LGm2 | LGr3 | G-CIMP-high |
| TCGA-FG-A60J-01A | TCGA-FG-A60J | 47 | 0:LIVING | Female | Primary | Oligoastrocytoma G2 | Astrocytoma, IDH-mutant lower grade |  |  | DDRm2 | DDRr1 | No combined CNA | Non-codel | Mutant | Methylated | Mutant | 27 | IDHmut-non-codel | LGm2 | LGr3 | G-CIMP-high |
| TCGA-FG-A60K-01A | TCGA-FG-A60K | 34 | 0:LIVING | Female | Primary | Oligoastrocytoma G2 | Oligodendroglioma, IDH-mutant |  |  | DDRm2 | DDRr2 | No combined CNA | Codel | Mutant | Methylated | Mutant | 17 | IDHmut-codel | LGm2 | LGr1 | Codel |
| TCGA-FG-A60L-01A | TCGA-FG-A60L | 34 | 0:LIVING | Female | Primary | Astrocytoma G2 | Astrocytoma, IDH-mutant lower grade |  |  | DDRm2 | DDRr1 | No combined CNA | Non-codel | Mutant | Methylated | NA | 19 | IDHmut-non-codel | LGm2 | LGr3 | G-CIMP-high |
| TCGA-FG-A6IZ-01A | TCGA-FG-A6IZ | 60 | 0:LIVING | Male | Primary | Oligodendroglioma G2 | Oligodendroglioma, IDH-mutant |  |  | DDRm2 | DDRr1 | No combined CNA | Codel | Mutant | Methylated | NA | 33 | IDHmut-codel | LGm3 | LGr1 | Codel |
| TCGA-FG-A6J1-01A | TCGA-FG-A6J1 | 44 | 0:LIVING | Female | Primary | Oligodendroglioma G2 | Oligodendroglioma, IDH-mutant |  |  | DDRm2 | DDRr1 | No combined CNA | Codel | Mutant | Methylated | NA | 9 | IDHmut-codel | LGm3 | LGr1 | Codel |
| TCGA-FG-A6J3-01A | TCGA-FG-A6J3 | 52 | 0:LIVING | Female | Primary | Astrocytoma G3 | Astrocytoma, IDH-mutant lower grade |  |  | DDRm2 | DDRr1 | No combined CNA | Non-codel | Mutant | Methylated | NA | 53 | IDHmut-non-codel | LGm1 | LGr3 | G-CIMP-low |
| TCGA-FG-A70Y-01A | TCGA-FG-A70Y | 20 | 0:LIVING | Female | Primary | Oligodendroglioma G2 | Astrocytoma, IDH-mutant G4 | CDKN2A/Bdel |  | DDRm2 | DDRr1 | No combined CNA | Non-codel | Mutant | Methylated | NA | 12 | IDHmut-non-codel | LGm2 | LGr3 | G-CIMP-high |
| TCGA-FG-A70Z-01A | TCGA-FG-A70Z | 53 | 0:LIVING | Female | Primary | Oligoastrocytoma G3 | Glioblastoma G4 | +7/-10, EGFR amp |  | DDRm1 | DDRr4 | Gain chr 7 & loss chr 10 | Non-codel | WT | Methylated | NA | 39 | IDHwt | LGm5 | LGr4 | Mesenchymal-like |
| TCGA-FG-A710-01A | TCGA-FG-A710 | 50 | 0:LIVING | Female | Primary | Oligodendroglioma G2 | Oligodendroglioma, IDH-mutant |  |  | DDRm2 | DDRr2 | No combined CNA | Codel | Mutant | Methylated | NA | 15 | IDHmut-codel | LGm3 | LGr1 | Codel |
| TCGA-FG-A711 | TCGA-74-6573 | NA | NA | NA | Normal | Normal | Normal |  |  | NA | NA | NA | NA | NA | NA | NA | NA | NA | NA | NA | NA |
| TCGA-FG-A711-01A | TCGA-FG-A711 | 33 | 0:LIVING | Female | Primary | Oligodendroglioma G2 | Astrocytoma, IDH-mutant lower grade |  |  | DDRm2 | DDRr1 | No combined CNA | Non-codel | Mutant | Unmethylated | NA | 27 | IDHmut-non-codel | LGm2 | LGr3 | G-CIMP-high |
| TCGA-FG-A713-01A | TCGA-FG-A713 | 74 | 0:LIVING | Female | Primary | Oligoastrocytoma G2 | Oligodendroglioma, IDH-mutant |  |  | DDRm2 | DDRr3 | No combined CNA | Codel | Mutant | Methylated | NA | 17 | IDHmut-codel | LGm3 | LGr2 | Codel |
| TCGA-FG-A87N-01A | TCGA-FG-A87N | 37 | 0:LIVING | Female | Primary | Astrocytoma G3 | Astrocytoma, IDH-mutant G4 | CDKN2A/Bdel |  | DDRm2 | DDRr4 | No combined CNA | Non-codel | Mutant | Methylated | NA | 22 | IDHmut-non-codel | LGm1 | LGr1 | G-CIMP-low |
| TCGA-FG-A87Q-01A | TCGA-FG-A87Q | 61 | 0:LIVING | Female | Primary | Astrocytoma G3 | Glioblastoma G4 | +7/-10, EGFR amp |  | DDRm1 | DDRr1 | Gain chr 7 & loss chr 10 | Non-codel | WT | Methylated | NA | 48 | IDHwt | LGm4 | LGr4 | Classic-like |
| TCGA-FN-7833-01A | TCGA-FN-7833 | 25 | 0:LIVING | Male | Primary | Oligoastrocytoma G3 | Astrocytoma, IDH-mutant lower grade |  |  | DDRm2 | DDRr1 | No combined CNA | Non-codel | Mutant | Methylated | WT | 16 | IDHmut-non-codel | LGm2 | LGr3 | G-CIMP-high |
| TCGA-HT-7467-01A | TCGA-HT-7467 | 54 | 0:LIVING | Male | Primary | Oligodendroglioma G2 | Oligodendroglioma, IDH-mutant |  |  | DDRm2 | DDRr3 | No combined CNA | Codel | Mutant | Methylated | Mutant | 19 | IDHmut-codel | LGm2 | LGr2 | Codel |
| TCGA-HT-7468-01A | TCGA-HT-7468 | 30 | 0:LIVING | Male | Primary | Oligodendroglioma G3 | Oligodendroglioma, IDH-mutant |  |  | DDRm2 | DDRr2 | No combined CNA | Codel | Mutant | Methylated | Mutant | 13 | IDHmut-codel | LGm3 | LGr1 | Codel |
| TCGA-HT-7469-01A | TCGA-HT-7469 | 30 | 1:DECEASED | Male | Primary | Oligodendroglioma G3 | Glioblastoma G4 | Necrosis |  | NA | NA | No combined CNA | Non-codel | WT | Methylated | WT | 23 | IDHwt | LGm6 | LGr4 | PA-like |
| TCGA-HT-7470-01A | TCGA-HT-7470 | 37 | 0:LIVING | Male | Primary | Oligodendroglioma G3 | Astrocytoma, IDH-mutant lower grade |  |  | DDRm2 | DDRr3 | No combined CNA | Non-codel | Mutant | Methylated | WT | 30 | IDHmut-non-codel | LGm2 | LGr2 | G-CIMP-high |
| TCGA-HT-7471-01A | TCGA-HT-7471 | 37 | 0:LIVING | Female | Primary | Oligodendroglioma G3 | Oligodendroglioma, IDH-mutant |  |  | DDRm2 | DDRr2 | No combined CNA | Codel | Mutant | Methylated | Mutant | 12 | IDHmut-codel | LGm3 | LGr1 | Codel |
| TCGA-HT-7472 | TCGA-06-AABW | NA | NA | NA | Normal | Normal | Normal |  |  | NA | NA | NA | NA | NA | NA | NA | NA | NA | NA | NA | NA |
| TCGA-HT-7472-01A | TCGA-HT-7472 | 38 | 0:LIVING | Male | Primary | Oligodendroglioma G2 | Astrocytoma, IDH-mutant lower grade |  |  | DDRm2 | DDRr1 | No combined CNA | Non-codel | Mutant | Methylated | WT | 15 | IDHmut-non-codel | LGm2 | LGr3 | G-CIMP-high |
| TCGA-HT-7473-01A | TCGA-HT-7473 | 28 | 0:LIVING | Male | Primary | Oligoastrocytoma G2 | Astrocytoma, IDH-mutant lower grade |  |  | DDRm2 | DDRr1 | No combined CNA | Non-codel | Mutant | Unmethylated | WT | 12 | IDHmut-non-codel | LGm2 | LGr3 | G-CIMP-high |
| TCGA-HT-7474-01A | TCGA-HT-7474 | 52 | 0:LIVING | Male | Primary | Oligoastrocytoma G2 | Astrocytoma, IDH-mutant lower grade |  |  | DDRm2 | DDRr3 | No combined CNA | Non-codel | Mutant | Methylated | WT | 13 | IDHmut-non-codel | LGm2 | LGr2 | G-CIMP-high |
| TCGA-HT-7475-01A | TCGA-HT-7475 | 67 | 0:LIVING | Male | Primary | Oligoastrocytoma G3 | Astrocytoma, IDH-mutant lower grade |  |  | DDRm2 | DDRr3 | No combined CNA | Non-codel | Mutant | Methylated | WT | 37 | IDHmut-non-codel | LGm2 | LGr3 | G-CIMP-high |
| TCGA-HT-7476-01A | TCGA-HT-7476 | 26 | 0:LIVING | Male | Primary | Astrocytoma G2 | Astrocytoma, IDH-mutant G4 | CDKN2A/Bdel |  | DDRm2 | DDRr3 | No combined CNA | Non-codel | Mutant | Methylated | WT | 15 | IDHmut-non-codel | LGm2 | LGr3 | G-CIMP-high |
| TCGA-HT-7477-01B | TCGA-HT-7477 | 62 | 0:LIVING | Male | Primary | Astrocytoma G3 | Astrocytoma, IDH-mutant G4 | CDKN2A/Bdel |  | DDRm2 | DDRr4 | No combined CNA | Non-codel | Mutant | Methylated | WT | 34 | IDHmut-non-codel | LGm1 | LGr3 | G-CIMP-high |
| TCGA-HT-7478-01A | TCGA-HT-7478 | 36 | 0:LIVING | Male | Primary | Astrocytoma G2 | Astrocytoma, IDH-mutant lower grade |  |  | DDRm2 | DDRr1 | No combined CNA | Non-codel | Mutant | Unmethylated | WT | 18 | IDHmut-non-codel | LGm2 | LGr3 | G-CIMP-high |
| TCGA-HT-7479-01A | TCGA-HT-7479 | 44 | 0:LIVING | Male | Primary | Astrocytoma G3 | Astrocytoma, IDH-mutant lower grade |  |  | DDRm2 | DDRr1 | No combined CNA | Non-codel | Mutant | Methylated | Mutant | 16 | IDHmut-non-codel | LGm2 | LGr3 | G-CIMP-high |
| TCGA-HT-7480-01A | TCGA-HT-7480 | 33 | 0:LIVING | Male | Primary | Oligodendroglioma G2 | Oligodendroglioma, IDH-mutant |  |  | DDRm2 | DDRr2 | No combined CNA | Codel | Mutant | Methylated | Mutant | 15 | IDHmut-codel | LGm3 | LGr2 | Codel |
| TCGA-HT-7481-01A | TCGA-HT-7481 | 39 | 0:LIVING | Male | Primary | Oligodendroglioma G2 | Oligodendroglioma, IDH-mutant |  |  | DDRm2 | DDRr3 | No combined CNA | Codel | Mutant | Methylated | Mutant | 19 | IDHmut-codel | LGm2 | LGr2 | Codel |
| TCGA-HT-7482-01A | TCGA-HT-7482 | 18 | 0:LIVING | Female | Primary | Oligoastrocytoma G2 | Astrocytoma, IDH-mutant lower grade |  |  | DDRm2 | DDRr1 | No combined CNA | Non-codel | Mutant | Methylated | WT | 12 | IDHmut-non-codel | LGm2 | LGr3 | G-CIMP-high |
| TCGA-HT-7483-01A | TCGA-HT-7483 | 14 | 0:LIVING | Male | Primary | Oligoastrocytoma G2 | Astrocytoma, IDH-mutant lower grade |  |  | DDRm2 | DDRr1 | No combined CNA | Non-codel | Mutant | Unmethylated | WT | 9 | IDHmut-non-codel | LGm2 | LGr3 | G-CIMP-high |
| TCGA-HT-7485-01A | TCGA-HT-7485 | 42 | 0:LIVING | Male | Primary | Astrocytoma G2 | Astrocytoma, IDH-mutant lower grade |  |  | DDRm2 | DDRr1 | No combined CNA | Non-codel | Mutant | Methylated | WT | 11 | IDHmut-non-codel | LGm2 | LGr3 | G-CIMP-high |
| TCGA-HT-7601-01A | TCGA-HT-7601 | 30 | 0:LIVING | Female | Primary | Astrocytoma G3 | Astrocytoma, IDH-mutant lower grade |  |  | DDRm2 | DDRr1 | No combined CNA | Non-codel | Mutant | Methylated | WT | 14 | IDHmut-non-codel | LGm1 | LGr3 | G-CIMP-high |
| TCGA-HT-7602 | TCGA-06-0675 | NA | NA | NA | Normal | Normal | Normal |  |  | NA | NA | NA | NA | NA | NA | NA | NA | NA | NA | NA | NA |
| TCGA-HT-7602-01A | TCGA-HT-7602 | 21 | 0:LIVING | Male | Primary | Oligodendroglioma G2 | Astrocytoma, IDH-mutant lower grade |  |  | DDRm2 | DDRr1 | No combined CNA | Non-codel | Mutant | Methylated | WT | 3 | IDHmut-non-codel | LGm2 | LGr3 | G-CIMP-high |
| TCGA-HT-7603 | TCGA-06-0678 | NA | NA | NA | Normal | Normal | Normal |  |  | NA | NA | NA | NA | NA | NA | NA | NA | NA | NA | NA | NA |
| TCGA-HT-7603-01A | TCGA-HT-7603 | 29 | 0:LIVING | Male | Primary | Oligodendroglioma G2 | Astrocytoma, IDH-mutant lower grade |  |  | DDRm2 | DDRr3 | No combined CNA | Non-codel | Mutant | Methylated | WT | 21 | IDHmut-non-codel | LGm2 | LGr2 | G-CIMP-high |
| TCGA-HT-7604-01A | TCGA-HT-7604 | 50 | 0:LIVING | Male | Primary | Astrocytoma G2 | Astrocytoma, IDH-mutant lower grade |  |  | DDRm2 | DDRr1 | No combined CNA | Non-codel | Mutant | Methylated | WT | 27 | IDHmut-non-codel | LGm2 | LGr3 | G-CIMP-high |
| TCGA-HT-7605-01A | TCGA-HT-7605 | 38 | 0:LIVING | Male | Primary | Oligodendroglioma G2 | Oligodendroglioma, IDH-mutant |  |  | DDRm2 | DDRr3 | No combined CNA | Codel | Mutant | Methylated | Mutant | 15 | IDHmut-codel | LGm2 | LGr2 | Codel |
| TCGA-HT-7606-01A | TCGA-HT-7606 | 30 | 0:LIVING | Female | Primary | Astrocytoma G2 | Astrocytoma, IDH-mutant lower grade |  |  | DDRm2 | DDRr4 | No combined CNA | Non-codel | Mutant | Unmethylated | WT | 20 | IDHmut-non-codel | LGm2 | LGr1 | G-CIMP-high |
| TCGA-HT-7607-01A | TCGA-HT-7607 | 61 | 1:DECEASED | Female | Primary | Astrocytoma G2 | Oligodendroglioma, IDH-mutant |  |  | DDRm2 | DDRr3 | No combined CNA | Codel | Mutant | Methylated | Mutant | 20 | IDHmut-codel | LGm3 | LGr2 | Codel |
| TCGA-HT-7608-01A | TCGA-HT-7608 | 61 | 0:LIVING | Male | Primary | Oligoastrocytoma G2 | Oligodendroglioma, IDH-mutant |  |  | DDRm2 | DDRr2 | No combined CNA | Codel | Mutant | Methylated | Mutant | 12 | IDHmut-codel | LGm3 | LGr3 | Codel |
| TCGA-HT-7609-01A | TCGA-HT-7609 | 34 | 0:LIVING | Male | Primary | Oligoastrocytoma G3 | Astrocytoma, IDH-mutant G4 | Vascular proliferation, necrosis |  | DDRm2 | DDRr1 | No combined CNA | Non-codel | Mutant | Methylated | WT | 15 | IDHmut-non-codel | LGm2 | LGr3 | G-CIMP-high |
| TCGA-HT-7610-01A | TCGA-HT-7610 | 25 | 0:LIVING | Female | Primary | Oligoastrocytoma G2 | Astrocytoma, IDH-mutant lower grade |  |  | DDRm2 | DDRr3 | No combined CNA | Non-codel | Mutant | Methylated | WT | 11 | IDHmut-non-codel | LGm2 | LGr2 | G-CIMP-high |
| TCGA-HT-7611-01A | TCGA-HT-7611 | 36 | 0:LIVING | Male | Primary | Oligoastrocytoma G2 | Astrocytoma, IDH-mutant lower grade |  |  | DDRm2 | DDRr1 | No combined CNA | Non-codel | Mutant | Methylated | WT | 20 | IDHmut-non-codel | LGm2 | LGr3 | G-CIMP-high |
| TCGA-HT-7616-01A | TCGA-HT-7616 | 75 | 1:DECEASED | Male | Primary | Oligodendroglioma G3 | Oligodendroglioma, IDH-mutant |  |  | DDRm2 | DDRr2 | No combined CNA | Codel | Mutant | Methylated | Mutant | 31 | IDHmut-codel | LGm3 | LGr1 | Codel |
| TCGA-HT-7620-01A | TCGA-HT-7620 | 40 | 0:LIVING | Male | Primary | Oligodendroglioma G3 | Oligodendroglioma, IDH-mutant |  |  | DDRm2 | DDRr2 | No combined CNA | Codel | Mutant | Methylated | Mutant | 15 | IDHmut-codel | LGm2 | LGr3 | Codel |
| TCGA-HT-7676 | TCGA-06-0680 | NA | NA | NA | Normal | Normal | Normal |  |  | NA | NA | NA | NA | NA | NA | NA | NA | NA | NA | NA | NA |
| TCGA-HT-7676-01A | TCGA-HT-7676 | 26 | 0:LIVING | Male | Primary | Oligodendroglioma G2 | Astrocytoma, IDH-mutant lower grade |  |  | DDRm2 | DDRr1 | No combined CNA | Non-codel | Mutant | Unmethylated | WT | 12 | IDHmut-non-codel | LGm2 | LGr3 | G-CIMP-high |
| TCGA-HT-7677-01A | TCGA-HT-7677 | 53 | 0:LIVING | Male | Primary | Oligodendroglioma G3 | Oligodendroglioma, IDH-mutant |  |  | DDRm2 | DDRr2 | No combined CNA | Codel | Mutant | Methylated | Mutant | 17 | IDHmut-codel | LGm3 | LGr1 | Codel |
| TCGA-HT-7680-01A | TCGA-HT-7680 | 32 | 0:LIVING | Female | Primary | Astrocytoma G2 | Glioma, IDH-wildtype NEC |  |  | DDRm1 | DDRr1 | No combined CNA | Non-codel | WT | Unmethylated | WT | 1 | IDHwt | LGm6 | LGr4 | PA-like |
| TCGA-HT-7681-01A | TCGA-HT-7681 | 29 | 0:LIVING | Female | Primary | Oligoastrocytoma G2 | Oligodendroglioma, IDH-mutant |  |  | DDRm2 | DDRr3 | No combined CNA | Codel | Mutant | Methylated | Mutant | 11 | IDHmut-codel | LGm2 | LGr2 | Codel |
| TCGA-HT-7684-01A | TCGA-HT-7684 | 58 | 0:LIVING | Male | Primary | Oligoastrocytoma G3 | Astrocytoma, IDH-mutant G4 | Vascular proliferation |  | DDRm2 | DDRr1 | No combined CNA | Non-codel | Mutant | Methylated | Mutant | 25 | IDHmut-non-codel | LGm2 | LGr3 | G-CIMP-high |
| TCGA-HT-7686-01A | TCGA-HT-7686 | 29 | 0:LIVING | Female | Primary | Astrocytoma G3 | Astrocytoma, IDH-mutant lower grade |  |  | DDRm2 | DDRr1 | No combined CNA | Non-codel | Mutant | Methylated | WT | 12 | IDHmut-non-codel | LGm1 | LGr3 | G-CIMP-high |
| TCGA-HT-7687-01A | TCGA-HT-7687 | 74 | 0:LIVING | Male | Primary | Oligodendroglioma G3 | Oligodendroglioma, IDH-mutant |  |  | DDRm2 | DDRr2 | No combined CNA | Codel | Mutant | Methylated | Mutant | 23 | IDHmut-codel | LGm3 | LGr2 | Codel |
| TCGA-HT-7688-01A | TCGA-HT-7688 | 59 | 0:LIVING | Male | Primary | Oligodendroglioma G3 | Astrocytoma, IDH-mutant lower grade |  |  | DDRm2 | DDRr3 | No combined CNA | Non-codel | Mutant | Methylated | WT | 39 | IDHmut-non-codel | LGm2 | LGr2 | G-CIMP-high |
| TCGA-HT-7689 | TCGA-06-0681 | NA | NA | NA | Normal | Normal | Normal |  |  | NA | NA | NA | NA | NA | NA | NA | NA | NA | NA | NA | NA |
| TCGA-HT-7689-01A | TCGA-HT-7689 | 58 | 0:LIVING | Female | Primary | Oligodendroglioma G2 | Astrocytoma, IDH-mutant lower grade |  |  | DDRm2 | DDRr1 | No combined CNA | Non-codel | Mutant | Methylated | WT | 32 | IDHmut-non-codel | LGm1 | LGr3 | G-CIMP-high |
| TCGA-HT-7690-01A | TCGA-HT-7690 | 29 | 0:LIVING | Male | Primary | Oligoastrocytoma G3 | Astrocytoma, IDH-mutant lower grade |  |  | DDRm2 | DDRr1 | No combined CNA | Non-codel | Mutant | Methylated | WT | 15 | IDHmut-non-codel | LGm2 | LGr3 | G-CIMP-high |
| TCGA-HT-7691-01A | TCGA-HT-7691 | 31 | 0:LIVING | Female | Primary | Astrocytoma G2 | Glioma, IDH-wildtype NEC |  |  | DDRm1 | DDRr1 | No combined CNA | Non-codel | WT | Unmethylated | WT | 5 | IDHwt | LGm6 | LGr4 | PA-like |
| TCGA-HT-7692-01A | TCGA-HT-7692 | 43 | 0:LIVING | Male | Primary | Oligoastrocytoma G2 | Oligodendroglioma, IDH-mutant |  |  | DDRm2 | DDRr2 | No combined CNA | Codel | Mutant | Methylated | Mutant | 14 | IDHmut-codel | LGm2 | LGr1 | Codel |
| TCGA-HT-7693-01A | TCGA-HT-7693 | 51 | 0:LIVING | Female | Primary | Oligodendroglioma G2 | Astrocytoma, IDH-mutant lower grade |  |  | DDRm2 | DDRr1 | No combined CNA | Non-codel | Mutant | Methylated | WT | 19 | IDHmut-non-codel | LGm2 | LGr3 | G-CIMP-high |
| TCGA-HT-7694-01A | TCGA-HT-7694 | 60 | 0:LIVING | Male | Primary | Oligodendroglioma G3 | Oligodendroglioma, IDH-mutant |  |  | DDRm2 | DDRr3 | No combined CNA | Codel | Mutant | Methylated | Mutant | 19 | IDHmut-codel | LGm3 | LGr2 | Codel |
| TCGA-HT-7695-01A | TCGA-HT-7695 | 29 | 0:LIVING | Female | Primary | Oligodendroglioma G2 | Oligodendroglioma, IDH-mutant |  |  | DDRm2 | DDRr3 | No combined CNA | Codel | Mutant | Methylated | Mutant | 8 | IDHmut-codel | LGm2 | LGr2 | Codel |
| TCGA-HT-7854-01A | TCGA-HT-7854 | 62 | 0:LIVING | Male | Primary | Astrocytoma G2 | Glioblastoma G4 | TERTp mut |  | DDRm1 | DDRr1 | No combined CNA | Non-codel | WT | Unmethylated | Mutant | 20 | IDHwt | LGm6 | LGr4 | PA-like |
| TCGA-HT-7855-01A | TCGA-HT-7855 | 39 | 0:LIVING | Male | Primary | Astrocytoma G3 | Astrocytoma, IDH-mutant G4 | CDKN2A/Bdel |  | DDRm2 | DDRr1 | No combined CNA | Non-codel | Mutant | Methylated | WT | 28 | IDHmut-non-codel | LGm2 | LGr3 | G-CIMP-high |
| TCGA-HT-7856-01A | TCGA-HT-7856 | 35 | 0:LIVING | Male | Primary | Oligodendroglioma G3 | Oligodendroglioma, IDH-mutant |  |  | DDRm2 | DDRr3 | No combined CNA | Codel | Mutant | Methylated | Mutant | 4 | IDHmut-codel | LGm2 | LGr2 | Codel |
| TCGA-HT-7857-01A | TCGA-HT-7857 | 24 | 0:LIVING | Female | Primary | Astrocytoma G3 | Glioma, IDH-wildtype NEC |  |  | DDRm1 | DDRr4 | No combined CNA | Non-codel | WT | Unmethylated | WT | 12 | IDHwt | LGm6 | LGr4 | PA-like |
| TCGA-HT-7858-01A | TCGA-HT-7858 | 28 | 0:LIVING | Male | Primary | Astrocytoma G2 | Astrocytoma, IDH-mutant lower grade |  |  | DDRm2 | DDRr1 | No combined CNA | Non-codel | Mutant | Methylated | WT | 9 | IDHmut-non-codel | LGm2 | LGr3 | G-CIMP-high |
| TCGA-HT-7860-01A | TCGA-HT-7860 | 60 | 0:LIVING | Female | Primary | Astrocytoma G3 | Glioblastoma G4 | +7/-10, TERTp mut |  | DDRm1 | DDRr4 | Gain chr 7 & loss chr 10 | Non-codel | WT | Methylated | Mutant | 56 | IDHwt | LGm4 | LGr4 | Classic-like |
| TCGA-HT-7873-01B | TCGA-HT-7873 | 29 | 0:LIVING | Male | Primary | Oligoastrocytoma G2 | Astrocytoma, IDH-mutant lower grade |  |  | DDRm2 | DDRr1 | No combined CNA | Non-codel | Mutant | Methylated | WT | 18 | IDHmut-non-codel | LGm2 | LGr3 | G-CIMP-high |
| TCGA-HT-7874-01A | TCGA-HT-7874 | 41 | 0:LIVING | Female | Primary | Oligodendroglioma G3 | Oligodendroglioma, IDH-mutant |  |  | DDRm2 | DDRr3 | No combined CNA | Codel | Mutant | Methylated | Mutant | 12 | IDHmut-codel | LGm2 | LGr2 | Codel |
| TCGA-HT-7875-01A | TCGA-HT-7875 | 56 | 0:LIVING | Male | Primary | Oligodendroglioma G2 | Oligodendroglioma, IDH-mutant |  |  | DDRm2 | DDRr2 | No combined CNA | Codel | Mutant | Methylated | Mutant | 21 | IDHmut-codel | LGm3 | LGr2 | Codel |
| TCGA-HT-7877-01A | TCGA-HT-7877 | 20 | 0:LIVING | Female | Primary | Oligodendroglioma G2 | Oligodendroglioma, IDH-mutant |  |  | DDRm2 | DDRr2 | No combined CNA | Codel | Mutant | Methylated | Mutant | 8 | IDHmut-codel | LGm2 | LGr2 | Codel |
| TCGA-HT-7879-01A | TCGA-HT-7879 | 31 | 0:LIVING | Male | Primary | Oligoastrocytoma G3 | Astrocytoma, IDH-mutant lower grade |  |  | DDRm2 | DDRr1 | No combined CNA | Non-codel | Mutant | Methylated | WT | 12 | IDHmut-non-codel | LGm2 | LGr3 | G-CIMP-high |
| TCGA-HT-7880-01A | TCGA-HT-7880 | 30 | 0:LIVING | Male | Primary | Oligoastrocytoma G2 | Astrocytoma, IDH-mutant lower grade |  |  | DDRm2 | DDRr3 | No combined CNA | Non-codel | Mutant | Methylated | WT | 4 | IDHmut-non-codel | LGm1 | LGr2 | G-CIMP-high |
| TCGA-HT-7881-01A | TCGA-HT-7881 | 38 | 0:LIVING | Male | Primary | Oligodendroglioma G2 | Oligodendroglioma, IDH-mutant |  |  | DDRm2 | DDRr3 | No combined CNA | Codel | Mutant | Methylated | Mutant | 7 | IDHmut-codel | LGm2 | LGr2 | Codel |
| TCGA-HT-7882-01A | TCGA-HT-7882 | 66 | 1:DECEASED | Male | Primary | Oligodendroglioma G3 | Glioblastoma G4 | TERTp mut |  | DDRm1 | DDRr4 | No combined CNA | Non-codel | WT | Methylated | Mutant | 26 | IDHwt | LGm5 | LGr4 | Mesenchymal-like |
| TCGA-HT-7884-01B | TCGA-HT-7884 | 44 | 0:LIVING | Female | Primary | Astrocytoma G2 | Astrocytoma, IDH-mutant lower grade |  |  | DDRm2 | DDRr1 | No combined CNA | Non-codel | Mutant | Methylated | WT | 21 | IDHmut-non-codel | LGm2 | LGr3 | G-CIMP-high |
| TCGA-HT-7902-01A | TCGA-HT-7902 | 30 | 0:LIVING | Female | Primary | Oligoastrocytoma G2 | Astrocytoma, IDH-mutant lower grade |  |  | DDRm2 | DDRr1 | No combined CNA | Non-codel | Mutant | Methylated | WT | 11 | IDHmut-non-codel | LGm1 | LGr3 | G-CIMP-high |
| TCGA-HT-8010-01A | TCGA-HT-8010 | 64 | 0:LIVING | Female | Primary | Oligodendroglioma G2 | Oligodendroglioma, IDH-mutant |  |  | DDRm2 | DDRr3 | No combined CNA | Codel | Mutant | Methylated | WT | 8 | IDHmut-codel | LGm2 | LGr2 | Codel |
| TCGA-HT-8011-01A | TCGA-HT-8011 | 55 | 0:LIVING | Male | Primary | Astrocytoma G3 | Glioblastoma G4 | +7/-10, TERTp mut |  | DDRm1 | DDRr4 | Gain chr 7 & loss chr 10 | Non-codel | WT | Unmethylated | Mutant | 35 | IDHwt | LGm5 | LGr4 | Mesenchymal-like |
| TCGA-HT-8012-01A | TCGA-HT-8012 | 30 | 0:LIVING | Female | Primary | Oligodendroglioma G2 | Oligodendroglioma, IDH-mutant |  |  | DDRm2 | DDRr2 | No combined CNA | Codel | Mutant | Methylated | Mutant | 11 | IDHmut-codel | LGm3 | LGr1 | Codel |
| TCGA-HT-8013-01A | TCGA-HT-8013 | 37 | 1:DECEASED | Female | Primary | Oligoastrocytoma G2 | Astrocytoma, IDH-mutant G4 | Necrosis |  | DDRm2 | DDRr1 | No combined CNA | Non-codel | Mutant | Methylated | WT | 20 | IDHmut-non-codel | LGm2 | LGr3 | G-CIMP-high |
| TCGA-HT-8015-01B | TCGA-HT-8015 | 21 | 0:LIVING | Male | Primary | Astrocytoma G2 | Glioma, IDH-wildtype NEC |  |  | DDRm1 | DDRr3 | No combined CNA | Non-codel | WT | Unmethylated | WT | 1 | IDHwt | LGm6 | LGr2 | PA-like |
| TCGA-HT-8018-01A | TCGA-HT-8018 | 40 | 0:LIVING | Female | Primary | Oligoastrocytoma G2 | Astrocytoma, IDH-mutant lower grade |  |  | DDRm2 | DDRr3 | No combined CNA | Non-codel | Mutant | Methylated | WT | 11 | IDHmut-non-codel | LGm1 | LGr2 | G-CIMP-high |
| TCGA-HT-8019-01A | TCGA-HT-8019 | 34 | 0:LIVING | Female | Primary | Oligodendroglioma G3 | Glioma, IDH-wildtype NEC |  |  | DDRm1 | DDRr3 | No combined CNA | Non-codel | WT | Unmethylated | WT | 1 | IDHwt | LGm6 | LGr2 | PA-like |
| TCGA-HT-8104-01A | TCGA-HT-8104 | 51 | 0:LIVING | Female | Primary | Astrocytoma G3 | Glioblastoma G4 | EGFR amp, +7/-10, TERTp mut |  | DDRm1 | DDRr4 | Gain chr 7 & loss chr 10 | Non-codel | WT | Unmethylated | Mutant | 49 | IDHwt | LGm4 | LGr4 | Classic-like |
| TCGA-HT-8105-01A | TCGA-HT-8105 | 54 | 0:LIVING | Male | Primary | Oligodendroglioma G3 | Oligodendroglioma, IDH-mutant |  |  | DDRm2 | DDRr2 | No combined CNA | Codel | Mutant | Methylated | Mutant | 37 | IDHmut-codel | LGm3 | LGr2 | Codel |
| TCGA-HT-8106-01A | TCGA-HT-8106 | 53 | 0:LIVING | Male | Primary | Astrocytoma G3 | Astrocytoma, IDH-mutant lower grade |  |  | DDRm2 | DDRr1 | No combined CNA | Non-codel | Mutant | Methylated | WT | 15 | IDHmut-non-codel | LGm1 | LGr3 | G-CIMP-high |
| TCGA-HT-8107-01A | TCGA-HT-8107 | 62 | 0:LIVING | Male | Primary | Oligodendroglioma G2 | Glioma, IDH-wildtype NEC |  |  | DDRm1 | DDRr3 | No combined CNA | Non-codel | WT | Methylated | WT | 1 | IDHwt | LGm6 | LGr2 | PA-like |
| TCGA-HT-8108-01A | TCGA-HT-8108 | 26 | 0:LIVING | Female | Primary | Oligodendroglioma G2 | Astrocytoma, IDH-mutant lower grade |  |  | DDRm2 | DDRr1 | No combined CNA | Non-codel | Mutant | Methylated | WT | 15 | IDHmut-non-codel | LGm2 | LGr3 | G-CIMP-high |
| TCGA-HT-8109-01A | TCGA-HT-8109 | 64 | 0:LIVING | Male | Primary | Oligodendroglioma G3 | Oligodendroglioma, IDH-mutant |  |  | DDRm2 | DDRr3 | No combined CNA | Codel | Mutant | Methylated | Mutant | 19 | IDHmut-codel | LGm3 | LGr2 | Codel |
| TCGA-HT-8110-01A | TCGA-HT-8110 | 57 | 0:LIVING | Male | Primary | Astrocytoma G3 | Glioblastoma G4 | EGFR amp, +7/-10, TERTp mut |  | DDRm1 | DDRr4 | Gain chr 7 & loss chr 10 | Non-codel | WT | Methylated | Mutant | 26 | IDHwt | LGm5 | LGr4 | Mesenchymal-like |
| TCGA-HT-8111-01A | TCGA-HT-8111 | 32 | 0:LIVING | Male | Primary | Oligoastrocytoma G3 | Astrocytoma, IDH-mutant lower grade |  |  | DDRm2 | DDRr1 | No combined CNA | Non-codel | Mutant | Methylated | WT | 7 | IDHmut-non-codel | LGm1 | LGr3 | G-CIMP-high |
| TCGA-HT-8113-01A | TCGA-HT-8113 | 49 | 0:LIVING | Female | Primary | Oligodendroglioma G2 | Astrocytoma, IDH-mutant lower grade |  |  | DDRm2 | DDRr3 | No combined CNA | Non-codel | Mutant | Methylated | Mutant | 7 | IDHmut-non-codel | LGm2 | LGr2 | Codel |
| TCGA-HT-8114-01A | TCGA-HT-8114 | 36 | 0:LIVING | Male | Primary | Oligoastrocytoma G3 | Astrocytoma, IDH-mutant G4 | Focal necrosis |  | DDRm2 | DDRr1 | No combined CNA | Non-codel | Mutant | Methylated | WT | 12 | IDHmut-non-codel | LGm2 | LGr3 | G-CIMP-high |
| TCGA-HT-8558-01A | TCGA-HT-8558 | 29 | 0:LIVING | Female | Primary | Oligodendroglioma G2 | Glioma, IDH-wildtype NOS |  |  | DDRm1 | DDRr3 | NA | Non-codel | WT | Unmethylated | WT | 2 | IDHwt | LGm6 | LGr2 | PA-like |
| TCGA-HT-8563-01A | TCGA-HT-8563 | 30 | 0:LIVING | Female | Primary | Astrocytoma G3 | Astrocytoma, IDH-mutant lower grade |  |  | DDRm2 | DDRr1 | No combined CNA | Non-codel | Mutant | Unmethylated | WT | 18 | IDHmut-non-codel | LGm1 | LGr4 | G-CIMP-high |
| TCGA-HT-8564-01A | TCGA-HT-8564 | 47 | 0:LIVING | Male | Primary | Astrocytoma G3 | Glioma, IDH-wildtype NEC |  |  | DDRm1 | DDRr3 | No combined CNA | Non-codel | WT | Unmethylated | WT | 377 | IDHwt | LGm6 | LGr2 | PA-like |
| TCGA-HT-A4DS-01A | TCGA-HT-A4DS | 55 | 0:LIVING | Female | Primary | Astrocytoma G3 | Glioblastoma G4 | TERTp mut |  | DDRm1 | DDRr1 | No combined CNA | Non-codel | WT | Unmethylated | Mutant | 25 | IDHwt | LGm5 | LGr3 | Mesenchymal-like |
| TCGA-HT-A4DV-01A | TCGA-HT-A4DV | 51 | 0:LIVING | Female | Primary | Oligodendroglioma G3 | Oligodendroglioma, IDH-mutant |  |  | DDRm2 | DDRr1 | No combined CNA | Codel | Mutant | Methylated | Mutant | 8 | IDHmut-codel | LGm2 | LGr1 | Codel |
| TCGA-HT-A5R5-01A | TCGA-HT-A5R5 | 33 | 0:LIVING | Female | Primary | Oligodendroglioma G2 | Astrocytoma, IDH-mutant lower grade |  |  | DDRm2 | DDRr1 | No combined CNA | Non-codel | Mutant | Methylated | WT | 14 | IDHmut-non-codel | LGm2 | LGr4 | G-CIMP-high |
| TCGA-HT-A5R7-01A | TCGA-HT-A5R7 | 33 | 0:LIVING | Female | Primary | Astrocytoma G3 | Astrocytoma, IDH-mutant lower grade |  |  | DDRm2 | DDRr3 | No combined CNA | Non-codel | Mutant | Methylated | WT | 13 | IDHmut-non-codel | LGm1 | LGr2 | G-CIMP-high |
| TCGA-HT-A5R9-01A | TCGA-HT-A5R9 | 48 | 0:LIVING | Female | Primary | Oligodendroglioma G3 | Oligodendroglioma, IDH-mutant |  |  | DDRm2 | DDRr2 | No combined CNA | Codel | Mutant | Methylated | Mutant | 26 | IDHmut-codel | LGm3 | LGr1 | Codel |
| TCGA-HT-A5RA-01A | TCGA-HT-A5RA | 65 | 0:LIVING | Female | Primary | Astrocytoma G3 | Glioblastoma G4 | EGFR amp, TERTp mut |  | DDRm1 | DDRr4 | No combined CNA | Non-codel | WT | Unmethylated | Mutant | 36 | IDHwt | LGm5 | LGr4 | Mesenchymal-like |
| TCGA-HT-A5RB-01A | TCGA-HT-A5RB | 24 | 0:LIVING | Male | Primary | Astrocytoma G2 | Astrocytoma, IDH-mutant lower grade |  |  | DDRm2 | DDRr1 | No combined CNA | Non-codel | Mutant | Methylated | WT | 14 | IDHmut-non-codel | LGm2 | LGr3 | G-CIMP-high |
| TCGA-HT-A5RC-01A | TCGA-HT-A5RC | 70 | 1:DECEASED | Female | Primary | Astrocytoma G3 | Glioblastoma G4 | +7/-10, EGFR amp |  | DDRm1 | DDRr4 | Gain chr 7 & loss chr 10 | Non-codel | WT | Unmethylated | WT | 39 | IDHwt | LGm4 | LGr4 | Classic-like |
| TCGA-HT-A614-01A | TCGA-HT-A614 | 47 | 0:LIVING | Male | Primary | Oligoastrocytoma G2 | Astrocytoma, IDH-mutant lower grade |  |  | DDRm2 | DDRr1 | No combined CNA | Non-codel | Mutant | Methylated | WT | 24 | IDHmut-non-codel | LGm1 | LGr3 | G-CIMP-high |
| TCGA-HT-A615-01A | TCGA-HT-A615 | 38 | 0:LIVING | Female | Primary | Oligodendroglioma G2 | Oligodendroglioma, IDH-mutant |  |  | DDRm2 | DDRr2 | No combined CNA | Codel | Mutant | Methylated | Mutant | 27 | IDHmut-codel | LGm2 | LGr1 | Codel |
| TCGA-HT-A616-01A | TCGA-HT-A616 | 36 | 0:LIVING | Female | Primary | Astrocytoma G2 | Astrocytoma, IDH-mutant lower grade |  |  | DDRm2 | DDRr3 | No combined CNA | Non-codel | Mutant | Methylated | WT | 14 | IDHmut-non-codel | LGm2 | LGr2 | G-CIMP-high |
| TCGA-HT-A617-01A | TCGA-HT-A617 | 47 | 0:LIVING | Male | Primary | Oligodendroglioma G2 | Glioblastoma G4 | +7/-10, TERTp mut |  | DDRm1 | DDRr1 | Gain chr 7 & loss chr 10 | Non-codel | WT | Unmethylated | Mutant | 10 | IDHwt | LGm4 | LGr4 | Classic-like |
| TCGA-HT-A618-01A | TCGA-HT-A618 | 37 | 0:LIVING | Female | Primary | Astrocytoma G3 | Astrocytoma, IDH-mutant lower grade |  |  | DDRm2 | DDRr1 | No combined CNA | Non-codel | Mutant | Methylated | WT | 11 | IDHmut-non-codel | LGm1 | LGr3 | G-CIMP-high |
| TCGA-HT-A619-01A | TCGA-HT-A619 | 51 | 0:LIVING | Female | Primary | Oligodendroglioma G3 | Oligodendroglioma, IDH-mutant |  |  | DDRm2 | DDRr2 | No combined CNA | Codel | Mutant | Methylated | Mutant | 42 | IDHmut-codel | LGm3 | LGr1 | Codel |
| TCGA-HT-A61A-01A | TCGA-HT-A61A | 20 | 0:LIVING | Female | Primary | Oligodendroglioma G2 | Astrocytoma, IDH-mutant lower grade |  |  | DDRm2 | DDRr3 | NA | Non-codel | Mutant | Methylated | Mutant | 4 | IDHmut-non-codel | LGm1 | NA | G-CIMP-high |
| TCGA-HT-A61B-01A | TCGA-HT-A61B | NA | NA | NA | Primary | Astrocytoma G3 | Astrocytoma, IDH-mutant lower grade |  |  | DDRm2 | DDRr1 | No combined CNA | Non-codel | Mutant | Methylated | WT | 32 | IDHmut-non-codel | LGm2 | LGr3 | G-CIMP-high |
| TCGA-HT-A61C-01A | TCGA-HT-A61C | 66 | 0:LIVING | Male | Primary | Oligodendroglioma G3 | Glioblastoma G4 | +7/-10, TERTp mut |  | DDRm1 | DDRr4 | Gain chr 7 & loss chr 10 | Non-codel | WT | Unmethylated | Mutant | 33 | IDHwt | LGm5 | LGr1 | Mesenchymal-like |
| TCGA-HT-A74H-01A | TCGA-HT-A74H | 62 | 0:LIVING | Male | Primary | Astrocytoma G3 | Glioblastoma G4 | -0.7 |  | DDRm1 | DDRr1 | Gain chr 7 & loss chr 10 | Non-codel | WT | Unmethylated | NA | 34 | IDHwt | LGm5 | LGr4 | Mesenchymal-like |
| TCGA-HT-A74J-01A | TCGA-HT-A74J | 33 | 0:LIVING | Male | Primary | Oligoastrocytoma G2 | Astrocytoma, IDH-mutant G4 | CDKN2A/Bdel |  | DDRm2 | DDRr1 | No combined CNA | Non-codel | Mutant | Unmethylated | NA | 18 | IDHmut-non-codel | LGm2 | LGr3 | G-CIMP-high |
| TCGA-HT-A74K-01A | TCGA-HT-A74K | NA | NA | NA | Primary | Oligodendroglioma G3 | Oligodendroglioma, IDH-mutant |  |  | DDRm2 | DDRr1 | No combined CNA | Codel | Mutant | Methylated | NA | 23 | IDHmut-codel | LGm3 | LGr1 | Codel |
| TCGA-HT-A74L-01A | TCGA-HT-A74L | NA | NA | NA | Primary | Oligoastrocytoma G2 | Oligodendroglioma, IDH-mutant |  |  | DDRm2 | DDRr2 | No combined CNA | Codel | Mutant | Methylated | NA | 11 | IDHmut-codel | LGm2 | LGr1 | Codel |
| TCGA-HT-A74O-01A | TCGA-HT-A74O | 34 | 0:LIVING | Male | Primary | Astrocytoma G3 | Astrocytoma, IDH-mutant G4 | Necrosis |  | DDRm2 | DDRr1 | No combined CNA | Non-codel | Mutant | Methylated | NA | 10 | IDHmut-non-codel | LGm2 | LGr3 | G-CIMP-high |
| TCGA-HW-7486-01A | TCGA-HW-7486 | 37 | 0:LIVING | Male | Primary | Oligodendroglioma G2 | Oligodendroglioma, IDH-mutant |  |  | DDRm2 | DDRr2 | No combined CNA | Codel | Mutant | Methylated | Mutant | 6 | IDHmut-codel | LGm2 | LGr3 | Codel |
| TCGA-HW-7487-01A | TCGA-HW-7487 | 39 | 0:LIVING | Male | Primary | Oligodendroglioma G2 | Oligodendroglioma, IDH-mutant |  |  | DDRm2 | DDRr3 | No combined CNA | Codel | Mutant | Methylated | Mutant | 13 | IDHmut-codel | LGm3 | LGr2 | Codel |
| TCGA-HW-7489-01A | TCGA-HW-7489 | 38 | 0:LIVING | Male | Primary | Oligoastrocytoma G2 | Astrocytoma, IDH-mutant lower grade |  |  | DDRm2 | DDRr3 | No combined CNA | Non-codel | Mutant | Methylated | WT | 10 | IDHmut-non-codel | LGm2 | LGr2 | G-CIMP-high |
| TCGA-HW-7490-01A | TCGA-HW-7490 | 41 | 0:LIVING | Male | Primary | Astrocytoma G2 | Astrocytoma, IDH-mutant lower grade |  |  | DDRm2 | DDRr1 | No combined CNA | Non-codel | Mutant | Methylated | WT | 26 | IDHmut-non-codel | LGm2 | LGr3 | G-CIMP-high |
| TCGA-HW-7491-01A | TCGA-HW-7491 | 35 | 0:LIVING | Male | Primary | Oligodendroglioma G2 | Oligodendroglioma, IDH-mutant |  |  | DDRm2 | DDRr2 | No combined CNA | Codel | Mutant | Unmethylated | Mutant | 11 | IDHmut-codel | LGm3 | LGr1 | Codel |
| TCGA-HW-7493-01A | TCGA-HW-7493 | 40 | 0:LIVING | Female | Primary | Astrocytoma G2 | Glioma NOS |  |  | DDRm2 | DDRr1 | No combined CNA | Non-codel | NA | Methylated | NA | NA | NA | LGm2 | LGr4 | NA |
| TCGA-HW-7495-01A | TCGA-HW-7495 | 45 | 0:LIVING | Female | Primary | Oligodendroglioma G2 | Oligodendroglioma, IDH-mutant |  |  | DDRm2 | DDRr3 | No combined CNA | Codel | Mutant | Methylated | Mutant | 10 | IDHmut-codel | LGm2 | LGr2 | Codel |
| TCGA-HW-8319-01A | TCGA-HW-8319 | 34 | 0:LIVING | Female | Primary | Astrocytoma G3 | Astrocytoma, IDH-mutant lower grade |  |  | DDRm2 | DDRr1 | No combined CNA | Non-codel | Mutant | Unmethylated | WT | 24 | IDHmut-non-codel | LGm1 | LGr3 | G-CIMP-high |
| TCGA-HW-8320-01A | TCGA-HW-8320 | 36 | 0:LIVING | Male | Primary | Astrocytoma G3 | Astrocytoma, IDH-mutant lower grade |  |  | DDRm2 | DDRr1 | No combined CNA | Non-codel | Mutant | Methylated | WT | 14 | IDHmut-non-codel | LGm2 | LGr3 | G-CIMP-high |
| TCGA-HW-8321-01A | TCGA-HW-8321 | 31 | 0:LIVING | Male | Primary | Astrocytoma G3 | Astrocytoma, IDH-mutant lower grade |  |  | DDRm2 | DDRr1 | No combined CNA | Non-codel | Mutant | Methylated | WT | 21 | IDHmut-non-codel | LGm2 | LGr3 | G-CIMP-high |
| TCGA-HW-8322-01A | TCGA-HW-8322 | 39 | 0:LIVING | Male | Primary | Oligodendroglioma G2 | Oligodendroglioma, IDH-mutant |  |  | DDRm2 | DDRr2 | No combined CNA | Codel | Mutant | Methylated | Mutant | 14 | IDHmut-codel | LGm2 | LGr1 | Codel |
| TCGA-HW-A5KJ-01A | TCGA-HW-A5KJ | 68 | 0:LIVING | Male | Primary | Oligodendroglioma G3 | Oligodendroglioma, IDH-mutant |  |  | DDRm2 | DDRr1 | No combined CNA | Codel | Mutant | Methylated | Mutant | 35 | IDHmut-codel | LGm3 | LGr1 | Codel |
| TCGA-HW-A5KK-01A | TCGA-HW-A5KK | 64 | 1:DECEASED | Male | Primary | Astrocytoma G3 | Glioblastoma G4 | EGFR amp, TERTp mut |  | DDRm1 | DDRr1 | No combined CNA | Non-codel | WT | Methylated | Mutant | 22 | IDHwt | LGm5 | LGr4 | Mesenchymal-like |
| TCGA-HW-A5KL-01A | TCGA-HW-A5KL | 42 | 0:LIVING | Female | Primary | Astrocytoma G2 | Astrocytoma, IDH-mutant lower grade |  |  | DDRm2 | DDRr1 | No combined CNA | Non-codel | Mutant | Methylated | WT | 18 | IDHmut-non-codel | LGm2 | LGr3 | G-CIMP-high |
| TCGA-HW-A5KM-01A | TCGA-HW-A5KM | 35 | 0:LIVING | Male | Primary | Astrocytoma G2 | Astrocytoma, IDH-mutant lower grade |  |  | DDRm2 | DDRr1 | No combined CNA | Non-codel | Mutant | Methylated | WT | 13 | IDHmut-non-codel | LGm2 | LGr3 | G-CIMP-high |
| TCGA-IK-7675-01A | TCGA-IK-7675 | 43 | 1:DECEASED | Male | Primary | Oligodendroglioma G2 | Astrocytoma, IDH-mutant lower grade |  |  | DDRm2 | DDRr1 | No combined CNA | Non-codel | Mutant | Methylated | WT | 37 | IDHmut-non-codel | LGm1 | LGr3 | G-CIMP-high |
| TCGA-IK-8125-01A | TCGA-IK-8125 | 62 | 0:LIVING | Male | Primary | Oligoastrocytoma G3 | Oligodendroglioma, IDH-mutant |  |  | DDRm2 | DDRr2 | No combined CNA | Codel | Mutant | Methylated | Mutant | 34 | IDHmut-codel | LGm3 | LGr2 | Codel |
| TCGA-KT-A74X-01A | TCGA-KT-A74X | 26 | 0:LIVING | Male | Primary | Oligoastrocytoma G3 | Oligodendroglioma, IDH-mutant |  |  | DDRm2 | DDRr2 | No combined CNA | Codel | Mutant | Methylated | NA | 6 | IDHmut-codel | LGm2 | LGr1 | Codel |
| TCGA-KT-A7W1-01A | TCGA-KT-A7W1 | 45 | 0:LIVING | Female | Primary | Astrocytoma G3 | Glioblastoma G4 | +7/-10, EGFR amp |  | DDRm1 | DDRr4 | Gain chr 7 & loss chr 10 | Non-codel | WT | Methylated | NA | 33 | IDHwt | LGm4 | LGr4 | Classic-like |
| TCGA-OX-A56R-01A | TCGA-OX-A56R | 68 | 1:DECEASED | Male | Primary | Glioblastoma G4 | Glioblastoma G4 |  |  | NA | NA | Gain chr 7 & loss chr 10 | Non-codel | WT | Methylated | NA | 33 | NA | LGm5 | NA | Mesenchymal-like |
| TCGA-P5-A5ET-01A | TCGA-P5-A5ET | 27 | 0:LIVING | Male | Primary | Oligodendroglioma G2 | Oligodendroglioma, IDH-mutant |  |  | DDRm2 | DDRr2 | No combined CNA | Codel | Mutant | Methylated | Mutant | 17 | IDHmut-codel | LGm2 | LGr1 | Codel |
| TCGA-P5-A5EU-01A | TCGA-P5-A5EU | 35 | 0:LIVING | Male | Primary | Astrocytoma G3 | Astrocytoma, IDH-mutant G4 | CDKN2A/Bdel |  | DDRm2 | DDRr1 | No combined CNA | Non-codel | Mutant | Unmethylated | WT | 19 | IDHmut-non-codel | LGm1 | LGr3 | G-CIMP-low |
| TCGA-P5-A5EV-01A | TCGA-P5-A5EV | 39 | 0:LIVING | Male | Primary | Astrocytoma G2 | Astrocytoma, IDH-mutant lower grade |  |  | DDRm2 | DDRr1 | No combined CNA | Non-codel | Mutant | Methylated | WT | 53 | IDHmut-non-codel | LGm2 | LGr3 | G-CIMP-high |
| TCGA-P5-A5EW-01A | TCGA-P5-A5EW | 20 | 0:LIVING | Female | Primary | Astrocytoma G2 | Astrocytoma, IDH-mutant lower grade |  |  | DDRm2 | DDRr1 | No combined CNA | Non-codel | Mutant | Methylated | WT | 12 | IDHmut-non-codel | LGm2 | LGr3 | G-CIMP-high |
| TCGA-P5-A5EX-01A | TCGA-P5-A5EX | 41 | 0:LIVING | Female | Primary | Oligodendroglioma G3 | Oligodendroglioma, IDH-mutant |  |  | DDRm2 | DDRr1 | No combined CNA | Codel | Mutant | Methylated | Mutant | 18 | IDHmut-codel | LGm2 | LGr4 | Codel |
| TCGA-P5-A5EY-01A | TCGA-P5-A5EY | 21 | 0:LIVING | Male | Primary | Astrocytoma G2 | Glioma, IDH-wildtype NEC |  |  | DDRm1 | DDRr3 | No combined CNA | Non-codel | WT | Unmethylated | WT | 2 | IDHwt | LGm6 | LGr2 | PA-like |
| TCGA-P5-A5EZ-01A | TCGA-P5-A5EZ | 39 | 0:LIVING | Male | Primary | Astrocytoma G3 | Astrocytoma, IDH-mutant lower grade |  |  | DDRm2 | DDRr1 | No combined CNA | Non-codel | Mutant | Methylated | WT | 19 | IDHmut-non-codel | LGm2 | LGr3 | G-CIMP-high |
| TCGA-P5-A5F0-01A | TCGA-P5-A5F0 | 33 | 0:LIVING | Male | Primary | Oligodendroglioma G2 | Oligodendroglioma, IDH-mutant |  |  | DDRm2 | DDRr3 | No combined CNA | Codel | Mutant | Methylated | Mutant | 19 | IDHmut-codel | LGm3 | LGr2 | Codel |
| TCGA-P5-A5F1-01A | TCGA-P5-A5F1 | 30 | 0:LIVING | Male | Primary | Astrocytoma G2 | Astrocytoma, IDH-mutant lower grade |  |  | DDRm2 | DDRr1 | No combined CNA | Non-codel | Mutant | Unmethylated | WT | 12 | IDHmut-non-codel | LGm2 | LGr3 | G-CIMP-high |
| TCGA-P5-A5F2-01A | TCGA-P5-A5F2 | 36 | 0:LIVING | Female | Primary | Astrocytoma G2 | Astrocytoma, IDH-mutant lower grade |  |  | DDRm2 | DDRr1 | No combined CNA | Non-codel | Mutant | Methylated | WT | 19 | IDHmut-non-codel | LGm2 | LGr4 | G-CIMP-high |
| TCGA-P5-A5F4-01A | TCGA-P5-A5F4 | 35 | 0:LIVING | Female | Primary | Oligodendroglioma G3 | Astrocytoma, IDH-mutant lower grade |  |  | DDRm2 | DDRr3 | No combined CNA | Non-codel | Mutant | Methylated | WT | 25 | IDHmut-non-codel | LGm2 | LGr2 | G-CIMP-high |
| TCGA-P5-A5F6-01A | TCGA-P5-A5F6 | 26 | 0:LIVING | Male | Primary | Oligodendroglioma G2 | Glioma, IDH-wildtype NOS |  |  | DDRm1 | DDRr1 | No combined CNA | Non-codel | WT | Unmethylated | WT | 0 | IDHwt | LGm6 | LGr4 | PA-like |
| TCGA-P5-A72U-01A | TCGA-P5-A72U | 71 | 0:LIVING | Female | Primary | Oligodendroglioma G3 | Glioblastoma G4 | -0.7 |  | DDRm1 | DDRr4 | Gain chr 7 & loss chr 10 | Non-codel | WT | Methylated | NA | 41 | IDHwt | LGm4 | LGr4 | Classic-like |
| TCGA-P5-A72W-01A | TCGA-P5-A72W | 35 | 0:LIVING | Male | Primary | Astrocytoma G3 | Astrocytoma, IDH-mutant lower grade |  |  | DDRm2 | DDRr1 | No combined CNA | Non-codel | Mutant | Unmethylated | NA | 33 | IDHmut-non-codel | LGm2 | LGr3 | G-CIMP-high |
| TCGA-P5-A72X-01A | TCGA-P5-A72X | 21 | 0:LIVING | Male | Primary | Astrocytoma G3 | Astrocytoma, IDH-mutant lower grade |  |  | DDRm2 | DDRr1 | No combined CNA | Non-codel | Mutant | Methylated | NA | 8 | IDHmut-non-codel | LGm2 | LGr3 | G-CIMP-high |
| TCGA-P5-A72Z-01A | TCGA-P5-A72Z | 64 | 0:LIVING | Female | Primary | Oligodendroglioma G3 | Oligodendroglioma, IDH-mutant |  |  | DDRm2 | DDRr2 | No combined CNA | Codel | Mutant | Methylated | NA | 21 | IDHmut-codel | LGm3 | LGr1 | Codel |
| TCGA-P5-A730-01A | TCGA-P5-A730 | 22 | 0:LIVING | Male | Primary | Oligoastrocytoma G3 | Oligodendroglioma, IDH-mutant |  |  | DDRm2 | DDRr2 | No combined CNA | Codel | Mutant | Methylated | NA | 18 | IDHmut-codel | LGm3 | LGr1 | Codel |
| TCGA-P5-A731-01A | TCGA-P5-A731 | 59 | 0:LIVING | Female | Primary | Oligoastrocytoma G2 | Astrocytoma, IDH-mutant lower grade |  |  | DDRm2 | DDRr1 | No combined CNA | Non-codel | Mutant | Methylated | NA | 19 | IDHmut-non-codel | LGm2 | LGr3 | G-CIMP-high |
| TCGA-P5-A733-01A | TCGA-P5-A733 | 52 | 0:LIVING | Female | Primary | Astrocytoma G2 | Astrocytoma, IDH-mutant lower grade |  |  | DDRm2 | DDRr1 | No combined CNA | Non-codel | Mutant | Methylated | NA | 23 | IDHmut-non-codel | LGm2 | LGr3 | G-CIMP-high |
| TCGA-P5-A735-01A | TCGA-P5-A735 | NA | NA | NA | Primary | Astrocytoma G2 | Astrocytoma, IDH-mutant lower grade |  |  | DDRm2 | DDRr1 | No combined CNA | Non-codel | Mutant | Methylated | NA | 21 | IDHmut-non-codel | LGm2 | LGr3 | G-CIMP-high |
| TCGA-P5-A736-01A | TCGA-P5-A736 | NA | NA | NA | Primary | Astrocytoma G3 | Astrocytoma, IDH-mutant lower grade |  |  | DDRm2 | DDRr1 | No combined CNA | Non-codel | Mutant | Methylated | NA | 14 | IDHmut-non-codel | LGm2 | LGr3 | G-CIMP-high |
| TCGA-P5-A737-01A | TCGA-P5-A737 | NA | NA | NA | Primary | Oligoastrocytoma G2 | Oligodendroglioma, IDH-mutant |  |  | DDRm2 | DDRr2 | No combined CNA | Codel | Mutant | Methylated | NA | 12 | IDHmut-codel | LGm3 | LGr1 | Codel |
| TCGA-P5-A77W-01A | TCGA-P5-A77W | NA | NA | NA | Primary | Oligoastrocytoma G3 | Oligodendroglioma, IDH-mutant |  |  | DDRm2 | DDRr2 | No combined CNA | Codel | Mutant | Methylated | NA | 21 | IDHmut-codel | LGm3 | LGr1 | Codel |
| TCGA-P5-A77X-01A | TCGA-P5-A77X | NA | NA | NA | Primary | Oligoastrocytoma G2 | Oligodendroglioma, IDH-mutant |  |  | DDRm2 | DDRr2 | No combined CNA | Codel | Mutant | Methylated | NA | 20 | IDHmut-codel | LGm3 | LGr2 | Codel |
| TCGA-P5-A780-01A | TCGA-P5-A780 | NA | NA | NA | Primary | Astrocytoma G3 | Astrocytoma, IDH-mutant G4 | CDKN2A/Bdel |  | DDRm2 | DDRr1 | No combined CNA | Non-codel | Mutant | Methylated | NA | 30 | IDHmut-non-codel | LGm2 | LGr3 | G-CIMP-high |
| TCGA-P5-A781-01A | TCGA-P5-A781 | NA | NA | NA | Primary | Astrocytoma G3 | Oligodendroglioma, IDH-mutant |  |  | DDRm2 | DDRr2 | No combined CNA | Codel | Mutant | Methylated | NA | 8 | IDHmut-codel | LGm3 | LGr1 | Codel |
| TCGA-QH-A65R-01A | TCGA-QH-A65R | 38 | 0:LIVING | Female | Primary | Oligodendroglioma G3 | Oligodendroglioma, IDH-mutant |  |  | DDRm2 | DDRr2 | No combined CNA | Codel | Mutant | Methylated | NA | 21 | IDHmut-codel | LGm3 | LGr1 | Codel |
| TCGA-QH-A65S-01A | TCGA-QH-A65S | 32 | 0:LIVING | Female | Primary | Oligoastrocytoma G2 | Astrocytoma, IDH-mutant lower grade |  |  | DDRm2 | DDRr1 | No combined CNA | Non-codel | Mutant | Methylated | WT | 15 | IDHmut-non-codel | LGm1 | LGr3 | G-CIMP-high |
| TCGA-QH-A65V-01A | TCGA-QH-A65V | 43 | 0:LIVING | Female | Primary | Oligodendroglioma G2 | Oligodendroglioma, IDH-mutant |  |  | DDRm2 | DDRr1 | No combined CNA | Codel | Mutant | Methylated | Mutant | 19 | IDHmut-codel | LGm2 | LGr1 | Codel |
| TCGA-QH-A65X-01A | TCGA-QH-A65X | 28 | 0:LIVING | Female | Primary | Oligoastrocytoma G3 | Oligodendroglioma, IDH-mutant |  |  | DDRm2 | DDRr1 | No combined CNA | Codel | Mutant | Methylated | NA | 17 | IDHmut-codel | LGm3 | LGr1 | Codel |
| TCGA-QH-A65Z-01A | TCGA-QH-A65Z | 54 | 0:LIVING | Male | Primary | Oligodendroglioma G2 | Oligodendroglioma, IDH-mutant |  |  | DDRm2 | DDRr2 | No combined CNA | Codel | Mutant | Methylated | Mutant | 21 | IDHmut-codel | LGm3 | LGr1 | Codel |
| TCGA-QH-A6CS-01A | TCGA-QH-A6CS | 41 | 0:LIVING | Male | Primary | Astrocytoma G3 | Glioblastoma G4 | -0.7 |  | DDRm1 | DDRr1 | Gain chr 7 & loss chr 10 | Non-codel | WT | Unmethylated | NA | 10 | IDHwt | LGm6 | NA | PA-like |
| TCGA-QH-A6CU-01A | TCGA-QH-A6CU | 52 | 0:LIVING | Female | Primary | Oligodendroglioma G3 | Oligodendroglioma, IDH-mutant |  |  | DDRm2 | DDRr1 | No combined CNA | Codel | Mutant | Methylated | NA | 11 | IDHmut-codel | LGm2 | LGr1 | Codel |
| TCGA-QH-A6CV-01A | TCGA-QH-A6CV | 51 | 0:LIVING | Male | Primary | Oligoastrocytoma G3 | Glioblastoma G4 | +7/-10, EGFR amp |  | DDRm1 | DDRr4 | Gain chr 7 & loss chr 10 | Non-codel | WT | Unmethylated | NA | 36 | IDHwt | LGm4 | LGr4 | Classic-like |
| TCGA-QH-A6CW-01A | TCGA-QH-A6CW | 43 | 0:LIVING | Male | Primary | Oligoastrocytoma G3 | Astrocytoma, IDH-mutant lower grade |  |  | DDRm2 | DDRr1 | No combined CNA | Non-codel | Mutant | Methylated | NA | 26 | IDHmut-non-codel | LGm2 | LGr3 | G-CIMP-high |
| TCGA-QH-A6CX-01A | TCGA-QH-A6CX | 66 | 0:LIVING | Male | Primary | Astrocytoma G2 | Glioblastoma G4 | EGFR amp |  | DDRm1 | DDRr1 | No combined CNA | Non-codel | WT | Unmethylated | NA | 21 | IDHwt | LGm5 | LGr4 | Mesenchymal-like |
| TCGA-QH-A6CY-01A | TCGA-QH-A6CY | 38 | 0:LIVING | Male | Primary | Oligoastrocytoma G3 | Oligodendroglioma, IDH-mutant |  |  | DDRm2 | DDRr3 | No combined CNA | Codel | Mutant | Methylated | NA | 10 | IDHmut-codel | LGm3 | LGr2 | Codel |
| TCGA-QH-A6CZ-01A | TCGA-QH-A6CZ | 38 | 0:LIVING | Male | Primary | Oligoastrocytoma G2 | Oligodendroglioma, IDH-mutant |  |  | DDRm2 | DDRr3 | No combined CNA | Codel | Mutant | Methylated | NA | 18 | IDHmut-codel | LGm3 | LGr2 | Codel |
| TCGA-QH-A6X3-01A | TCGA-QH-A6X3 | 27 | 0:LIVING | Male | Primary | Oligoastrocytoma G2 | Astrocytoma, IDH-mutant lower grade |  |  | DDRm2 | DDRr1 | No combined CNA | Non-codel | Mutant | Methylated | NA | 18 | IDHmut-non-codel | LGm2 | LGr3 | G-CIMP-high |
| TCGA-QH-A6X4-01A | TCGA-QH-A6X4 | 47 | 0:LIVING | Male | Primary | Oligoastrocytoma G3 | Oligodendroglioma, IDH-mutant |  |  | DDRm2 | DDRr2 | No combined CNA | Codel | Mutant | Methylated | NA | 22 | IDHmut-codel | LGm3 | LGr1 | Codel |
| TCGA-QH-A6X5-01A | TCGA-QH-A6X5 | 58 | 0:LIVING | Female | Primary | Oligoastrocytoma G2 | Oligodendroglioma, IDH-mutant |  |  | DDRm2 | DDRr2 | No combined CNA | Codel | Mutant | Methylated | NA | 12 | IDHmut-codel | LGm3 | LGr1 | Codel |
| TCGA-QH-A6X8-01A | TCGA-QH-A6X8 | 56 | 0:LIVING | Female | Primary | Oligodendroglioma G3 | Oligodendroglioma, IDH-mutant |  |  | DDRm2 | DDRr2 | No combined CNA | Codel | Mutant | Methylated | NA | 21 | IDHmut-codel | LGm3 | LGr1 | Codel |
| TCGA-QH-A6X9-01A | TCGA-QH-A6X9 | 73 | 0:LIVING | Female | Primary | Oligodendroglioma G2 | Astrocytoma, IDH-mutant G4 | CDKN2A/Bdel |  | DDRm2 | DDRr1 | No combined CNA | Non-codel | Mutant | Methylated | NA | 30 | IDHmut-non-codel | LGm2 | LGr3 | G-CIMP-high |
| TCGA-QH-A6XA-01A | TCGA-QH-A6XA | 23 | 0:LIVING | Female | Primary | Oligoastrocytoma G2 | Astrocytoma, IDH-mutant lower grade |  |  | DDRm2 | DDRr1 | No combined CNA | Non-codel | Mutant | Methylated | NA | 9 | IDHmut-non-codel | LGm2 | LGr3 | G-CIMP-high |
| TCGA-QH-A6XC-01A | TCGA-QH-A6XC | 48 | 0:LIVING | Male | Primary | Astrocytoma G3 | Glioblastoma G4 | +7/-10, EGFR amp |  | DDRm1 | DDRr4 | Gain chr 7 & loss chr 10 | Non-codel | WT | Methylated | NA | 40 | IDHwt | LGm4 | LGr4 | Classic-like |
| TCGA-QH-A86X-01A | TCGA-QH-A86X | 33 | 0:LIVING | Male | Primary | Oligodendroglioma G2 | Oligodendroglioma, IDH-mutant |  |  | DDRm2 | DDRr2 | No combined CNA | Codel | Mutant | Methylated | NA | 10 | IDHmut-codel | LGm2 | LGr1 | Codel |
| TCGA-QH-A870-01A | TCGA-QH-A870 | 38 | 0:LIVING | Female | Primary | Oligoastrocytoma G3 | Astrocytoma, IDH-mutant lower grade |  |  | DDRm2 | DDRr1 | No combined CNA | Non-codel | Mutant | Methylated | NA | 14 | IDHmut-non-codel | LGm2 | LGr3 | G-CIMP-high |
| TCGA-R8-A6MK-01A | TCGA-R8-A6MK | 40 | 0:LIVING | Male | Primary | Oligodendroglioma G2 | Oligodendroglioma, IDH-mutant |  |  | DDRm2 | DDRr2 | No combined CNA | Codel | Mutant | Methylated | NA | 15 | IDHmut-codel | LGm2 | LGr3 | Codel |
| TCGA-R8-A6ML-01A | TCGA-R8-A6ML | 52 | 0:LIVING | Male | Primary | Oligodendroglioma G3 | Oligodendroglioma, IDH-mutant |  |  | DDRm2 | DDRr2 | No combined CNA | Codel | Mutant | Methylated | NA | 18 | IDHmut-codel | LGm3 | LGr1 | Codel |
| TCGA-R8-A6MO-01A | TCGA-R8-A6MO | 53 | 0:LIVING | Female | Primary | Oligodendroglioma G2 | Oligodendroglioma, IDH-mutant |  |  | DDRm2 | DDRr2 | No combined CNA | Codel | Mutant | Methylated | NA | 21 | IDHmut-codel | LGm3 | LGr1 | Codel |
| TCGA-R8-A6YH-01A | TCGA-R8-A6YH | NA | NA | NA | Primary | Astrocytoma G3 | Astrocytoma, IDH-mutant lower grade |  |  | DDRm2 | DDRr1 | No combined CNA | Non-codel | Mutant | Unmethylated | NA | 6 | IDHmut-non-codel | LGm2 | LGr3 | G-CIMP-high |
| TCGA-R8-A73M-01A | TCGA-R8-A73M | 48 | 0:LIVING | Female | Primary | Oligodendroglioma G2 | Oligodendroglioma, IDH-mutant |  |  | DDRm2 | DDRr1 | No combined CNA | Codel | Mutant | Methylated | NA | 22 | IDHmut-codel | LGm3 | LGr1 | Codel |
| TCGA-RR-A6KA-01A | TCGA-RR-A6KA | 72 | 1:DECEASED | Female | Primary | Glioblastoma G4 | Glioblastoma G4 |  |  | NA | NA | No combined CNA | Non-codel | WT | Methylated | NA | 30 | NA | LGm5 | NA | Mesenchymal-like |
| TCGA-RR-A6KB-01A | TCGA-RR-A6KB | NA | NA | NA | Primary | Glioblastoma G4 | Glioblastoma G4 |  |  | NA | NA | Gain chr 7 & loss chr 10 | Non-codel | WT | Unmethylated | NA | 30 | NA | LGm5 | NA | Mesenchymal-like |
| TCGA-RR-A6KC-01A | TCGA-RR-A6KC | 55 | 1:DECEASED | Male | Primary | Glioblastoma G4 | Glioblastoma G4 |  |  | NA | NA | Gain chr 7 & loss chr 10 | Non-codel | WT | Unmethylated | NA | 36 | NA | LGm4 | NA | Classic-like |
| TCGA-RY-A83X-01A | TCGA-RY-A83X | NA | NA | NA | Primary | Oligodendroglioma G2 | Oligodendroglioma, IDH-mutant |  |  | DDRm2 | DDRr2 | No combined CNA | Codel | Mutant | Methylated | NA | 11 | IDHmut-codel | LGm3 | LGr1 | Codel |
| TCGA-RY-A83Y-01A | TCGA-RY-A83Y | NA | NA | NA | Primary | Oligodendroglioma G2 | Oligodendroglioma, IDH-mutant |  |  | DDRm2 | DDRr1 | No combined CNA | Codel | Mutant | Methylated | NA | 26 | IDHmut-codel | LGm3 | LGr1 | Codel |
| TCGA-RY-A83Z-01A | TCGA-RY-A83Z | NA | NA | NA | Primary | Astrocytoma G3 | Astrocytoma, IDH-mutant G4 | CDKN2A/Bdel |  | DDRm2 | DDRr1 | No combined CNA | Non-codel | Mutant | Methylated | NA | 38 | IDHmut-non-codel | LGm1 | LGr3 | G-CIMP-low |
| TCGA-RY-A840-01A | TCGA-RY-A840 | NA | NA | NA | Primary | Oligodendroglioma G3 | Oligodendroglioma, IDH-mutant |  |  | DDRm2 | DDRr2 | No combined CNA | Codel | Mutant | Methylated | NA | 11 | IDHmut-codel | LGm3 | LGr1 | Codel |
| TCGA-RY-A843-01A | TCGA-RY-A843 | NA | NA | NA | Primary | Astrocytoma G3 | Astrocytoma, IDH-mutant lower grade |  |  | DDRm2 | DDRr2 | No combined CNA | Non-codel | Mutant | Methylated | NA | 17 | IDHmut-non-codel | LGm2 | LGr3 | G-CIMP-high |
| TCGA-RY-A845-01A | TCGA-RY-A845 | NA | NA | NA | Primary | Oligoastrocytoma G2 | Astrocytoma, IDH-mutant lower grade |  |  | DDRm2 | DDRr1 | No combined CNA | Non-codel | Mutant | Methylated | NA | 20 | IDHmut-non-codel | LGm2 | LGr3 | G-CIMP-high |
| TCGA-RY-A847-01A | TCGA-RY-A847 | NA | NA | NA | Primary | Oligodendroglioma G2 | Oligodendroglioma, IDH-mutant |  |  | DDRm2 | DDRr3 | No combined CNA | Codel | Mutant | Methylated | NA | 10 | IDHmut-codel | LGm2 | LGr2 | Codel |
| TCGA-S9-A6TS-01A | TCGA-S9-A6TS | 48 | 1:DECEASED | Female | Primary | Astrocytoma G3 | Astrocytoma, IDH-mutant lower grade |  |  | DDRm2 | DDRr1 | No combined CNA | Non-codel | Mutant | Methylated | NA | 45 | IDHmut-non-codel | LGm2 | LGr3 | G-CIMP-high |
| TCGA-S9-A6TU-01A | TCGA-S9-A6TU | 38 | 0:LIVING | Male | Primary | Astrocytoma G2 | Astrocytoma, IDH-mutant lower grade |  |  | DDRm2 | DDRr1 | No combined CNA | Non-codel | Mutant | Methylated | NA | 12 | IDHmut-non-codel | LGm2 | LGr3 | G-CIMP-high |
| TCGA-S9-A6TV-01A | TCGA-S9-A6TV | 50 | 0:LIVING | Male | Primary | Oligoastrocytoma G3 | Astrocytoma, IDH-mutant lower grade |  |  | DDRm2 | DDRr1 | No combined CNA | Non-codel | Mutant | Methylated | NA | 29 | IDHmut-non-codel | LGm1 | LGr3 | G-CIMP-high |
| TCGA-S9-A6TW-01A | TCGA-S9-A6TW | 40 | 0:LIVING | Male | Primary | Oligodendroglioma G3 | Oligodendroglioma, IDH-mutant |  |  | DDRm2 | DDRr2 | No combined CNA | Codel | Mutant | Methylated | NA | 23 | IDHmut-codel | LGm3 | LGr1 | Codel |
| TCGA-S9-A6TX-01A | TCGA-S9-A6TX | 46 | 0:LIVING | Male | Primary | Oligodendroglioma G3 | Oligodendroglioma, IDH-mutant |  |  | DDRm2 | DDRr2 | No combined CNA | Codel | Mutant | Methylated | NA | 23 | IDHmut-codel | LGm3 | LGr1 | Codel |
| TCGA-S9-A6TY-01A | TCGA-S9-A6TY | 50 | 0:LIVING | Male | Primary | Oligodendroglioma G2 | Oligodendroglioma, IDH-mutant |  |  | DDRm2 | DDRr2 | No combined CNA | Codel | Mutant | Methylated | NA | 13 | IDHmut-codel | LGm3 | LGr2 | Codel |
| TCGA-S9-A6TZ-01A | TCGA-S9-A6TZ | 39 | 0:LIVING | Female | Primary | Astrocytoma G2 | Astrocytoma, IDH-mutant lower grade |  |  | DDRm2 | DDRr1 | No combined CNA | Non-codel | Mutant | Methylated | NA | 14 | IDHmut-non-codel | LGm2 | LGr3 | G-CIMP-high |
| TCGA-S9-A6U0-01A | TCGA-S9-A6U0 | 46 | 0:LIVING | Male | Primary | Astrocytoma G3 | Glioblastoma G4 | +7/-10, EGFR amp |  | DDRm1 | DDRr4 | Gain chr 7 & loss chr 10 | Non-codel | WT | Methylated | NA | 31 | IDHwt | LGm5 | LGr4 | Mesenchymal-like |
| TCGA-S9-A6U1-01A | TCGA-S9-A6U1 | 22 | 0:LIVING | Female | Primary | Astrocytoma G3 | Astrocytoma, IDH-mutant lower grade |  |  | DDRm2 | DDRr1 | No combined CNA | Non-codel | Mutant | Methylated | NA | 6 | IDHmut-non-codel | LGm2 | LGr3 | G-CIMP-high |
| TCGA-S9-A6U2-01A | TCGA-S9-A6U2 | 48 | 0:LIVING | Female | Primary | Oligodendroglioma G2 | Oligodendroglioma, IDH-mutant |  |  | DDRm2 | DDRr2 | No combined CNA | Codel | Mutant | Methylated | NA | 20 | IDHmut-codel | LGm3 | LGr1 | Codel |
| TCGA-S9-A6U5-01A | TCGA-S9-A6U5 | 33 | 0:LIVING | Male | Primary | Astrocytoma G2 | Oligodendroglioma, IDH-mutant |  |  | DDRm2 | DDRr2 | No combined CNA | Codel | Mutant | Methylated | NA | 13 | IDHmut-codel | LGm2 | LGr1 | Codel |
| TCGA-S9-A6U6-01A | TCGA-S9-A6U6 | 28 | 0:LIVING | Male | Primary | Astrocytoma G3 | Astrocytoma, IDH-mutant lower grade |  |  | DDRm2 | DDRr1 | No combined CNA | Non-codel | Mutant | Methylated | NA | 25 | IDHmut-non-codel | LGm2 | LGr3 | G-CIMP-high |
| TCGA-S9-A6U8-01A | TCGA-S9-A6U8 | 24 | 0:LIVING | Male | Primary | Astrocytoma G2 | Astrocytoma, IDH-mutant lower grade |  |  | DDRm2 | DDRr1 | No combined CNA | Non-codel | Mutant | Methylated | NA | 14 | IDHmut-non-codel | LGm2 | LGr3 | G-CIMP-high |
| TCGA-S9-A6U9-01A | TCGA-S9-A6U9 | 36 | 0:LIVING | Male | Primary | Astrocytoma G3 | Astrocytoma, IDH-mutant lower grade |  |  | DDRm2 | DDRr1 | No combined CNA | Non-codel | Mutant | Methylated | NA | 13 | IDHmut-non-codel | LGm2 | LGr3 | G-CIMP-high |
| TCGA-S9-A6UA-01A | TCGA-S9-A6UA | 66 | 1:DECEASED | Male | Primary | Astrocytoma G3 | Glioma, IDH-wildtype NOS |  |  | DDRm1 | DDRr1 | No combined CNA | Non-codel | WT | Methylated | NA | 30 | IDHwt | LGm6 | LGr4 | PA-like |
| TCGA-S9-A6UB-01A | TCGA-S9-A6UB | 52 | 0:LIVING | Male | Primary | Oligodendroglioma G2 | Oligodendroglioma, IDH-mutant |  |  | DDRm2 | DDRr2 | No combined CNA | Codel | Mutant | Methylated | NA | 20 | IDHmut-codel | LGm3 | LGr1 | Codel |
| TCGA-S9-A6WD-01A | TCGA-S9-A6WD | 58 | 0:LIVING | Male | Primary | Oligodendroglioma G3 | Oligodendroglioma, IDH-mutant |  |  | DDRm2 | DDRr2 | No combined CNA | Codel | Mutant | Methylated | NA | 20 | IDHmut-codel | LGm3 | LGr1 | Codel |
| TCGA-S9-A6WE-01A | TCGA-S9-A6WE | 34 | 0:LIVING | Male | Primary | Oligodendroglioma G2 | Oligodendroglioma, IDH-mutant |  |  | DDRm2 | DDRr2 | No combined CNA | Codel | Mutant | Methylated | NA | 9 | IDHmut-codel | LGm2 | LGr1 | Codel |
| TCGA-S9-A6WG-01A | TCGA-S9-A6WG | 31 | 0:LIVING | Male | Primary | Astrocytoma G3 | Astrocytoma, IDH-mutant lower grade |  |  | DDRm2 | DDRr4 | No combined CNA | Non-codel | Mutant | Methylated | NA | 15 | IDHmut-non-codel | LGm2 | LGr3 | G-CIMP-high |
| TCGA-S9-A6WH-01A | TCGA-S9-A6WH | 73 | 0:LIVING | Female | Primary | Oligoastrocytoma G2 | Oligodendroglioma, IDH-mutant |  |  | DDRm2 | DDRr2 | No combined CNA | Codel | Mutant | Methylated | NA | 32 | IDHmut-codel | LGm3 | LGr1 | Codel |
| TCGA-S9-A6WI-01A | TCGA-S9-A6WI | 56 | 0:LIVING | Female | Primary | Oligoastrocytoma G2 | Astrocytoma, IDH-mutant lower grade |  |  | DDRm2 | DDRr3 | No combined CNA | Non-codel | Mutant | Methylated | NA | 9 | IDHmut-non-codel | LGm1 | LGr2 | G-CIMP-high |
| TCGA-S9-A6WL-01A | TCGA-S9-A6WL | 52 | 0:LIVING | Male | Primary | Astrocytoma G3 | Oligodendroglioma, IDH-mutant |  |  | DDRm2 | DDRr3 | No combined CNA | Codel | Mutant | Methylated | NA | 31 | IDHmut-codel | LGm3 | LGr2 | Codel |
| TCGA-S9-A6WM-01A | TCGA-S9-A6WM | 59 | 0:LIVING | Female | Primary | Astrocytoma G3 | Glioblastoma G4 | +7/-10, EGFR amp |  | DDRm1 | DDRr4 | Gain chr 7 & loss chr 10 | Non-codel | WT | Unmethylated | NA | 45 | IDHwt | LGm5 | LGr4 | Mesenchymal-like |
| TCGA-S9-A6WN-01A | TCGA-S9-A6WN | 38 | 0:LIVING | Female | Primary | Astrocytoma G3 | Oligodendroglioma, IDH-mutant |  |  | DDRm2 | DDRr1 | No combined CNA | Codel | Mutant | Methylated | NA | 23 | IDHmut-codel | LGm3 | LGr4 | Codel |
| TCGA-S9-A6WO-01A | TCGA-S9-A6WO | 29 | 0:LIVING | Male | Primary | Astrocytoma G2 | Astrocytoma, IDH-mutant lower grade |  |  | DDRm2 | DDRr1 | No combined CNA | Non-codel | Mutant | Methylated | NA | 20 | IDHmut-non-codel | LGm2 | LGr3 | G-CIMP-high |
| TCGA-S9-A6WP-01A | TCGA-S9-A6WP | 42 | 0:LIVING | Male | Primary | Oligoastrocytoma G3 | Oligodendroglioma, IDH-mutant |  |  | DDRm2 | DDRr2 | No combined CNA | Codel | Mutant | Methylated | NA | 20 | IDHmut-codel | LGm3 | LGr1 | Codel |
| TCGA-S9-A6WQ-01A | TCGA-S9-A6WQ | 57 | 0:LIVING | Female | Primary | Oligoastrocytoma G2 | Astrocytoma, IDH-mutant lower grade |  |  | DDRm2 | DDRr1 | No combined CNA | Non-codel | Mutant | Methylated | NA | 26 | IDHmut-non-codel | LGm2 | LGr3 | G-CIMP-high |
| TCGA-S9-A7IQ-01A | TCGA-S9-A7IQ | 45 | 0:LIVING | Female | Primary | Oligoastrocytoma G2 | Oligodendroglioma, IDH-mutant |  |  | DDRm2 | DDRr3 | No combined CNA | Codel | Mutant | Methylated | NA | 10 | IDHmut-codel | LGm2 | LGr2 | Codel |
| TCGA-S9-A7IS-01A | TCGA-S9-A7IS | 33 | 1:DECEASED | Female | Primary | Astrocytoma G3 | Astrocytoma, IDH-mutant G4 | CDKN2A/Bdel |  | DDRm2 | DDRr4 | No combined CNA | Non-codel | Mutant | Methylated | NA | 38 | IDHmut-non-codel | LGm1 | LGr1 | G-CIMP-low |
| TCGA-S9-A7IX-01A | TCGA-S9-A7IX | 57 | 0:LIVING | Male | Primary | Astrocytoma G3 | Glioblastoma G4 | +7/-10, EGFR amp |  | DDRm1 | DDRr1 | Gain chr 7 & loss chr 10 | Non-codel | WT | Unmethylated | NA | 25 | IDHwt | LGm5 | LGr4 | Mesenchymal-like |
| TCGA-S9-A7IY-01A | TCGA-S9-A7IY | 39 | 0:LIVING | Male | Primary | Oligoastrocytoma G3 | Oligodendroglioma, IDH-mutant |  |  | DDRm2 | DDRr2 | No combined CNA | Codel | Mutant | Methylated | NA | 20 | IDHmut-codel | LGm2 | LGr3 | Codel |
| TCGA-S9-A7IZ-01A | TCGA-S9-A7IZ | 48 | 0:LIVING | Female | Primary | Astrocytoma G3 | Astrocytoma, IDH-mutant G4 | CDKN2A/Bdel |  | DDRm2 | DDRr1 | No combined CNA | Non-codel | Mutant | Methylated | NA | 17 | IDHmut-non-codel | LGm2 | LGr3 | G-CIMP-high |
| TCGA-S9-A7J0-01A | TCGA-S9-A7J0 | 30 | 0:LIVING | Female | Primary | Oligodendroglioma G3 | Astrocytoma, IDH-mutant G4 | CDKN2A/Bdel |  | DDRm2 | DDRr4 | No combined CNA | Non-codel | Mutant | Methylated | NA | 35 | IDHmut-non-codel | LGm2 | LGr1 | G-CIMP-high |
| TCGA-S9-A7J1-01A | TCGA-S9-A7J1 | 43 | 0:LIVING | Male | Primary | Oligodendroglioma G2 | Oligodendroglioma, IDH-mutant |  |  | DDRm2 | DDRr2 | No combined CNA | Codel | Mutant | Methylated | NA | 15 | IDHmut-codel | LGm3 | LGr1 | Codel |
| TCGA-S9-A7J2-01A | TCGA-S9-A7J2 | 25 | 0:LIVING | Male | Primary | Oligodendroglioma G3 | Oligodendroglioma, IDH-mutant |  |  | DDRm2 | DDRr2 | No combined CNA | Codel | Mutant | Methylated | NA | 7 | IDHmut-codel | LGm2 | LGr1 | Codel |
| TCGA-S9-A7J3-01A | TCGA-S9-A7J3 | 52 | 0:LIVING | Female | Primary | Oligodendroglioma G3 | Oligodendroglioma, IDH-mutant |  |  | DDRm2 | DDRr2 | No combined CNA | Codel | Mutant | Methylated | NA | 11 | IDHmut-codel | LGm3 | LGr1 | Codel |
| TCGA-S9-A7QW-01A | TCGA-S9-A7QW | 54 | 0:LIVING | Female | Primary | Astrocytoma G3 | Astrocytoma, IDH-mutant lower grade |  |  | DDRm2 | DDRr1 | No combined CNA | Non-codel | Mutant | Methylated | NA | 22 | IDHmut-non-codel | LGm2 | LGr3 | G-CIMP-high |
| TCGA-S9-A7QX-01A | TCGA-S9-A7QX | 36 | 0:LIVING | Female | Primary | Astrocytoma G3 | Astrocytoma, IDH-mutant lower grade |  |  | DDRm2 | DDRr1 | No combined CNA | Non-codel | Mutant | Methylated | NA | 21 | IDHmut-non-codel | LGm2 | LGr3 | G-CIMP-high |
| TCGA-S9-A7QY-01A | TCGA-S9-A7QY | 35 | 0:LIVING | Female | Primary | Oligoastrocytoma G2 | Oligodendroglioma, IDH-mutant |  |  | DDRm2 | DDRr2 | No combined CNA | Codel | Mutant | Methylated | NA | 19 | IDHmut-codel | LGm3 | LGr1 | Codel |
| TCGA-S9-A7QZ-01A | TCGA-S9-A7QZ | 41 | 0:LIVING | Male | Primary | Oligodendroglioma G2 | Oligodendroglioma, IDH-mutant |  |  | DDRm2 | DDRr2 | No combined CNA | Codel | Mutant | Methylated | NA | 28 | IDHmut-codel | LGm3 | LGr2 | Codel |
| TCGA-S9-A7R1-01A | TCGA-S9-A7R1 | 35 | 0:LIVING | Male | Primary | Oligodendroglioma G2 | Oligodendroglioma, IDH-mutant |  |  | DDRm2 | DDRr2 | No combined CNA | Codel | Mutant | Methylated | NA | 11 | IDHmut-codel | LGm3 | LGr2 | Codel |
| TCGA-S9-A7R2-01A | TCGA-S9-A7R2 | 69 | 1:DECEASED | Male | Primary | Astrocytoma G3 | Glioblastoma G4 | -0.7 |  | DDRm1 | DDRr1 | Gain chr 7 & loss chr 10 | Non-codel | WT | Unmethylated | NA | 26 | IDHwt | LGm5 | LGr4 | Mesenchymal-like |
| TCGA-S9-A7R3-01A | TCGA-S9-A7R3 | 28 | 0:LIVING | Female | Primary | Astrocytoma G2 | Astrocytoma, IDH-mutant lower grade |  |  | DDRm2 | DDRr1 | No combined CNA | Non-codel | Mutant | Methylated | NA | 23 | IDHmut-non-codel | LGm2 | LGr3 | G-CIMP-high |
| TCGA-S9-A7R4-01A | TCGA-S9-A7R4 | 46 | 0:LIVING | Male | Primary | Astrocytoma G3 | Astrocytoma, IDH-mutant lower grade |  |  | DDRm2 | DDRr1 | No combined CNA | Non-codel | Mutant | Methylated | NA | 19 | IDHmut-non-codel | LGm2 | LGr3 | G-CIMP-high |
| TCGA-S9-A7R7-01A | TCGA-S9-A7R7 | 27 | 0:LIVING | Male | Primary | Astrocytoma G2 | Astrocytoma, IDH-mutant lower grade |  |  | DDRm2 | DDRr1 | No combined CNA | Non-codel | Mutant | Methylated | NA | 20 | IDHmut-non-codel | LGm1 | LGr3 | G-CIMP-high |
| TCGA-S9-A7R8-01A | TCGA-S9-A7R8 | 44 | 1:DECEASED | Female | Primary | Astrocytoma G3 | Astrocytoma, IDH-mutant lower grade |  |  | DDRm2 | DDRr1 | No combined CNA | Non-codel | Mutant | Methylated | NA | 26 | IDHmut-non-codel | LGm2 | LGr3 | G-CIMP-high |
| TCGA-S9-A89V-01A | TCGA-S9-A89V | 70 | 0:LIVING | Male | Primary | Astrocytoma G3 | Glioma, IDH-wildtype NOS |  |  | DDRm1 | DDRr1 | No combined CNA | Non-codel | WT | Methylated | NA | 26 | IDHwt | LGm6 | LGr4 | PA-like |
| TCGA-S9-A89Z-01A | TCGA-S9-A89Z | 40 | 0:LIVING | Male | Primary | Astrocytoma G3 | Astrocytoma, IDH-mutant G4 | CDKN2A/Bdel |  | DDRm2 | DDRr1 | No combined CNA | Non-codel | Mutant | Methylated | NA | 48 | IDHmut-non-codel | LGm2 | LGr3 | G-CIMP-high |
| TCGA-TM-A7C3-01A | TCGA-TM-A7C3 | 43 | 0:LIVING | Female | Primary | Astrocytoma G3 | Glioblastoma G4 | +7/-10, EGFR amp |  | DDRm1 | DDRr1 | Gain chr 7 & loss chr 10 | Non-codel | WT | Methylated | NA | 47 | IDHwt | LGm4 | LGr4 | Classic-like |
| TCGA-TM-A7C4-01A | TCGA-TM-A7C4 | 39 | 0:LIVING | Female | Primary | Astrocytoma G2 | Astrocytoma, IDH-mutant lower grade |  |  | DDRm2 | DDRr3 | No combined CNA | Non-codel | Mutant | Methylated | NA | 30 | IDHmut-non-codel | LGm2 | LGr2 | G-CIMP-high |
| TCGA-TM-A7C5-01A | TCGA-TM-A7C5 | 30 | 0:LIVING | Male | Primary | Oligoastrocytoma G2 | Oligodendroglioma, IDH-mutant |  |  | DDRm2 | DDRr2 | No combined CNA | Codel | Mutant | Methylated | NA | 18 | IDHmut-codel | LGm3 | LGr1 | Codel |
| TCGA-TM-A7CA-01A | TCGA-TM-A7CA | 44 | 0:LIVING | Male | Primary | Astrocytoma G2 | Astrocytoma, IDH-mutant lower grade |  |  | DDRm2 | DDRr1 | No combined CNA | Non-codel | Mutant | Methylated | NA | 19 | IDHmut-non-codel | LGm2 | LGr3 | G-CIMP-high |
| TCGA-TM-A7CF-01A | TCGA-TM-A7CF | 41 | 0:LIVING | Female | Primary | Astrocytoma G2 | Astrocytoma, IDH-mutant lower grade |  |  | DDRm2 | DDRr3 | No combined CNA | Non-codel | Mutant | Methylated | WT | 21 | IDHmut-non-codel | LGm2 | LGr2 | G-CIMP-high |
| TCGA-TM-A7CF-02A | TCGA-TM-A7CF | NA | NA | NA | Recurrent | Astrocytoma G3 | Astrocytoma, IDH-mutant lower grade |  |  | DDRm2 | DDRr1 | NA | NA | NA | NA | NA | NA | NA | NA | NA | NA |
| TCGA-TM-A84B-01A | TCGA-TM-A84B | 40 | 0:LIVING | Male | Primary | Astrocytoma G3 | Glioblastoma G4 | -0.7 |  | DDRm1 | DDRr4 | Gain chr 7 & loss chr 10 | Non-codel | WT | Unmethylated | NA | 26 | IDHwt | LGm5 | LGr4 | Mesenchymal-like |
| TCGA-TM-A84C-01A | TCGA-TM-A84C | 32 | 0:LIVING | Male | Primary | Astrocytoma G2 | Glioma, IDH-wildtype NOS |  |  | NA | NA | No combined CNA | Non-codel | WT | Unmethylated | NA | 15 | IDHwt | LGm6 | LGr4 | PA-like |
| TCGA-TM-A84F-01A | TCGA-TM-A84F | 48 | 0:LIVING | Male | Primary | Astrocytoma G3 | Astrocytoma, IDH-mutant lower grade |  |  | DDRm2 | DDRr1 | No combined CNA | Non-codel | Mutant | Methylated | NA | 13 | IDHmut-non-codel | LGm1 | LGr3 | G-CIMP-high |
| TCGA-TM-A84G-01A | TCGA-TM-A84G | 54 | 0:LIVING | Female | Primary | Oligodendroglioma G3 | Oligodendroglioma, IDH-mutant |  |  | DDRm2 | DDRr2 | No combined CNA | Codel | Mutant | Methylated | NA | 29 | IDHmut-codel | LGm3 | LGr1 | Codel |
| TCGA-TM-A84H-01A | TCGA-TM-A84H | 44 | 0:LIVING | Female | Primary | Oligoastrocytoma G3 | Astrocytoma, IDH-mutant lower grade |  |  | DDRm2 | DDRr1 | No combined CNA | Non-codel | Mutant | Methylated | NA | 35 | IDHmut-non-codel | LGm2 | LGr3 | G-CIMP-high |
| TCGA-TM-A84I-01A | TCGA-TM-A84I | 30 | 0:LIVING | Male | Primary | Astrocytoma G3 | Astrocytoma, IDH-mutant lower grade |  |  | DDRm2 | DDRr1 | No combined CNA | Non-codel | Mutant | Methylated | NA | 28 | IDHmut-non-codel | LGm1 | LGr3 | G-CIMP-low |
| TCGA-TM-A84J-01A | TCGA-TM-A84J | 63 | 0:LIVING | Male | Primary | Oligodendroglioma G3 | Glioblastoma G4 | Vascular proliferation |  | DDRm1 | DDRr1 | No combined CNA | Non-codel | WT | Unmethylated | NA | 29 | IDHwt | LGm6 | LGr1 | PA-like |
| TCGA-TM-A84L-01A | TCGA-TM-A84L | 31 | 1:DECEASED | Male | Primary | Oligoastrocytoma G2 | Astrocytoma, IDH-mutant lower grade |  |  | DDRm2 | DDRr1 | No combined CNA | Non-codel | Mutant | Methylated | NA | 19 | IDHmut-non-codel | LGm2 | LGr3 | G-CIMP-high |
| TCGA-TM-A84M-01A | TCGA-TM-A84M | 40 | 0:LIVING | Male | Primary | Oligodendroglioma G3 | Oligodendroglioma, IDH-mutant |  |  | DDRm2 | DDRr2 | No combined CNA | Codel | Mutant | Methylated | NA | 18 | IDHmut-codel | LGm3 | LGr1 | Codel |
| TCGA-TM-A84O-01A | TCGA-TM-A84O | 61 | 0:LIVING | Female | Primary | Oligodendroglioma G3 | Oligodendroglioma, IDH-mutant |  |  | DDRm2 | DDRr2 | No combined CNA | Codel | Mutant | Methylated | NA | 25 | IDHmut-codel | LGm3 | LGr1 | Codel |
| TCGA-TM-A84Q-01A | TCGA-TM-A84Q | 31 | 0:LIVING | Male | Primary | Astrocytoma G2 | Astrocytoma, IDH-mutant lower grade |  |  | DDRm2 | DDRr1 | No combined CNA | Non-codel | Mutant | Methylated | NA | 21 | IDHmut-non-codel | LGm2 | LGr3 | G-CIMP-high |
| TCGA-TM-A84R-01A | TCGA-TM-A84R | 46 | 0:LIVING | Male | Primary | Oligodendroglioma G2 | Oligodendroglioma, IDH-mutant |  |  | DDRm2 | DDRr3 | No combined CNA | Codel | Mutant | Methylated | NA | 2 | IDHmut-codel | LGm2 | LGr2 | Codel |
| TCGA-TM-A84S-01A | TCGA-TM-A84S | 36 | 0:LIVING | Male | Primary | Oligodendroglioma G3 | Oligodendroglioma, IDH-mutant |  |  | DDRm2 | DDRr2 | No combined CNA | Codel | Mutant | Methylated | NA | 9 | IDHmut-codel | LGm2 | LGr1 | Codel |
| TCGA-TM-A84T-01A | TCGA-TM-A84T | 19 | 0:LIVING | Male | Primary | Oligoastrocytoma G2 | Astrocytoma, IDH-mutant lower grade |  |  | DDRm2 | DDRr1 | No combined CNA | Non-codel | Mutant | Methylated | NA | 18 | IDHmut-non-codel | LGm2 | LGr3 | G-CIMP-high |
| TCGA-TQ-A7RF-01A | TCGA-TQ-A7RF | NA | NA | NA | Primary | Oligodendroglioma G3 | Astrocytoma, IDH-mutant G4 | Necrosis |  | DDRm2 | DDRr1 | No combined CNA | Non-codel | Mutant | Unmethylated | NA | 17 | IDHmut-non-codel | LGm2 | LGr3 | G-CIMP-high |
| TCGA-TQ-A7RG-01A | TCGA-TQ-A7RG | NA | NA | NA | Primary | Oligoastrocytoma G2 | Oligodendroglioma, IDH-mutant |  |  | DDRm2 | DDRr2 | No combined CNA | Codel | Mutant | Methylated | NA | 18 | IDHmut-codel | LGm2 | LGr1 | Codel |
| TCGA-TQ-A7RH-01A | TCGA-TQ-A7RH | NA | NA | NA | Primary | Oligoastrocytoma G2 | Astrocytoma, IDH-mutant lower grade |  |  | DDRm2 | DDRr1 | No combined CNA | Non-codel | Mutant | Methylated | NA | 24 | IDHmut-non-codel | LGm2 | LGr3 | G-CIMP-high |
| TCGA-TQ-A7RI-01A | TCGA-TQ-A7RI | NA | NA | NA | Primary | Oligoastrocytoma G2 | Oligodendroglioma, IDH-mutant |  |  | DDRm2 | DDRr2 | No combined CNA | Codel | Mutant | Methylated | NA | 17 | IDHmut-codel | LGm2 | LGr1 | Codel |
| TCGA-TQ-A7RJ-01A | TCGA-TQ-A7RJ | NA | NA | NA | Primary | Oligoastrocytoma G2 | Astrocytoma, IDH-mutant lower grade |  |  | DDRm2 | DDRr1 | No combined CNA | Non-codel | Mutant | Methylated | NA | 17 | IDHmut-non-codel | LGm2 | LGr3 | G-CIMP-high |
| TCGA-TQ-A7RK-01A | TCGA-TQ-A7RK | NA | NA | NA | Primary | Oligoastrocytoma G2 | Astrocytoma, IDH-mutant lower grade |  |  | DDRm2 | DDRr1 | No combined CNA | Non-codel | Mutant | Methylated | WT | 18 | IDHmut-non-codel | LGm2 | LGr3 | G-CIMP-high |
| TCGA-TQ-A7RK-02A | TCGA-TQ-A7RK | NA | NA | NA | Recurrent | Oligoastrocytoma G2 | Astrocytoma, IDH-mutant lower grade |  |  | DDRm2 | DDRr1 | NA | NA | NA | NA | NA | NA | NA | NA | NA | NA |
| TCGA-TQ-A7RK-02B | TCGA-TQ-A7RK | NA | NA | NA | Recurrent | Oligoastrocytoma G2 | Astrocytoma, IDH-mutant lower grade |  |  | DDRm2 | DDRr1 | NA | NA | NA | NA | NA | NA | NA | NA | NA | NA |
| TCGA-TQ-A7RM-01A | TCGA-TQ-A7RM | NA | NA | NA | Primary | Oligoastrocytoma G3 | Astrocytoma, IDH-mutant G4 | Vascular proliferation |  | DDRm2 | DDRr4 | Gain chr 7 & loss chr 10 | Non-codel | Mutant | Methylated | NA | 44 | IDHmut-non-codel | LGm1 | LGr3 | G-CIMP-high |
| TCGA-TQ-A7RN-01A | TCGA-TQ-A7RN | NA | NA | NA | Primary | Oligodendroglioma G3 | Oligodendroglioma, IDH-mutant |  |  | DDRm2 | DDRr2 | No combined CNA | Codel | Mutant | Methylated | NA | 17 | IDHmut-codel | LGm2 | LGr1 | Codel |
| TCGA-TQ-A7RO-01A | TCGA-TQ-A7RO | NA | NA | NA | Primary | Oligoastrocytoma G2 | Oligodendroglioma, IDH-mutant |  |  | DDRm2 | DDRr2 | No combined CNA | Codel | Mutant | Methylated | NA | 15 | IDHmut-codel | LGm3 | LGr1 | Codel |
| TCGA-TQ-A7RP-01A | TCGA-TQ-A7RP | NA | NA | NA | Primary | Oligoastrocytoma G2 | Glioma, IDH-wildtype NOS |  |  | DDRm1 | DDRr1 | No combined CNA | Non-codel | WT | Methylated | NA | 21 | IDHwt | LGm5 | LGr4 | Mesenchymal-like |
| TCGA-TQ-A7RQ-01A | TCGA-TQ-A7RQ | NA | NA | NA | Primary | Oligodendroglioma G2 | Oligodendroglioma, IDH-mutant |  |  | DDRm2 | DDRr2 | No combined CNA | Codel | Mutant | Methylated | NA | 10 | IDHmut-codel | LGm3 | LGr1 | Codel |
| TCGA-TQ-A7RR-01A | TCGA-TQ-A7RR | NA | NA | NA | Primary | Oligoastrocytoma G3 | Astrocytoma, IDH-mutant lower grade |  |  | DDRm2 | DDRr1 | No combined CNA | Non-codel | Mutant | Unmethylated | NA | 17 | IDHmut-non-codel | LGm1 | LGr3 | G-CIMP-high |
| TCGA-TQ-A7RS-01A | TCGA-TQ-A7RS | NA | NA | NA | Primary | Oligodendroglioma G2 | Oligodendroglioma, IDH-mutant |  |  | DDRm2 | DDRr2 | No combined CNA | Codel | Mutant | Methylated | NA | 14 | IDHmut-codel | LGm2 | LGr1 | Codel |
| TCGA-TQ-A7RU-01A | TCGA-TQ-A7RU | NA | NA | NA | Primary | Oligodendroglioma G2 | Oligodendroglioma, IDH-mutant |  |  | DDRm2 | DDRr2 | No combined CNA | Codel | Mutant | Methylated | NA | 24 | IDHmut-codel | LGm3 | LGr1 | Codel |
| TCGA-TQ-A7RV-01A | TCGA-TQ-A7RV | NA | NA | NA | Primary | Astrocytoma G2 | Astrocytoma, IDH-mutant lower grade |  |  | DDRm2 | DDRr1 | No combined CNA | Non-codel | Mutant | Methylated | WT | 12 | IDHmut-non-codel | LGm2 | LGr3 | G-CIMP-high |
| TCGA-TQ-A7RV-02A | TCGA-TQ-A7RV | NA | NA | NA | Recurrent | Astrocytoma G2 | Astrocytoma, IDH-mutant lower grade |  |  | DDRm2 | DDRr1 | NA | NA | NA | NA | NA | NA | NA | NA | NA | NA |
| TCGA-TQ-A7RW-01A | TCGA-TQ-A7RW | NA | NA | NA | Primary | Oligodendroglioma G2 | Astrocytoma, IDH-mutant lower grade |  |  | DDRm2 | DDRr1 | No combined CNA | Non-codel | Mutant | Methylated | NA | 18 | IDHmut-non-codel | LGm2 | LGr3 | G-CIMP-high |
| TCGA-TQ-A8XE-01A | TCGA-TQ-A8XE | NA | NA | NA | Primary | Oligodendroglioma G2 | Astrocytoma, IDH-mutant lower grade |  |  | DDRm2 | DDRr1 | No combined CNA | Non-codel | Mutant | Methylated | WT | 31 | IDHmut-non-codel | LGm2 | LGr3 | G-CIMP-high |
| TCGA-TQ-A8XE-02A | TCGA-TQ-A8XE | NA | NA | NA | Recurrent | Oligodendroglioma G3 | Astrocytoma, IDH-mutant G4 | Vascular proliferation, necrosis |  | DDRm2 | DDRr1 | NA | NA | NA | NA | NA | NA | NA | NA | NA | NA |
| TCGA-VM-A8C8-01A | TCGA-VM-A8C8 | 50 | 0:LIVING | Female | Primary | Oligodendroglioma G2 | Astrocytoma, IDH-mutant lower grade |  |  | DDRm2 | DDRr1 | No combined CNA | Non-codel | Mutant | Unmethylated | NA | 28 | IDHmut-non-codel | LGm2 | LGr3 | G-CIMP-high |
| TCGA-VM-A8C9-01A | TCGA-VM-A8C9 | 37 | 0:LIVING | Female | Primary | Astrocytoma G2 | Glioma, IDH-wildtype NOS |  |  | DDRm1 | DDRr1 | No combined CNA | Non-codel | WT | Unmethylated | NA | 2 | IDHwt | LGm6 | LGr3 | PA-like |
| TCGA-VM-A8CA-01A | TCGA-VM-A8CA | 54 | 0:LIVING | Male | Primary | Oligodendroglioma G2 | Astrocytoma, IDH-mutant lower grade |  |  | DDRm2 | DDRr1 | No combined CNA | Non-codel | Mutant | Methylated | NA | 14 | IDHmut-non-codel | LGm2 | LGr3 | Codel |
| TCGA-VM-A8CB-01A | TCGA-VM-A8CB | 33 | 0:LIVING | Male | Primary | Oligodendroglioma G3 | Oligodendroglioma, IDH-mutant |  |  | DDRm2 | DDRr1 | No combined CNA | Codel | Mutant | Methylated | NA | 23 | IDHmut-codel | LGm2 | LGr1 | Codel |
| TCGA-VM-A8CD-01A | TCGA-VM-A8CD | 58 | 1:DECEASED | Male | Primary | Astrocytoma G3 | Glioma, IDH-wildtype NOS |  |  | DDRm1 | DDRr4 | No combined CNA | Non-codel | WT | Unmethylated | NA | 20 | IDHwt | LGm5 | LGr4 | Mesenchymal-like |
| TCGA-VM-A8CE-01A | TCGA-VM-A8CE | 25 | 0:LIVING | Male | Primary | Oligodendroglioma G2 | Oligodendroglioma, IDH-mutant |  |  | DDRm2 | DDRr2 | No combined CNA | Codel | Mutant | Methylated | NA | 8 | IDHmut-codel | LGm2 | LGr1 | Codel |
| TCGA-VM-A8CF-01A | TCGA-VM-A8CF | NA | NA | NA | Primary | Astrocytoma G3 | Astrocytoma, IDH-mutant lower grade |  |  | DDRm2 | DDRr1 | No combined CNA | Non-codel | Mutant | Methylated | NA | 27 | IDHmut-non-codel | LGm2 | LGr3 | G-CIMP-high |
| TCGA-VM-A8CH-01A | TCGA-VM-A8CH | 24 | 0:LIVING | Female | Primary | Astrocytoma G2 | Astrocytoma, IDH-mutant lower grade |  |  | DDRm2 | DDRr1 | No combined CNA | Non-codel | Mutant | Unmethylated | NA | 8 | IDHmut-non-codel | LGm2 | LGr3 | G-CIMP-high |
| TCGA-VV-A829-01A | TCGA-VV-A829 | 44 | 0:LIVING | Male | Primary | Oligoastrocytoma G3 | Oligodendroglioma, IDH-mutant |  |  | DDRm2 | DDRr2 | No combined CNA | Codel | Mutant | Methylated | NA | 22 | IDHmut-codel | LGm3 | LGr1 | Codel |
| TCGA-VV-A86M-01A | TCGA-VV-A86M | 36 | 0:LIVING | Female | Primary | Astrocytoma G3 | Astrocytoma, IDH-mutant G4 | CDKN2A/Bdel |  | DDRm2 | DDRr1 | No combined CNA | Non-codel | Mutant | Methylated | NA | 8 | IDHmut-non-codel | LGm2 | LGr3 | G-CIMP-high |
| TCGA-VW-A7QS-01A | TCGA-VW-A7QS | 35 | 0:LIVING | Female | Primary | Oligodendroglioma G3 | Oligodendroglioma, IDH-mutant |  |  | DDRm2 | DDRr2 | No combined CNA | Codel | Mutant | Methylated | NA | 27 | IDHmut-codel | LGm3 | LGr1 | Codel |
| TCGA-VW-A8FI-01A | TCGA-VW-A8FI | 66 | 1:DECEASED | Male | Primary | Astrocytoma G3 | Glioblastoma G4 | -0.7 |  | DDRm1 | DDRr4 | Gain chr 7 & loss chr 10 | Non-codel | WT | Unmethylated | NA | 37 | IDHwt | LGm4 | LGr4 | Classic-like |
| TCGA-W9-A837-01A | TCGA-W9-A837 | 47 | 0:LIVING | Male | Primary | Oligodendroglioma G2 | Oligodendroglioma, IDH-mutant |  |  | DDRm2 | DDRr3 | No combined CNA | Codel | Mutant | Methylated | NA | 18 | IDHmut-codel | LGm3 | LGr2 | Codel |
| TCGA-WH-A86K-01A | TCGA-WH-A86K | 65 | 0:LIVING | Male | Primary | Astrocytoma G2 | Astrocytoma, IDH-mutant lower grade |  |  | DDRm2 | DDRr1 | No combined CNA | Non-codel | Mutant | Methylated | NA | 28 | IDHmut-non-codel | LGm2 | LGr3 | G-CIMP-high |
| TCGA-WY-A858-01A | TCGA-WY-A858 | 32 | 0:LIVING | Female | Primary | Astrocytoma G3 | Astrocytoma, IDH-mutant lower grade |  |  | DDRm2 | DDRr1 | No combined CNA | Non-codel | Mutant | Methylated | NA | 25 | IDHmut-non-codel | LGm2 | LGr3 | G-CIMP-high |
| TCGA-WY-A859-01A | TCGA-WY-A859 | NA | NA | NA | Primary | Astrocytoma G2 | Astrocytoma, IDH-mutant lower grade |  |  | DDRm2 | DDRr3 | No combined CNA | Non-codel | Mutant | Methylated | NA | 24 | IDHmut-non-codel | LGm2 | LGr2 | G-CIMP-high |
| TCGA-WY-A85A-01A | TCGA-WY-A85A | NA | NA | NA | Primary | Astrocytoma G2 | Astrocytoma, IDH-mutant lower grade |  |  | DDRm2 | DDRr1 | No combined CNA | Non-codel | Mutant | Methylated | NA | 10 | IDHmut-non-codel | LGm2 | LGr3 | G-CIMP-high |
| TCGA-WY-A85B-01A | TCGA-WY-A85B | NA | NA | NA | Primary | Astrocytoma G2 | Astrocytoma, IDH-mutant lower grade |  |  | DDRm2 | DDRr1 | No combined CNA | Non-codel | Mutant | Methylated | NA | 7 | IDHmut-non-codel | LGm2 | LGr3 | G-CIMP-high |
| TCGA-WY-A85C-01A | TCGA-WY-A85C | NA | NA | NA | Primary | Astrocytoma G2 | Astrocytoma, IDH-mutant lower grade |  |  | DDRm2 | DDRr1 | No combined CNA | Non-codel | Mutant | Methylated | NA | 21 | IDHmut-non-codel | LGm2 | LGr3 | G-CIMP-high |
| TCGA-WY-A85D-01A | TCGA-WY-A85D | NA | NA | NA | Primary | Astrocytoma G2 | Astrocytoma, IDH-mutant lower grade |  |  | DDRm2 | DDRr1 | No combined CNA | Non-codel | Mutant | Unmethylated | NA | 29 | IDHmut-non-codel | LGm2 | LGr3 | G-CIMP-high |
| TCGA-WY-A85E-01A | TCGA-WY-A85E | NA | NA | NA | Primary | Oligoastrocytoma G2 | Astrocytoma, IDH-mutant lower grade |  |  | DDRm2 | DDRr1 | No combined CNA | Non-codel | Mutant | Methylated | NA | 41 | IDHmut-non-codel | LGm2 | LGr3 | G-CIMP-high |
