# Supplementary material for: DNA damage response profile distinguishes poor-acting gliomas with shared methylome signatures
Source: Neuro Oncol. 2025 Aug 27;28(1):117–29. doi: 10.1093/neuonc/noaf199 (PMC12962623; doi:10.1093/neuonc/noaf199)
Supplement: noaf199_Supplementary_Data [file noaf199_supplementary_data.zip › noaf199_suppl_Supplementary_Tables_S2.docx]

| **Table S2 DDR genes and pathways annotation** | | | | | |  |  |  |  |  |
| --- | --- | --- | --- | --- | --- | --- | --- | --- | --- | --- |
|  |  |  |  |  |  |  |  |  |  |  |
| **Gene** | **BER** | **NER** | **MMR** | **FA** | **HDR** | **NHEJ** | **DR** | **TLS** | **NP** | **Others** |
| AEN |  |  |  |  |  |  |  |  |  | AEN |
| ALKBH1 | ALKBH1 |  |  |  |  |  |  |  |  |  |
| ALKBH2 |  |  |  |  |  |  | ALKBH2 |  |  |  |
| ALKBH3 |  |  |  |  |  |  | ALKBH3 |  |  |  |
| APEX1 | APEX1 |  |  |  |  |  |  |  |  |  |
| APEX2 | APEX2 |  |  |  |  |  |  |  |  |  |
| APITD1 |  |  |  | APITD1 |  |  |  |  |  |  |
| APLF | APLF |  |  |  |  |  |  |  |  |  |
| APTX | APTX |  |  |  |  |  |  |  |  |  |
| ASCC3 |  |  |  |  |  |  | ASCC3 |  |  |  |
| ATM |  |  |  |  |  |  |  |  |  | ATM |
| ATR |  |  |  |  |  |  |  |  |  | ATR |
| ATRIP |  |  |  |  |  |  |  |  |  | ATRIP |
| ATRX |  |  |  |  |  |  |  |  |  | ATRX |
| BABAM1 |  |  |  |  |  |  |  |  |  | BABAM1 |
| BARD1 |  |  |  | BARD1 | BARD1 |  |  |  |  |  |
| BCAS2 |  |  |  |  |  |  |  |  |  | BCAS2 |
| BLM |  |  |  | BLM | BLM |  |  |  |  |  |
| BRCA1 |  |  |  | BRCA1 | BRCA1 |  |  |  |  |  |
| BRCA2 |  |  |  | BRCA2 | BRCA2 |  |  |  |  |  |
| BRCC3 |  |  |  |  |  |  |  |  |  | BRCC3 |
| BRE |  |  |  | BRE |  |  |  |  |  |  |
| BRIP1 |  |  |  | BRIP1 | BRIP1 |  |  |  |  |  |
| CCNH |  | CCNH |  |  |  |  |  |  |  |  |
| CDC25A |  |  |  |  |  |  |  |  |  | CDC25A |
| CDC25B |  |  |  |  |  |  |  |  |  | CDC25B |
| CDC25C |  |  |  |  |  |  |  |  |  | CDC25C |
| CDC5L |  |  |  |  |  |  |  |  |  | CDC5L |
| CDK7 |  | CDK7 |  |  |  |  |  |  |  |  |
| CETN2 |  | CETN2 |  |  |  |  |  |  |  |  |
| CHAF1A |  |  |  |  |  |  |  |  |  | CHAF1A |
| CHEK1 |  |  |  |  |  |  |  |  |  | CHEK1 |
| CHEK2 |  |  |  |  |  |  |  |  |  | CHEK2 |
| CLK2 |  |  |  |  |  |  |  |  |  | CLK2 |
| CUL3 |  | CUL3 |  |  |  |  |  |  |  |  |
| CUL4A |  | CUL4A |  |  |  |  |  |  |  |  |
| CUL5 |  | CUL5 |  |  |  |  |  |  |  |  |
| DCLRE1A |  |  |  |  |  |  |  |  |  | DCLRE1A |
| DCLRE1B |  |  |  |  |  |  |  |  |  | DCLRE1B |
| DCLRE1C |  |  |  |  |  | DCLRE1C |  |  |  |  |
| DDB1 |  | DDB1 |  |  |  |  |  |  |  |  |
| DDB2 |  | DDB2 |  |  |  |  |  |  |  |  |
| DMC1 |  |  |  |  | DMC1 |  |  |  |  |  |
| DNA2 |  |  |  |  | DNA2 |  |  |  |  |  |
| DNTT |  |  |  |  |  | DNTT |  |  |  |  |
| DUT |  |  |  |  |  |  |  |  |  | DUT |
| EID3 |  |  |  |  | EID3 |  |  |  |  |  |
| EME1 |  |  |  |  | EME1 |  |  |  |  |  |
| EME2 |  |  |  |  | EME2 |  |  |  |  |  |
| ENDOV |  |  |  |  |  |  |  |  |  | ENDOV |
| ERCC1 |  | ERCC1 |  | ERCC1 | ERCC1 |  |  |  |  |  |
| ERCC2 |  | ERCC2 |  |  |  |  |  |  |  |  |
| ERCC3 |  | ERCC3 |  |  |  |  |  |  |  |  |
| ERCC4 |  | ERCC4 |  | ERCC4 |  |  |  |  |  |  |
| ERCC5 |  | ERCC5 |  |  |  |  |  |  |  |  |
| ERCC6 |  | ERCC6 |  |  |  |  |  |  |  |  |
| ERCC8 |  | ERCC8 |  |  |  |  |  |  |  |  |
| EXO1 |  |  | EXO1 |  | EXO1 |  |  |  |  |  |
| EXO5 |  |  |  |  |  |  |  |  |  | EXO5 |
| FAAP100 |  |  |  | FAAP100 |  |  |  |  |  |  |
| FAAP20 |  |  |  | FAAP20 |  |  |  |  |  |  |
| FAAP24 |  |  |  | FAAP24 |  |  |  |  |  |  |
| FAM175A |  |  |  |  |  | FAM175A |  |  |  |  |
| FAN1 |  |  |  | FAN1 |  |  |  |  |  |  |
| FANCA |  |  |  | FANCA |  |  |  |  |  |  |
| FANCB |  |  |  | FANCB |  |  |  |  |  |  |
| FANCC |  |  |  | FANCC |  |  |  |  |  |  |
| FANCD2 |  |  |  | FANCD2 |  |  |  |  |  |  |
| FANCE |  |  |  | FANCE |  |  |  |  |  |  |
| FANCF |  |  |  | FANCF |  |  |  |  |  |  |
| FANCG |  |  |  | FANCG |  |  |  |  |  |  |
| FANCI |  |  |  | FANCI |  |  |  |  |  |  |
| FANCL |  |  |  | FANCL |  |  |  |  |  |  |
| FANCM |  |  |  | FANCM | FANCM |  |  |  |  |  |
| FEN1 | FEN1 |  |  |  | FEN1 |  |  |  |  |  |
| GADD45A |  |  |  |  |  |  |  |  |  | GADD45A |
| GADD45G |  |  |  |  |  |  |  |  |  | GADD45G |
| GEN1 |  |  |  |  | GEN1 |  |  |  |  |  |
| GTF2H1 |  | GTF2H1 |  |  |  |  |  |  |  |  |
| GTF2H2 |  | GTF2H2 |  |  |  |  |  |  |  |  |
| GTF2H3 |  | GTF2H3 |  |  |  |  |  |  |  |  |
| GTF2H4 |  | GTF2H4 |  |  |  |  |  |  |  |  |
| GTF2H5 |  | GTF2H5 |  |  |  |  |  |  |  |  |
| H2AFX |  |  |  |  | H2AFX |  |  |  |  |  |
| HELQ |  |  |  | HELQ | HELQ |  |  |  |  |  |
| HERC2 |  |  |  |  |  |  |  |  |  | HERC2 |
| HES1 |  |  |  | HES1 |  |  |  |  |  |  |
| HFM1 |  |  |  |  | HFM1 |  |  |  |  |  |
| HLTF |  |  |  |  |  |  |  | HLTF |  |  |
| HMGB1 | HMGB1 |  | HMGB1 |  |  |  |  | HMGB1 |  |  |
| HMGB2 | HMGB2 |  |  |  |  |  |  |  |  |  |
| HUS1 |  |  |  |  |  |  |  |  |  | HUS1 |
| IDH1 |  |  |  |  |  |  |  |  |  | IDH1 |
| INO80 |  |  |  |  | INO80 |  |  |  |  |  |
| KAT5 |  |  |  |  | KAT5 |  |  |  |  |  |
| LIG1 | LIG1 | LIG1 | LIG1 |  | LIG1 |  |  |  |  |  |
| LIG3 | LIG3 |  |  |  |  |  |  |  |  |  |
| LIG4 |  |  |  |  |  | LIG4 |  |  |  |  |
| MAD2L2 |  |  |  | MAD2L2 |  |  |  | MAD2L2 |  |  |
| MBD4 | MBD4 |  |  |  |  |  |  |  |  |  |
| MDC1 |  |  |  |  |  |  |  |  |  | MDC1 |
| MGMT |  |  |  |  |  |  | MGMT |  |  |  |
| MLH1 |  |  | MLH1 |  |  |  |  |  |  |  |
| MLH3 |  |  | MLH3 |  |  |  |  |  |  |  |
| MMS19 |  | MMS19 |  |  |  |  |  |  |  |  |
| MNAT1 |  | MNAT1 |  |  |  |  |  |  |  |  |
| MORF4L1 |  |  |  |  |  |  |  |  |  | MORF4L1 |
| MPG | MPG |  |  |  |  |  |  |  |  |  |
| MPLKIP |  |  |  |  |  |  |  |  |  | MPLKIP |
| MRE11A |  |  |  |  | MRE11A | MRE11A |  |  |  |  |
| MRPL40 |  |  |  |  |  |  |  |  |  | MRPL40 |
| MSH2 |  |  | MSH2 |  |  |  |  |  |  |  |
| MSH3 |  |  | MSH3 |  |  |  |  |  |  |  |
| MSH6 |  |  | MSH6 |  |  |  |  |  |  |  |
| MUS81 |  |  |  |  | MUS81 |  |  |  |  |  |
| MUTYH | MUTYH |  |  |  |  |  |  |  |  |  |
| NABP2 |  |  |  |  |  |  |  |  |  | NABP2 |
| NBN |  |  |  |  | NBN | NBN |  |  |  |  |
| NEIL1 | NEIL1 |  |  |  |  |  |  |  |  |  |
| NEIL2 | NEIL2 |  |  |  |  |  |  |  |  |  |
| NEIL3 | NEIL3 |  |  |  |  |  |  |  |  |  |
| NFATC2IP |  |  |  |  | NFATC2IP |  |  |  |  |  |
| NHEJ1 |  |  |  |  |  | NHEJ1 |  |  |  |  |
| NSMCE1 |  |  |  |  | NSMCE1 |  |  |  |  |  |
| NSMCE2 |  |  |  |  | NSMCE2 |  |  |  |  |  |
| NSMCE3 |  |  |  |  | NSMCE3 |  |  |  |  |  |
| NSMCE4A |  |  |  |  | NSMCE4A |  |  |  |  |  |
| NTHL1 | NTHL1 |  |  |  |  |  |  |  |  |  |
| NUDT1 |  |  |  |  |  |  |  |  | NUDT1 |  |
| NUDT15 |  |  |  |  |  |  |  |  | NUDT15 |  |
| NUDT18 |  |  |  |  |  |  |  |  | NUDT18 |  |
| OGG1 | OGG1 |  |  |  |  |  |  |  |  |  |
| PALB2 |  |  |  | PALB2 | PALB2 |  |  |  |  |  |
| PARG | PARG |  |  |  | PARG | PARG |  |  |  |  |
| PARP1 | PARP1 |  |  |  | PARP1 | PARP1 |  |  |  |  |
| PARP2 | PARP2 |  |  |  | PARP2 |  |  |  |  |  |
| PARP3 | PARP3 |  |  |  |  | PARP3 |  |  |  |  |
| PARP4 | PARP4 |  |  |  |  |  |  |  |  |  |
| PARPBP |  |  |  |  | PARPBP |  |  |  |  |  |
| PAXIP1 |  |  |  |  | PAXIP1 |  |  |  |  |  |
| PCNA | PCNA | PCNA | PCNA |  | PCNA |  |  | PCNA |  |  |
| PER1 |  |  |  |  |  |  |  |  |  | PER1 |
| PLK3 |  |  |  |  |  |  |  |  |  | PLK3 |
| PLRG1 |  |  |  |  |  |  |  |  |  | PLRG1 |
| PMS1 |  |  | PMS1 |  |  |  |  |  |  |  |
| PMS2 |  |  | PMS2 |  |  |  |  |  |  |  |
| PNKP | PNKP |  |  |  |  | PNKP |  |  |  |  |
| POLA1 |  |  |  |  |  |  |  |  |  | POLA1 |
| POLB | POLB |  |  |  |  | POLB |  | POLB |  |  |
| POLD1 | POLD1 | POLD1 | POLD1 |  | POLD1 |  |  |  |  |  |
| POLD2 | POLD2 | POLD2 | POLD2 |  | POLD2 |  |  |  |  |  |
| POLD3 | POLD3 | POLD3 | POLD3 |  | POLD3 |  |  |  |  |  |
| POLD4 | POLD4 | POLD4 | POLD4 |  | POLD4 |  |  |  |  |  |
| POLE | POLE | POLE |  |  |  |  |  |  |  |  |
| POLE2 | POLE2 | POLE2 |  |  |  |  |  |  |  |  |
| POLE3 | POLE3 | POLE3 |  |  |  |  |  |  |  |  |
| POLE4 | POLE4 | POLE4 |  |  |  |  |  |  |  |  |
| POLG |  |  |  |  |  |  |  |  |  | POLG |
| POLH |  |  |  |  | POLH |  |  | POLH |  |  |
| POLI |  |  |  |  |  |  |  | POLI |  |  |
| POLK | POLK |  |  |  |  |  |  | POLK |  |  |
| POLL | POLL |  |  |  |  | POLL |  |  |  |  |
| POLM |  |  |  |  |  | POLM |  | POLM |  |  |
| POLN |  |  |  |  |  |  |  | POLN |  |  |
| POLQ |  |  |  |  | POLQ |  |  | POLQ |  |  |
| PPP4C |  |  |  |  | PPP4C |  |  |  |  |  |
| PPP4R1 |  |  |  |  | PPP4R1 |  |  |  |  |  |
| PPP4R2 |  |  |  |  | PPP4R2 |  |  |  |  |  |
| PPP4R4 |  |  |  |  | PPP4R4 |  |  |  |  |  |
| PRKDC |  |  |  |  |  | PRKDC |  |  |  |  |
| PRPF19 |  |  |  |  |  |  |  |  |  | PRPF19 |
| PTEN |  |  |  |  |  |  |  |  |  | PTEN |
| RAD1 |  |  |  |  |  |  |  |  |  | RAD1 |
| RAD17 |  |  |  |  |  |  |  |  |  | RAD17 |
| RAD18 |  |  |  |  |  |  |  | RAD18 |  |  |
| RAD23A |  | RAD23A |  |  |  |  |  |  |  |  |
| RAD23B |  | RAD23B |  |  |  |  |  |  |  |  |
| RAD50 |  |  |  |  | RAD50 | RAD50 |  |  |  |  |
| RAD51 |  |  |  | RAD51 | RAD51 |  |  |  |  |  |
| RAD51B |  |  |  |  | RAD51B |  |  |  |  |  |
| RAD51C |  |  |  | RAD51C | RAD51C |  |  |  |  |  |
| RAD51D |  |  |  |  | RAD51D |  |  |  |  |  |
| RAD52 |  |  |  |  | RAD52 |  |  |  |  |  |
| RAD54B |  |  |  |  | RAD54B |  |  |  |  |  |
| RAD54L |  |  |  |  | RAD54L |  |  |  |  |  |
| RAD9A |  |  |  |  |  |  |  |  |  | RAD9A |
| RAD9B |  |  |  |  |  |  |  |  |  | RAD9B |
| RBBP8 |  |  |  |  | RBBP8 |  |  |  |  |  |
| RBX1 |  | RBX1 |  |  |  |  |  |  |  |  |
| RDM1 |  |  |  |  | RDM1 |  |  |  |  |  |
| RECQL |  |  |  |  | RECQL |  |  |  |  |  |
| RECQL4 |  |  |  |  | RECQL4 |  |  |  |  |  |
| RECQL5 |  |  |  |  | RECQL5 |  |  |  |  |  |
| REV1 |  |  |  |  |  |  |  | REV1 |  |  |
| REV3L |  |  |  |  |  |  |  | REV3L |  |  |
| RFC1 | RFC1 | RFC1 | RFC1 |  | RFC1 |  |  |  |  |  |
| RFC2 | RFC2 | RFC2 | RFC2 |  | RFC2 |  |  |  |  |  |
| RFC3 | RFC3 | RFC3 | RFC3 |  | RFC3 |  |  |  |  |  |
| RFC4 | RFC4 | RFC4 | RFC4 |  | RFC4 |  |  |  |  |  |
| RFC5 | RFC5 | RFC5 | RFC5 |  | RFC5 |  |  |  |  |  |
| RIF1 |  |  |  |  |  | RIF1 |  |  |  | RIF1 |
| RMI1 |  |  |  | RMI1 | RMI1 |  |  |  |  |  |
| RMI2 |  |  |  | RMI2 | RMI2 |  |  |  |  |  |
| RNF168 |  |  |  |  |  | RNF168 |  |  |  |  |
| RNF169 |  |  |  |  |  |  |  |  |  | RNF169 |
| RNF4 |  |  |  |  |  |  |  |  |  | RNF4 |
| RNF8 |  |  |  |  |  | RNF8 |  |  |  |  |
| RNMT |  |  |  |  |  |  |  |  |  | RNMT |
| RPA1 |  | RPA1 | RPA1 |  | RPA1 |  |  |  |  |  |
| RPA2 |  | RPA2 | RPA2 |  | RPA2 |  |  |  |  |  |
| RPA3 |  | RPA3 | RPA3 |  | RPA3 |  |  |  |  |  |
| RPA4 |  | RPA4 | RPA4 |  | RPA4 |  |  |  |  |  |
| RRM1 |  |  |  |  |  |  |  |  | RRM1 |  |
| RRM2 |  |  |  |  |  |  |  |  | RRM2 |  |
| RRM2B |  |  |  |  |  |  |  |  |  | RRM2B |
| RTEL1 |  |  |  |  | RTEL1 |  |  |  |  |  |
| SETMAR |  |  |  |  |  |  |  |  |  | SETMAR |
| SHFM1 |  |  |  |  | SHFM1 |  |  |  |  |  |
| SHPRH |  |  |  |  |  |  |  | SHPRH |  |  |
| SLX1B |  |  |  |  | SLX1B |  |  |  |  |  |
| SLX4 |  |  |  | SLX4 | SLX4 |  |  |  |  | SLX4 |
| SMARCA4 |  |  |  |  |  |  |  |  |  | SMARCA4 |
| SMARCAD1 |  |  |  |  | SMARCAD1 |  |  |  |  |  |
| SMARCC1 |  |  |  |  |  |  |  |  |  | SMARCC1 |
| SMC5 |  |  |  |  | SMC5 |  |  |  |  |  |
| SMC6 |  |  |  |  | SMC6 |  |  |  |  |  |
| SMUG1 | SMUG1 |  |  |  |  |  |  |  |  |  |
| SOX4 |  |  |  |  |  |  |  |  |  | SOX4 |
| SPO11 |  |  |  |  | SPO11 |  |  |  |  |  |
| SPRTN |  |  |  |  |  |  |  |  |  | SPRTN |
| STRA13 |  |  |  | STRA13 |  |  |  |  |  |  |
| SWI5 |  |  |  |  |  |  |  |  |  | SWI5 |
| SWSAP1 |  |  |  |  | SWSAP1 |  |  |  |  |  |
| TCEA1 |  | TCEA1 |  |  |  |  |  |  |  |  |
| TCEB1 |  | TCEB1 |  |  |  |  |  |  |  |  |
| TCEB2 |  | TCEB2 |  |  |  |  |  |  |  |  |
| TCEB3 |  | TCEB3 |  |  |  |  |  |  |  |  |
| TDG | TDG |  |  |  |  |  |  |  |  |  |
| TDP1 | TDP1 |  |  |  |  |  |  |  |  |  |
| TDP2 |  |  |  |  |  |  |  |  |  | TDP2 |
| TELO2 |  |  |  | TELO2 |  |  |  |  |  |  |
| TOP3A |  |  |  | TOP3A | TOP3A |  |  |  |  |  |
| TOP3B |  |  |  | TOP3B | TOP3B |  |  |  |  |  |
| TOPBP1 |  |  |  |  |  |  |  |  |  | TOPBP1 |
| TP53 |  |  |  |  |  |  |  |  |  | TP53 |
| TP53BP1 |  |  |  |  | TP53BP1 | TP53BP1 |  |  |  |  |
| TREX2 |  |  |  |  |  |  |  |  |  | TREX2 |
| TTK |  |  |  |  |  |  |  |  |  | TTK |
| TYMS |  |  |  |  |  |  |  |  |  | TYMS |
| UBE2A |  |  | UBE2A |  |  |  |  | UBE2A |  |  |
| UBE2B |  |  |  |  |  |  |  | UBE2B |  |  |
| UBE2N |  |  |  |  |  |  |  | UBE2N |  |  |
| UBE2T |  |  |  | UBE2T |  |  |  |  |  |  |
| UBE2V2 |  |  |  |  |  |  |  | UBE2V2 |  |  |
| UIMC1 |  |  |  |  | UIMC1 |  |  |  |  |  |
| UNG | UNG |  |  |  |  |  |  |  |  |  |
| USP1 |  |  |  | USP1 |  |  |  | USP1 |  |  |
| UVSSA |  | UVSSA |  |  |  |  |  |  |  |  |
| WDR48 |  |  |  | WDR48 |  |  |  | WDR48 |  |  |
| WEE1 |  |  |  |  |  |  |  |  |  | WEE1 |
| WRN | WRN |  |  |  | WRN |  |  |  |  |  |
| XAB2 |  | XAB2 |  |  |  |  |  |  |  |  |
| XPA |  | XPA |  |  |  |  |  |  |  |  |
| XPC |  | XPC |  |  |  |  |  |  |  |  |
| XRCC1 | XRCC1 |  |  |  |  |  |  |  |  |  |
| XRCC2 |  |  |  | XRCC2 | XRCC2 |  |  |  |  |  |
| XRCC3 |  |  |  |  | XRCC3 |  |  |  |  |  |
| XRCC4 |  |  |  |  |  | XRCC4 |  |  |  |  |
| XRCC5 |  |  |  |  |  | XRCC5 |  |  |  |  |
| XRCC6 |  |  |  |  |  | XRCC6 |  |  |  |  |
| YWHAB |  |  |  |  |  |  |  |  |  | YWHAB |
| YWHAE |  |  |  |  |  |  |  |  |  | YWHAE |
| YWHAG |  |  |  |  |  |  |  |  |  | YWHAG |
| ZSWIM7 |  |  |  |  | ZSWIM7 |  |  |  |  |  |
