# Supplementary figures and images for: DNA damage response profile distinguishes poor-acting gliomas with shared methylome signatures
Source: Neuro Oncol. 2025 Aug 27;28(1):117–29. doi: 10.1093/neuonc/noaf199 (PMC12962623; doi:10.1093/neuonc/noaf199)

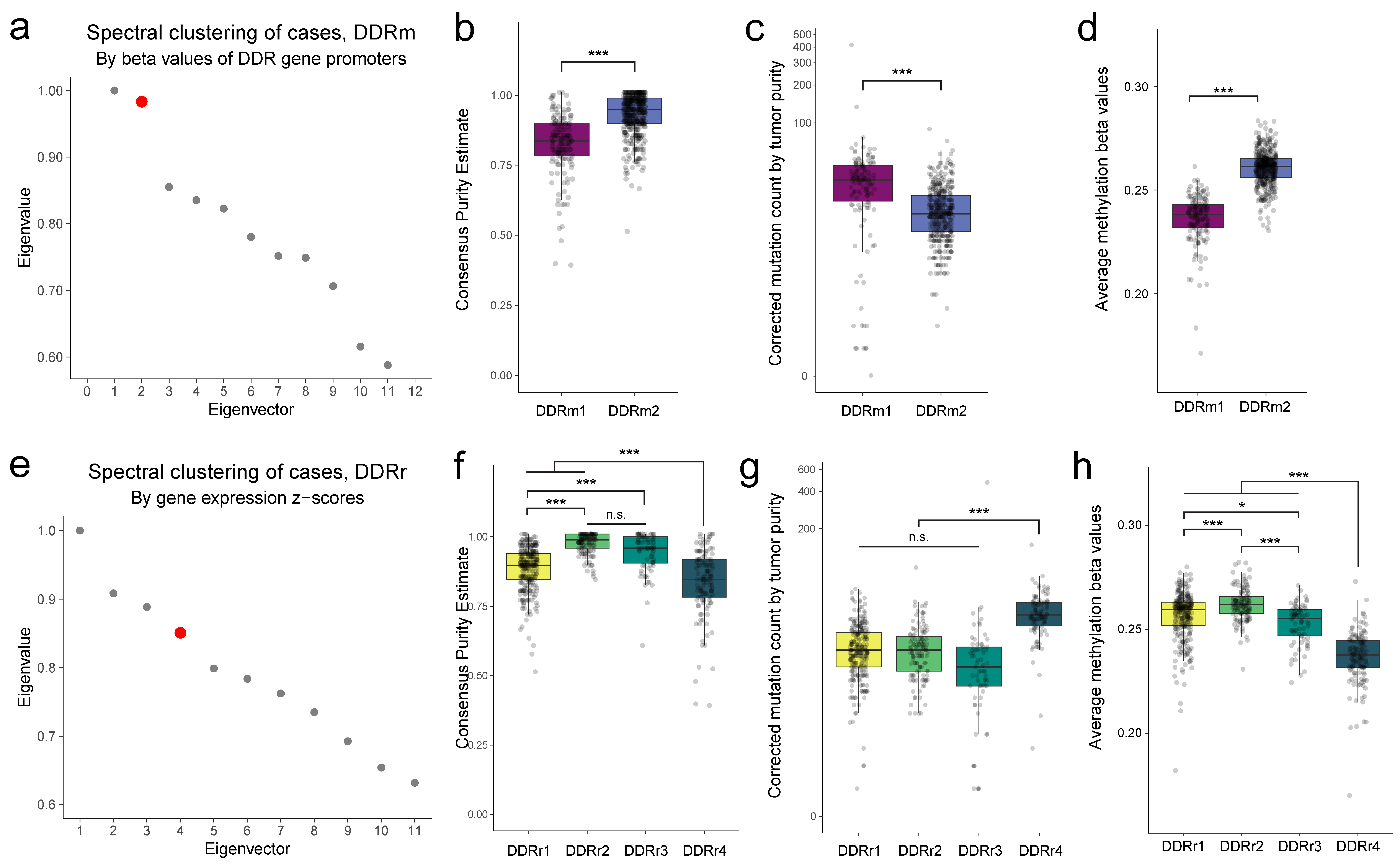

Supplement: noaf199_Supplementary_Data [file noaf199_supplementary_data.zip › noaf199_suppl_Supplementary_Figures_S3.tif]
